# Global, regional, and national burden of tetanus in 204 countries and territories, 1990–2023: a systematic analysis for the Global Burden of Disease Study 2023

**DOI:** 10.1016/j.eclinm.2026.104202

**Published:** 2026-09-28

**Authors:** GBD 2023 Tetanus Collaborators, Demelash Areda, Hyunjee Kim, Jiseung Kang, Noga Shalev, Ishu Poudyal, Min Seo Kim, Benn Sartorius, Hasan Aalruz, Nadin M.I. Abdel Razeq, Sherief Abd-Elsalam, Meriem Abdoun, Jeza Muhamad Abdul Aziz, Abdulrakib Abdulrahim, Mesfin Abebe, Olifan Zewdie Abil, Richard Gyan Aboagye, Khaled M. Aboshanab, Amr Selim Abu Lila, Nermeen Abu-Elala, Aishah Fadila Adamu, Tajudeen Adesanmi Adebisi, Ayo Stephen Adebowale, Daniel Adeyemi Adepoju, Belete Muluadam Admassie, Qorinah Estiningtyas Sakilah Adnani, Aanuoluwapo Adeyimika Afolabi, Muhammad Sohail Afzal, Saira Afzal, Gizachew Beykaso Agafari, Percival Delali Agordoh, Salome Wumpini Agordoh, Abdul-Raheem Folorunsho Ahmad, Khabir Ahmad, Muayyad M. Ahmad, Ali Ahmed, Ayman Ahmed, Gasha Salih Ahmed, Meqdad Saleh Ahmed, Mohamed Mustaf Mustaf Ahmed, Oluwasefunmi Akeju, Akil Akhtar, Muhammad Nadeem Akhtar, Olumide Odunayo Akinniyi, Karolina Akinosoglou, Damilare Michael Akintunde, Salah Al Awaidy, Muath A. Alammar, Mohammed Albashtawy, Ali M. Alfalki, Abdelazeem M. Algammal, Mohammad Daud Ali, Mohammed Usman Ali, Mohammed Alissa, Alaa A.A. Aljabali, Sameer Abdulmalik Alkubati, Mohammed Z. Allouh, Joseph Uy Almazan, Mohammed Almunef, Khaldoon Aied Alnawafleh, Mushtak Talib Salih Al-Ouqaili, Mohammad R. Alqudimat, Ahmad Rajeh Al-Qudimat, Najim Z. Alshahrani, Ali Salman Al-Shami, Abdalkarem Fedgash Alsharari, Zaid Altaany, Awais Altaf, Kassaye D. Demeke Altaye, Nelson Alvis-Guzman, Hany Aly, Raed M. Al-Zoubi, Nada A. Alzunaidy, Abhishek Anil, Mohammad Azam Ansari, Boluwatife Stephen Anuoluwa, Razique Anwer, Mosab Arafat, Muhammad Shahzad Aslam, Maha Moh'd Wahbi Atout, Alok Atreya, Matteo Augello, Usman Ayub Awan, Kemal Lemnuro Awol, Habtamu Molla Ayele, A.K.M. Azad, Ahmed Y. Azzam, Syed Babar Jamal Bacha, Riadh Badraoui, Nayereh Baghcheghi, Khlood K. Baghlaf, Senthilkumar Balakrishnan, Aleksandra Barac, Dennis Bardoe, Hiba Jawdat Barqawi, Shazia Batool, Ravi Batra, Nebiyou Simegnew Bayleyegn, Narasimha M. Beeraka, Desalegn Weldebrhan Bekru, Asnake Gashaw Belayneh, Samiun Nazrin Bente Kamal Tune, Pankaj Bhardwaj, Sonu Bhaskar, Jasvinder Singh Bhatti, Md Mehedi Hasan Bhuiyan, Can Bilgin, Trupti Bodhare, Eyob Ketema Bogale, Souad Bouaoud, Christopher Boxe, Sayed Muhammad Ata Ullah Shah Bukhari, Yasser Bustanji, Zahid A. Butt, Bindiya C.C. Gowda, Sandip Chakraborty, Imane Chamkhi, Rama Mohan Chandika, Vijay Kumar Chattu, Lam Duc Chau, Akhilanand Chaurasia, Haiyan Chen, Patrick R. Ching, Arrigo Francesco Giuseppe Cicero, Bashir Dabo, Omid Dadras, Xiaochen Dai, Fernando Pio De la Hoz, Bryan Gervais de Liyis, Sindhura Deekonda, Ivan Delgado-Enciso, Vinoth Gnana Chellaiyan Devanbu, Devananda Devegowda, Syed Masudur Rahman Dewan, Giuseppe Di Martino, Marcello Di Pumpo, Ojas Prakashbhai Doshi, Robert Kokou Dowou, Hasan Durmuş, Veli DurmuŞ, Oyewole Christopher Durojaiye, Siddhartha Dutta, Venkata Ravikanth Eddula, Martin Herbas Ekat, Rabie Adel El Arab, Muhammed Elhadi, Yasir Ahmed Mohammed Elhadi, Yasser S. El-Sayed, Chadi Eltaha, Abdelgawad Salah Eltahawy, Simachew Getaneh Endalamew, Collins Nduka Esomchi, Peter Maduabuchi Eze, Adeniyi Francis Fagbamigbe, Ayesha Fahim, Ebenezer Gezahegn Fanta, Aisha Farhana, Wafa Fatima, Olufunmilayo Ibitola Fawole, Getahun Fetensa, Tommaso Filippini, Claudio Fiorilla, Florian Fischer, Aemon Berhane Fissha, Federica Fogacci, Takeshi Fukumoto, Dominic Dormenyo Gadeka, Márió Gajdács, Ezhilkugan Ganessane, Jacopo Garlasco, Rupesh K. Gautam, Mebin George Mathew, Ali Gerami Matin, Shumiye Shiferaw Gessess, Ramy Mohamed Ghazy, Sama Ghoba, Syed Abdullah Gilani, Najam Gohar, Mahaveer Golechha, Yitayal Ayalew Goshu, Bashar Haruna Gulumbe, Sapna Gupta, Awoke Derbie Habteyohannes, Hideharu Hagiya, Teklehaimanot Gereziher Haile, Pritam Halder, Raz Mohammed Hama Salih, Mohamed H. Hamad, Fatimah Othman Hamarasool, Samer Hamidi, Abdelrahman M. Hamouda, Didem Han Yekdeş, Arief Hargono, Sara Harsini, Abdul Haseeb, Md Saquib Hasnain, Ibrahim Nagmeldin Hassan, Simon I. Hay, Zaw Myo Hein, Abdelaziz Hendy, Md Sabbir Hossain, M.D. Talib Hussain, Segun Emmanuel Ibitoye, Sumaira Idrees, Ann Oluchi Ikeabbah, Adalia Ikiroma, Olayinka Stephen Ilesanmi, Irena M. Ilic, Milena D. Ilic, Korakrit Imwattana, Md. Mojahedul Islam, Aloysia Ispriantari, Mihajlo Jakovljevic, Arshad Jamal, Jazlan Jamaluddin, Jerin James, Yovanthi Anurangi Jayasinghe, Bijay Mukesh Jeswani, Nitin Joseph, Charity Ehimwenma Joshua, Fozi Musa Jundi, Dhinesh Babu K, Divya K. L, Saltanat Kamenova, Oleksandr Kamyshnyi, Kehinde Kazeem Kanmodi, Suthanthira Kannan S, Reddy Praneeth Karnam, Tomasz M. Karpiński, Awoke Amare Kebede, Himanshu Khajuria, Sidra Khalid, Hayat Ahmad Khan, Muhammad Arslan Khan, Raees Khan, Ramsha Mushtaq Khan, Yusuf Saleem Khan, Khaled Khatab, Feriha Fatima Khidri, Daniel Gebrehawaria Kinfe, Shivakumar K.M. Kondlahalli, Aida Kondybayeva, Hamid Reza Koohestani, Vladimir Andreevich Korshunov, Kewal Krishan, Mukhtar Kulimbet, Shweta Kulshreshtha, Dewesh Kumar, Pradeep Kumar, Rakesh Kumar, Abigail Kusi Amponsah, Dian Kusuma, Ville Kytö, Chandrakant Lahariya, Yemane Leake, Caterina Ledda, Hoi Leong Lee, Hwamin Lee, Seung Won Lee, Ambreen Liaqat, Anqi Lin, Madeeha Shahzad Lodhi, José Francisco López-Gil, Peng Luo, Ellina Lytvyak, Franciele Maboni Siqueira, Mahmoud Mabrok, Monika Machoy-Rakoczy, Máté Mackei, Edward Augustine Magwe, Sasikumar Mahalingam, Nozad Hussein Mahmood, Tabarak Malik, Aseer Manilal, Kaleem Maqsood, Bernardo Alfonso Martinez-Guerra, Medha Mathur, Fernanda Penido Matozinhos, Prosanjit Mazumder, Uwakwe C. Mba, Agezegn Asegid Mekonnen, Hadush Negash Meles, Tomislav Mestrovic, Muhammad Agus Mikrajab, Bhagyashree Mishra, Nouh Saad Mohamed, Khabab Abbasher Hussien Mohamed Ahmed, Yahaya Mohammed, Ali H. Mokdad, Lorenzo Monasta, Rohith Motappa, Sumaira Mubarik, Christopher J.L. Murray, Ghulam Mustafa, Hastyar Hama Rashid Najmuldeen, Zuhair S. Natto, Biswa Prakash Nayak, Nini Asfaw Negash, Charles Richard James Newton, Cuong Tat Nguyen, Trang Nguyen, Vikram Niranjan, Ali Aahil Noorali, Emira Noumi, Chisom Adaobi Nri-Ezedi, Fred Nugen, Alex Odoom, Felix Akpojene Ogbo, Foluso Olamide Olamide Ojo, Osaretin Christabel Okonji, Michael Chinyem Okonkwo, Lenz Nwachinemere Okoro, Morufat Oluwatosin Olaitan, Atef Oreiby, Thompson Osei, Olutosin Ademola Otekunrin, Amel Ouyahia, Mayowa O. Owolabi, Kehinde Adewole Oyeniran, Ilker Ozsahin, Jagadish Rao Padubidri, Chulwoo Park, Sameena Parveen, Shankargouda Patil, Mohamed Anas Patni, Iuliia Pavlova, Zahra Zahid Piracha, Amit Porwal, Xiang Qi, Igor Radenkov, Fakher Rahim, Bilal Ahmad Rahimi, Md Sazedur Rahman, Mosiur Rahman, Jeffrey Pradeep Raj, Gunaseelan Rajendran, Mahmoud Mohammed Ramadan, Kadar Ramadhan, Lakshmi Ramamoorthy, Nemanja Rancic, Mithun Rao, Elrashdy M. Redwan, Abdul Rehman, Wajiha Rehman, Yohanes Andy Rias, Luca Ronfani, Parimal Roy, Aly M.A. Saad, Umar Saeed, Ashok Kumar Sah, Indranil Saha, Fatemeh Saheb Sharif-Askari, Narjes Saheb Sharif-Askari, Amirhossein Sahebkar, Joseph W. Sakshaug, Samreen Saleem, Mohamed A. Saleh, Malik Adebayo Sanusi, Aswini Saravanan, Brijesh Sathian, Mukesh Kumar Sathya Narayanan, Subramanian Senthilkumaran, Yashendra Sethi, Muhammad Shabbir, Disha Sanjivkumar Shah, Liloma Shah, Syed Ali Raza Shah, Shazlin Shaharudin, Sunder Sham, Mohammed Shannawaz, Amit Baran Sharangi, Nadim Sharif, Sumaira Sharif, Avimanu Sharma, Bhoopesh Kumar Sharma, Kamlesh Sharma, Ravi Kumar Sharma, Bereket Beyene Shashamo, Mahabalesh Shetty, Pavanchand Shetty, Premalatha K. Shetty, Aminu Shittu, Km Shivangi, Seyed Afshin Shorofi, Suleiman Adeiza Shuaibu, Harpreet Singh, Jasvinder A. Singh, Abhijeet Prasad Sinha, Mejdi Snoussi, Yavuzalp solak, Ahmed M. Soliman, Yizhe Song, Aayushi Sood, Anna Maria Spagnolo, Marjia Sultana, Joshua Sumankuuro, Tarun Kumar Suvvari, Ramin Tabibi, Zanan Mohammed-Ameen Taha, Jianye Tan, Rekha Thiruvengadam, Christina S. Thornton, Marcos Roberto Tovani-Palone, Nguyen Tran Minh Duc, Amritha Trikkur Madom Seetharaman, Munkhtuya Tumurkhuu, Md Jamal Uddin, Irfan Ullah, Lawan Umar, Deepadarshan Urs, Hande Uzunçıbuk, Giacomo Pietro Vigezzi, Vignesh Vikram, Tuan Vinh, Yasir Waheed, Xingxin Wang, Tanveer Wani, Nuwan Darshana Wickramasinghe, Mieszko Wieckiewicz, Camillus Abawiera Wongnaa, Yihun Miskir Miskir Wubie, Galal Yahya, Hanwen Yang, Vahit Yiğit, Naohiro Yonemoto, Fathiah Zakham, Giulia Zamagni, Aurora Zanghì, Seema Zargar, Mohammed G.M. Zeariya, Basit Zeshan, Mohamed Ali Zoromba, Abdulhakim Mussema Zuber, Ashley A. Harris, Jonathan F. Mosser, Dong Keon Yon

**Keywords:** Global burden of disease, GBD 2023, Tetanus, Vaccine-preventable diseases, Immunization

## Abstract

**Background:**

Tetanus is a life-threatening, vaccine-preventable disease. Its incidence decreased substantially following global initiatives such as the Expanded Programme on Immunization (EPI) and Maternal and Neonatal Tetanus Elimination (MNTE). Despite these efforts, tetanus elimination has not been achieved, and significant disparities in morbidity and mortality persist across various regions. Therefore, we sought to investigate the global, regional, and national burden of tetanus from 1990 to 2023, using data from the Global Burden of Disease Study (GBD) 2023.

**Methods:**

We estimated the global burden of tetanus across 204 countries and territories, seven GBD super-regions, and 21 GBD regions from 1990 to 2023. For investigation, tetanus was defined based on International Classification of Diseases (ICD)-10 codes A33–A35.0 and Z23.5 and ICD-9 codes 037–037.9, 771.3, and V03.7. The tetanus case fatality rate (CFR) was defined as the ratio of fatal tetanus cases to the total number of tetanus cases within a given sample. Incidence and prevalence of tetanus were estimated by linking cause-specific mortality estimates with modeled CFRs and disease-duration assumptions. Disease burden was quantified using age-standardized prevalence, incidence, disability-adjusted life years (DALYs), and mortality rates per 100,000 population, along with corresponding 95% uncertainty intervals (UIs) and total percent change over the study period. Estimates were stratified by age group, sex, and Socio-demographic Index (SDI) across countries and regions.

**Findings:**

Global age-standardized incidence rate of tetanus significantly decreased from 11.32 (95% UI, 7.98–16.36) per 100,000 population in 1990 to 0.70 (0.40–1.22) in 2023, representing a 93.8% decrease. The global age-standardized DALY rate also declined by 95.37% between 1990 and 2023. In 2023, the highest age-standardized DALY burden was estimated in sub-Saharan Africa (46.07 [95% UI, 25.71–77.17] per 100,000 population) and South Asia (20.47 [11.99–32.50]), whereas Southeast Asia had the highest age-standardized prevalence and incidence rates. In contrast, high-income regions and Central Europe, Eastern Europe, and Central Asia had the lowest burden of tetanus in 2023, with near-zero incidence and mortality. Tetanus burden showed a strong inverse association with SDI across all epidemiological measures. At the national level, Somalia, the Lao People's Democratic Republic, and Chad had the highest age-standardized DALY rates. Pronounced age- and sex-specific patterns were observed, with the highest rates concentrated among neonates younger than 28 days and generally higher rates in males than in females across most age groups. Although absolute rates declined over time, these demographic inequities persisted.

**Interpretation:**

Over the past three decades, global initiatives such as the EPI and MNTE have led to a substantial reduction in the burden of tetanus, bringing it to very low levels in high-income and high SDI countries and territories. Nevertheless, tetanus continues to impose a considerable burden in several regions, particularly in South Asia and sub-Saharan Africa. Because tetanus is a vaccine-preventable infection, the remaining burden and persistent inequities are largely avoidable and should be prioritized in immunization policy.

**Funding:**

Gates Foundation.

**Research in context**

**Evidence before this study**

On Jan 20, 2026, we searched PubMed for global trends in the burden of tetanus by searching the following terms ([“tetanus”] AND [“burden” OR “disability-adjusted life years (DALYs)”]) and variants. We then systematically collected and reviewed papers related to global, regional, and national trends in prevalence, incidence, mortality, and DALYs due to tetanus. Previous Global Burden of Disease (GBD) studies primarily addressed tetanus in the context of vaccine-preventable diseases or within broader infectious disease frameworks. Other studies have reported tetanus burden as part of broader pathogen-based analyses or described the global epidemiology of tetanus in the pre-COVID-19 period. Prior GBD-based research, including GBD 2019, showed that tetanus has often been assessed as one of multiple pathogens within aggregated infectious disease frameworks, underscoring the need for more focused evaluation. Previous studies have not fully characterized tetanus-specific patterns in prevalence, incidence, mortality, DALYs, and sociodemographic inequalities using GBD 2023 estimates.

**Added value of this study**

Using data from GBD 2023, this study provides an updated, tetanus-specific assessment of tetanus burden from 1990 to 2023 at global, regional, subregional, and national levels. We extend previous work by quantifying trends in prevalence, incidence, mortality, and DALYs across all age groups, characterizing age- and sex-specific patterns, and examining associations with socio-demographic development. Our analysis shows that the global burden of tetanus declined substantially over the study period but remained closely associated with socio-demographic disparities. Countries and territories with high Socio-demographic Index (SDI) achieved near-zero tetanus incidence and burden by 2023, whereas countries and territories with low SDI continued to experience a substantial burden of this preventable condition.

**Implications of all the available evidence**

The available evidence indicates that while global tetanus burden has declined markedly, elimination has not yet been achieved and substantial inequities persist. Our findings underscore the need for sustained and equitable implementation of immunization strategies, including routine childhood vaccination, maternal immunization, and booster coverage across the life course. The persistence of tetanus burden beyond childhood, including among older adults, suggests that waning immunity and inadequate booster delivery remain neglected gaps in current programs. Sustained progress toward tetanus elimination requires targeted policy efforts in low-resource and high-burden settings to strengthen health system capacity, reduce barriers to care, and ensure equitable access to vaccination.

## Introduction

The Expanded Programme on Immunization (EPI), launched by the World Health Organization (WHO) in 1974, established routine childhood vaccination as a cornerstone of global infectious disease control, with the diphtheria–tetanus–pertussis (DTP) vaccine as a central component.^1^^,^^2^ Using a multidose schedule delivered in early infancy and reinforced by maternal tetanus toxoid vaccination, EPI has substantially reduced tetanus-related morbidity and mortality worldwide. These efforts were later strengthened by the Maternal and Neonatal Tetanus Elimination (MNTE) initiative, which integrated maternal immunization with improved antenatal care and hygienic safe delivery practices.^1^^,^^3^ As a result, most high-income countries (HICs) and territories have achieved near-zero incidence of tetanus, demonstrating that sustained vaccination coverage and robust health systems can effectively eliminate this preventable disease.^4^ Tetanus remains a sensitive tracer of health system fragility, reflecting gaps in routine immunization, maternal and delivery care, wound management, and booster coverage, and continues to be highlighted within the Immunization Agenda 2030 (IA2030) as part of global concern regarding zero-dose and under-immunized populations.^5^

Despite these achievements, tetanus remains a public health concern in many low- and middle-income settings. Previous evidence indicates that the global burden of tetanus is highly uneven, with persistently high incidence and mortality in regions with low-resource settings.^6^^,^^7^ These disparities reflect structural vulnerabilities, including incomplete routine immunization, limited access to skilled birth attendance, fragile health systems, and other barriers to care.^8^ The persistence of tetanus in settings with a low Socio-demographic Index (SDI) also aligns with global concerns highlighted in the IA2030, particularly regarding zero-dose and under-immunized children who remain outside routine vaccination systems.^8^^,^^9^ Moreover, recent stagnation or decline in coverage of essential childhood vaccines, including DTP, in several countries has raised concerns about renewed susceptibility to these vaccine-preventable diseases.^1^^,^^2^ Sustaining and expanding tetanus protection beyond primary immunization schedules requires the introduction of tetanus toxoid-containing vaccine booster doses, which provide protection against waning immunity, enable catch-up vaccination among under-immunized populations, and offer a more sustainable framework for protecting mothers and neonates in low-income settings.^10^, ^11^, ^12^ The WHO recommends booster doses throughout the life course, and Gavi (Global Alliance for Vaccines and Immunization) began offering programmatic support for their introduction in 2023.^10^^,^^11^

Previous Global Burden of Diseases, Injuries, and Risk Factors Study (GBD)-based studies have assessed tetanus within the context of childhood vaccine-preventable diseases or broader infectious disease and pathogen burden frameworks.^3^^,^^4^ GBD 2019 estimates have been used to quantify the global burden of tetanus from 1990 to 2019, whereas GBD 2021 estimates have more recently been used to assess temporal trends and project its future burden.^13^^,^^14^ However, an updated tetanus-specific assessment using GBD 2023 data remains needed to comprehensively evaluate prevalence, incidence, mortality, and disability-adjusted life years (DALYs) across geographic, age, and sex strata. This study aimed to (i) evaluate global trends in tetanus burden from 1990 to 2023, (ii) describe age- and sex-specific patterns, and (iii) examine the associations between tetanus burden and the SDI, using data from GBD 2023. Our findings provide updated and comprehensive evidence on the global burden of tetanus to inform equitable and evidence-based strategies towards elimination, and to support sustained progress in strengthening routine immunization coverage and primary health systems worldwide.

## Methods

### Overview

GBD 2023 quantified the burden of 375 diseases and injuries and 88 risk factors across 204 countries and territories.^15^^,^^16^ It provides annual estimates of incidence, prevalence, years lived with disability (YLDs), deaths, years of life lost (YLLs), and DALYs from 1990 to 2023, disaggregated by age, sex, and location.^17^, ^18^, ^19^ Using data from GBD 2023, the current study assessed the global, regional, and national burden of tetanus from 1990 to 2023. The burden of tetanus was evaluated using prevalence, incidence, mortality, and DALYs, in accordance with GBD case definitions and modeling frameworks.^15^^,^^16^ This analysis followed the Guidelines for Accurate and Transparent Health Estimates Reporting (GATHER).^20^ Data sources are accessible through the Global Health Data Exchange (https://ghdx.healthdata.org/gbd-2023), and the estimates used in this study are available through the GBD Results Tool (https://vizhub.healthdata.org/gbd-results/). This study was conducted as part of the GBD Collaborator Network under the GBD protocol.

### Input data and estimation of tetanus

Tetanus estimates in GBD 2023 were calculated using an integrated estimation framework that combined multiple data sources and modeling approaches to quantify fatal and non-fatal disease burden across locations, age groups, sexes, and years.^15^^,^^16^ For cause-of-death estimation, tetanus mortality data were mapped using International Classification of Diseases (ICD)-10 codes A33–A35.0 and ICD-9 codes 037–037.9 and 771.3 within the GBD cause-of-death framework.^13^^,^^15^^,^^16^ In the GBD 2023 non-fatal estimation framework, the tetanus case-definition documentation lists ICD-10 codes A33–A35.0 and Z23.5 and ICD-9 codes 037–037.9, 771.3, and V03.7.^14^

Non-fatal burden was derived from cause-specific mortality estimates and modeled case fatality rates (CFRs), and acute prevalence was subsequently estimated from incidence using disease-duration assumptions.^15^^,^^16^ Tetanus CFRs were estimated using data obtained from systematic reviews of the tetanus CFR literature.^16^ CFRs were defined as the proportion of fatal tetanus cases among tetanus cases reported in the source studies.^13^^,^^15^ CFR data that were not sex-specific or were reported in age intervals wider than ten years were split into sex- and age-specific groups prior to modeling. Given the limited availability of location- and year-specific CFR data, global sex ratios and age patterns were estimated using all available sex- and age-specific data and used to disaggregate the aggregated observations, while propagating uncertainty throughout this process. Sex-specific adjustment factors were estimated using Meta-Regression Bayesian, Regularized, Trimmed (MR-BRT), a Bayesian meta-regression framework designed to reduce the influence of sparse or outlying data. Global age patterns were generated using Disease Modeling Meta-Regression 2.1 (DisMod-MR 2.1), a Bayesian meta-regression model that synthesizes epidemiological data across age, sex, location, and year. Finally, DisMod-MR 2.1 was fit to all available CFR data following age- and sex-disaggregation, with the Healthcare Access and Quality (HAQ) Index included as a location-level covariate and a directional prior specifying lower CFR at higher HAQ levels. This model produced location-, year-, age-, and sex-specific tetanus CFR estimates, which were subsequently used to derive incidence by dividing GBD 2023 mortality estimates by the predicted CFRs.^15^

Prevalence of acute tetanus was estimated from incidence by applying an average disease duration of 20 days, as specified in the GBD 2023 non-fatal estimation framework and informed by prior clinical and review literature.^15^^,^^16^ Because incidence was estimated on an annual scale, duration was expressed as a fraction of a year to derive the expected point prevalence of the acute tetanus health state. For neonatal tetanus, long-term impairments among survivors were modeled separately and contributed to prevalence and YLD estimates across subsequent ages. Impairment distributions followed those used for neonatal encephalopathy, with additional modeling conducted to estimate the prevalence of mild and moderate-to-severe long-term impairments among survivors across ages, sexes, years, and locations.

The cause-specific mortality estimates used in this derivation were generated as follows. Mortality data were excluded before modeling when they were implausibly high or low, inconsistent with established age or temporal patterns, or markedly discrepant from other available data from the same location or from locations with similar sociodemographic characteristics. Age-, sex-, location-, and year-specific tetanus mortality estimates were produced using the Cause of Death Ensemble modeling (CODEm) framework.^21^ CODEm is an ensemble approach that combines individual statistical models weighted by their out-of-sample predictive validity. Separate sex-specific models were developed for neonatal tetanus (under 1 year of age) and for tetanus among children and adults aged 1 year and older. Models were additionally stratified by the quality of vital registration data, with both data-rich and global models fitted for each age–sex group and subsequently combined to generate a single set of mortality estimates across all sex-age-locations. Covariates included in the tetanus CODEm framework comprised indicators of immunization coverage and sociodemographic development, including the HAQ Index, lag-distributed income (LDI), SDI, and mean years of education per capita. In the neonatal (under 1 year of age) models, additional covariates related to maternal and perinatal care were included, such as tetanus toxoid coverage, in-facility deliveries, and skilled birth attendance. To account for disruptions in routine immunization during the COVID-19 pandemic, the COVID-inclusive five-year lagged coverage of the third dose of the diphtheria–tetanus–pertussis vaccine (DTP3) was included as a covariate.^3^ Further methodological details are described in the Supplementary Appendix.

### Uncertainty estimation

Uncertainty was quantified by randomly sampling 250 draws from the parameter distributions and incorporated throughout all stages of the analysis. Final estimates were presented using the 2.5th and 97.5th percentiles of the posterior distribution, defining the 95% uncertainty interval (UI).^15^^,^^17^

### Data presentation

Tetanus burden was calculated using age-standardized rates per 100,000 population for prevalence, incidence, mortality, and DALYs. All age-standardized rates were calculated using the GBD global standard population to ensure comparability across locations and over time.^15^^,^^17^ Estimates were presented at the global, regional, and national levels, stratified by age and sex where appropriate. Countries and territories were grouped according to the GBD location hierarchy into 21 regions and seven super-regions, which reflect both geographic proximity and broad sociodemographic characteristics. The full classification is provided in the Supplementary Appendix. We also analyzed the relationship between tetanus burden and the SDI. SDI is a composite indicator defined as the geometric mean of three normalized components: total fertility rate among women younger than 25 years, mean educational attainment among individuals aged 15 years and older, and lag-distributed income per capita. SDI values range from 0 to 1, with higher values serving as a proxy for higher levels of sociodemographic development.^17^ The association between SDI and tetanus burden was assessed using linear regression models, with age-standardized tetanus rates as the outcome and SDI as the explanatory variable. Regression coefficients (β) and corresponding two-sided p-values were estimated to quantify the relationship between SDI and tetanus burden.

To further explore country-level deviations from the overall association between SDI and tetanus burden, we conducted an exploratory country-level benchmarking analysis across 204 countries and territories.^15^ For each burden metric, age-standardized rates for both sexes from 1990 to 2023 were plotted against annual country-level SDI values. Expected age-standardized burden in 2023 was estimated using a locally weighted scatterplot smoothing-fitted relationship between SDI and each burden metric across all countries. Among countries with SDI < 0.50 in 2023, the five countries with the lowest observed-to-expected ratios were highlighted for each measure.^22^, ^23^, ^24^ All statistical analyses were conducted using Python (version 3.11.11).^25^

### Role of funding sources

The funders of the study had no role in study design, data collection, data analysis, data interpretation, manuscript preparation, or the decision to submit the manuscript for publication.

## Results

In 2023, tetanus was responsible for an estimated 53,478 incident cases (95% UI, 30,198–93,877), 1,243,074 DALYs (95% UI, 748,566–1,921,250), and 19,758 deaths (95% UI, 11,735–31,048) globally (Table S1). The global age-standardized incidence rate of tetanus decreased substantially from 11.32 (95% UI, 7.98–16.36) per 100,000 population in 1990 to 0.70 (95% UI, 0.40–1.22) in 2023, representing a 93.8% decrease (95% UI, 89.0–96.3; Table 1 and Fig. 1). In 2023, Southeast Asia (2.68 [95% UI, 1.16–5.49]), Eastern sub-Saharan Africa (2.23 [95% UI, 1.10–4.71]), and Central sub-Saharan Africa (1.64 [95% UI, 0.39–4.89]) per 100,000 population had the highest age-standardized incidence rates of tetanus. In contrast, the lowest age-standardized incidence rates were estimated in Central Europe, Eastern Europe, and Central Asia (0.0046 [95% UI, 0.0030–0.0117]) per 100,000 population, followed by the High-income region (0.0130 [95% UI, 0.0082–0.0222]) in 2023. At the national level, the highest age-standardized incidence rates in 2023 were observed in Lao People's Democratic Republic (18.72 [95% UI, 7.72–35.20]) per 100,000 population, followed by Somalia (15.21 [95% UI, 4.11–46.30]) and Micronesia (Federated States of) (7.35 [95% UI, 0.39–19.57]; Table S2).

**Table 1:** Age-standardized prevalence and DALY rates per 100,000 (95% UI) and total percentage change in GBD super region in 1990 and 2023.

Region	Age-standardized prevalence rate per 100,000 (95% UI)			Age-standardized DALY rate per 100,000 (95% UI)		
	1990	2023	Total percentage change (%)	1990	2023	Total percentage change (%)
Global	2.5032 (1.9051–3.1237)	0.4860 (0.3526–0.6290)	−80.6 (−82.9 to −78.1)	386.5975 (278.4726–515.8872)	17.8834 (10.8607–27.3813)	−95.4 (−97.1 to −92.6)
Central Europe, Eastern Europe, and Central Asia	0.0060 (0.0046–0.0078)	0.0011 (0.0007–0.0018)	−81.0 (−88.2 to −69.7)	1.0416 (0.8000–1.2859)	0.0472 (0.0353–0.0614)	−95.5 (−96.6 to −93.6)
Central Asia	0.0043 (0.0021–0.0067)	0.0013 (0.0008–0.0021)	−68.5 (−80.7 to −49.1)	0.7521 (0.2616–1.0151)	0.0549 (0.0197–0.0748)	−92.7 (−94.8 to −90.0)
Central Europe	0.0072 (0.0047–0.0093)	0.0008 (0.0004–0.0013)	−89.5 (−94.2 to −80.3)	1.2584 (0.7850–1.5275)	0.0453 (0.0263–0.0700)	−96.4 (−97.5 to −94.3)
Eastern Europe	0.0056 (0.0040–0.0082)	0.0012 (0.0007–0.0020)	−78.3 (−86.9 to −67.0)	0.9665 (0.7438–1.2364)	0.0406 (0.0296–0.0595)	−95.8 (−97.2 to −93.6)
High-income	0.0149 (0.0119–0.0189)	0.0028 (0.0017–0.0042)	−81.3 (−86.8 to −74.7)	0.8442 (0.6643–1.0108)	0.0535 (0.0377–0.0696)	−93.7 (−95.5 to −90.7)
Australasia	0.0036 (0.0017–0.0073)	0.0007 (0.0003–0.0013)	−80.2 (−89.4 to −63.1)	0.0666 (0.0350–0.0944)	0.0045 (0.0025–0.0080)	−93.2 (−95.9 to −88.4)
High-income Asia Pacific	0.0349 (0.0269–0.0442)	0.0075 (0.0048–0.0110)	−78.5 (−84.9 to −72.4)	1.6641 (1.2000–2.1634)	0.0978 (0.0628–0.1329)	−94.1 (−96.2 to −91.1)
High-income North America	0.0026 (0.0016–0.0042)	0.0015 (0.0009–0.0025)	−41.7 (−58.4 to −26.3)	0.1722 (0.1225–0.2295)	0.0279 (0.0185–0.0418)	−83.8 (−89.1 to −73.9)
Southern Latin America	0.0642 (0.0498–0.0903)	0.0063 (0.0033–0.0120)	−90.2 (−94.1 to −84.4)	3.8544 (2.9166–4.9768)	0.0919 (0.0554–0.1856)	−97.6 (−98.6 to −95.3)
Western Europe	0.0094 (0.0068–0.0122)	0.0014 (0.0009–0.0022)	−85.0 (−90.3 to −76.6)	0.6659 (0.4519–0.8405)	0.0516 (0.0312–0.0757)	−92.3 (−94.9 to −87.4)
Latin America and Caribbean	0.3441 (0.2635–0.4286)	0.0682 (0.0452–0.0995)	−80.2 (−83.8 to −75.7)	40.8847 (33.4737–49.0454)	1.6337 (1.0743–2.5555)	−96.0 (−97.3 to −93.2)
Andean Latin America	0.3467 (0.2686–0.4513)	0.1029 (0.0798–0.1380)	−70.3 (−76.1 to −64.3)	46.4705 (30.3539–65.2108)	1.4920 (0.8424–2.6931)	−96.8 (−98.4 to −94.2)
Caribbean	0.5200 (0.3628–0.7206)	0.2792 (0.2072–0.3742)	−46.3 (−62.1 to −25.6)	88.5160 (42.9311–158.4639)	8.8985 (3.6126–19.4786)	−89.9 (−96.3 to −71.0)
Central Latin America	0.2216 (0.1707–0.2774)	0.0315 (0.0178–0.0509)	−85.8 (−89.9 to −81.0)	29.8887 (23.5469–34.2144)	0.6312 (0.4212–1.0357)	−97.9 (−98.6 to −96.4)
Tropical Latin America	0.4343 (0.3284–0.5521)	0.0534 (0.0298–0.0856)	−87.7 (−91.3 to −83.8)	41.6995 (34.2537–51.4715)	1.0667 (0.7703–1.7292)	−97.4 (−98.2 to −96.3)
North Africa and Middle East	0.4917 (0.3512–0.7096)	0.1810 (0.1337–0.2383)	−63.2 (−72.2 to −53.0)	132.6086 (58.6953–225.8487)	6.4437 (3.3798–11.7830)	−95.1 (−97.9 to −86.6)
South Asia	4.8933 (3.7242–6.0367)	0.8995 (0.6619–1.1483)	−81.6 (−83.5 to −79.7)	953.4989 (757.8554–1206.8020)	20.4670 (11.9923–32.4995)	−97.9 (−98.7 to −96.5)
Southeast Asia, East Asia, and Oceania	3.8823 (2.9284–4.9361)	0.5441 (0.4007–0.7105)	−86.0 (−87.7 to −84.1)	270.2856 (171.4542–393.2439)	11.5883 (6.6174–19.1465)	−95.7 (−97.6 to −92.2)
East Asia	3.3166 (2.4727–4.2079)	0.2572 (0.1704–0.3774)	−92.2 (−94.1 to −90.3)	131.7197 (82.5072–198.5849)	0.7816 (0.4261–1.3113)	−99.4 (−99.7 to −98.8)
Oceania	0.0823 (0.0416–0.1834)	0.0633 (0.0255–0.1642)	−23.1 (−60.1 to 81.6)	4.0024 (1.4649–8.8212)	2.2059 (0.6224–6.9851)	−44.9 (−81.5 to 25.0)
Southeast Asia	5.3990 (4.0138–7.2624)	1.0849 (0.8167–1.4222)	−79.9 (−83.8 to −75.1)	578.6584 (271.9176–930.6941)	29.5945 (15.4937–52.3252)	−94.9 (−97.7 to −88.6)
Sub-Saharan Africa	2.4184 (1.7322–3.3084)	0.6596 (0.4608–0.8740)	−72.7 (−77.9 to −66.5)	516.0565 (241.2869–778.8970)	46.0741 (25.7120–77.1697)	−91.1 (−95.3 to −80.9)
Central Sub-Saharan Africa	0.7083 (0.4150–1.2314)	0.3339 (0.2244–0.5128)	−52.9 (−67.0 to −33.1)	199.4168 (96.8163–340.8515)	42.0874 (18.3371–81.3981)	−78.9 (−90.3 to −55.9)
Eastern Sub-Saharan Africa	2.5183 (1.7517–3.5737)	0.6668 (0.4678–0.8850)	−73.5 (−80.1 to −64.9)	804.7214 (384.8621–1214.1290)	66.5141 (37.5382–115.9018)	−91.7 (−95.7 to −82.3)
Southern Sub-Saharan Africa	0.2235 (0.1670–0.3000)	0.1161 (0.0962–0.1433)	−48.0 (−56.3 to −37.6)	16.3020 (9.3953–25.4428)	3.8742 (2.4156–6.2978)	−76.2 (−87.4 to −54.4)
Western Sub-Saharan Africa	3.4141 (2.4271–4.5511)	0.8549 (0.5975–1.1291)	−75.0 (−78.8 to −70.8)	438.9143 (198.7314–703.6900)	36.7503 (19.5472–61.3866)	−91.6 (−95.8 to −81.2)

DALY = disability-adjusted life year; GBD = Global Burden of Diseases, Injuries, and Risk Factors Study; UI = uncertainty interval.

**Fig. 1:**
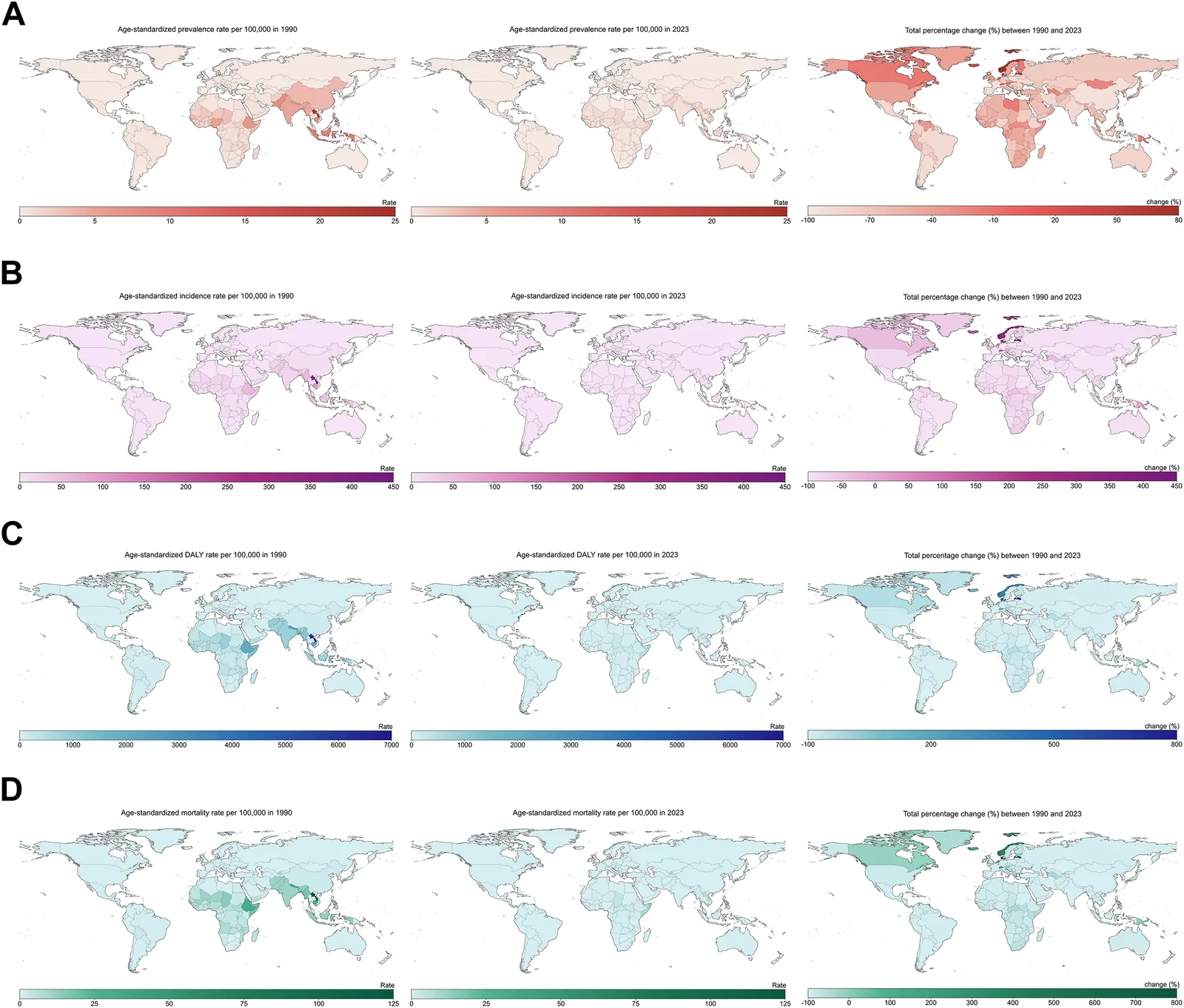
World maps of age-standardized (A) prevalence, (B) incidence rate, (C) DALY, and (D) mortality rates (per 100,000 population) in 1990 and 2023, and the total percentage change (%). DALY = disability-adjusted life year.

Similarly, the global age-standardized DALY rate declined from 386.60 (95% UI, 278.47–515.89) per 100,000 population in 1990 to 17.88 (95% UI, 10.86–27.38) in 2023, representing a 95.37% decrease (95% UI, 92.62–97.08). In 2023, sub-Saharan Africa had the highest age-standardized DALY rate (46.07 [95% UI, 25.71–77.17]) per 100,000 population, followed by South Asia (20.47 [95% UI, 11.99–32.50]), and Southeast Asia, East Asia, and Oceania (11.59 [95% UI, 6.62–19.15]). The lowest age-standardized DALY rate was estimated in Central Europe, Eastern Europe, and Central Asia (0.0472 [95% UI, 0.0353–0.0614]) per 100,000 population and HICs (0.0535 [95% UI, 0.0377–0.0696]). At the national level, Somalia (448.71 [95% UI, 137.66–978.12]) per 100,000 population, the Lao People's Democratic Republic (182.22 [95% UI, 81.58–325.50]), and Chad (172.80 [95% UI, 69.60–348.24]) showed the highest age-standardized DALY rate of tetanus. Conversely, Mongolia had the lowest age-standardized DALY rate in 2023 (0.0005 [95% UI, 0.0003–0.0009] per 100,000 population). From 1990 to 2023, Portugal showed the largest reduction in age-standardized DALY rates (−99.84% [95% UI, −99.93 to −99.48]; Tables 2 and Table S3–S7). Age-standardized YLL rate declined markedly across all regions from 1990 to 2023, reflecting substantial reductions in tetanus mortality (Figure S1). Age-standardized YLD rate remained comparatively low throughout the study period and decreased over time. Overall, the decline in age-standardized DALY rate was largely driven by reductions in premature mortality (Tables S8 and S9).

**Table 2:** Age-standardized incidence and mortality rate per 100,000 (95% UI) and total percentage change in GBD super region in 1990 and 2023.

Region	Age-standardized incidence rate per 100,000 (95% UI)			Age-standardized mortality rate per 100,000 (95% UI)		
	1990	2023	Total percentage change (%)	1990	2023	Total percentage change (%)
Global	11.3168 (7.9785–16.3575)	0.6995 (0.4042–1.2198)	−93.8 (−96.3 to −89.0)	4.9356 (3.5865–6.3802)	0.2659 (0.1602–0.4091)	−94.6 (−96.7 to −91.3)
Central Europe, Eastern Europe, and Central Asia	0.0872 (0.0670–0.1196)	0.0046 (0.0030–0.0117)	−94.7 (−96.2 to −86.6)	0.0386 (0.0305–0.0483)	0.0016 (0.0012–0.0021)	−95.9 (−96.8 to −94.2)
Central Asia	0.0537 (0.0173–0.0969)	0.0050 (0.0013–0.0102)	−90.7 (−94.2 to −85.0)	0.0206 (0.0077–0.0283)	0.0017 (0.0005–0.0026)	−91.5 (−94.5 to −87.2)
Central Europe	0.1184 (0.0739–0.1549)	0.0052 (0.0029–0.0089)	−95.6 (−97.4 to −87.9)	0.0524 (0.0305–0.0643)	0.0018 (0.0011–0.0026)	−96.6 (−97.6 to −94.4)
Eastern Europe	0.0752 (0.0552–0.1070)	0.0039 (0.0024–0.0109)	−94.8 (−97.0 to −88.2)	0.0339 (0.0260–0.0446)	0.0013 (0.0010–0.0021)	−96.0 (−97.4 to −94.0)
High-income	0.1343 (0.1057–0.1750)	0.0130 (0.0082–0.0222)	−90.3 (−94.0 to −82.0)	0.0274 (0.0218–0.0332)	0.0023 (0.0016–0.0031)	−91.5 (−94.1 to −86.9)
Australasia	0.0553 (0.0254–0.1187)	0.0049 (0.0022–0.0139)	−91.1 (−95.3 to −82.1)	0.0040 (0.0022–0.0057)	0.0003 (0.0002–0.0005)	−92.0 (−95.3 to −86.3)
High-income Asia Pacific	0.2257 (0.1620–0.3364)	0.0347 (0.0193–0.0541)	−84.6 (−92.5 to −73.9)	0.0376 (0.0280–0.0507)	0.0037 (0.0022–0.0051)	−90.0 (−94.8 to −84.7)
High-income North America	0.0181 (0.0119–0.0266)	0.0031 (0.0018–0.0072)	−82.7 (−89.4 to −63.6)	0.0058 (0.0039–0.0082)	0.0010 (0.0006–0.0015)	−83.4 (−89.5 to −70.8)
Southern Latin America	0.6387 (0.4443–1.0448)	0.0209 (0.0097–0.0896)	−96.7 (−98.4 to −86.7)	0.1161 (0.0869–0.1654)	0.0028 (0.0017–0.0061)	−97.6 (−98.6 to −94.9)
Western Europe	0.1267 (0.0900–0.1653)	0.0085 (0.0052–0.0154)	−93.3 (−95.7 to −85.0)	0.0294 (0.0211–0.0367)	0.0026 (0.0016–0.0038)	−91.2 (−94.3 to −85.6)
Latin America and Caribbean	1.9279 (1.5654–2.4489)	0.1204 (0.0758–0.2025)	−93.8 (−96.1 to −88.6)	0.7659 (0.6434–0.9020)	0.0338 (0.0250–0.0472)	−95.6 (−96.7 to −93.5)
Andean Latin America	2.4091 (1.5908–3.5877)	0.1061 (0.0528–0.2460)	−95.6 (−98.1 to −90.8)	0.9327 (0.6188–1.3172)	0.0360 (0.0195–0.0658)	−96.1 (−98.2 to −93.1)
Caribbean	4.9091 (2.6427–8.6564)	0.6876 (0.3101–1.4721)	−86.0 (−94.1 to −63.0)	1.1574 (0.6262–1.8990)	0.1308 (0.0638–0.2417)	−88.7 (−94.8 to −73.0)
Central Latin America	1.0588 (0.8070–1.3330)	0.0409 (0.0213–0.1024)	−96.1 (−98.0 to −90.6)	0.6045 (0.4653–0.7223)	0.0161 (0.0102–0.0282)	−97.3 (−98.3 to −95.2)
Tropical Latin America	2.0678 (1.6449–2.8320)	0.0820 (0.0515–0.1818)	−96.0 (−97.5 to −90.7)	0.8303 (0.6761–1.0490)	0.0297 (0.0212–0.0475)	−96.4 (−97.5 to −94.8)
North Africa and Middle East	3.5227 (1.6776–6.5173)	0.2164 (0.1071–0.4757)	−93.9 (−97.2 to −83.1)	1.7595 (0.8598–2.7368)	0.0948 (0.0512–0.1620)	−94.6 (−97.4 to −87.0)
South Asia	23.3766 (17.3914–31.3912)	0.7231 (0.3644–1.3370)	−96.9 (−98.2 to −94.7)	12.1504 (9.5994–15.1653)	0.2987 (0.1689–0.4870)	−97.5 (−98.5 to −96.0)
Southeast Asia, East Asia, and Oceania	13.7699 (8.5288–20.9080)	0.9207 (0.4603–1.8203)	−93.3 (−96.6 to −86.2)	3.8386 (2.3681–5.5597)	0.2064 (0.1082–0.3512)	−94.6 (−97.0 to −90.4)
East Asia	7.3339 (4.7214–11.0469)	0.0969 (0.0409–0.1897)	−98.7 (−99.4 to −96.8)	1.7390 (1.1436–2.4758)	0.0174 (0.0082–0.0325)	−99.0 (−99.5 to −97.9)
Oceania	0.9075 (0.2255–2.8909)	0.7702 (0.1362–2.7472)	−15.1 (−75.1 to 181.1)	0.1788 (0.0467–0.4449)	0.1397 (0.0308–0.4718)	−21.9 (−71.6 to 90.4)
Southeast Asia	30.4712 (14.6317–53.4297)	2.6819 (1.1586–5.4949)	−91.2 (−96.3 to −77.3)	9.0864 (4.4899–15.0659)	0.6115 (0.2824–1.1091)	−93.3 (−96.9 to −83.0)
Sub-Saharan Africa	14.8309 (7.2087–25.0851)	1.6122 (0.7978–3.2632)	−89.1 (−94.8 to −74.9)	7.8753 (3.7619–12.2633)	0.8567 (0.4554–1.5497)	−89.1 (−94.3 to −76.9)
Central Sub-Saharan Africa	6.7776 (2.1746–15.1486)	1.6377 (0.3911–4.8877)	−75.8 (−89.8 to −41.6)	4.4031 (1.4614–8.4588)	0.9936 (0.2910–2.1468)	−77.4 (−89.3 to −47.0)
Eastern Sub-Saharan Africa	22.5505 (11.0435–37.2177)	2.2335 (1.1003–4.7062)	−90.1 (−95.5 to −76.6)	12.2402 (5.9970–18.7099)	1.2310 (0.6615–2.2140)	−89.9 (−94.8 to −78.6)
Southern Sub-Saharan Africa	0.9920 (0.5191–1.8230)	0.2595 (0.1282–0.6146)	−73.8 (−88.0 to −37.8)	0.3096 (0.1773–0.5197)	0.0807 (0.0472–0.1383)	−73.9 (−86.6 to −46.7)
Western Sub-Saharan Africa	13.3153 (6.2101–23.9037)	1.3464 (0.6726–2.4587)	−89.9 (−95.3 to −76.7)	6.6285 (3.1712–10.7004)	0.6636 (0.3471–1.1332)	−90.0 (−95.2 to −77.1)

GBD = Global Burden of Diseases, Injuries, and Risk Factors Study; UI = uncertainty interval.

Across GBD regions, the burden of tetanus declined substantially from 1990 to 2023 (Fig. 2). Southeast Asia had the highest age-standardized prevalence and incidence rates during the study period, whereas HICs consistently showed the lowest age-standardized DALY rates. South Asia had the highest age-standardized DALY rate in 1990; however, by 2023, Eastern sub-Saharan Africa had the highest DALY rate. In Southeast Asia, the age-standardized incidence rate decreased from 30.47 (95% UI, 14.63–53.43) per 100,000 population in 1990 to 2.68 (95% UI, 1.16–5.50) in 2023. In Eastern sub-Saharan Africa, the age-standardized DALY rate declined from 804.72 (95% UI, 384.86–1214.13) per 100,000 population in 1990 to 66.51 (95% UI, 37.54–115.90) in 2023. Similarly, in South Asia, the age-standardized DALY rate decreased markedly from 953.50 (95% UI, 757.86–1206.80) per 100,000 population in 1990 to 20.47 (95% UI, 11.99–32.50) in 2023. Eastern sub-Saharan Africa had the highest age-standardized mortality rate throughout the study period (Fig. 3). In contrast, Australasia consistently had the lowest age-standardized DALY and mortality rates throughout the study period.

**Fig. 2:**
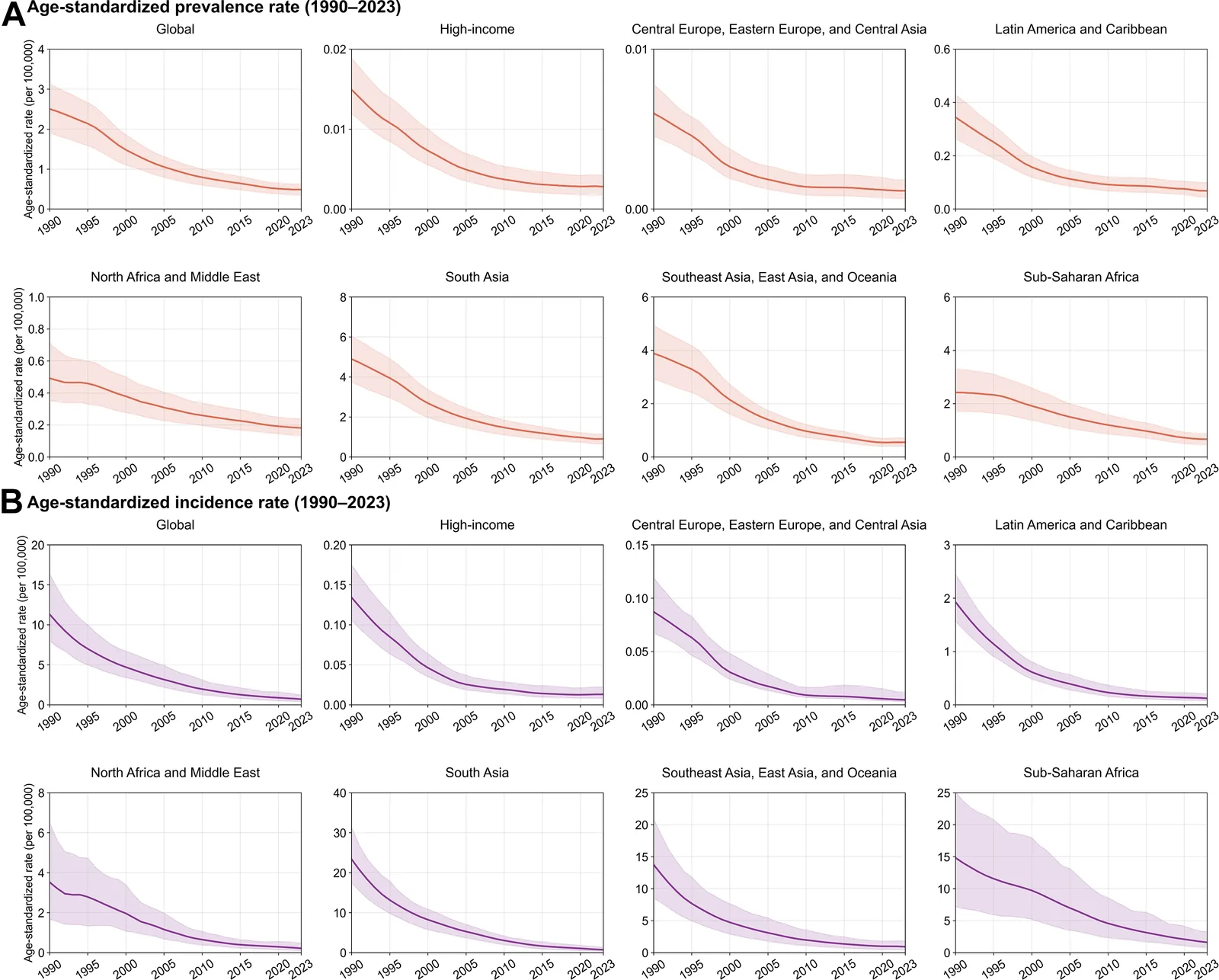

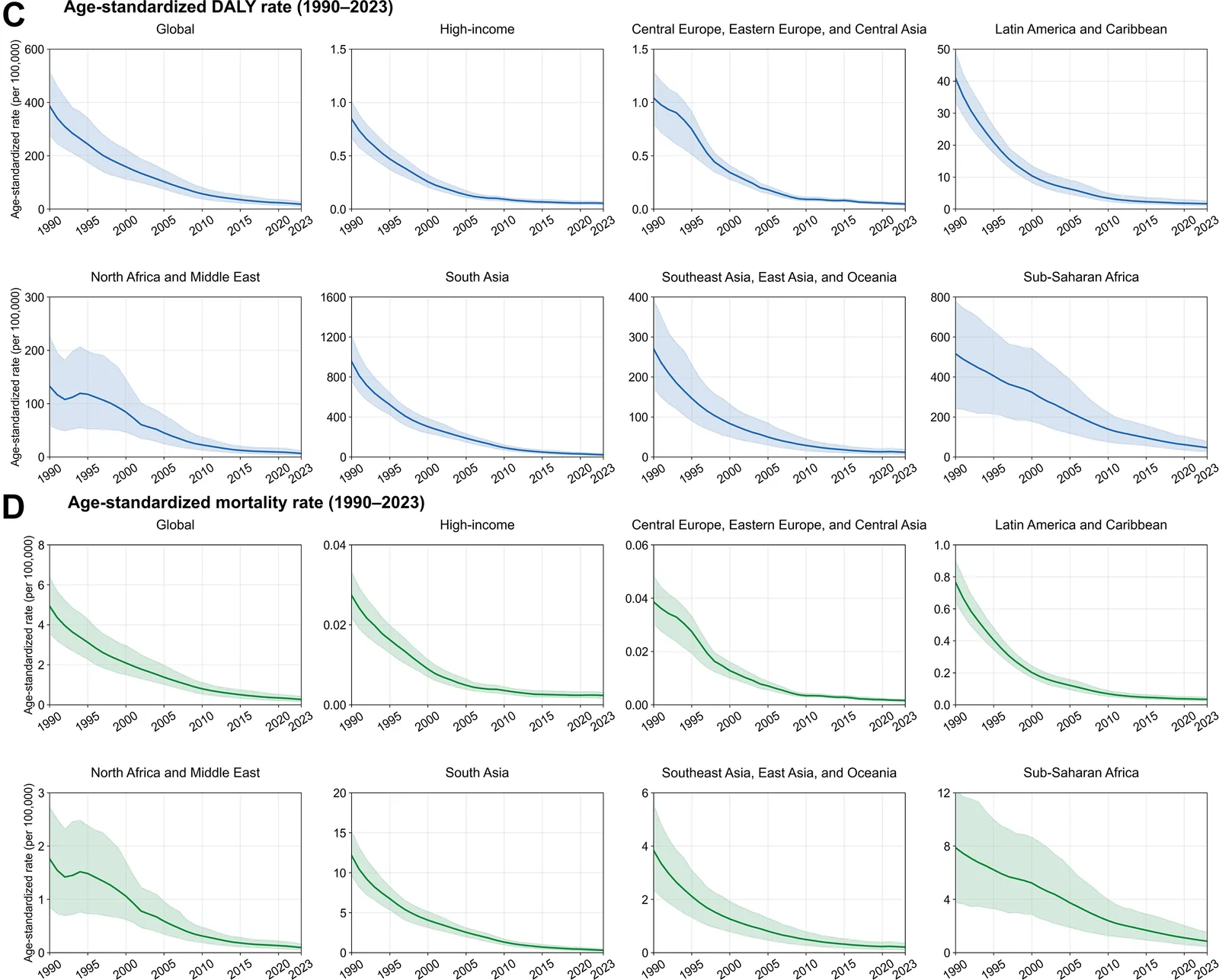
Trend in age-standardized prevalence (A), incidence (B), DALY (C), and mortality (D) rates (per 100,000 population) by GBD super region from 1990 to 2023. DALY = disability-adjusted life year; GBD = Global Burden of Diseases, Injuries, and Risk Factors Study.

**Fig. 3:**
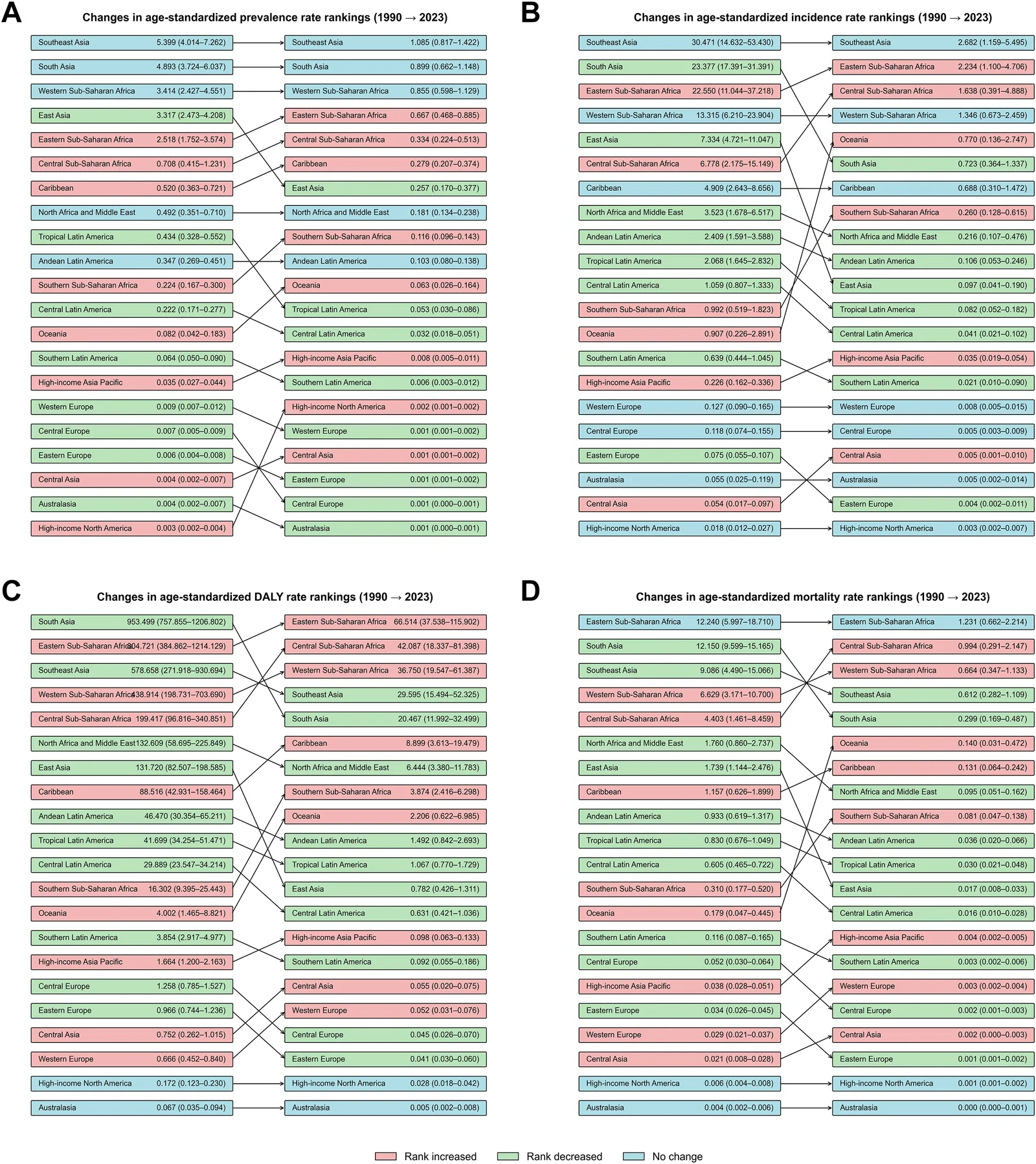
Changes in ranking in age-standardized (A) prevalence, (B) incidence rate, (C) DALY, and (D) mortality from 1990 to 2023. Regions are connected by lines between 1990 and 2023 to illustrate changes in rank over time. Box colors indicate changes in rank between the two time points: red represents increased rank, green represents decreased rank, and blue represents no change in rank. Values within boxes represent age-standardized rates per 100,000 population, with numbers in parentheses indicating 95% uncertainty intervals. DALY = disability-adjusted life year; UI = uncertainty interval.

The relationships between SDI and the age-standardized prevalence, incidence, mortality, and DALY rates of tetanus across 21 GBD regions from 1990 to 2023 are presented in Fig. 4. The results show a persistent gap in tetanus burden in low and high SDI regions throughout the study period (1990–2023). Across all regions and epidemiological measures, tetanus burden was strongly and consistently inversely associated with SDI (P < 0.01), with higher burden observed in lower-SDI settings (Figure S2). This gradient was particularly pronounced in South Asia and Western, Eastern, and Central sub-Saharan Africa, where prevalence, incidence, mortality, and DALY rates remained substantially higher than in high-SDI regions. In contrast, high-SDI regions consistently showed near-zero prevalence, incidence, and mortality and markedly lower age-standardized DALY rates throughout the study period (Table S10). Inverse associations between SDI and disease burden were similarly shown across 204 countries (Figure S3).

**Fig. 4:**
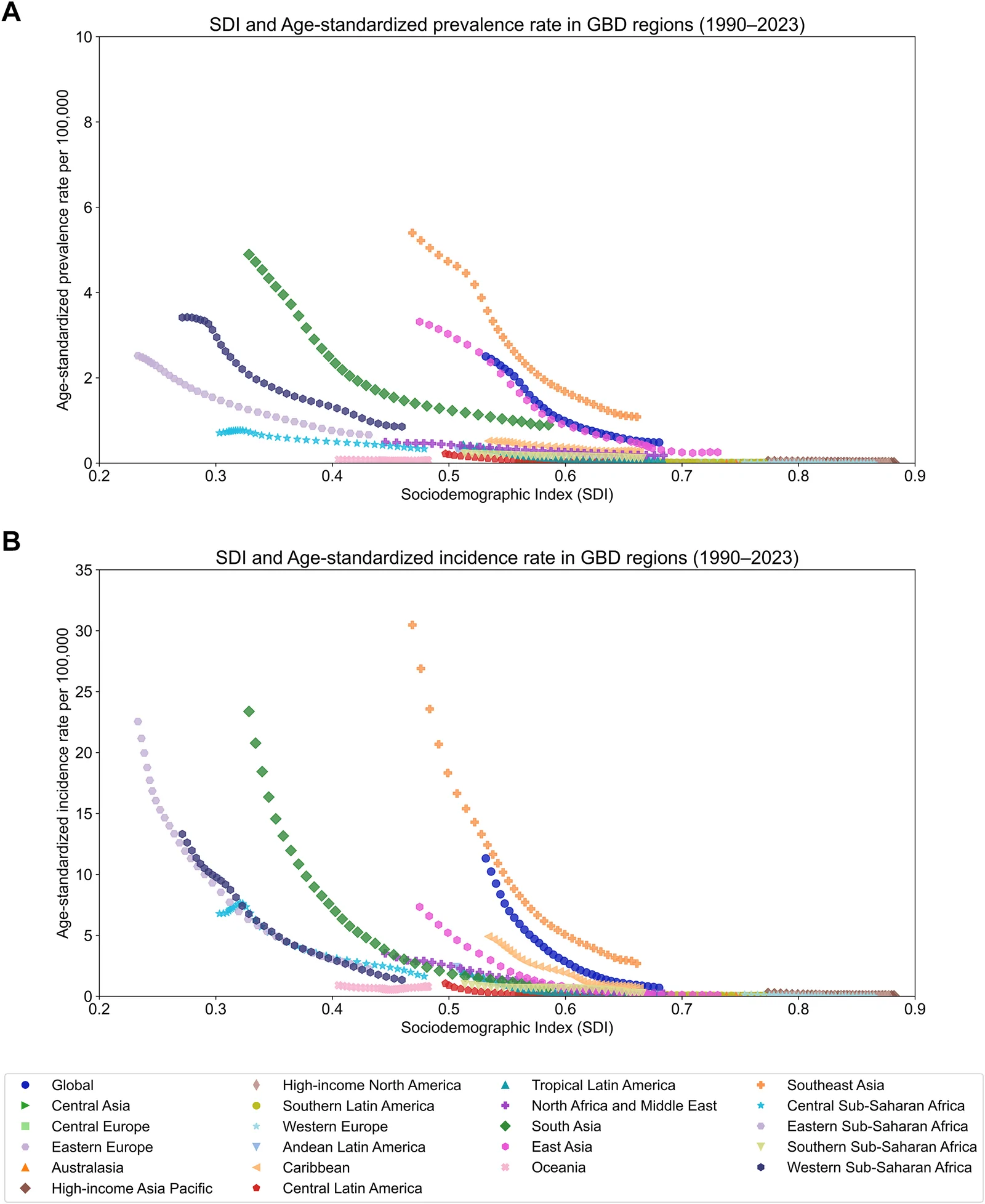

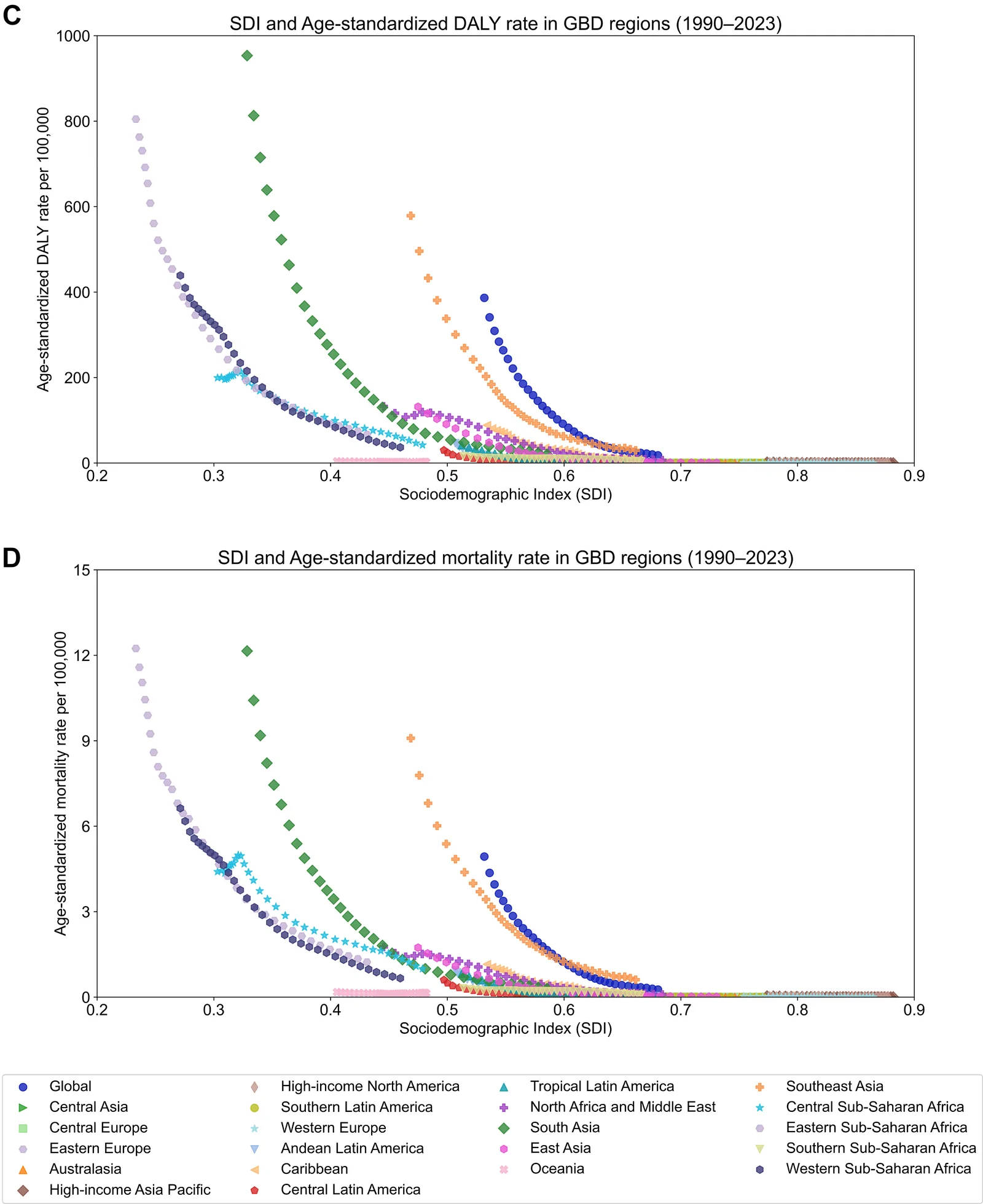
Trends in age-standardized (A) prevalence, (B) incidence rate, (C) DALY, and (D) mortality rates of tetanus across 21 GBD regions from 1990 to 2023. Each line represents a GBD region and illustrates the relationship between the Socio-demographic Index (SDI) and age-standardized tetanus burden over time. The SDI value for each region represents the population-weighted average SDI of all countries and territories within that region. DALY = disability-adjusted life year; GBD = Global Burden of Diseases, Injuries, and Risk Factors Study; SDI = Socio-demographic Index.

Fig. 5 presents age- and sex-specific patterns. Throughout the study period, age-specific prevalence, incidence, DALY, and mortality rates were highest among neonates younger than 28 days, followed by infants aged 1–5 months and 6–11 months, and were generally higher in males than in females across most age groups. Among neonates younger than 28 days, the age-specific incidence rate in males declined markedly from 4187.69 (95% UI, 2568.50–6853.34) per 100,000 population in 1990 to 123.50 (95% UI, 54.62–280.79) in 2023, while the corresponding rate in females decreased from 3156.17 (95% UI, 1968.39–5095.12) to 104.23 (95% UI, 45.79–202.41). Although absolute rates declined substantially over time, the concentration of burden in the neonatal period and early infancy remained pronounced. After infancy, prevalence rates remained low and generally decreased with age, whereas incidence and mortality rates tended to rise after age 50 years, particularly among older adults. Age-specific DALY and mortality rates also declined markedly across all age groups over time while generally remaining higher in males than in females (Tables S11 and S12).

**Fig. 5:**
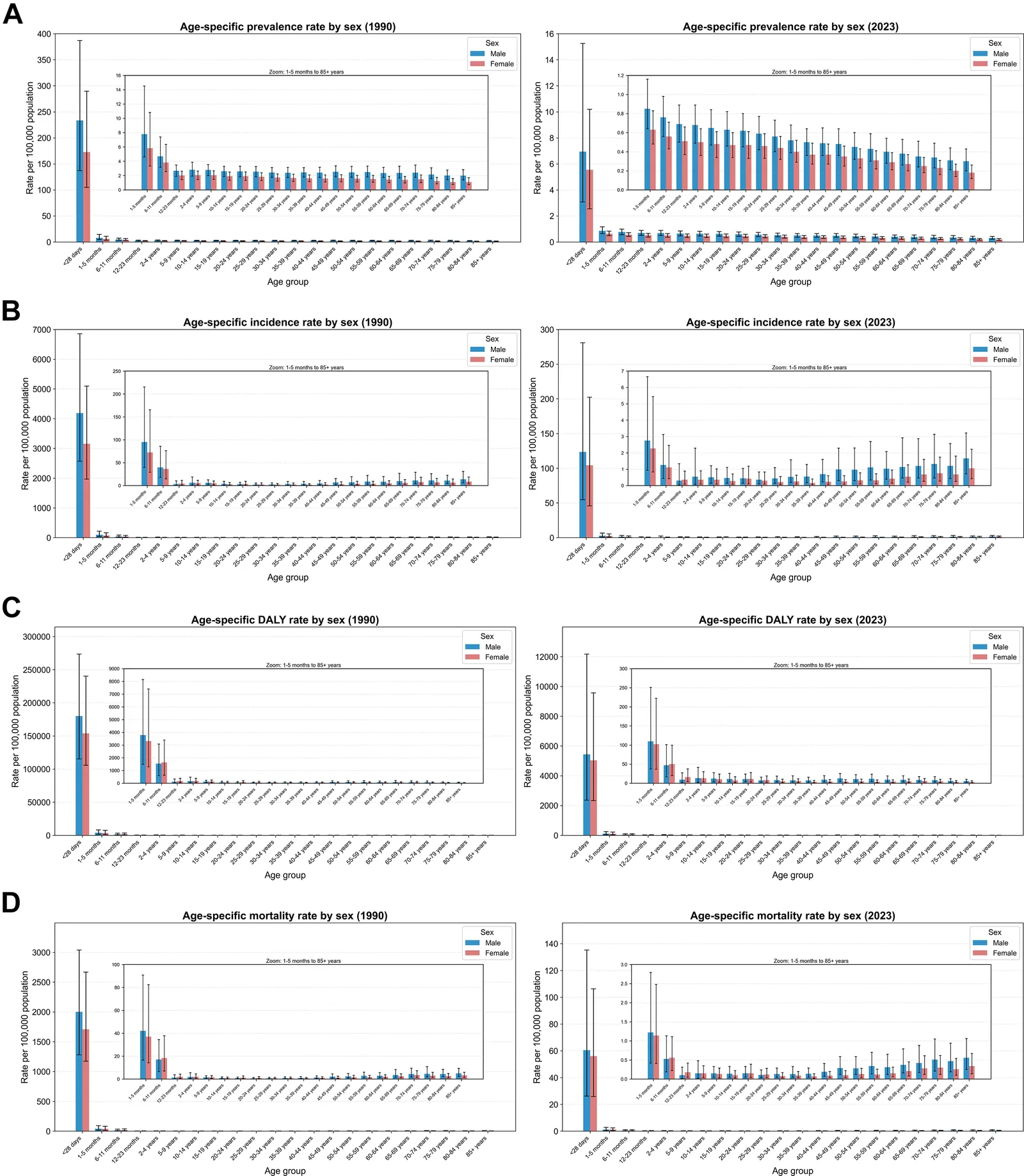
Age-specific (A) prevalence, (B) incidence rate, (C) DALY, and (D) mortality rate per 100,000 by sex. DALY = disability-adjusted life year.

## Discussion

This study provides an updated analysis of the burden of tetanus at global, regional, subregional, and national levels from 1990 to 2023. These findings are broadly consistent with those of a recent secondary analysis of GBD 2021, which similarly showed declining global burden of tetanus and concentrated burden among neonates and males.^14^ The present study, conducted within the official GBD 2023 Collaborator Network, extends this evidence through 2023 using updated data sources and methodologically refined estimates. There are several key findings. First, the global age-standardized prevalence, incidence, mortality, and DALY rates of tetanus declined substantially over the past three decades, indicating major progress in tetanus prevention worldwide. Despite this achievement, tetanus remains an important and preventable cause of morbidity and mortality, with a global age-standardized incidence rate of 0.70 per 100,000 population and DALY rate of 17.88 per 100,000 population in 2023. Second, marked geographic disparities persisted throughout the study period. South Asia and sub-Saharan Africa experienced the highest DALY burden, whereas high-income regions and Central Europe, Eastern Europe, and Central Asia remained among the lowest-burden settings. These findings highlight ongoing regional inequities despite overall global progress. Third, the relative ordering of regions changed little over time. Southeast Asia had the highest age-standardized prevalence and incidence rates during the study period, whereas Eastern sub-Saharan Africa had the highest age-standardized mortality rate throughout the study period. Australasia consistently had the lowest age-standardized DALY and mortality rates. Fourth, the burden of tetanus showed a strong and consistent inverse association with SDI: across all regions and epidemiological measures, higher SDI was associated with substantially lower tetanus prevalence, incidence, mortality, and DALY rates, illustrating persistent global inequalities in tetanus burden. Fifth, pronounced age- and sex-specific patterns persisted over time. Across the study period, the highest burden was observed among neonates younger than 28 days, with generally higher rates in males than in females across most age groups. Although absolute rates declined markedly between 1990 and 2023, the age gradient and sex differences remained evident, and mortality rates tended to increase gradually with advancing age in adulthood, particularly among males.

Our global trend analysis revealed a substantial decline in tetanus burden over the past three decades across all four epidemiological indicators: prevalence, incidence, DALYs, and mortality. This sustained reduction likely reflects long-term progress in global immunization programs, including EPI and IA2030, in alignment with the United Nations Sustainable Development Goals (SDGs).^1^^,^^26^ The observed 90% reduction in mortality rate and 95% reduction in DALY rate of tetanus between 1990 and 2023 indicate major progress toward SDG target 3.2,^27^ which aims to end preventable deaths among newborns and children younger than 5 years, as well as SDG target 3.8 on universal health coverage.^28^^,^^29^ These findings are consistent with previous GBD estimates of tetanus and underscore the resilience of global immunization programs despite disruptions related to the COVID-19 pandemic and regional conflict.^3^^,^^4^ Such disruptions to routine immunization and health services, together with rising vaccine hesitancy, might have slowed catch-up vaccination in some settings, although attribution to specific countries warrants caution. The marked decline in global incidence, which decreased by 93.8% from 1990 to 2023, is consistent with coordinated international efforts led by the WHO and the United Nations Children's Fund (UNICEF) through the EPI and MNTE.^30^ These programs have emphasized expanded vaccination coverage, including diphtheria and tetanus vaccination, maternal immunization, strengthened antenatal care, and the promotion of hygienic delivery practices since the late 1980s.^1^^,^^30^

Nevertheless, these estimates suggest that substantial regional heterogeneity in both the pace and magnitude of progress in reducing the burden of tetanus persists. This heterogeneity may reflect ongoing inequities in immunization coverage, health system capacity, and access to skilled birth attendance.^1^^,^^5^ Notably, previous estimates indicate that coverage of DTP3 declined between 2010 and 2019 in 94 countries and territories.^2^ Globally, only 11 countries were estimated to have achieved the WHO Global Vaccine Action Plan target of at least 90% coverage for all assessed vaccines by 2019.^2^ Although the global number of zero-dose children, defined as those who had not received the first dose of the diphtheria–tetanus–pertussis vaccine (DTP1), declined markedly from 1980 to 2019, progress has stagnated in recent years, with a disproportionate concentration of zero-dose children in conflict-affected and resource-constrained settings, particularly in sub-Saharan Africa.^3^ As vaccination coverage has stagnated or declined, several countries, territories, and regions have experienced new or resurgent vaccine-preventable disease threats, with implications for tetanus susceptibility.^3^^,^^10^

These geographic disparities were closely aligned with sociodemographic development. Our estimates suggested that incidence, mortality, and DALY rates of tetanus were inversely associated with SDI. SDI is a composite indicator including per capita income, educational attainment, and total fertility rate, which broadly reflects levels of sociodemographic development. Regions with lower SDI often have reduced access to and utilization of antenatal care, immunization services, and safe delivery practices, which may increase susceptibility to tetanus.^1^^,^^7^ In addition, lower-SDI settings frequently have higher fertility rates and lower vaccination coverage, increasing the number of susceptible neonates and potentially contributing to higher tetanus burden.^10^ Nevertheless, these SDI-related patterns should be interpreted with caution, because vulnerability to tetanus can also be shaped by factors beyond development level, including natural disasters, fragile infrastructure, and gaps in vaccination coverage or post-exposure prophylaxis.^31^ Regional averages may also mask substantial country-level heterogeneity, as countries within the same GBD region can differ markedly in SDI, vaccination coverage, health-system capacity, and exposure to acute disruption.

Regionally, East Asia and South Asia experienced the largest absolute reductions in tetanus incidence and mortality, consistent with expanded childhood and maternal immunization coverage and improvements in antenatal and perinatal care. These reductions are consistent with progress toward the WHO's MNTE targets in many countries in these regions.^10^^,^^32^ This broader pattern should be interpreted at the super-region level, as Southeast Asia, East Asia, and Oceania include heterogeneous subregions. Slower and more variable progress in Southeast Asia, East Asia, and Oceania may reflect challenges related to geographic isolation, inconsistent vaccine delivery, and limited access to timely critical care following infection, especially in low-income and middle-income countries.^5^ In sub-Saharan Africa, although substantial reductions were observed, progress remains uneven across subregions. Persistently higher burden of tetanus in this region likely reflects lower immunization coverage, limited access to skilled birth attendance, and systemic disruptions caused by poverty, conflict, population displacement, and fragile health infrastructure.^7^^,^^10^ These factors can interrupt vaccine supply chains, undermine disease surveillance, and delay treatment, thereby sustaining the preventable burden of tetanus.^27^^,^^33^

Pronounced age- and sex-specific patterns persisted across the study period. The highest tetanus burden was concentrated among neonates younger than 28 days, followed by infants aged 1–5 months and 6–11 months, underscoring the continued importance of MNTE-aligned strategies, including maternal immunization, hygienic delivery practices, clean cord care, and early postnatal health services.^30^ Although the neonatal and early infancy burden declined markedly by 2023, residual risk after infancy and in adulthood may reflect waning immunity in the absence of booster vaccination, cumulative exposure to injury, and a higher prevalence of comorbidities such as diabetes and immunosuppression.^34^^,^^35^ Consistently higher tetanus burden among males may be attributable to greater exposure to injury-related activities, differences in healthcare-seeking behavior, and lower uptake of routine booster vaccination in adulthood; rare cases have also been reported following voluntary medical male circumcision.^7^^,^^36^

From a policy perspective, our findings indicate that global efforts to reduce the burden of tetanus have yielded considerable gains in many locations, consistent with progress towards the MNTE and IA2030 targets.^1^^,^^2^^,^^5^ Western Europe, high-income North America, Australasia, and high-income Asia Pacific showed near-zero tetanus incidence and mortality in 2023, a pattern that is consistent with strong routine immunization systems, maternal care, and health system capacity. However, tetanus remains an important public health concern in South Asia and sub-Saharan Africa, underscoring the need for context-specific policy action. Country-level estimates are therefore essential for policy prioritization, because immunization delivery, maternal and neonatal care, and injury management are largely planned and implemented at the national level.

In some countries, a higher burden of tetanus may reflect persistent structural and financial barriers to effective prevention. Chronic conflict, political instability, population displacement, and fragile health systems continue to undermine routine immunization programs.^11^ For example, particularly in Africa, low DTP1 coverage (≤50%) and high relative dropout from DTP1 to DTP3 (≥30%) together contributed to low DTP3 coverage, leaving many children under-immunized or zero-dose, defined as not having received DTP1.^37^ Limited access to healthcare facilities and heavy reliance on out-of-pocket health expenditures further constrain vaccine uptake.^6^ In low SDI settings, where annual per capita health spending is among the lowest globally, households often face a trade-off between seeking preventive services and avoiding catastrophic health expenditures, which may constrain vaccine uptake and hinder progress toward universal health coverage.^6^ Limited immunization financing and inflationary pressure on national health budgets may further weaken vaccine procurement, delivery, and catch-up activities in low-SDI countries, highlighting the need for sustained domestic and international support.^38^

Reducing the persistent tetanus burden in high-risk locations requires proactive policy. Strengthening routine immunization systems and integrating tetanus prevention into broader maternal, neonatal, and child health services remain essential, through continued support for global initiatives such as the EPI and MNTE.^1^^,^^2^ Tetanus-containing vaccine coverage among women of reproductive age and pregnant women attending antenatal care should be prioritized, because maternal vaccination protects both mothers and newborns and reinforces opportunities for safe delivery and skilled birth attendance.^39^ In conflict-affected settings, strategies such as mobile vaccination teams, community-based engagement, and periodic catch-up campaigns are needed to reach zero-dose children.^6^ Beyond primary immunization, the introduction of tetanus toxoid-containing vaccine booster doses offers an important complementary strategy, addressing waning immunity and enabling catch-up vaccination among under-immunized adolescents and adults.^11^ The WHO recommends booster doses across the life course, and Gavi began offering programmatic support for their introduction in low-income countries in 2023, providing a critical financing mechanism to accelerate uptake in vulnerable populations.^1^^,^^2^ Reducing financial barriers through the expansion of universal health coverage, supported by international partners such as Gavi and UNICEF, will be critical to achieving equitable and durable tetanus control.^1^^,^^2^

This study has several limitations related to the GBD 2023 analytical framework. First, tetanus can be underdiagnosed and underreported in settings with incomplete vital registration, surveillance systems, and cause-of-death certification.^15^ Heterogeneity in reporting practices may contribute to underestimation in data-sparse locations, leading to greater statistical volatility where case counts are low. Accordingly, estimates should be interpreted in the context of uncertainty intervals generated throughout the modeling process, as observed differences may reflect variation in data density and model-related uncertainty rather than true epidemiological contrasts. Furthermore, apparent deviations from the expected association between SDI and tetanus burden in certain regions or countries, including Zimbabwe, Vanuatu, and Solomon Islands, particularly those with limited vital registration coverage and fragile surveillance systems, may reflect data sparsity and underreporting rather than true epidemiological differences. The wide uncertainty intervals observed in such regions are consistent with reduced data availability and limited confidence in the modeled estimates. In addition, country-level estimates may not fully capture subnational disparities in vaccination coverage, health-service access, and tetanus burden, particularly in rural areas. Second, cause-specific mortality estimates may be affected by misclassification of tetanus on death certificates and by the redistribution of deaths coded as resulting from non-specific causes within the GBD cause-of-death pipeline.^16^^,^^21^ Although these procedures improve comparability across locations and over time, they introduce additional uncertainty, particularly in areas with limited or heterogeneous mortality data. Third, key non-fatal quantities were not directly observed but were estimated through model-based approaches. Specifically, incidence was inferred by dividing modeled mortality by modeled CFRs, and prevalence was calculated using an assumed average duration of illness.^40^ While this approach enables internally consistent estimation across locations and time, residual uncertainty in mortality and CFR inputs may translate into uncertainty in incidence, prevalence, and YLD estimates. In addition, acute tetanus was modeled as a severe acute health state with fixed disability weights. However, clinical severity and outcomes can vary by setting and by access to timely care, which could influence YLD and DALY estimates. Comorbidities such as diabetes and immunosuppression, which can affect immune response or wound healing, were also not explicitly modeled as determinants of tetanus incidence or outcomes. Fourth, CFR inputs were obtained from published literature and required age-sex splitting when granular strata were unavailable. As a result, CFR estimates may be influenced by publication and selection bias in the available literature which can limit generalizability and introduce bias in subsequent estimates, even after adjustment for healthcare access and quality. Lastly, the interpretation of risk factor attribution for tetanus differs from that for many behavioral or metabolic risk factors in the GBD framework, because tetanus prevention is primarily shaped by immunization, safe delivery, clean cord care, and wound management.^1^^,^^3^^,^^15^ Although maternal immunization, in-facility delivery, and skilled birth attendance were incorporated as covariates in the neonatal tetanus model, the present descriptive analysis was not designed to estimate the independent contribution of each factor to regional differences in neonatal tetanus burden.^8^ Accordingly, these programmatic and health-system factors should be interpreted as important contextual determinants rather than as separately attributable risk factors in this study. While we have analyzed the association between tetanus incidence, prevalence, and DALY rates and SDI, we note that these comparisons are ecological and should be interpreted cautiously without implying causality.

Overall, this global analysis found substantial progress in reducing the burden of tetanus from 1990 to 2023, reflecting the long-term effectiveness of vaccination initiatives such as EPI and MNTE. Nevertheless, the remaining burden is largely preventable and is concentrated in populations and settings marked by persistent gaps in vaccination, maternal and neonatal care, safe delivery practices, wound management, and timely treatment. The strong inverse association between tetanus burden and the SDI underscores that persistent inequities are closely linked to structural barriers in prevention and care rather than unavoidable biological differences. Age- and sex-specific patterns, including the continued burden among neonates and males, further suggest that prevention strategies should span different stages of life, including maternal immunization and booster vaccination in adolescence and adulthood. Sustained progress toward tetanus elimination will require coordinated efforts that extend beyond immunization, including strengthening health systems, improving safe delivery and injury care, and advancing universal health coverage. Ultimately, eliminating this vaccine-preventable disease will depend on prioritizing high-burden and under-immunized populations and addressing the structural barriers that allow it to persist.

## Contributors

Please see Appendix 2 (pp10–14) for more detailed information about individual author contributions to the research, divided into the following categories: providing data or critical feedback on data sources; developing methods or computational machinery; providing critical feedback on methods or results; drafting the manuscript or revising it critically for important intellectual content; and managing the estimation or publications process. Members of the core research team for this topic area, Dong Keon Yon and Jonathan F Mosser, had full access to and verified the underlying data used to generate the estimates presented in this study. All other authors had access to and reviewed estimates as part of the research evaluation process, which includes additional stages of formal review. All authors read and approved the final version of the manuscript. Demelash Areda, Hyunjee Kim, and Jiseung Kang contributed equally as the first authors. Jonathan F Mosser and Dong Keon Yon jointly supervised this work (corresponding authors). The corresponding authors had final responsibility for the decision to submit for publication.

## Data sharing statement

The findings from this study were produced using data available in public online repositories or in the published literature, data that are publicly available on request from the data provider, and data that are not publicly available due to restrictions by the data provider and which were used under license for the current study. Details on data sources can be found on the GHDx website, including information about the data provider and links to where the data can be accessed or requested (where available). To download the data used in these analyses, please visit the Global Health Data Exchange GBD 2023 website at https://ghdx.healthdata.org/gbd-2023.

## Editor note

The Lancet Group takes a neutral position with respect to territorial claims in published maps and institutional affiliations.

## Declaration of interests

Q E S Adnani reports grants or contracts from the Faculty of Medicine assignment grant for 2026 (RKDU) at Universitas Padjadjaran, Indonesia (contract number 6235/UN6.C/PT.02/2026), outside the submitted work. S Afzal reports institutional support from the Institute of Public Health Lahore for study materials, medical writing, and library resources; grants or contracts from the Dean Office, Institute of Public Health Lahore; payment or honoraria for lectures, presentations, speakers bureaus, manuscript writing, or educational events provided by the Dean, Institute of Public Health Lahore; support for attending meetings or travel provided by the Dean, Institute of Public Health Lahore, Pakistan; participation on a data safety monitoring board or advisory board as a Member of the Pakistan National Bioethics Committee, a Member of the Institutional Review Board of Fatima Jinnah Medical University, a Member of the Ethical Review Board and Data Monitoring Board at the Institute of Public Health Lahore, Pakistan, In Charge of the Clinical Research Organization at King Edward Medical University, and a Member of the Annals of King Edward Medical University Advisory Board; leadership or fiduciary roles in other boards, societies, committees, or advocacy groups, paid or unpaid, as a Member of the Pakistan Higher Education Commission Research Committee, a Member of the Pakistan Medical and Dental Commission Research and Journals Committee, a Member of the Pakistan National Bioethics Committee, a Member of the Pakistan Society of Internal Medicine, a Member of the Pakistan Association of Medical Editors, a Member of the Medical Microbiology and Infectious Diseases Society, a Fellow of Leads International, a Fellow of the Faculty of Public Health UK, and a Fellow of the College of Physicians and Surgeons Pakistan; receipt of equipment, materials, drugs, medical writing, gifts, or other services in the form of computer software and equipment from Bergen University, Norway, for research writing; and other financial or non-financial interests from the Dean Public Health, Institute of Public Health Birdwood Lahore, as a Member of the National Bioethics Committee Pakistan and a Member of the Prime Minister Taskforce on HCV, HIV, and Infectious Diseases; all outside the submitted work. M S Aslam reports grants or contracts from the Xiamen University Malaysia Research Fund (XMUMRF; grant number XMUMRF/2025-C15/ITCM/0006) for the project “Therapeutic and Toxicity Evaluation of Selected Medicinal Herbs for NAFLD: Exploring the Inter-Organelle Contact Sites Modulation Theory” as Co-Investigator from Jan, 2025, to Dec, 2027 (ongoing); from the Xiamen University Malaysia Research Fund (XMUMRF; grant number XMUMRF/2023-C11/ISEM/0041) for the project “Children's Rights Education in the Early Years of Divorce: An Exploration of Adolescents' Perspectives” as Co-Investigator from January, 2023, to December, 2025 (ongoing); and from the Xiamen University Malaysia Research Fund (XMUMRF; grant number XMUMRF/2026-C17/ITCM/0007) for the project “Olfactory Stimulation and Autonomic Regulation in Menstrual Disorders” as Co-Investigator from January, 2026, to December, 2028 (ongoing); all were internal XMUMRF research grants administered by Xiamen University Malaysia, with funds disbursed only to an institutional research account and no salary, honoraria, or personal payments made to the author; all outside the submitted work. S Bhaskar reports grants or contracts from the Japan Society for the Promotion of Science (JSPS) and the Japanese Ministry of Education, Culture, Sports, Science and Technology (MEXT) Grant-in-Aid for Scientific Research (KAKENHI; grant ID 23KF0126), and from the JSPS and Australian Academy of Science JSPS International Fellowship (grant ID P23712); payment or honoraria for lectures, presentations, speakers bureaus, manuscript writing, or educational events from the Japan Stroke Society, Japan, for an invited keynote presentation at the 50th Annual Meeting of the Japan Stroke Society in Osaka, and from Semey Medical University, Kazakhstan, for an invited professorship and teaching course in neurocardiovascular therapy; support for attending meetings or travel from the Japan Stroke Society, Japan, for the invited keynote presentation at the 50th Annual Meeting of the Japan Stroke Society in Osaka, and from Asialink, The University of Melbourne, for the opening invited keynote at the 31st Asialink Leaders Foundation Week Program 2026 in Melbourne; and leadership or fiduciary roles in other boards, societies, committees, or advocacy groups, paid or unpaid, as District Chair for Diversity, Equity, Inclusion, and Belonging with Rotary District 9675, Sydney, Australia; Chair, Founding Member, and Manager of the Global Health & Migration Hub Community, Global Health Hub Germany, Berlin, Germany; Editorial Board Member of PLOS One, BMC Neurology, Frontiers in Neurology, Frontiers in Stroke, Frontiers in Public Health, Journal of Aging Research, Neurology International, Diagnostics, and BMC Medical Research Methodology; Member of the College of Reviewers, Canadian Institutes of Health Research, Government of Canada; Director of Research at the World Headache Society, Bengaluru, India; Expert Adviser and Reviewer for the Cariplo Foundation, Milan, Italy; Visiting Director at the National Cerebral and Cardiovascular Center, Department of Neurology, Division of Cerebrovascular Medicine and Neurology, Suita, Osaka, Japan; Member of the Scientific Review Committee, Cardiff University Biobank, Cardiff, UK; Rotary Reconciliation Action Plan Chair; and Healthcare and Medical Adviser for Japan Connect, Osaka, Japan; all outside the submitted work. I Ilic reports grants from the Ministry of Science, Technological Development and Innovation of the Republic of Serbia (number 451-03-137/2025-03/200110), outside the submitted work. K Krishan reports other financial or non-financial interests in the form of non-financial support from the UGC Centre of Advanced Study, CAS II, awarded to the Department of Anthropology, and the RUSA 2.O grant awarded by the Ministry of Education to Panjab University, Chandigarh, India, outside the submitted work. L Monasta reports support from the Italian Ministry of Health (Ricerca Corrente 34/2017), with payments made to the Institute for Maternal and Child Health IRCCS Burlo Garofolo, Trieste, Italy, outside the submitted work. J F Mosser reports support for the present manuscript from the Gates Foundation (grant funding); and grants or contracts from Gavi; payment or honoraria for lectures, presentations, speakers bureaus, manuscript writing, or educational events from Providence Health & Services for a continuing medical education presentation; and support for attending meetings or travel from the Gates Foundation; all outside the submitted work. L Ronfani reports support from the Italian Ministry of Health through the contribution given to the Institute for Maternal and Child Health IRCCS Burlo Garofolo, Trieste, Italy (Ricerca Corrente 34/2017), outside the submitted work. S Saleem reports payment or honoraria for lectures, presentations, speakers bureaus, manuscript writing, or educational events from the Health Services Academy, Islamabad, Pakistan, outside the submitted work. J A Singh reports consulting fees from ROMTech, Atheneum, Clearview Healthcare Partners, Yale, Hulio, Horizon Pharmaceuticals/DINORA, ANI/Exeltis USA Inc., SOBI Pharmaceuticals, Frictionless Solutions, Schipher, Crealta/Horizon, Medisys, Fidia, PK Med, Two Labs Inc., Adept Field Solutions, Clinical Care Options, Putnam Associates, Focus Forward, Navigant Consulting, Spherix, MedIQ, Jupiter Life Science, UBM LLC, Trio Health, Medscape, WebMD, Practice Point Communications, the US National Institutes of Health, and the American College of Rheumatology, with consultant fees paid to J A Singh by each entity; payment or honoraria for lectures, presentations, speakers bureaus, manuscript writing, or educational events from Simply Speaking, for which J A Singh serves on the speakers bureau, and reports that he previously served as a consultant and received consultant fees; support for attending meetings or travel from OMERACT, as a past steering committee member, to attend its meeting every 2 years; participation on a Data Safety Monitoring Board or Advisory Board with the US Food and Drug Administration Arthritis Advisory Committee, as a previous member without financial support; a leadership or fiduciary role in other boards, societies, committees, or advocacy groups, paid or unpaid, as a past steering committee member of OMERACT, an international organization that develops measures for clinical trials and receives arm's-length funding from 12 pharmaceutical companies, for which J A Singh previously received support to attend its meeting every 2 years; stock or stock options in Atyr Pharmaceuticals, Atai Life Sciences, Kintara Therapeutics, Intelligent Biosolutions, Acumen Pharmaceutical, TPT Global Tech, Vaxart Pharmaceuticals, Atyu Biopharma, Adaptimmune Therapeutics, GeoVax Labs, Pieris Pharmaceuticals, Enzolytics Inc., Seres Therapeutics, Tonix Pharmaceuticals Holding Corp., Aebona Pharmaceuticals, and Charlotte's Web Holdings, Inc.; and previous ownership of stock options in Amarin, Viking, and Moderna Pharmaceuticals; all outside the submitted work. G Zamagni reports support from the Italian Ministry of Health through the contribution given to the Institute for Maternal and Child Health IRCCS Burlo Garofolo, Trieste, Italy, outside the submitted work.
