# Supplementary material for: Global, regional, and national burden of tetanus in 204 countries and territories, 1990–2023: a systematic analysis for the Global Burden of Disease Study 2023
Source: eClinicalMedicine. 2026 Sep 28;100:104202. doi: 10.1016/j.eclinm.2026.104202 (PMC13639606; doi:10.1016/j.eclinm.2026.104202)
Supplement: Supplementary file 1 [file mmc1.docx]

**Appendix 1: Supplementary methods and results to “Global, regional, and national burden of tetanus in 204 countries and territories, 1990-2023: a systematic analysis for the Global Burden of Disease Study 2023”**

This appendix provides further methodological details and results for “Global, regional, and national burden of tetanus in 204 countries and territories, 1990-2023: a systematic analysis for the Global Burden of Disease Study 2023”.

**Preamble**

This appendix provides further methodological details and results for “Global, regional, and national burden of tetanus in 204 countries and territories, 1990-2023: a systematic analysis for the Global Burden of Disease Study 2023”. Portions of this appendix have been reproduced or adapted from appendices for GBD 2023 Diseases, Injuries and Risk Factors Collaborators,^1^ GBD 2023 Causes of Death Collaborators,^2^ GBD 2023 Demographic collaborators,^3^ and GBD 2023 Cancer Collaborators.^4^ References are provided for reproduced or adapted sections.

**Table of Contents**

**1.Supplementary Methods …………………………………………………………………………………3**

1.1 Overview

1.2. Classification of 7 super regions and 21 GBD regions

1.3 Detail definition and modelling of cause of death

1.4 Detail definition and modelling of non-fatal

1.5 Country-level SDI benchmarking analysis

**2. References………………………………………………………………………………………………14**

**3.** **Supplementary Figures ………………………………………………………………………….……16**

**Figure S1.** Age-standardized YLL and YLD rate (95% UI) per 100,000 population from 1990 to 2023.

**Figure S2.** Association of SDI and age-standardized (A) prevalence, (B) incidence, (C) DALY, and (D) mortality rate per 100,000 population in 2023.

**Figure S3.** Trends in age-standardized (A) prevalence, (B) incidence rate, (C) DALY, and (D) mortality rates of tetanus across 204 countries from 1990 to 2023.

**4. Supplementary Tables …………………………………………………………………………………23**

**Table S1.** Prevalence, incidence, DALY, and mortality counts (95% UI) in 1990 and 2023.

**Table S2.** Age-standardized prevalence and DALY rate (95% UI) per 100,000 population in 1990 and 2023.

**Table S3.** Age-standardized incidence and mortality rate (95% UI) per 100,000 population in 1990 and 2023.

**Table S4.** Age-standardized prevalence rate (95% UI) per 100,000 population in GBD super region from 1990 to 2023.

**Table S5.** Age-standardized incidence rate (95% UI) per 100,000 population in GBD super region from 1990 to 2023.

**Table S6.** Age-standardized DALY rate (95% UI) per 100,000 population in GBD super region from 1990 to 2023.

**Table S7.** Age-standardized mortality rate (95% UI) per 100,000 population in GBD super region from 1990 to 2023.

**Table S8.** Age-standardized YLD rate (95% UI) per 100,000 population from 1990 to 2023.

**Table S9.** Age-standardized YLL rate (95% UI) per 100,000 population from 1990 to 2023.

**Table S10.** SDI and age-standardized prevalence, incidence, DALY, and mortality rate (95% UI) per 100,000 population from 1990 to 2023.

**Table S11.** Age-specific prevalence and incidence rate (95% UI) per 100,000 population by sex in 1990 and 2023.

**Table S12.** Age-specific DALY and mortality rate (95% UI) per 100,000 population by sex in 1990 and 2023.

**1. Supplementary Methods**

**1.1 Overview**

The Global Burden of Diseases, Injuries, and Risk Factors Study (GBD) 2023 comprehensively assessed the burden of tetanus across 204 countries and territories from 1990 to 2023, stratified by age group and sex. The burden of tetanus was quantified using standard GBD metrics, including prevalence, incidence, mortality, years of life lost (YLLs), years lived with disability (YLDs), and disability-adjusted life years (DALYs). GBD 2023 follows the Guidelines for Accurate and Transparent Health Estimates Reporting (GATHER) to ensure transparency and comparability of health estimates.^5^

Advanced modelling approaches were applied to generate estimates by location, age group, sex, and year. Uncertainty was quantified using 250 posterior simulation draws, with 95% uncertainty intervals (UIs) defined as the 2.5th and 97.5th percentiles of the posterior distributions. Age-standardized rates were calculated using the GBD 2023 global standard population, allowing for meaningful comparisons of tetanus burden across regions and over time.^1-3^

**1.2. Classification of 7 super regions and 21 GBD regions**

| **Super Region** | **GBD Region** |
| --- | --- |
| High-income | High-income North America |
|  | Western Europe |
|  | Australasia |
|  | High-income Asia Pacific |
| Central Europe, Eastern Europe, and Central Asia | Central Europe |
|  | Eastern Europe |
|  | Central Asia |
| North Africa and Middle East | North Africa and Middle East |
| South Asia | South Asia |
| Southeast Asia, East Asia, and Oceania | Southeast Asia |
|  | East Asia |
|  | Oceania |
| Sub-Saharan Africa | Western Sub-Saharan Africa |
|  | Eastern Sub-Saharan Africa |
|  | Central Sub-Saharan Africa |
|  | Southern Sub-Saharan Africa |
| Latin America and Caribbean | Andean Latin America |
|  | Central Latin America |
|  | Southern Latin America |
|  | Tropical Latin America |
|  | Caribbean |

**1.3 Detail definition and modelling of cause of death**

***Input data***

Cause-of-death data of tetanus were obtained from multiple sources, including vital registration systems, verbal autopsy studies, and disease surveillance data, depending on availability by location. Cause-of-death data that were highly incongruent with other available evidence from the same location or from locations with similar sociodemographic characteristics were excluded prior to analysis.^2^ For cause-of-death estimation, tetanus mortality was mapped using International Classification of Diseases (ICD)-10 codes A33–A35.0 and ICD-9 codes 037–037.9 and 771.3 within the GBD cause-of-death framework.^2^

***Modelling strategy***

A Cause of Death Ensemble modelling (CODEm) approach was used to generate age-, sex-, location-, and year-specific tetanus mortality estimates.^6^ In GBD 2023, the final ensemble incorporated both count-based and rate-based model specifications. Separate sex-specific models were fitted for neonatal tetanus (under 1 year of age) and for tetanus among children and adults aged 1 year and older, reflecting differences in epidemiology, data availability, and risk structures across age groups. To address heterogeneity in data completeness, models were further stratified by the quality of vital registration systems, with both data-rich and global models developed for each age–sex group and subsequently combined to produce a single, harmonized set of mortality estimates across all locations.

Candidate models included covariates capturing immunization coverage, health system performance, sociodemographic development, and maternal and perinatal care, selected based on biological plausibility and empirical performance. To account for disruptions in routine immunization services during the COVID-19 pandemic, the COVID-inclusive average lagged coverage of the third dose of the diphtheria–tetanus–pertussis vaccine (DTP3) over the preceding five years was included as a key covariate in both neonatal and non-neonatal models. A five-year lag was used to reflect the fact that tetanus susceptibility is influenced by recent cohort-level immunization history rather than only by vaccination coverage in the year of death, and to reduce the influence of short-term annual fluctuations in reported coverage. This lagged covariate therefore captures recent immunization history and pandemic-related disruptions in population protection, rather than vaccination coverage in the year of death alone. Additional covariates in the neonatal tetanus models included tetanus toxoid coverage, the proportion of in-facility deliveries, skilled birth attendance, the Healthcare Access and Quality (HAQ) Index, lag-distributed income per capita, the Socio-demographic Index (SDI), and mean years of education. For tetanus among individuals aged 1 year and older, covariates included sanitation access, the HAQ Index, lag-distributed income per capita, SDI, and mean years of education. The direction of association for each covariate was constrained based on prior epidemiological knowledge. Detailed lists of covariates used in the neonatal and over-1-year models are provided in **Tables 1a** and **1b**, respectively.

**Table 1a**. Summary of covariates used in the tetanus cause-of-death model for ages under 1 year.

| **Level** | **Covariates** | **Direction** |
| --- | --- | --- |
| 1 | Average COVID-inclusive diphtheria-tetanus-pertussis third dose (DTP3) vaccination coverage over the past five years | - |
|  | Tetanus toxoid coverage | - |
| 2 | In-facility deliveries (proportion) | - |
|  | Skilled birth attendance (proportion) | - |
|  | Healthcare Access and Quality (HAQ) Index | - |
| 3 | Lag-distributed income (LDI) | - |
|  | Socio-demographic Index (SDI) | - |
|  | Mean years of education per capita | - |

**Table 1b**. Summary of covariates used in the tetanus cause-of-death model for ages 1 year and older.

| **Level** | **Covariates** | **Direction** |
| --- | --- | --- |
| 1 | Average COVID-inclusive diphtheria-tetanus-pertussis third dose (DTP3) vaccination coverage over the past five years | - |
| 2 | Healthcare Access and Quality (HAQ) Index | - |
| 3 | Sanitation access (proportion) | - |
|  | Lag-distributed income (LDI) | - |
|  | Socio-demographic Index (SDI) | - |
|  | Mean years of education per capita | - |

***Cause-of-death modelling framework***

Cause-specific mortality estimates for tetanus were generated using the GBD cause-of-death modelling framework, which integrates heterogeneous mortality data sources through a standardized, multi-step analytical pipeline. This framework was designed to produce internally consistent estimates of cause-specific mortality by age, sex, location, and year from 1980 to 2023.^3^

Mortality data inputs for tetanus included vital registration systems, verbal autopsy studies, and disease surveillance data, depending on data availability and quality by location. All input data underwent systematic preprocessing, including standardization of age and sex groups, mapping to the GBD cause list based on ICD codes, and redistribution of non-specific or implausible cause assignments (“garbage codes”) to valid underlying causes using established redistribution algorithms. Data sources deemed highly inconsistent with other evidence from the same location or from locations with similar sociodemographic characteristics were excluded prior to modelling.

Following data preparation, tetanus mortality was estimated using the CODEm framework. CODEm evaluates a large set of plausible statistical models that relate observed cause-specific mortality to covariates capturing demographic, socioeconomic, and health system characteristics. Candidate models include both count-based and rate-based specifications, with varying combinations of covariates, functional forms, and temporal smoothing strategies. Model performance is assessed using out-of-sample predictive validity, and the final ensemble estimate is constructed as a weighted combination of the best-performing models.

Separate sex-specific CODEm models were fitted for neonatal tetanus (under 1 year of age) and for tetanus among children and adults aged 1 year and older, reflecting distinct epidemiological patterns and data structures across age groups. To account for variation in data completeness, models were additionally stratified by the quality of vital registration systems, with both data-rich and global models developed for each age–sex group. Outputs from these models were subsequently combined to produce a single, harmonized set of tetanus mortality estimates for all locations.

To capture disruptions in routine immunization services during the COVID-19 pandemic, the COVID-inclusive average lagged coverage of the third dose of the diphtheria–tetanus–pertussis vaccine over the preceding five years was incorporated as a covariate in tetanus mortality models. This approach allowed the models to reflect recent declines in vaccination coverage and their potential impact on tetanus mortality.

Final estimates of tetanus mortality were adjusted to ensure consistency with all-cause mortality envelopes using the GBD cause-of-death correction process, such that the sum of cause-specific deaths equaled independently estimated all-cause mortality for each age, sex, location, and year. These corrected mortality estimates formed the basis for subsequent estimation of incidence, prevalence, YLLs, and DALYs for tetanus in GBD 2023.

**1.4 Detail definition and modelling of non-fatal**

***Input data***

In the GBD 2023 non-fatal estimation framework, the tetanus case-definition documentation included ICD-10 codes A33–A35.0 and Z23.5 and ICD-9 codes 037–037.9, 771.3, and V03.7.^1,2^ Non-fatal burden was derived from cause-specific mortality estimates and modelled case fatality rates (CFRs), and acute prevalence was subsequently estimated from incidence using disease-duration assumptions.^1,7^ For neonatal tetanus, long-term impairment outcomes among survivors were modelled separately and contributed to non-fatal burden estimates across subsequent ages.

The non-fatal model of tetanus was constructed using two primary data inputs. First, data were obtained from systematic reviews of the tetanus CFR literature. Second, tetanus mortality estimates generated using the CODEm framework were incorporated to support the estimation of non-fatal outcomes. The literature search was conducted in PubMed using predefined search strings combining terms related to tetanus, mortality, case fatality, and burden of disease.

Prior to modelling, CFR data that were not reported separately by sex or that were presented in age intervals wider than ten years were split into sex- and age-specific groups. Because sex- and age-specific CFR data were sparse, location- and year-specific sex and age patterns could not be reliably estimated. Instead, global sex ratios and age patterns were derived using all available sex- and age-specific CFR data and subsequently applied to disaggregate aggregated observations prior to inclusion in the model. Uncertainty was propagated throughout this splitting process.

Sex-specific adjustment factors were estimated using Meta-Regression Bayesian, Regularized, Trimmed (MR-BRT), a Bayesian meta-regression framework. The resulting female-to-male sex adjustment factor applied in GBD 2023 tetanus modelling was 1.20 (95% uncertainty interval, 1.16–1.24), compared with 1.15 (1.01–1.29). To generate a global age pattern, tetanus CFR data reported in age intervals of ten years or less were used to fit a Disease Modeling Meta-Regression (DisMod-MR) model. The HAQ Index was included as a location-level covariate, reflecting variation in health system performance. The final global age pattern, estimated for each sex and age group from early neonatal to 95 years and older, was then applied to split the remaining aggregated CFR data.

***Modelling strategy***

CFR estimates were generated using DisMod-MR to produce location-, year-, age-, and sex-specific estimates from sex- and age-specific input data following the splitting procedures.^3^ The HAQ Index was included as a location-level covariate in the model, with a directional prior imposed such that locations with higher HAQ Index values were predicted to have lower tetanus CFRs. Covariate effects were estimated on the log scale and exponentiated to facilitate interpretation as odds ratios.

Tetanus incidence rates were derived by dividing tetanus mortality rate estimates by the predicted CFRs, as follows:

$$Incidence=Mortality\div CFR$$

Consistent with previous GBD cycles, tetanus mortality rates were estimated using separate CODEm models for children younger than 1 year and for children and adults aged 1 year and older, stratified by data-rich and non–data-rich locations and by sex. Prevalence of acute tetanus was estimated from incidence by applying the average case duration of 20 days specified in the GBD 2023 non-fatal estimation framework.^1^ The case-duration input was informed by prior clinical and review literature identified in the GBD 2023 Sources Tool.^8-13^ This duration parameter represents the average length of the acute disease state used for prevalence estimation.

$$Acute prevalence=Incidence \times Average duration of acute disease$$

This calculation converts annual incidence into the expected average point prevalence of the acute tetanus health state during a given year. Conceptually, this corresponds to the expected number of individuals with acute tetanus on a randomly selected day in that year. These calculations were performed across posterior draws, and final estimates were summarized as the mean with corresponding 95% UIs. The acute prevalence component was used to estimate YLDs for severe acute tetanus. Separately, for neonatal tetanus, impairment distributions were aligned with those used for neonatal encephalopathy. To estimate the burden of long-term impairment among neonatal tetanus survivors, the incidence of survival following neonatal tetanus was first calculated.

$$Incidence of survival=Incidence\times(1-CFR)$$

Survivors were then proportionally allocated to mild and moderate-to-severe impairment categories using estimates from the literature. These impairment-specific incidence estimates were subsequently used as input data for separate DisMod-MR models, which produced prevalence estimates of mild and moderate-to-severe impairment attributable to neonatal tetanus across all ages, sexes, years, and locations. As in previous GBD studies, excess mortality associated with neonatal encephalopathy was incorporated as a prior in the moderate-to-severe impairment model.

***Non-fatal modelling framework***

The non-fatal estimation of tetanus was implemented within the standard GBD non-fatal analytical pipeline, linking mortality-informed incidence estimation to prevalence and disability outcomes across age, sex, location, and year. Location-, year-, age-, and sex-specific incidence was derived from mortality rates and modelled CFRs, and prevalence was subsequently estimated using an assumed average episode duration. YLDs were calculated by applying disability weights to prevalent cases under the assumption that acute tetanus represents a severe state of health. For neonatal tetanus, additional long-term impairment outcomes among survivors were explicitly modelled by allocating survivors into mild and moderate-to-severe impairment states using evidence from the literature and estimating impairment-specific prevalence through separate DisMod-MR models. Consistent with the GBD framework, YLD estimates were adjusted for comorbidity to account for the non-independence of co-occurring health states before aggregation to summary non-fatal burden metrics.

**1.5 Country-level SDI benchmarking analysis**

We conducted an exploratory country-level benchmarking analysis across 204 countries and territories to identify low-SDI countries with lower-than-expected tetanus burden in 2023.^1^ For each burden metric, age-standardized rates per 100,000 population for both sexes from 1990 to 2023 were linked with corresponding annual country-level SDI values. For the 2023 analysis, the expected burden for each country was estimated from the locally weighted scatterplot smoothing (LOWESS)-fitted relationship between SDI and each age-standardized burden metric across all countries. For country *i* and burden metric *m*, the observed-to-expected ratio was calculated as follows:

$${Observed-to-expected ratio}_{i,m}=\frac{{observed age-standardized burden}_{i,m}}{{Expected age-standardized burden}_{i,m}}$$

Countries with SDI <0.50 in 2023 were considered low-SDI settings. Among these countries, the five countries with the lowest observed-to-expected ratios were highlighted for each burden metric.^14-16^ This analysis was performed to support exploratory interpretation of country-level deviations from the overall SDI–burden relationship.

**REFERENCES**

1.	Hay SI, Ong KL, Santomauro DF, et al. Burden of 375 diseases and injuries, risk-attributable burden of 88 risk factors, and healthy life expectancy in 204 countries and territories, including 660 subnational locations, 1990-2023: a systematic analysis for the Global Burden of Disease Study 2023. *The Lancet* 2025; **406**(10513): 1873–922.

2.	Naghavi M, Kyu HH, A B, et al. Global burden of 292 causes of death in 204 countries and territories and 660 subnational locations, 1990-2023: a systematic analysis for the Global Burden of Disease Study 2023. *The Lancet* 2025; **406**(10513): 1811–72.

3.	Schumacher AE, Zheng P, Barber RM, et al. Global age-sex-specific all-cause mortality and life expectancy estimates for 204 countries and territories and 660 subnational locations, 1950-2023: a demographic analysis for the Global Burden of Disease Study 2023. *The Lancet* 2025; **406**(10513): 1731–810.

4.	Force LM, Kocarnik JM, May ML, et al. The global, regional, and national burden of cancer, 1990-2023, with forecasts to 2050: a systematic analysis for the Global Burden of Disease Study 2023. *The Lancet* 2025; **406**(10512): 1565–86.

5.	Stevens GA, Alkema L, Black RE, et al. Guidelines for Accurate and Transparent Health Estimates Reporting: the GATHER statement. *Lancet* 2016; **388**(10062): e19–e23.

6.	Foreman KJ, Lozano R, Lopez AD, Murray CJ. Modeling causes of death: an integrated approach using CODEm. *Popul Health Metr* 2012; **10**: 1.

7.	Li J, Liu Z, Yu C, et al. Global epidemiology and burden of tetanus from 1990 to 2019: A systematic analysis for the Global Burden of Disease Study 2019. *Int J Infect Dis* 2023; **132**: 118–26.

8.	Fair E, Murphy TV, Golaz A, Wharton M. Philosophic objection to vaccination as a risk for tetanus among children younger than 15 years. *Pediatrics* 2002; **109**(1): E2.

9.	Atkinson WL. Epidemiology and Prevention of Vaccine-Preventable Diseases. 6th ed. Atlanta, GA: Centers for Disease Control and Prevention; 2000.

10.	Ernst ME, Klepser ME, Fouts M, Marangos MN. Tetanus: pathophysiology and management. *Ann Pharmacother* 1997; **31**(12): 1507–13.

11.	Lau LG, Kong KO, Chew PH. A ten-year retrospective study of tetanus at a general hospital in Malaysia. *Singapore Med J* 2001; **42**(8): 346–50.

12.	Cook TM, Protheroe RT, Handel JM. Tetanus: a review of the literature. *Br J Anaesth* 2001; **87**(3): 477–87.

13.	Edlich RF, Hill LG, Mahler CA, et al. Management and prevention of tetanus. *J Long Term Eff Med Implants* 2003; **13**(3): 139–54.

14.	Comfort H, McHugh TA, Schumacher AE, et al. Global, regional, and national stillbirths at 20 weeks' gestation or longer in 204 countries and territories, 1990–2021: findings from the Global Burden of Disease Study 2021. *The Lancet* 2024; **404**(10466): 1955–88.

15.	Kyu HH, Abate D, Abate KH, et al. Global, regional, and national disability-adjusted life-years (DALYs) for 359 diseases and injuries and healthy life expectancy (HALE) for 195 countries and territories, 1990–2017: a systematic analysis for the Global Burden of Disease Study 2017. *The Lancet* 2018; **392**(10159): 1859–922.

16.	Gardner WM, Razo C, McHugh TA, et al. Prevalence, years lived with disability, and trends in anaemia burden by severity and cause, 1990–2021: findings from the Global Burden of Disease Study 2021. *The Lancet Haematology* 2023; **10**(9): e713–e34.

**Supplementary Figures**

**Figure S1.** Age-standardized YLL and YLD rate (95% UI) per 100,000 population from 1990 to 2023.

UI= uncertainty interval; YLD= years lived with disability; YLL= years of life lost.

**Figure S2**. Association of SDI and age-standardized (A) prevalence, (B) incidence, (C) DALY, and (D) mortality rate per 100,000 population in 2023.

DALY= disability-adjusted life year; GBD= Global Burden of Diseases, Injuries, and Risk Factors Study; SDI= Socio-demographic Index.

**Figure S3**. Trends in age-standardized (A) prevalence, (B) incidence rate, (C) DALY, and (D) mortality rates of tetanus across 204 countries from 1990 to 2023.

DALY= disability-adjusted life year; SDI= Socio-demographic Index.

**Supplementary Tables**

**Table S1.** Prevalence, incidence, DALY, and mortality counts (95% UI) in 1990 and 2023.

|  | **Prevalence** | | **Incidence** | | **DALY** | | **Mortality** | |
| --- | --- | --- | --- | --- | --- | --- | --- | --- |
|  | **1990** | **2023** | **1990** | **2023** | **1990** | **2023** | **1990** | **2023** |
| **Global** | 138,724 (105,894 to 173,200) | 38,231 (27,743 to 49,536) | 657,360 (467,967 to 962,281) | 53,478 (30,198 to 93,877) | 23,657,221 (16,987,348 to 31,921,726) | 1,243,074 (748,566 to 1,921,250) | 290,705 (210,908 to 377,911) | 19,758 (11,735 to 31,048) |
| **High-income** | 147 (118 to 189) | 40 (26 to 58) | 1,485 (1,145 to 1,952) | 311 (187 to 513) | 8,235 (6,520 to 9,868) | 994 (682 to 1,312) | 308 (244 to 373) | 57 (38 to 78) |
| **Central Europe, Eastern Europe, and Central Asia** | 27 (20 to 35) | 5 (3 to 8) | 395 (301 to 544) | 29 (19 to 69) | 4,746 (3,694 to 5,871) | 263 (204 to 345) | 176 (140 to 219) | 10 (8 to 13) |
| **Latin America and Caribbean** | 1,337 (1,013 to 1,673) | 397 (262 to 583) | 6,977 (5,514 to 9,280) | 692 (449 to 1,241) | 173,392 (142,198 to 213,856) | 8,760 (6,118 to 12,889) | 2,659 (2,177 to 3,145) | 200 (152 to 281) |
| **North Africa and Middle East** | 1,897 (1,300 to 2,787) | 1,158 (858 to 1,524) | 15,692 (6,903 to 31,671) | 1,218 (606 to 2,674) | 658,081 (276,087 to 1,158,195) | 37,362 (19,754 to 67,894) | 7,847 (3,474 to 13,364) | 527 (287 to 900) |
| **South Asia** | 56,798 (44,148 to 69,642) | 17,013 (12,498 to 21,741) | 316,258 (233,296 to 418,506) | 11,877 (5,813 to 22,080) | 14,038,398 (11,082,591 to 18,018,213) | 330,041 (189,692 to 518,253) | 166,434 (132,582 to 209,330) | 4,790 (2,658 to 7,893) |
| **Southeast Asia, East Asia, and Oceania** | 65,323 (49,269 to 82,837) | 11,497 (8,431 to 15,126) | 219,013 (139,070 to 339,172) | 20,856 (9,203 to 41,176) | 4,575,000 (2,915,750 to 6,687,052) | 209,717 (108,112 to 372,761) | 60,749 (37,501 to 88,710) | 4,625 (2,121 to 8,435) |
| **Sub-Saharan Africa** | 13,196 (9,537 to 17,653) | 8,120 (5,711 to 10,664) | 97,539 (46,613 to 163,791) | 18,496 (9,342 to 35,998) | 4,199,369 (1,980,338 to 6,547,660) | 655,937 (357,378 to 1,080,795) | 52,533 (24,736 to 79,595) | 9,550 (5,324 to 16,001) |

DALY= disability-adjusted life year; UI= uncertainty interval.

**Table S2.** Age-standardized prevalence and DALY rate (95% UI) per 100,000 population in 1990 and 2023.

| Country | Age-standardized prevalence rate (95% UI) per 100,000 | | | | Age-standardized DALY rate per (95% UI) 100,000 | | |
| --- | --- | --- | --- | --- | --- | --- | --- |
|  | 1990 | 2023 | Total percentage change (%) | 1990 | | 2023 | Total percentage change (%) |
| Global | 2.5032 (1.9051 to 3.1237) | 0.4860 (0.3526 to 0.6290) | -80.5865 (-82.8570 to -78.0574) | 386.5975 (278.4726 to 515.8872) | | 17.8834 (10.8607 to 27.3813) | -95.3741 (-97.0757 to -92.6210) |
| Afghanistan | 1.5906 (0.8337 to 2.7766) | 0.5749 (0.3797 to 0.7600) | -63.8583 (-81.8615 to -34.0345) | 942.9652 (254.3325 to 2,004.6643) | | 32.0772 (13.5919 to 65.1593) | -96.5983 (-99.0061 to -85.6417) |
| Albania | 0.0037 (0.0022 to 0.0058) | 0.0007 (0.0003 to 0.0013) | -81.0396 (-90.8171 to -69.0783) | 0.7862 (0.4428 to 1.3698) | | 0.0127 (0.0051 to 0.0264) | -98.3828 (-99.3275 to -96.7172) |
| Algeria | 0.1988 (0.1377 to 0.2682) | 0.0895 (0.0707 to 0.1163) | -54.9923 (-63.8035 to -43.9511) | 19.4063 (5.8074 to 40.3183) | | 0.9226 (0.4005 to 1.7519) | -95.2458 (-98.1420 to -83.2570) |
| American Samoa | 0.0308 (0.0159 to 0.0685) | 0.0145 (0.0078 to 0.0239) | -52.7121 (-73.2907 to -28.6789) | 1.3696 (0.5465 to 2.7869) | | 0.2241 (0.0869 to 0.4238) | -83.6357 (-93.7148 to -58.1314) |
| Andorra | 0.0019 (0.0008 to 0.0067) | 0.0007 (0.0003 to 0.0014) | -63.8140 (-84.2638 to -32.9494) | 0.0915 (0.0405 to 0.1772) | | 0.0084 (0.0048 to 0.0145) | -90.7832 (-95.6174 to -77.8818) |
| Angola | 0.8361 (0.4272 to 1.5617) | 0.2264 (0.1428 to 0.5060) | -72.9230 (-83.7333 to -53.1585) | 346.6676 (164.1203 to 631.8710) | | 31.3070 (13.6429 to 73.3299) | -90.9692 (-96.0200 to -78.3532) |
| Antigua and Barbuda | 0.1086 (0.0903 to 0.1315) | 0.0954 (0.0764 to 0.1192) | -12.1933 (-22.0383 to -3.2122) | 1.2275 (0.4798 to 1.9133) | | 0.1206 (0.0645 to 0.1760) | -90.1731 (-94.5295 to -83.4194) |
| Argentina | 0.0830 (0.0625 to 0.1205) | 0.0083 (0.0042 to 0.0154) | -90.0330 (-94.0304 to -84.5364) | 4.9066 (3.5621 to 6.5404) | | 0.1249 (0.0718 to 0.2631) | -97.4545 (-98.5560 to -94.6289) |
| Armenia | 0.0029 (0.0018 to 0.0044) | 0.0005 (0.0002 to 0.0011) | -81.8335 (-91.8741 to -65.9477) | 0.6887 (0.3559 to 1.0043) | | 0.0144 (0.0084 to 0.0260) | -97.9059 (-98.7618 to -95.0867) |
| Australia | 0.0038 (0.0018 to 0.0077) | 0.0007 (0.0003 to 0.0013) | -82.1934 (-90.9616 to -65.8468) | 0.0718 (0.0386 to 0.1050) | | 0.0044 (0.0026 to 0.0079) | -93.8370 (-96.4020 to -88.9333) |
| Austria | 0.0012 (0.0004 to 0.0021) | 0.0010 (0.0003 to 0.0017) | -10.9475 (-55.1724 to 38.0945) | 0.0479 (0.0088 to 0.0805) | | 0.0335 (0.0014 to 0.0574) | -29.9719 (-85.9996 to 51.1404) |
| Azerbaijan | 0.0037 (0.0016 to 0.0134) | 0.0011 (0.0005 to 0.0024) | -70.0513 (-84.2145 to -45.4684) | 0.4758 (0.1951 to 1.1992) | | 0.0391 (0.0159 to 0.0965) | -91.7855 (-96.6404 to -82.5838) |
| Bahamas | 0.1109 (0.0812 to 0.1520) | 0.1045 (0.0784 to 0.1430) | -5.7737 (-12.8962 to 0.8762) | 0.1144 (0.0533 to 0.1930) | | 0.0445 (0.0258 to 0.0647) | -61.0737 (-77.7699 to -31.7621) |
| Bahrain | 0.1205 (0.1029 to 0.1426) | 0.1214 (0.1037 to 0.1406) | 0.7090 (-6.2015 to 7.7228) | 1.5610 (0.8478 to 2.7722) | | 0.1469 (0.0718 to 0.2642) | -90.5866 (-95.6475 to -81.1069) |
| Bangladesh | 3.8301 (2.9172 to 4.7239) | 1.8179 (1.2796 to 2.3936) | -52.5369 (-61.1103 to -43.6213) | 1,325.7941 (925.5951 to 1,776.9532) | | 29.7304 (14.9344 to 50.6547) | -97.7575 (-98.8025 to -95.8888) |
| Barbados | 0.2178 (0.1269 to 0.2946) | 0.0897 (0.0732 to 0.1086) | -58.8060 (-70.1055 to -29.0207) | 11.7107 (3.6744 to 16.7284) | | 0.5211 (0.2863 to 0.9148) | -95.5499 (-97.6894 to -88.8525) |
| Belarus | 0.0021 (0.0006 to 0.0033) | 0.0004 (0.0001 to 0.0008) | -79.9089 (-92.9278 to -41.4621) | 0.3471 (0.0546 to 0.5780) | | 0.0017 (0.0010 to 0.0031) | -99.5245 (-99.7649 to -97.2481) |
| Belgium | 0.0027 (0.0012 to 0.0042) | 0.0010 (0.0004 to 0.0018) | -62.5025 (-80.7759 to -26.0570) | 0.1494 (0.0448 to 0.2413) | | 0.0249 (0.0044 to 0.0499) | -83.3497 (-93.8249 to -56.7744) |
| Belize | 0.1919 (0.1413 to 0.2498) | 0.1046 (0.0763 to 0.1475) | -45.5179 (-56.6926 to -33.1568) | 6.9983 (2.6661 to 11.2678) | | 0.0559 (0.0382 to 0.0861) | -99.2016 (-99.5123 to -98.0135) |
| Benin | 1.8459 (1.2544 to 2.6738) | 0.9131 (0.6215 to 1.2199) | -50.5329 (-63.8454 to -35.7022) | 425.9970 (165.2606 to 736.3005) | | 35.4244 (16.6936 to 68.9787) | -91.6844 (-95.9963 to -79.8687) |
| Bermuda | 0.1115 (0.0889 to 0.1396) | 0.1074 (0.0869 to 0.1309) | -3.7342 (-11.3097 to 4.7370) | 0.1617 (0.0816 to 0.2402) | | 0.0546 (0.0362 to 0.0724) | -66.2256 (-76.1530 to -48.7354) |
| Bhutan | 2.7529 (1.9684 to 3.8852) | 1.1850 (0.8248 to 1.5960) | -56.9562 (-66.6173 to -44.9109) | 591.5342 (178.7983 to 1,140.6978) | | 7.8122 (2.7917 to 14.8324) | -98.6793 (-99.4876 to -96.5075) |
| Bolivia (Plurinational State of) | 0.7244 (0.4724 to 1.0507) | 0.1383 (0.0973 to 0.1907) | -80.9138 (-87.1653 to -72.7271) | 147.5736 (73.2385 to 240.3490) | | 3.5863 (1.4059 to 7.4029) | -97.5698 (-99.1560 to -94.8578) |
| Bosnia and Herzegovina | 0.0024 (0.0010 to 0.0042) | 0.0013 (0.0003 to 0.0024) | -46.0648 (-77.9893 to 18.8436) | 0.3021 (0.1058 to 0.5427) | | 0.1300 (0.0127 to 0.2690) | -56.9722 (-90.9578 to 20.3436) |
| Botswana | 0.2175 (0.1583 to 0.3030) | 0.1229 (0.1028 to 0.1573) | -43.4808 (-57.4080 to -22.3863) | 19.2091 (7.1935 to 38.3171) | | 3.1555 (1.1607 to 6.9425) | -83.5728 (-94.8534 to -27.9607) |
| Brazil | 0.4324 (0.3277 to 0.5494) | 0.0504 (0.0271 to 0.0823) | -88.3361 (-92.0631 to -84.2801) | 41.1299 (33.4114 to 51.1758) | | 1.0566 (0.7478 to 1.7361) | -97.4310 (-98.2520 to -96.2827) |
| Brunei Darussalam | 0.0018 (0.0004 to 0.0119) | 0.0010 (0.0004 to 0.0029) | -41.3165 (-77.4682 to 62.7585) | 0.0324 (0.0128 to 0.0682) | | 0.0151 (0.0026 to 0.0263) | -53.3929 (-90.4385 to 46.2377) |
| Bulgaria | 0.0040 (0.0024 to 0.0057) | 0.0006 (0.0002 to 0.0012) | -85.7123 (-93.9006 to -69.8862) | 0.6201 (0.3394 to 0.8792) | | 0.0208 (0.0122 to 0.0423) | -96.6508 (-98.2463 to -92.7247) |
| Burkina Faso | 1.5512 (1.0980 to 2.0549) | 0.6549 (0.4298 to 0.9164) | -57.7839 (-67.2572 to -49.0367) | 273.2912 (143.0008 to 428.5699) | | 19.3924 (9.7907 to 32.0693) | -92.9041 (-96.9458 to -83.5979) |
| Burundi | 1.4089 (0.9421 to 2.0323) | 0.7874 (0.5271 to 1.1078) | -44.1109 (-59.5475 to -27.4147) | 446.7495 (171.4550 to 803.2828) | | 74.3934 (20.9447 to 188.0103) | -83.3479 (-93.0945 to -63.9718) |
| Cabo Verde | 1.1568 (0.8065 to 1.6394) | 0.4302 (0.3042 to 0.5905) | -62.8101 (-71.5760 to -53.2692) | 131.8341 (43.2122 to 338.7083) | | 4.4053 (1.9187 to 8.9285) | -96.6585 (-99.0453 to -87.4999) |
| Cambodia | 7.2401 (3.1703 to 13.0575) | 1.3732 (0.9650 to 1.8215) | -81.0329 (-88.8683 to -61.9205) | 2,124.1919 (703.1347 to 3,629.2379) | | 39.4286 (12.4404 to 78.6165) | -98.1438 (-99.2371 to -95.9318) |
| Cameroon | 1.2977 (0.9083 to 1.8159) | 0.5598 (0.3604 to 0.8146) | -56.8575 (-66.8646 to -45.7570) | 245.8924 (116.5229 to 446.6185) | | 27.4233 (12.0731 to 51.3005) | -88.8474 (-94.9842 to -68.4166) |
| Canada | 0.0006 (0.0002 to 0.0012) | 0.0005 (0.0002 to 0.0010) | -13.0234 (-36.3562 to 8.9746) | 0.0077 (0.0038 to 0.0117) | | 0.0050 (0.0008 to 0.0081) | -34.9414 (-79.5159 to 8.1015) |
| Central African Republic | 0.7661 (0.4066 to 1.2591) | 0.5344 (0.3261 to 0.8374) | -30.2381 (-52.4826 to 9.9036) | 287.3407 (99.3306 to 504.4940) | | 152.1926 (50.8244 to 306.5605) | -47.0341 (-75.3048 to 23.6941) |
| Chad | 2.8175 (1.6879 to 4.6727) | 1.4200 (0.9869 to 1.9784) | -49.6014 (-66.0767 to -25.5147) | 862.4303 (330.9776 to 1,617.7286) | | 172.7978 (69.5954 to 348.2366) | -79.9638 (-91.3016 to -51.1097) |
| Chile | 0.0275 (0.0202 to 0.0401) | 0.0024 (0.0014 to 0.0039) | -91.2529 (-94.9071 to -85.0118) | 1.6992 (1.1170 to 2.3359) | | 0.0309 (0.0187 to 0.0563) | -98.1810 (-98.8639 to -96.5162) |
| China | 3.4147 (2.5447 to 4.3330) | 0.2524 (0.1652 to 0.3745) | -92.6088 (-94.4754 to -90.6480) | 136.0700 (85.1453 to 205.1648) | | 0.7099 (0.3797 to 1.2789) | -99.4783 (-99.7383 to -98.9275) |
| Colombia | 0.2282 (0.1708 to 0.2963) | 0.0378 (0.0222 to 0.0576) | -83.4195 (-87.5404 to -78.6071) | 31.6685 (26.2194 to 37.1877) | | 0.9214 (0.6245 to 1.3303) | -97.0905 (-98.0558 to -95.5150) |
| Comoros | 0.9406 (0.6606 to 1.3261) | 0.3761 (0.2676 to 0.5314) | -60.0116 (-69.4715 to -49.0978) | 203.4827 (95.2741 to 393.2169) | | 15.5098 (8.1037 to 26.6982) | -92.3778 (-96.5666 to -80.0749) |
| Congo | 0.2096 (0.1374 to 0.3262) | 0.1257 (0.0953 to 0.1832) | -40.0232 (-59.4110 to -8.7353) | 45.7626 (22.1714 to 80.5005) | | 11.8415 (4.6489 to 26.1247) | -74.1240 (-90.6542 to -31.6241) |
| Cook Islands | 0.0289 (0.0136 to 0.0537) | 0.0201 (0.0099 to 0.0352) | -30.6569 (-59.6249 to -6.5437) | 0.6719 (0.1001 to 1.7901) | | 0.0760 (0.0121 to 0.2070) | -88.6888 (-95.1499 to -58.0502) |
| Costa Rica | 0.0052 (0.0032 to 0.0079) | 0.0031 (0.0015 to 0.0052) | -39.8748 (-61.2723 to -17.8516) | 0.4570 (0.1402 to 0.6520) | | 0.0128 (0.0038 to 0.0239) | -97.1918 (-98.4548 to -94.8927) |
| Croatia | 0.0176 (0.0035 to 0.0280) | 0.0007 (0.0003 to 0.0013) | -96.1493 (-98.3585 to -79.0542) | 1.8176 (0.5123 to 2.6532) | | 0.0217 (0.0116 to 0.0400) | -98.8062 (-99.4318 to -96.4798) |
| Cuba | 0.1492 (0.1145 to 0.1942) | 0.1403 (0.1100 to 0.1863) | -5.9898 (-12.8204 to 1.5709) | 0.4172 (0.0941 to 0.6657) | | 0.0371 (0.0258 to 0.0507) | -91.1058 (-95.3232 to -58.9282) |
| Cyprus | 0.0027 (0.0008 to 0.0065) | 0.0010 (0.0003 to 0.0017) | -63.4846 (-84.2998 to -9.5587) | 0.1115 (0.0213 to 0.2769) | | 0.0291 (0.0020 to 0.0618) | -73.8781 (-94.0899 to 3.1597) |
| Czechia | 0.0009 (0.0005 to 0.0016) | 0.0005 (0.0002 to 0.0010) | -46.9916 (-70.0994 to -23.1120) | 0.0657 (0.0316 to 0.1130) | | 0.0078 (0.0022 to 0.0139) | -88.1369 (-95.1239 to -70.1602) |
| Côte d'Ivoire | 1.8224 (1.2707 to 2.4562) | 1.0540 (0.7383 to 1.3837) | -42.1674 (-52.7742 to -30.8072) | 277.2134 (136.5389 to 477.2667) | | 36.9953 (18.1761 to 63.7603) | -86.6546 (-93.3281 to -67.2980) |
| Democratic People's Republic of Korea | 0.7803 (0.5618 to 1.0489) | 0.5038 (0.3741 to 0.6736) | -35.4435 (-47.5756 to -19.5896) | 24.0528 (11.1792 to 41.1966) | | 4.4818 (1.5009 to 10.9188) | -81.3669 (-92.6381 to -54.4427) |
| Democratic Republic of the Congo | 0.7180 (0.4347 to 1.2164) | 0.3832 (0.2609 to 0.6061) | -46.6268 (-61.6742 to -26.3844) | 164.2227 (80.3791 to 284.2775) | | 40.7120 (15.7813 to 80.6973) | -75.2093 (-88.7783 to -47.8197) |
| Denmark | 0.0006 (0.0002 to 0.0015) | 0.0009 (0.0004 to 0.0015) | 44.6237 (-28.3166 to 212.4654) | 0.0052 (0.0034 to 0.0108) | | 0.0282 (0.0083 to 0.0394) | 442.0924 (-4.5553 to 926.4770) |
| Djibouti | 0.8344 (0.5680 to 1.2366) | 0.4365 (0.2914 to 0.6846) | -47.6905 (-59.6096 to -32.4512) | 132.2585 (59.8792 to 245.4728) | | 30.2312 (11.5515 to 70.6859) | -77.1423 (-90.0372 to -33.9493) |
| Dominica | 0.1112 (0.0924 to 0.1413) | 0.0826 (0.0671 to 0.1041) | -25.7266 (-32.9690 to -16.9802) | 1.7022 (1.1110 to 2.3343) | | 0.3594 (0.1885 to 0.6009) | -78.8841 (-88.1754 to -64.7584) |
| Dominican Republic | 0.5714 (0.4034 to 1.0125) | 0.2580 (0.1833 to 0.3866) | -54.8529 (-70.0051 to -41.0071) | 34.9071 (23.5434 to 56.5630) | | 6.6209 (3.7636 to 12.0753) | -81.0329 (-89.4050 to -67.6537) |
| Ecuador | 0.3865 (0.2982 to 0.4843) | 0.1060 (0.0821 to 0.1386) | -72.5635 (-77.3944 to -66.6878) | 41.7572 (25.2830 to 53.3649) | | 1.3878 (0.9258 to 2.2541) | -96.6764 (-97.9355 to -94.0761) |
| Egypt | 0.5854 (0.4316 to 0.8477) | 0.2535 (0.1810 to 0.3386) | -56.6947 (-67.9464 to -48.7941) | 86.9172 (57.1235 to 131.2909) | | 5.3526 (2.9433 to 8.4332) | -93.8417 (-97.1309 to -88.3795) |
| El Salvador | 0.1860 (0.1114 to 0.2707) | 0.0213 (0.0102 to 0.0372) | -88.5485 (-93.9167 to -79.9756) | 39.7879 (18.0299 to 67.7068) | | 0.2130 (0.0673 to 0.5679) | -99.4648 (-99.8651 to -97.8190) |
| Equatorial Guinea | 0.9070 (0.3993 to 2.0053) | 0.1045 (0.0775 to 0.1772) | -88.4771 (-93.5404 to -74.2676) | 439.1204 (194.9058 to 855.8188) | | 4.3183 (1.1961 to 11.1230) | -99.0166 (-99.7105 to -96.9868) |
| Eritrea | 2.7414 (1.7271 to 4.8676) | 0.5556 (0.3965 to 0.7449) | -79.7315 (-87.5429 to -67.2881) | 1,433.8729 (736.8674 to 2,482.0630) | | 48.5533 (25.5814 to 90.4448) | -96.6138 (-98.3972 to -92.1048) |
| Estonia | 0.0007 (0.0003 to 0.0016) | 0.0005 (0.0002 to 0.0009) | -20.0321 (-60.9944 to 9.5327) | 0.0388 (0.0165 to 0.0568) | | 0.0159 (0.0011 to 0.0299) | -58.9833 (-94.5202 to -7.0109) |
| Eswatini | 0.3196 (0.2045 to 0.4753) | 0.1442 (0.1093 to 0.1895) | -54.8699 (-70.0709 to -35.8098) | 35.9449 (14.1188 to 72.9952) | | 7.9212 (3.7190 to 14.4326) | -77.9630 (-92.3085 to -24.1374) |
| Ethiopia | 6.2855 (4.2015 to 8.9044) | 1.0178 (0.7307 to 1.3672) | -83.8068 (-88.5714 to -77.3090) | 2,030.1691 (813.9084 to 3,288.5134) | | 61.5484 (30.4294 to 110.7495) | -96.9683 (-98.5904 to -91.9237) |
| Fiji | 0.0081 (0.0028 to 0.0193) | 0.0067 (0.0025 to 0.0140) | -17.8900 (-32.8904 to -5.0930) | 0.0912 (0.0240 to 0.2457) | | 0.0163 (0.0076 to 0.0303) | -82.1728 (-94.8547 to -28.4584) |
| Finland | 0.0012 (0.0006 to 0.0023) | 0.0010 (0.0005 to 0.0019) | -13.2875 (-46.3754 to 1.4762) | 0.0097 (0.0057 to 0.0170) | | 0.0031 (0.0006 to 0.0062) | -67.8345 (-90.1662 to -41.1833) |
| France | 0.0168 (0.0047 to 0.0234) | 0.0013 (0.0007 to 0.0021) | -92.4840 (-95.8143 to -76.2598) | 0.8976 (0.2771 to 1.1617) | | 0.0186 (0.0094 to 0.0319) | -97.9255 (-98.9142 to -94.7723) |
| Gabon | 0.1663 (0.1157 to 0.2808) | 0.1098 (0.0909 to 0.1641) | -33.9804 (-57.7022 to -11.8796) | 31.4235 (13.1781 to 64.0085) | | 4.7929 (1.5497 to 11.6914) | -84.7474 (-94.7190 to -63.4434) |
| Gambia | 1.1206 (0.7726 to 1.4726) | 0.6130 (0.3976 to 0.8665) | -45.2937 (-55.3470 to -35.3556) | 105.2651 (66.3192 to 150.0125) | | 18.0424 (7.3927 to 38.1589) | -82.8600 (-93.1229 to -63.3471) |
| Georgia | 0.0037 (0.0014 to 0.0060) | 0.0014 (0.0006 to 0.0024) | -61.8052 (-78.2390 to -34.5216) | 0.6796 (0.1840 to 1.0605) | | 0.1579 (0.0245 to 0.3232) | -76.7610 (-89.1353 to -46.4675) |
| Germany | 0.0019 (0.0008 to 0.0032) | 0.0005 (0.0002 to 0.0010) | -74.0051 (-89.3593 to -46.6202) | 0.1103 (0.0383 to 0.1745) | | 0.0017 (0.0010 to 0.0035) | -98.4408 (-99.1631 to -92.3118) |
| Ghana | 1.4202 (1.0307 to 1.8798) | 0.6065 (0.4179 to 0.8385) | -57.2975 (-67.7189 to -48.3531) | 301.4305 (165.3150 to 526.7923) | | 19.3046 (8.7625 to 35.0907) | -93.5957 (-97.2725 to -85.8451) |
| Greece | 0.0029 (0.0018 to 0.0062) | 0.0016 (0.0007 to 0.0025) | -45.5300 (-84.4771 to -0.5785) | 0.2098 (0.1438 to 0.3051) | | 0.0947 (0.0310 to 0.1604) | -54.8696 (-88.8704 to -7.1622) |
| Greenland | 0.0027 (0.0011 to 0.0123) | 0.0016 (0.0007 to 0.0030) | -41.3992 (-77.8655 to 41.3945) | 0.2488 (0.1117 to 0.6585) | | 0.1010 (0.0324 to 0.1812) | -59.4034 (-82.2252 to -0.7752) |
| Grenada | 0.2770 (0.2127 to 0.3458) | 0.1164 (0.0871 to 0.1610) | -57.9691 (-65.0029 to -50.2999) | 6.0304 (4.3232 to 7.7997) | | 0.1570 (0.1090 to 0.2399) | -97.3961 (-98.2963 to -95.8986) |
| Guam | 0.0146 (0.0072 to 0.0276) | 0.0128 (0.0059 to 0.0222) | -12.2921 (-33.3322 to 3.2136) | 0.0561 (0.0162 to 0.1781) | | 0.0414 (0.0212 to 0.0722) | -26.1939 (-86.7648 to 122.0084) |
| Guatemala | 0.1644 (0.1189 to 0.2349) | 0.0103 (0.0064 to 0.0169) | -93.7173 (-96.1426 to -88.1757) | 57.0653 (39.3303 to 79.5219) | | 1.2502 (0.7338 to 2.2730) | -97.8092 (-98.7138 to -95.8089) |
| Guinea | 1.4230 (0.9672 to 2.0779) | 0.5586 (0.3612 to 0.8068) | -60.7489 (-69.6825 to -48.5799) | 493.5483 (261.2153 to 877.5725) | | 44.7436 (19.2825 to 93.1411) | -90.9343 (-96.1963 to -76.1914) |
| Guinea-Bissau | 2.4884 (1.7223 to 3.5265) | 1.3861 (0.9852 to 1.7777) | -44.2974 (-60.4690 to -26.9424) | 803.1885 (339.7881 to 1,429.3959) | | 91.7251 (43.8531 to 170.9139) | -88.5799 (-94.3853 to -72.1303) |
| Guyana | 0.1194 (0.0946 to 0.1519) | 0.0644 (0.0506 to 0.0857) | -46.0623 (-55.6880 to -34.7787) | 3.6874 (2.2156 to 5.2809) | | 0.2164 (0.0614 to 0.5478) | -94.1327 (-97.7564 to -83.9519) |
| Haiti | 1.4339 (0.9046 to 2.2406) | 0.5474 (0.3742 to 0.7499) | -61.8214 (-75.4487 to -42.3335) | 292.0307 (126.8479 to 536.2321) | | 17.6678 (6.4727 to 40.1354) | -93.9500 (-97.9832 to -81.1160) |
| Honduras | 0.0222 (0.0104 to 0.0379) | 0.0046 (0.0023 to 0.0095) | -79.0917 (-90.3938 to -59.2673) | 11.1265 (4.7560 to 18.2094) | | 0.9153 (0.2209 to 2.2540) | -91.7740 (-97.5224 to -80.1351) |
| Hungary | 0.0126 (0.0057 to 0.0187) | 0.0005 (0.0002 to 0.0011) | -95.8052 (-98.5439 to -85.4051) | 2.1249 (1.0108 to 3.1812) | | 0.0136 (0.0073 to 0.0319) | -99.3610 (-99.7203 to -97.1028) |
| Iceland | 0.0012 (0.0007 to 0.0022) | 0.0017 (0.0007 to 0.0025) | 35.8256 (-28.8716 to 104.7959) | 0.0283 (0.0096 to 0.0521) | | 0.0575 (0.0052 to 0.0914) | 102.9561 (-56.7576 to 231.3365) |
| India | 4.6518 (3.5138 to 5.7646) | 0.6209 (0.4625 to 0.7825) | -86.6519 (-88.0915 to -85.2287) | 872.3840 (679.5964 to 1,119.3533) | | 13.4645 (7.4594 to 24.4574) | -98.4566 (-99.1415 to -97.0986) |
| Indonesia | 9.2452 (6.9674 to 12.2558) | 1.5133 (1.1263 to 1.9867) | -83.6315 (-86.6161 to -80.1241) | 749.4142 (357.7530 to 1,201.6104) | | 38.7663 (16.9624 to 70.4480) | -94.8271 (-97.8535 to -88.1743) |
| Iran (Islamic Republic of) | 0.2958 (0.2081 to 0.4230) | 0.0416 (0.0224 to 0.0683) | -85.9379 (-90.5738 to -81.3397) | 45.4662 (18.4408 to 88.1154) | | 0.4437 (0.2610 to 0.7365) | -99.0241 (-99.5616 to -97.1249) |
| Iraq | 0.5258 (0.3614 to 0.7476) | 0.1901 (0.1405 to 0.2653) | -63.8467 (-72.9467 to -54.7532) | 79.4414 (29.6299 to 171.4722) | | 3.3558 (1.5987 to 6.1819) | -95.7757 (-98.4703 to -86.3770) |
| Ireland | 0.0011 (0.0005 to 0.0039) | 0.0007 (0.0003 to 0.0012) | -39.8360 (-81.7954 to -6.2910) | 0.0393 (0.0276 to 0.0553) | | 0.0104 (0.0019 to 0.0176) | -73.6176 (-93.8563 to -47.3612) |
| Israel | 0.0011 (0.0005 to 0.0021) | 0.0009 (0.0004 to 0.0017) | -15.0146 (-47.9516 to -0.5505) | 0.0081 (0.0056 to 0.0133) | | 0.0025 (0.0009 to 0.0050) | -68.6930 (-87.7141 to -45.4596) |
| Italy | 0.0105 (0.0064 to 0.0207) | 0.0030 (0.0018 to 0.0044) | -71.4419 (-87.8080 to -52.7863) | 1.4575 (0.9797 to 2.0011) | | 0.2611 (0.1451 to 0.3947) | -82.0849 (-90.4043 to -68.7427) |
| Jamaica | 0.1862 (0.1470 to 0.2372) | 0.0829 (0.0653 to 0.1119) | -55.4517 (-65.2113 to -44.4162) | 8.3707 (4.4146 to 12.8400) | | 0.2040 (0.1234 to 0.3688) | -97.5633 (-98.7724 to -94.4886) |
| Japan | 0.0152 (0.0104 to 0.0221) | 0.0059 (0.0038 to 0.0084) | -60.8483 (-72.7004 to -45.5154) | 0.3932 (0.2790 to 0.5339) | | 0.0918 (0.0536 to 0.1319) | -76.6660 (-85.4584 to -61.7474) |
| Jordan | 0.1689 (0.1240 to 0.2215) | 0.0990 (0.0790 to 0.1260) | -41.3888 (-50.2233 to -30.4319) | 9.3403 (3.4692 to 21.6621) | | 0.2953 (0.1464 to 0.4941) | -96.8387 (-98.9109 to -90.5106) |
| Kazakhstan | 0.0061 (0.0028 to 0.0113) | 0.0013 (0.0007 to 0.0023) | -78.0704 (-89.9366 to -56.1562) | 1.1376 (0.3124 to 1.6782) | | 0.0085 (0.0050 to 0.0165) | -99.2485 (-99.5683 to -97.6073) |
| Kenya | 0.6960 (0.4847 to 0.9690) | 0.3683 (0.2876 to 0.4768) | -47.0780 (-55.8681 to -37.3600) | 102.0683 (42.6984 to 177.0916) | | 40.6932 (18.7605 to 69.3279) | -60.1314 (-83.9354 to -3.9023) |
| Kiribati | 0.1985 (0.1199 to 0.2904) | 0.1239 (0.0818 to 0.1802) | -37.5674 (-48.4081 to -24.4065) | 5.1692 (2.3491 to 9.6079) | | 1.3210 (0.5903 to 3.1271) | -74.4439 (-91.7011 to -26.5727) |
| Kuwait | 0.1426 (0.1105 to 0.1852) | 0.1394 (0.1112 to 0.1804) | -2.2407 (-8.0982 to 4.5153) | 0.0568 (0.0424 to 0.0767) | | 0.0409 (0.0285 to 0.0555) | -28.1309 (-44.3823 to -9.5098) |
| Kyrgyzstan | 0.0014 (0.0006 to 0.0039) | 0.0008 (0.0003 to 0.0016) | -42.8038 (-76.0182 to -11.8409) | 0.1089 (0.0142 to 0.1659) | | 0.0068 (0.0022 to 0.0152) | -93.7715 (-96.9960 to -81.0254) |
| Lao People's Democratic Republic | 24.1775 (11.8506 to 42.2697) | 2.3447 (1.5719 to 3.4692) | -90.3022 (-94.9067 to -78.6999) | 6,779.6362 (3,539.7095 to 10,684.6780) | | 182.2157 (81.5804 to 325.4978) | -97.3123 (-98.9286 to -93.2146) |
| Latvia | 0.0005 (0.0002 to 0.0013) | 0.0009 (0.0003 to 0.0016) | 66.4681 (-40.8788 to 328.1034) | 0.0097 (0.0062 to 0.0165) | | 0.0806 (0.0012 to 0.1558) | 733.1215 (-91.2404 to 1,860.5889) |
| Lebanon | 0.3913 (0.2685 to 0.5608) | 0.1614 (0.1261 to 0.2043) | -58.7621 (-67.9635 to -47.8164) | 48.1595 (20.0625 to 88.7625) | | 3.4286 (1.8783 to 5.1924) | -92.8807 (-96.6059 to -81.0350) |
| Lesotho | 0.4464 (0.2721 to 0.7590) | 0.2533 (0.1759 to 0.3612) | -43.2469 (-63.2496 to -21.8517) | 52.4678 (14.1207 to 125.5975) | | 16.0234 (5.9042 to 34.1651) | -69.4604 (-89.5723 to 0.8822) |
| Liberia | 1.2454 (0.8722 to 1.7228) | 0.5476 (0.3320 to 0.8214) | -56.0329 (-67.3243 to -43.3208) | 206.2287 (112.1546 to 333.9334) | | 15.5707 (4.9244 to 41.2573) | -92.4498 (-97.3523 to -81.9056) |
| Libya | 0.1012 (0.0840 to 0.1264) | 0.0860 (0.0730 to 0.1007) | -14.9806 (-26.0465 to -5.2031) | 4.1901 (1.6344 to 8.2271) | | 0.4270 (0.1259 to 0.9542) | -89.8092 (-96.5419 to -69.0506) |
| Lithuania | 0.0026 (0.0015 to 0.0040) | 0.0006 (0.0002 to 0.0011) | -76.7276 (-90.1360 to -58.0440) | 0.3648 (0.1757 to 0.5745) | | 0.0172 (0.0047 to 0.0293) | -95.2928 (-98.0185 to -89.9736) |
| Luxembourg | 0.0010 (0.0005 to 0.0028) | 0.0011 (0.0003 to 0.0019) | 10.5696 (-75.0770 to 101.9230) | 0.0367 (0.0254 to 0.0604) | | 0.0373 (0.0061 to 0.0636) | 1.5377 (-87.7781 to 98.8070) |
| Madagascar | 1.1505 (0.8573 to 1.5081) | 0.5511 (0.3779 to 0.7894) | -52.0992 (-61.2741 to -39.6151) | 303.4543 (214.6729 to 388.8867) | | 55.8144 (29.8759 to 102.1363) | -81.6070 (-90.1318 to -64.6960) |
| Malawi | 1.1277 (0.7721 to 1.6828) | 0.6251 (0.4342 to 0.8188) | -44.5621 (-61.8536 to -24.9282) | 281.4096 (152.4476 to 506.5826) | | 50.9614 (26.1738 to 89.5045) | -81.8907 (-91.9427 to -54.7379) |
| Malaysia | 0.6941 (0.5232 to 0.9841) | 0.3816 (0.3402 to 0.4403) | -45.0173 (-58.9882 to -28.7711) | 41.0504 (18.1687 to 75.2740) | | 3.1240 (1.5722 to 5.3349) | -92.3898 (-96.4072 to -78.4342) |
| Maldives | 1.8691 (1.0476 to 3.4035) | 0.3300 (0.2697 to 0.4082) | -82.3440 (-89.7538 to -68.9515) | 260.9139 (97.4841 to 481.2722) | | 4.8833 (2.4186 to 9.6786) | -98.1284 (-99.2763 to -93.6383) |
| Mali | 2.2735 (1.3966 to 3.7516) | 1.0882 (0.7669 to 1.4555) | -52.1338 (-68.9941 to -31.6442) | 545.6437 (210.7809 to 1,121.2569) | | 65.1349 (26.4807 to 124.9793) | -88.0627 (-93.7772 to -72.6387) |
| Malta | 0.0067 (0.0032 to 0.0096) | 0.0014 (0.0008 to 0.0023) | -78.9445 (-89.2275 to -62.0544) | 0.4932 (0.1745 to 0.6934) | | 0.0106 (0.0037 to 0.0178) | -97.8602 (-98.7460 to -96.1757) |
| Marshall Islands | 0.0329 (0.0104 to 0.1157) | 0.0151 (0.0073 to 0.0431) | -53.9757 (-76.4269 to 25.6054) | 1.4396 (0.3799 to 3.3158) | | 0.5109 (0.1658 to 1.2198) | -64.5101 (-84.4540 to 35.7050) |
| Mauritania | 1.5102 (1.0331 to 2.0795) | 0.6251 (0.4328 to 0.8884) | -58.6046 (-69.0649 to -47.0804) | 261.3768 (127.6058 to 493.7932) | | 15.6719 (6.6079 to 30.9055) | -94.0041 (-97.2956 to -84.6256) |
| Mauritius | 0.3448 (0.2827 to 0.4243) | 0.3308 (0.2543 to 0.4404) | -4.0503 (-12.8499 to 9.2963) | 3.0631 (1.0616 to 4.5160) | | 0.0908 (0.0658 to 0.1173) | -97.0360 (-98.1970 to -91.3498) |
| Mexico | 0.2969 (0.2234 to 0.3756) | 0.0366 (0.0185 to 0.0618) | -87.6603 (-92.3041 to -83.1442) | 35.2039 (26.4760 to 41.9425) | | 0.2614 (0.1747 to 0.4463) | -99.2575 (-99.5049 to -98.6938) |
| Micronesia (Federated States of) | 0.8247 (0.0533 to 2.1239) | 0.4197 (0.0339 to 1.1189) | -49.1128 (-87.3185 to 190.0786) | 78.8285 (5.7008 to 185.8544) | | 29.3193 (2.7040 to 63.0786) | -62.8062 (-88.4195 to 118.6113) |
| Monaco | 0.0014 (0.0007 to 0.0031) | 0.0009 (0.0005 to 0.0018) | -33.0765 (-65.7383 to 18.8197) | 0.0743 (0.0320 to 0.1600) | | 0.0370 (0.0163 to 0.0700) | -50.1868 (-80.2061 to 50.6086) |
| Mongolia | 0.0021 (0.0010 to 0.0063) | 0.0017 (0.0008 to 0.0031) | -20.7734 (-57.2426 to -5.2603) | 0.0635 (0.0204 to 0.1737) | | 0.0005 (0.0003 to 0.0009) | -99.1946 (-99.7272 to -97.4750) |
| Montenegro | 0.0012 (0.0005 to 0.0024) | 0.0008 (0.0003 to 0.0018) | -35.5926 (-71.3882 to 34.3903) | 0.1099 (0.0334 to 0.2513) | | 0.0456 (0.0110 to 0.1358) | -58.5303 (-90.0560 to 66.2188) |
| Morocco | 0.9228 (0.6529 to 1.2651) | 0.2490 (0.1711 to 0.3527) | -73.0130 (-80.3961 to -65.6429) | 215.4160 (113.8522 to 379.1363) | | 3.5752 (1.2996 to 8.3060) | -98.3403 (-99.4165 to -95.8366) |
| Mozambique | 1.4255 (0.9681 to 2.1366) | 0.5662 (0.3857 to 0.7518) | -60.2845 (-73.0919 to -44.0737) | 486.7140 (260.6651 to 855.5472) | | 46.6335 (26.6833 to 74.9363) | -90.4187 (-94.9976 to -78.9929) |
| Myanmar | 4.4373 (2.1551 to 7.6834) | 1.5578 (1.0801 to 2.1551) | -64.8935 (-79.8992 to -35.8974) | 1,131.1418 (364.0901 to 2,181.4817) | | 64.2233 (27.2665 to 127.4534) | -94.3223 (-97.5382 to -85.3786) |
| Namibia | 0.5039 (0.3138 to 1.0129) | 0.1348 (0.1050 to 0.1722) | -73.2416 (-84.0573 to -61.7649) | 81.8628 (39.2995 to 175.5816) | | 5.4418 (2.5623 to 9.9781) | -93.3526 (-97.0080 to -82.9442) |
| Nauru | 0.0161 (0.0056 to 0.0673) | 0.0086 (0.0038 to 0.0307) | -46.7137 (-72.1203 to 45.2390) | 0.5589 (0.1934 to 1.1404) | | 0.2737 (0.0905 to 0.6433) | -51.0198 (-81.2425 to 51.8241) |
| Nepal | 5.9322 (3.8929 to 9.1272) | 2.1077 (1.5432 to 2.7142) | -64.4704 (-78.8848 to -47.4982) | 2,467.7330 (1,078.9254 to 4,533.6144) | | 44.6564 (19.5170 to 89.4744) | -98.1904 (-99.2318 to -95.1509) |
| Netherlands | 0.0010 (0.0004 to 0.0018) | 0.0010 (0.0005 to 0.0017) | 2.3145 (-27.0756 to 26.7012) | 0.0095 (0.0062 to 0.0145) | | 0.0150 (0.0030 to 0.0228) | 58.3039 (-64.4416 to 172.1470) |
| New Zealand | 0.0025 (0.0012 to 0.0068) | 0.0009 (0.0004 to 0.0016) | -64.8358 (-79.0998 to -42.9533) | 0.0408 (0.0175 to 0.0599) | | 0.0050 (0.0015 to 0.0094) | -87.6782 (-93.4859 to -77.1079) |
| Nicaragua | 0.0314 (0.0084 to 0.0499) | 0.0028 (0.0013 to 0.0055) | -91.0464 (-96.2293 to -69.1491) | 8.9070 (1.6792 to 14.9169) | | 0.0196 (0.0074 to 0.0467) | -99.7798 (-99.9370 to -98.2461) |
| Niger | 2.2602 (1.5266 to 3.4751) | 0.9697 (0.6948 to 1.2852) | -57.0945 (-73.3780 to -42.0744) | 619.0228 (311.2204 to 1,129.8014) | | 56.8425 (29.9177 to 97.5780) | -90.8174 (-96.1866 to -79.1679) |
| Nigeria | 5.1680 (3.7065 to 6.8987) | 0.8148 (0.5779 to 1.0659) | -84.2328 (-86.4977 to -81.4840) | 488.8511 (168.4273 to 846.5616) | | 25.9478 (12.4297 to 46.6172) | -94.6921 (-97.6132 to -85.3669) |
| Niue | 0.0080 (0.0036 to 0.0249) | 0.0054 (0.0028 to 0.0135) | -31.7241 (-58.3413 to 32.3519) | 0.1740 (0.0677 to 0.3461) | | 0.0909 (0.0356 to 0.1924) | -47.7694 (-79.8933 to 63.9445) |
| North Macedonia | 0.0010 (0.0004 to 0.0021) | 0.0005 (0.0002 to 0.0010) | -52.2725 (-78.5028 to -10.9204) | 0.0957 (0.0234 to 0.1693) | | 0.0092 (0.0031 to 0.0282) | -90.3759 (-96.5567 to -67.8597) |
| Northern Mariana Islands | 0.1923 (0.0682 to 0.7365) | 0.0311 (0.0175 to 0.0524) | -83.8167 (-94.8923 to -58.8015) | 6.9667 (2.9874 to 20.5731) | | 0.9828 (0.4458 to 1.6129) | -85.8926 (-94.3062 to -64.4909) |
| Norway | 0.0012 (0.0006 to 0.0023) | 0.0020 (0.0010 to 0.0033) | 74.3972 (-37.2789 to 216.7416) | 0.0157 (0.0103 to 0.0257) | | 0.0695 (0.0097 to 0.1170) | 343.6850 (-56.5741 to 768.7611) |
| Oman | 0.2033 (0.1482 to 0.2996) | 0.1139 (0.0971 to 0.1374) | -43.9705 (-59.4651 to -30.1107) | 22.2490 (4.1773 to 61.4215) | | 0.4176 (0.1902 to 0.7911) | -98.1228 (-99.4769 to -87.9310) |
| Pakistan | 7.3123 (5.3531 to 9.6778) | 1.7367 (1.2683 to 2.2357) | -76.2499 (-80.6078 to -72.2654) | 782.4908 (438.7910 to 1,263.5495) | | 44.4168 (20.6212 to 84.8579) | -94.3237 (-97.2615 to -89.4863) |
| Palau | 0.0090 (0.0037 to 0.0230) | 0.0076 (0.0034 to 0.0145) | -16.4033 (-43.5654 to 0.6852) | 0.0391 (0.0064 to 0.1404) | | 0.0147 (0.0024 to 0.0559) | -62.4157 (-88.1008 to 19.8871) |
| Palestine | 0.1189 (0.0974 to 0.1499) | 0.0915 (0.0752 to 0.1108) | -23.0487 (-33.9645 to -12.7984) | 4.5990 (1.7877 to 8.6119) | | 0.3546 (0.1707 to 0.5985) | -92.2899 (-96.6105 to -77.1040) |
| Panama | 0.0219 (0.0084 to 0.0347) | 0.0023 (0.0011 to 0.0039) | -89.4546 (-94.9504 to -70.2223) | 6.9090 (2.7677 to 10.0672) | | 0.0126 (0.0070 to 0.0242) | -99.8183 (-99.9164 to -99.4759) |
| Papua New Guinea | 0.0992 (0.0400 to 0.2630) | 0.0810 (0.0260 to 0.2522) | -18.2954 (-59.6555 to 93.4745) | 3.8337 (0.6409 to 9.2321) | | 2.7532 (0.4922 to 10.3259) | -28.1838 (-82.7352 to 91.6658) |
| Paraguay | 0.4923 (0.3608 to 0.6417) | 0.1445 (0.1092 to 0.1947) | -70.6443 (-74.9664 to -65.6270) | 53.4748 (38.1970 to 70.2684) | | 1.3418 (0.6035 to 3.2522) | -97.4907 (-98.9772 to -93.7914) |
| Peru | 0.2173 (0.1614 to 0.2789) | 0.0899 (0.0704 to 0.1186) | -58.6385 (-66.0007 to -49.2076) | 16.9790 (10.3897 to 23.7558) | | 0.8729 (0.3800 to 2.2930) | -94.8588 (-98.0513 to -88.1016) |
| Philippines | 4.2255 (3.0597 to 5.4257) | 1.0369 (0.7988 to 1.3390) | -75.4613 (-77.8204 to -71.7380) | 142.8029 (89.2645 to 202.7791) | | 24.0602 (12.2650 to 38.2277) | -83.1514 (-91.1024 to -69.4943) |
| Poland | 0.0090 (0.0052 to 0.0136) | 0.0006 (0.0002 to 0.0011) | -93.8756 (-97.7861 to -85.2341) | 1.7097 (1.0545 to 2.4007) | | 0.0133 (0.0079 to 0.0277) | -99.2206 (-99.6089 to -97.7376) |
| Portugal | 0.0598 (0.0456 to 0.0779) | 0.0024 (0.0012 to 0.0040) | -95.9808 (-98.0876 to -92.7849) | 4.3656 (2.7950 to 5.8975) | | 0.0072 (0.0038 to 0.0154) | -99.8350 (-99.9279 to -99.4808) |
| Puerto Rico | 0.0898 (0.0733 to 0.1111) | 0.0840 (0.0693 to 0.1011) | -6.4672 (-15.8415 to 1.3504) | 0.5424 (0.3610 to 0.8274) | | 0.1521 (0.0612 to 0.2442) | -71.9667 (-91.6361 to -47.6950) |
| Qatar | 0.1473 (0.1264 to 0.1788) | 0.1317 (0.1135 to 0.1514) | -10.5346 (-26.1139 to -0.2987) | 4.1781 (2.0342 to 8.8380) | | 0.6050 (0.2910 to 1.0186) | -85.5194 (-96.0110 to -65.9161) |
| Republic of Korea | 0.0924 (0.0684 to 0.1345) | 0.0117 (0.0074 to 0.0178) | -87.3349 (-92.0814 to -81.5965) | 5.2672 (3.5514 to 7.8787) | | 0.1371 (0.0843 to 0.2177) | -97.3967 (-98.6113 to -95.3128) |
| Republic of Moldova | 0.0007 (0.0003 to 0.0031) | 0.0004 (0.0002 to 0.0009) | -36.5768 (-73.4637 to -7.0068) | 0.0315 (0.0217 to 0.0490) | | 0.0043 (0.0015 to 0.0108) | -86.4072 (-94.2249 to -73.7754) |
| Romania | 0.0066 (0.0041 to 0.0161) | 0.0012 (0.0006 to 0.0020) | -82.1145 (-94.4175 to -62.5856) | 1.5783 (1.0942 to 2.2484) | | 0.1417 (0.0726 to 0.2579) | -91.0196 (-95.7088 to -80.4584) |
| Russian Federation | 0.0052 (0.0035 to 0.0082) | 0.0014 (0.0008 to 0.0022) | -73.8944 (-85.1785 to -61.8033) | 0.8349 (0.5855 to 1.2261) | | 0.0360 (0.0208 to 0.0610) | -95.6865 (-97.5943 to -92.7411) |
| Rwanda | 1.0650 (0.7261 to 1.5325) | 0.3603 (0.2456 to 0.5249) | -66.1683 (-76.1578 to -55.5160) | 363.9139 (178.7118 to 601.5297) | | 23.7411 (10.9212 to 48.4976) | -93.4762 (-96.8387 to -85.6282) |
| Saint Kitts and Nevis | 0.0822 (0.0676 to 0.0987) | 0.0635 (0.0519 to 0.0755) | -22.7746 (-33.7799 to -9.0545) | 2.3432 (0.8942 to 3.3120) | | 0.0785 (0.0434 to 0.1279) | -96.6498 (-98.0920 to -93.7799) |
| Saint Lucia | 0.1180 (0.0957 to 0.1469) | 0.0900 (0.0718 to 0.1116) | -23.7679 (-33.6312 to -11.6050) | 2.4495 (1.1937 to 3.4069) | | 0.0703 (0.0487 to 0.1090) | -97.1285 (-98.2814 to -92.5319) |
| Saint Vincent and the Grenadines | 0.0782 (0.0635 to 0.1000) | 0.0733 (0.0600 to 0.0905) | -6.3605 (-14.1526 to 1.5431) | 0.2850 (0.1212 to 0.4447) | | 0.0548 (0.0298 to 0.0854) | -80.7555 (-89.0446 to -62.6589) |
| Samoa | 0.0285 (0.0103 to 0.0909) | 0.0153 (0.0070 to 0.0391) | -46.1581 (-70.9503 to 22.4017) | 0.8765 (0.3132 to 1.9016) | | 0.4004 (0.1437 to 0.9036) | -54.3256 (-81.0156 to 26.6892) |
| San Marino | 0.0035 (0.0012 to 0.0064) | 0.0007 (0.0004 to 0.0014) | -78.5095 (-90.9270 to -40.6232) | 0.1867 (0.0578 to 0.3432) | | 0.0072 (0.0044 to 0.0119) | -96.1654 (-98.3202 to -88.7881) |
| Sao Tome and Principe | 1.7947 (1.3018 to 2.4257) | 0.9481 (0.6883 to 1.2292) | -47.1733 (-59.1918 to -34.3689) | 323.1603 (170.7980 to 563.6389) | | 32.1135 (12.1276 to 71.3169) | -90.0627 (-96.2386 to -74.8643) |
| Saudi Arabia | 0.4285 (0.2977 to 0.5883) | 0.1414 (0.1150 to 0.1753) | -66.9976 (-74.7121 to -56.1754) | 30.8293 (14.4166 to 56.0299) | | 1.4628 (0.7616 to 2.3474) | -95.2553 (-97.9919 to -87.2158) |
| Senegal | 2.7317 (1.8329 to 3.8522) | 1.5210 (1.0763 to 1.9797) | -44.3229 (-60.0273 to -29.4797) | 345.6166 (130.4047 to 601.4720) | | 29.4272 (13.4468 to 57.4502) | -91.4856 (-95.6884 to -79.2228) |
| Serbia | 0.0063 (0.0011 to 0.0139) | 0.0012 (0.0004 to 0.0019) | -81.5423 (-91.3251 to -37.9246) | 0.8039 (0.1055 to 1.7088) | | 0.0774 (0.0113 to 0.1598) | -90.3778 (-95.6943 to -74.2241) |
| Seychelles | 0.7160 (0.5486 to 0.9323) | 0.4333 (0.3677 to 0.5321) | -39.4822 (-49.5486 to -27.8874) | 21.0830 (10.7804 to 38.8681) | | 4.3316 (2.2240 to 8.1751) | -79.4548 (-90.1502 to -46.6152) |
| Sierra Leone | 2.3719 (1.6387 to 3.3884) | 1.2163 (0.8644 to 1.5808) | -48.7207 (-62.2345 to -34.3255) | 602.3154 (310.4386 to 962.1422) | | 78.8510 (45.3285 to 123.3987) | -86.9087 (-93.5352 to -70.0356) |
| Singapore | 0.0010 (0.0003 to 0.0064) | 0.0006 (0.0002 to 0.0014) | -40.9278 (-83.1999 to 11.7625) | 0.0072 (0.0050 to 0.0109) | | 0.0033 (0.0003 to 0.0073) | -53.5334 (-96.7705 to -10.6328) |
| Slovakia | 0.0008 (0.0003 to 0.0016) | 0.0006 (0.0002 to 0.0012) | -21.5056 (-48.2140 to -2.3225) | 0.0190 (0.0094 to 0.0365) | | 0.0063 (0.0016 to 0.0142) | -66.7460 (-89.3193 to -19.0810) |
| Slovenia | 0.0041 (0.0020 to 0.0066) | 0.0017 (0.0006 to 0.0027) | -59.2439 (-75.6964 to -37.2558) | 0.3869 (0.1481 to 0.5990) | | 0.1061 (0.0112 to 0.1978) | -72.5817 (-93.8738 to -45.8763) |
| Solomon Islands | 0.0619 (0.0216 to 0.1602) | 0.0258 (0.0092 to 0.0584) | -58.2796 (-77.2668 to -1.4514) | 2.6257 (0.3583 to 6.0531) | | 0.9382 (0.1113 to 2.6654) | -64.2698 (-90.8875 to 6.4416) |
| Somalia | 2.8195 (1.2191 to 6.0209) | 1.8020 (1.0937 to 3.4590) | -36.0870 (-65.6336 to 12.9264) | 1,222.5517 (176.1621 to 2,617.8384) | | 448.7140 (137.6623 to 978.1156) | -63.2969 (-83.0015 to 34.6950) |
| South Africa | 0.2193 (0.1630 to 0.2924) | 0.1174 (0.0980 to 0.1437) | -46.4752 (-54.6063 to -34.8612) | 12.7305 (6.7446 to 20.1777) | | 3.6204 (2.2808 to 5.6848) | -71.5615 (-85.0714 to -41.7246) |
| South Sudan | 1.8204 (1.2077 to 2.8739) | 1.2504 (0.8804 to 1.6335) | -31.3106 (-53.9394 to -3.2006) | 444.5024 (224.2825 to 816.2794) | | 139.2462 (70.6575 to 262.6689) | -68.6737 (-85.8729 to -12.6273) |
| Spain | 0.0282 (0.0199 to 0.0396) | 0.0023 (0.0013 to 0.0047) | -91.6804 (-95.7092 to -83.5809) | 1.6266 (1.1062 to 2.1520) | | 0.0151 (0.0091 to 0.0284) | -99.0693 (-99.4768 to -97.8328) |
| Sri Lanka | 0.7570 (0.5699 to 1.0335) | 0.3139 (0.2784 to 0.3705) | -58.5362 (-68.7455 to -46.7680) | 39.5931 (17.8261 to 68.3671) | | 2.5056 (1.4302 to 4.4684) | -93.6717 (-96.9140 to -86.0661) |
| Sudan | 0.5244 (0.3107 to 0.8818) | 0.1244 (0.0900 to 0.1698) | -76.2763 (-85.7192 to -64.3099) | 180.1818 (53.6032 to 394.4370) | | 3.9769 (1.3885 to 7.9848) | -97.7928 (-99.1256 to -92.6156) |
| Suriname | 0.3798 (0.2537 to 0.4994) | 0.0994 (0.0739 to 0.1443) | -73.8331 (-80.3271 to -63.3117) | 18.7964 (8.9564 to 27.9517) | | 1.2856 (0.6988 to 2.5338) | -93.1605 (-96.8530 to -84.3331) |
| Sweden | 0.0010 (0.0005 to 0.0020) | 0.0006 (0.0002 to 0.0011) | -43.1136 (-62.7537 to -26.7751) | 0.0250 (0.0180 to 0.0355) | | 0.0020 (0.0010 to 0.0033) | -92.0466 (-95.7473 to -86.3500) |
| Switzerland | 0.0008 (0.0003 to 0.0015) | 0.0008 (0.0004 to 0.0015) | 3.5515 (-23.4826 to 36.3179) | 0.0041 (0.0025 to 0.0082) | | 0.0117 (0.0024 to 0.0194) | 184.5820 (-67.9377 to 478.9868) |
| Syrian Arab Republic | 0.3539 (0.2500 to 0.4784) | 0.1424 (0.1140 to 0.1815) | -59.7687 (-68.7909 to -49.5412) | 41.6061 (17.2919 to 91.8657) | | 1.7678 (0.6376 to 3.8642) | -95.7510 (-98.5279 to -86.9081) |
| Taiwan | 0.3421 (0.2979 to 0.3920) | 0.2522 (0.2181 to 0.2846) | -26.2826 (-33.2450 to -18.7878) | 3.3301 (2.0587 to 4.3579) | | 0.0894 (0.0641 to 0.1198) | -97.3143 (-98.2757 to -95.0693) |
| Tajikistan | 0.0015 (0.0006 to 0.0044) | 0.0007 (0.0003 to 0.0017) | -54.4221 (-72.0509 to -24.9910) | 0.1833 (0.0651 to 0.3724) | | 0.0049 (0.0028 to 0.0108) | -97.3469 (-98.6271 to -92.3075) |
| Thailand | 1.0068 (0.7530 to 1.4231) | 0.4120 (0.3516 to 0.5118) | -59.0786 (-69.3323 to -46.9687) | 38.5287 (20.8308 to 64.5767) | | 5.6800 (2.0209 to 10.1558) | -85.2576 (-93.4833 to -68.8445) |
| Timor-Leste | 6.8034 (2.9950 to 13.4824) | 1.5094 (1.0639 to 1.9927) | -77.8134 (-87.7953 to -55.9569) | 1,801.1788 (594.7448 to 3,186.0712) | | 52.0527 (16.3319 to 115.6387) | -97.1101 (-98.8339 to -93.1087) |
| Togo | 1.2692 (0.8973 to 1.7219) | 0.6924 (0.4738 to 0.9628) | -45.4458 (-56.1696 to -35.2091) | 212.4318 (100.6873 to 368.6733) | | 28.1075 (13.0474 to 50.2708) | -86.7687 (-94.0400 to -69.0110) |
| Tokelau | 0.0196 (0.0083 to 0.0588) | 0.0084 (0.0044 to 0.0162) | -57.0345 (-81.0667 to -12.1094) | 0.5299 (0.1806 to 1.2319) | | 0.0923 (0.0344 to 0.1994) | -82.5891 (-93.2096 to -39.6693) |
| Tonga | 0.0078 (0.0030 to 0.0219) | 0.0050 (0.0023 to 0.0112) | -35.4030 (-62.5098 to 36.4478) | 0.2545 (0.0717 to 0.5912) | | 0.1300 (0.0373 to 0.3006) | -48.9034 (-77.1169 to 47.7407) |
| Trinidad and Tobago | 0.1704 (0.1110 to 0.2239) | 0.0940 (0.0707 to 0.1261) | -44.8610 (-60.5592 to -12.8652) | 7.9254 (1.0499 to 12.7534) | | 0.0841 (0.0474 to 0.1704) | -98.9386 (-99.5225 to -93.7444) |
| Tunisia | 0.5537 (0.3564 to 0.8207) | 0.1668 (0.1233 to 0.2217) | -69.8782 (-79.4470 to -59.5333) | 92.0012 (30.3225 to 210.8078) | | 2.4215 (1.1936 to 4.3260) | -97.3680 (-98.9709 to -89.8601) |
| Turkmenistan | 0.0087 (0.0061 to 0.0128) | 0.0068 (0.0045 to 0.0098) | -21.9763 (-46.7654 to -1.8329) | 0.9368 (0.5905 to 1.2667) | | 0.4893 (0.1609 to 0.6868) | -47.7715 (-74.7382 to -15.9844) |
| Tuvalu | 0.0372 (0.0109 to 0.1283) | 0.0159 (0.0068 to 0.0476) | -57.3765 (-78.3314 to 4.6372) | 1.7964 (0.4523 to 3.5872) | | 0.5616 (0.1799 to 1.2866) | -68.7368 (-88.1175 to -5.4061) |
| Türkiye | 0.1890 (0.1250 to 0.2708) | 0.0885 (0.0686 to 0.1148) | -53.1904 (-68.8222 to -37.4260) | 57.2297 (17.3775 to 140.9624) | | 0.6304 (0.3568 to 1.1686) | -98.8985 (-99.6134 to -96.4275) |
| Uganda | 1.1559 (0.7141 to 1.6968) | 0.3409 (0.2320 to 0.4812) | -70.5124 (-80.4095 to -57.6744) | 345.1582 (119.7569 to 622.7417) | | 22.1601 (11.1611 to 43.3945) | -93.5797 (-97.5974 to -82.1782) |
| Ukraine | 0.0079 (0.0054 to 0.0109) | 0.0009 (0.0004 to 0.0022) | -88.0802 (-94.1488 to -72.5872) | 1.5903 (1.2354 to 2.0346) | | 0.0678 (0.0473 to 0.1498) | -95.7337 (-97.3907 to -90.2475) |
| United Arab Emirates | 0.6422 (0.2600 to 1.4030) | 0.1671 (0.1333 to 0.2089) | -73.9809 (-87.4735 to -43.8349) | 89.1322 (28.4511 to 221.5501) | | 1.1865 (0.4416 to 2.1686) | -98.6688 (-99.5504 to -95.7003) |
| United Kingdom | 0.0018 (0.0010 to 0.0040) | 0.0010 (0.0005 to 0.0018) | -41.9025 (-69.1264 to -23.7104) | 0.0638 (0.0466 to 0.1142) | | 0.0149 (0.0074 to 0.0229) | -76.7094 (-87.3547 to -62.3586) |
| United Republic of Tanzania | 0.7578 (0.5186 to 1.0812) | 0.3260 (0.2296 to 0.4499) | -56.9755 (-67.8289 to -46.1411) | 232.2578 (98.6259 to 409.6447) | | 21.8689 (11.7589 to 37.8719) | -90.5842 (-96.2295 to -77.9596) |
| United States Virgin Islands | 0.0972 (0.0783 to 0.1254) | 0.1261 (0.1031 to 0.1526) | 29.7256 (13.7131 to 45.7688) | 0.4188 (0.2688 to 0.6416) | | 1.7708 (0.8774 to 2.9252) | 322.8313 (82.3207 to 656.1089) |
| United States of America | 0.0028 (0.0017 to 0.0046) | 0.0016 (0.0009 to 0.0027) | -42.0924 (-58.8409 to -26.6733) | 0.1891 (0.1346 to 0.2524) | | 0.0305 (0.0202 to 0.0458) | -83.8494 (-89.1976 to -74.0028) |
| Uruguay | 0.0135 (0.0028 to 0.0252) | 0.0008 (0.0003 to 0.0020) | -94.3985 (-97.5226 to -73.6859) | 1.1614 (0.2080 to 1.8933) | | 0.0078 (0.0043 to 0.0153) | -99.3291 (-99.6554 to -96.3873) |
| Uzbekistan | 0.0034 (0.0013 to 0.0061) | 0.0008 (0.0004 to 0.0014) | -75.9361 (-87.1356 to -51.2848) | 0.7461 (0.1926 to 1.1991) | | 0.0347 (0.0089 to 0.0540) | -95.3536 (-97.2583 to -91.5201) |
| Vanuatu | 0.0649 (0.0228 to 0.1850) | 0.0262 (0.0102 to 0.0590) | -59.6366 (-76.0922 to -7.4779) | 2.6340 (0.5549 to 6.1578) | | 0.8813 (0.1582 to 2.2731) | -66.5418 (-89.9887 to -2.9141) |
| Venezuela (Bolivarian Republic of) | 0.0621 (0.0465 to 0.0818) | 0.0395 (0.0279 to 0.0670) | -36.3858 (-55.7158 to 12.8711) | 9.2623 (6.8017 to 12.4027) | | 2.0452 (0.8454 to 5.7096) | -77.9190 (-90.8268 to -36.6376) |
| Viet Nam | 1.2393 (0.9128 to 1.7071) | 0.3963 (0.3166 to 0.5191) | -68.0177 (-75.1844 to -58.5497) | 120.6485 (51.7344 to 215.7120) | | 4.4690 (2.1310 to 8.6784) | -96.2959 (-98.5005 to -90.2213) |
| Yemen | 1.1583 (0.5724 to 2.4121) | 0.3436 (0.2327 to 0.4782) | -70.3371 (-83.3757 to -44.2083) | 442.6164 (66.3343 to 1,068.7389) | | 12.6880 (3.2823 to 28.3202) | -97.1334 (-99.1360 to -85.2162) |
| Zambia | 1.1211 (0.7908 to 1.6035) | 0.4413 (0.3013 to 0.6244) | -60.6378 (-72.5467 to -44.7787) | 278.7605 (146.2773 to 446.3953) | | 35.3747 (15.4224 to 76.7973) | -87.3100 (-95.2175 to -62.7929) |
| Zimbabwe | 0.1658 (0.1195 to 0.2319) | 0.0891 (0.0701 to 0.1200) | -46.3022 (-61.2993 to -23.7054) | 14.3349 (6.9217 to 23.5206) | | 3.4348 (1.1602 to 8.8021) | -76.0387 (-93.6630 to -35.1649) |

DALY= disability-adjusted life year; UI= uncertainty interval.

**Table S3.** Age-standardized incidence and mortality rate (95% UI) per 100,000 population in 1990 and 2023.

| Country | Age-standardized incidence rate per 100,000 (95% UI) | | | Age-standardized mortality rate per 100,000 (95% UI) | | | |
| --- | --- | --- | --- | --- | --- | --- | --- |
|  | 1990 | 2023 | Total percentage change (%) | 1990 | 2023 | Total percentage change (%) |  |
| Global |  |  |  |  |  |  |  |
| Afghanistan | 18.0956 (5.6427 to 41.6948) | 0.7793 (0.3018 to 1.7760) | -95.6933 (-98.7787 to -84.4576) | 11.1624 (3.5339 to 22.9587) | 0.4241 (0.1766 to 0.8244) | -96.2008 (-98.8463 to -86.6427) |  |
| Albania | 0.0374 (0.0185 to 0.0644) | 0.0016 (0.0004 to 0.0117) | -95.7994 (-99.1106 to -81.8959) | 0.0184 (0.0088 to 0.0296) | 0.0004 (0.0002 to 0.0009) | -97.9657 (-99.3022 to -94.7548) |  |
| Algeria | 0.6420 (0.1972 to 1.3811) | 0.0541 (0.0221 to 0.1559) | -91.5753 (-97.2276 to -68.0783) | 0.2684 (0.0820 to 0.5363) | 0.0203 (0.0089 to 0.0444) | -92.4486 (-97.0602 to -73.2735) |  |
| American Samoa | 0.3123 (0.1093 to 0.9422) | 0.0510 (0.0155 to 0.1583) | -83.6548 (-93.2250 to -61.2265) | 0.0629 (0.0269 to 0.1331) | 0.0103 (0.0035 to 0.0200) | -83.6596 (-92.8421 to -59.6877) |  |
| Andorra | 0.0193 (0.0045 to 0.1050) | 0.0017 (0.0005 to 0.0124) | -91.4029 (-96.4904 to -78.4618) | 0.0018 (0.0008 to 0.0037) | 0.0002 (0.0001 to 0.0003) | -91.4612 (-95.3823 to -80.0320) |  |
| Angola | 11.2446 (3.6591 to 23.2435) | 1.4547 (0.4246 to 6.3156) | -87.0632 (-95.3445 to -66.1242) | 7.6047 (2.5402 to 15.2035) | 0.8295 (0.2905 to 2.3780) | -89.0928 (-95.2007 to -72.0333) |  |
| Antigua and Barbuda | 0.2258 (0.0708 to 0.3898) | 0.0210 (0.0067 to 0.0468) | -90.6849 (-95.6248 to -78.9478) | 0.0428 (0.0145 to 0.0674) | 0.0034 (0.0011 to 0.0054) | -92.0386 (-95.8349 to -85.1437) |  |
| Argentina | 0.8056 (0.5428 to 1.3587) | 0.0283 (0.0126 to 0.1219) | -96.4812 (-98.3561 to -86.2973) | 0.1462 (0.1047 to 0.2211) | 0.0039 (0.0022 to 0.0089) | -97.3440 (-98.5102 to -94.0732) |  |
| Armenia | 0.0418 (0.0241 to 0.0659) | 0.0014 (0.0007 to 0.0075) | -96.6303 (-98.5244 to -87.7768) | 0.0168 (0.0104 to 0.0254) | 0.0004 (0.0002 to 0.0006) | -97.9139 (-98.7819 to -95.2281) |  |
| Australia | 0.0600 (0.0284 to 0.1249) | 0.0049 (0.0022 to 0.0151) | -91.8358 (-95.8710 to -83.0529) | 0.0043 (0.0024 to 0.0064) | 0.0003 (0.0002 to 0.0005) | -92.8129 (-95.8683 to -87.0227) |  |
| Austria | 0.0111 (0.0020 to 0.0239) | 0.0096 (0.0003 to 0.0185) | -13.7387 (-86.8945 to 98.8155) | 0.0028 (0.0005 to 0.0047) | 0.0022 (0.0001 to 0.0039) | -19.5514 (-88.2189 to 77.7428) |  |
| Azerbaijan | 0.0440 (0.0107 to 0.2190) | 0.0044 (0.0009 to 0.0261) | -90.0373 (-96.9334 to -80.3718) | 0.0118 (0.0044 to 0.0286) | 0.0008 (0.0004 to 0.0021) | -92.7963 (-96.8447 to -86.0307) |  |
| Bahamas | 0.0229 (0.0059 to 0.0860) | 0.0066 (0.0009 to 0.0233) | -71.0620 (-88.3228 to -31.9026) | 0.0039 (0.0013 to 0.0076) | 0.0009 (0.0002 to 0.0018) | -76.6624 (-89.7105 to -43.5408) |  |
| Bahrain | 0.0660 (0.0290 to 0.2302) | 0.0071 (0.0016 to 0.0261) | -89.3160 (-96.6870 to -70.9755) | 0.0248 (0.0130 to 0.0442) | 0.0022 (0.0007 to 0.0048) | -91.2554 (-96.7824 to -79.7013) |  |
| Bangladesh | 25.6610 (17.3904 to 35.7247) | 0.7696 (0.3189 to 1.4692) | -97.0011 (-98.6157 to -94.6598) | 15.4974 (10.8941 to 20.4736) | 0.3518 (0.1723 to 0.6017) | -97.7301 (-98.7781 to -95.9855) |  |
| Barbados | 2.3909 (0.7275 to 3.5129) | 0.1185 (0.0585 to 0.2318) | -95.0451 (-97.5720 to -84.2975) | 0.5211 (0.1578 to 0.7528) | 0.0219 (0.0117 to 0.0389) | -95.7997 (-97.9169 to -88.3098) |  |
| Belarus | 0.0296 (0.0050 to 0.0489) | 0.0003 (0.0001 to 0.0021) | -99.0555 (-99.7589 to -93.0448) | 0.0132 (0.0023 to 0.0221) | 0.0001 (0.0000 to 0.0001) | -99.5853 (-99.8018 to -97.5566) |  |
| Belgium | 0.0395 (0.0149 to 0.0648) | 0.0098 (0.0014 to 0.0202) | -75.1923 (-92.4247 to -38.6283) | 0.0079 (0.0025 to 0.0129) | 0.0015 (0.0002 to 0.0031) | -80.7749 (-93.4883 to -52.1669) |  |
| Belize | 0.8497 (0.2496 to 1.4750) | 0.0100 (0.0027 to 0.0679) | -98.8192 (-99.6806 to -89.7222) | 0.1425 (0.0476 to 0.2341) | 0.0008 (0.0004 to 0.0015) | -99.4111 (-99.6908 to -98.3541) |  |
| Benin | 12.4881 (5.3589 to 23.6821) | 1.3280 (0.6014 to 2.8321) | -89.3658 (-95.0342 to -73.0611) | 6.7016 (2.7096 to 12.2144) | 0.6367 (0.3077 to 1.2442) | -90.4999 (-95.6437 to -78.0579) |  |
| Bermuda | 0.0306 (0.0102 to 0.0918) | 0.0054 (0.0007 to 0.0110) | -82.3804 (-93.1250 to -66.9958) | 0.0053 (0.0015 to 0.0087) | 0.0008 (0.0001 to 0.0013) | -84.5461 (-91.9834 to -72.3264) |  |
| Bhutan | 14.2268 (4.6331 to 30.0532) | 0.2765 (0.0950 to 0.6784) | -98.0564 (-99.3158 to -94.6590) | 7.1409 (2.2559 to 13.6053) | 0.1129 (0.0431 to 0.2215) | -98.4186 (-99.4074 to -95.9273) |  |
| Bolivia (Plurinational State of) | 7.0015 (3.4546 to 12.1232) | 0.2612 (0.0865 to 0.5604) | -96.2692 (-98.8297 to -91.8253) | 2.8258 (1.4041 to 4.7198) | 0.0897 (0.0327 to 0.1850) | -96.8247 (-98.9444 to -93.2226) |  |
| Bosnia and Herzegovina | 0.0356 (0.0101 to 0.0692) | 0.0164 (0.0013 to 0.0355) | -54.0828 (-91.6875 to 29.1289) | 0.0142 (0.0044 to 0.0263) | 0.0063 (0.0005 to 0.0133) | -55.3912 (-91.4641 to 29.2752) |  |
| Botswana | 1.0633 (0.3583 to 2.3032) | 0.2307 (0.0670 to 0.7125) | -78.3004 (-94.9498 to 3.2117) | 0.3910 (0.1314 to 0.8344) | 0.0743 (0.0254 to 0.1732) | -80.9933 (-94.8626 to -7.0578) |  |
| Brazil | 2.0634 (1.6198 to 2.8335) | 0.0813 (0.0505 to 0.1836) | -96.0593 (-97.5460 to -90.5640) | 0.8254 (0.6702 to 1.0471) | 0.0296 (0.0209 to 0.0479) | -96.4140 (-97.5496 to -94.7375) |  |
| Brunei Darussalam | 0.0219 (0.0024 to 0.1985) | 0.0091 (0.0004 to 0.0428) | -58.6674 (-89.7136 to 180.7579) | 0.0011 (0.0004 to 0.0032) | 0.0011 (0.0001 to 0.0021) | -4.8437 (-90.1884 to 195.6916) |  |
| Bulgaria | 0.0622 (0.0363 to 0.0899) | 0.0026 (0.0014 to 0.0101) | -95.7756 (-98.0404 to -81.7669) | 0.0255 (0.0155 to 0.0354) | 0.0009 (0.0005 to 0.0020) | -96.3807 (-98.1251 to -91.9139) |  |
| Burkina Faso | 7.8769 (4.4186 to 12.8234) | 0.7266 (0.3369 to 1.3037) | -90.7756 (-96.2141 to -77.6235) | 4.2133 (2.3326 to 6.5403) | 0.3733 (0.1771 to 0.6335) | -91.1412 (-96.1944 to -79.1526) |  |
| Burundi | 11.1906 (4.5040 to 21.1705) | 2.2657 (0.6357 to 6.4992) | -79.7533 (-91.2986 to -48.2637) | 6.7362 (2.6377 to 12.0967) | 1.3153 (0.3887 to 3.5428) | -80.4738 (-91.5499 to -54.8915) |  |
| Cabo Verde | 4.2247 (1.4696 to 10.5636) | 0.1879 (0.0749 to 0.4342) | -95.5523 (-99.0140 to -81.7557) | 1.8609 (0.6829 to 4.2815) | 0.0865 (0.0366 to 0.1866) | -95.3492 (-98.7735 to -81.7019) |  |
| Cambodia | 100.1420 (29.7444 to 201.6334) | 3.5809 (1.0148 to 9.2367) | -96.4242 (-98.5295 to -92.2235) | 29.6650 (9.1047 to 49.1145) | 0.8157 (0.2547 to 1.8091) | -97.2503 (-98.8992 to -94.2448) |  |
| Cameroon | 7.0147 (3.3255 to 13.2239) | 0.9318 (0.3545 to 2.0607) | -86.7158 (-94.5283 to -61.1703) | 4.0682 (2.0612 to 6.8676) | 0.5221 (0.2187 to 1.0642) | -87.1654 (-94.6497 to -65.2942) |  |
| Canada | 0.0016 (0.0005 to 0.0117) | 0.0011 (0.0001 to 0.0030) | -33.6502 (-82.9542 to 39.7702) | 0.0004 (0.0002 to 0.0006) | 0.0003 (0.0000 to 0.0005) | -12.8694 (-84.6691 to 56.0727) |  |
| Central African Republic | 7.9769 (2.1484 to 16.4998) | 4.4178 (1.1037 to 10.1442) | -44.6172 (-73.6778 to 36.2981) | 5.3185 (1.4743 to 10.3080) | 2.9773 (0.7617 to 6.4281) | -44.0204 (-73.1387 to 37.1610) |  |
| Chad | 27.1045 (9.9855 to 57.2413) | 6.6191 (2.5053 to 16.9291) | -75.5792 (-89.4571 to -38.2596) | 14.5384 (5.3793 to 29.0703) | 3.4299 (1.3662 to 7.3312) | -76.4076 (-89.4726 to -40.6649) |  |
| Chile | 0.2850 (0.1770 to 0.4756) | 0.0074 (0.0035 to 0.0305) | -97.3914 (-98.6888 to -90.8966) | 0.0527 (0.0360 to 0.0726) | 0.0010 (0.0006 to 0.0018) | -98.1901 (-98.9025 to -96.3235) |  |
| China | 7.5228 (4.8184 to 11.3650) | 0.0859 (0.0347 to 0.1886) | -98.8586 (-99.5208 to -96.8984) | 1.7916 (1.1769 to 2.5558) | 0.0162 (0.0076 to 0.0319) | -99.0945 (-99.5933 to -98.0041) |  |
| Colombia | 1.0231 (0.8019 to 1.2811) | 0.0453 (0.0275 to 0.0829) | -95.5701 (-97.3545 to -91.4552) | 0.5630 (0.4587 to 0.6805) | 0.0209 (0.0136 to 0.0320) | -96.2944 (-97.5565 to -93.9498) |  |
| Comoros | 5.1385 (2.4094 to 9.5998) | 0.5744 (0.2648 to 1.1595) | -88.8222 (-95.2687 to -68.0895) | 3.0540 (1.4788 to 5.5693) | 0.3032 (0.1514 to 0.5199) | -90.0737 (-95.4787 to -73.3288) |  |
| Congo | 1.7765 (0.6299 to 3.6107) | 0.5158 (0.1496 to 1.7437) | -70.9664 (-91.2313 to -1.5273) | 1.1775 (0.3923 to 2.4143) | 0.3365 (0.1190 to 0.9435) | -71.4204 (-90.0820 to -16.1371) |  |
| Cook Islands | 0.1538 (0.0101 to 0.5097) | 0.0237 (0.0016 to 0.1075) | -84.5876 (-94.7487 to -21.1325) | 0.0246 (0.0016 to 0.0714) | 0.0032 (0.0002 to 0.0097) | -87.1117 (-94.6851 to -48.5826) |  |
| Costa Rica | 0.0308 (0.0051 to 0.0537) | 0.0015 (0.0002 to 0.0057) | -95.2972 (-98.2157 to -84.5015) | 0.0165 (0.0029 to 0.0244) | 0.0005 (0.0001 to 0.0009) | -96.9495 (-98.3969 to -93.8160) |  |
| Croatia | 0.3080 (0.0601 to 0.5075) | 0.0049 (0.0023 to 0.0103) | -98.4011 (-99.3321 to -92.8597) | 0.0900 (0.0157 to 0.1390) | 0.0012 (0.0006 to 0.0023) | -98.6118 (-99.3912 to -94.5807) |  |
| Cuba | 0.0741 (0.0128 to 0.1249) | 0.0019 (0.0003 to 0.0113) | -97.4090 (-99.6335 to -78.3693) | 0.0129 (0.0023 to 0.0209) | 0.0001 (0.0001 to 0.0002) | -99.1513 (-99.5928 to -95.6379) |  |
| Cyprus | 0.0384 (0.0055 to 0.1023) | 0.0083 (0.0005 to 0.0189) | -78.4261 (-94.2288 to -14.6565) | 0.0096 (0.0015 to 0.0262) | 0.0020 (0.0001 to 0.0042) | -78.9445 (-94.2687 to -12.2980) |  |
| Czechia | 0.0084 (0.0041 to 0.0178) | 0.0013 (0.0003 to 0.0027) | -84.3247 (-95.0615 to -57.8642) | 0.0034 (0.0017 to 0.0058) | 0.0005 (0.0001 to 0.0008) | -86.2883 (-95.1323 to -64.6970) |  |
| Côte d'Ivoire | 9.5975 (4.7037 to 16.4467) | 1.5553 (0.6610 to 3.2424) | -83.7949 (-92.5637 to -55.1116) | 4.2662 (2.2111 to 7.0504) | 0.6352 (0.3102 to 1.1460) | -85.1097 (-92.8947 to -61.9310) |  |
| Democratic People's Republic of Korea | 3.4089 (1.4841 to 7.2324) | 0.7505 (0.1967 to 2.3772) | -77.9845 (-92.6455 to -12.8131) | 0.5120 (0.2345 to 0.9400) | 0.0963 (0.0335 to 0.2490) | -81.1972 (-92.8678 to -49.6080) |  |
| Democratic Republic of the Congo | 5.9047 (1.8720 to 13.9198) | 1.6354 (0.3550 to 4.5270) | -72.3039 (-88.9153 to -30.1333) | 3.7376 (1.2056 to 7.7539) | 0.9882 (0.2376 to 2.3254) | -73.5602 (-88.5534 to -35.9308) |  |
| Denmark | 0.0018 (0.0005 to 0.0154) | 0.0064 (0.0009 to 0.0111) | 252.0420 (-71.1146 to 1,400.7018) | 0.0002 (0.0001 to 0.0005) | 0.0014 (0.0002 to 0.0021) | 478.9788 (-63.3967 to 1,160.2007) |  |
| Djibouti | 4.2566 (1.7542 to 10.8070) | 1.3086 (0.3962 to 4.1912) | -69.2585 (-88.9199 to -5.0981) | 2.2966 (1.1144 to 4.5359) | 0.6225 (0.2305 to 1.6489) | -72.8925 (-89.0559 to -25.2247) |  |
| Dominica | 0.2941 (0.1570 to 0.6342) | 0.0884 (0.0264 to 0.3509) | -69.9327 (-88.6178 to -41.8968) | 0.0564 (0.0304 to 0.0823) | 0.0136 (0.0060 to 0.0250) | -75.8513 (-87.7070 to -53.6893) |  |
| Dominican Republic | 4.6666 (2.6013 to 12.1461) | 1.0730 (0.4896 to 2.7663) | -77.0073 (-89.7982 to -55.0820) | 0.8335 (0.5020 to 1.5064) | 0.1829 (0.0972 to 0.3525) | -78.0534 (-88.7348 to -58.9047) |  |
| Ecuador | 2.5287 (1.3428 to 3.4102) | 0.1091 (0.0627 to 0.2210) | -95.6863 (-97.5893 to -89.7634) | 0.9735 (0.5421 to 1.3024) | 0.0331 (0.0207 to 0.0552) | -96.5987 (-97.9993 to -93.4958) |  |
| Egypt | 3.3226 (2.0573 to 6.5283) | 0.2640 (0.1347 to 0.4507) | -92.0535 (-96.4382 to -82.8641) | 1.5483 (1.0098 to 2.4303) | 0.1074 (0.0543 to 0.1790) | -93.0618 (-96.7114 to -86.6544) |  |
| El Salvador | 1.7312 (0.5589 to 3.1392) | 0.0214 (0.0042 to 0.1076) | -98.7659 (-99.8172 to -92.4092) | 1.0220 (0.3452 to 1.8437) | 0.0075 (0.0024 to 0.0200) | -99.2647 (-99.8281 to -96.7430) |  |
| Equatorial Guinea | 15.3202 (4.8259 to 38.0388) | 0.2575 (0.0486 to 1.6547) | -98.3194 (-99.7048 to -94.7725) | 10.3965 (3.4435 to 22.4233) | 0.1246 (0.0370 to 0.3777) | -98.8012 (-99.6739 to -95.9437) |  |
| Eritrea | 36.5794 (17.1665 to 70.1292) | 1.5300 (0.7583 to 3.2321) | -95.8173 (-98.2423 to -89.2313) | 22.6334 (11.0541 to 40.7639) | 0.8769 (0.4586 to 1.7632) | -96.1257 (-98.2551 to -90.9711) |  |
| Estonia | 0.0038 (0.0018 to 0.0186) | 0.0023 (0.0001 to 0.0043) | -41.1012 (-95.5010 to 62.8791) | 0.0012 (0.0008 to 0.0017) | 0.0009 (0.0000 to 0.0017) | -22.4814 (-95.2789 to 65.3660) |  |
| Eswatini | 2.1169 (0.7060 to 4.6261) | 0.4967 (0.1967 to 1.0921) | -76.5384 (-93.3944 to -6.8592) | 0.7417 (0.2533 to 1.5684) | 0.1581 (0.0741 to 0.2943) | -78.6871 (-92.8181 to -20.3941) |  |
| Ethiopia | 58.6227 (26.6617 to 97.9463) | 2.3865 (1.0145 to 4.9873) | -95.9291 (-98.4698 to -88.0512) | 30.6136 (13.1158 to 48.6291) | 1.2563 (0.5743 to 2.4589) | -95.8961 (-98.2384 to -88.6355) |  |
| Fiji | 0.0231 (0.0022 to 0.1768) | 0.0124 (0.0011 to 0.1105) | -46.2729 (-88.5088 to 80.4223) | 0.0016 (0.0005 to 0.0035) | 0.0006 (0.0003 to 0.0013) | -63.0197 (-88.7244 to 9.5176) |  |
| Finland | 0.0030 (0.0011 to 0.0204) | 0.0010 (0.0001 to 0.0038) | -67.4334 (-92.0034 to -24.6249) | 0.0005 (0.0003 to 0.0009) | 0.0002 (0.0000 to 0.0003) | -65.8958 (-91.2584 to -32.3676) |  |
| France | 0.2788 (0.0759 to 0.3878) | 0.0066 (0.0032 to 0.0128) | -97.6180 (-98.8059 to -92.9163) | 0.0546 (0.0146 to 0.0717) | 0.0012 (0.0006 to 0.0021) | -97.7799 (-98.8285 to -93.8837) |  |
| Gabon | 1.2842 (0.4078 to 3.3815) | 0.2381 (0.0576 to 1.0464) | -81.4595 (-95.5414 to -38.6101) | 0.8724 (0.2768 to 2.2254) | 0.1338 (0.0469 to 0.3756) | -84.6666 (-94.6101 to -59.5386) |  |
| Gambia | 3.2285 (1.9811 to 5.3234) | 0.7277 (0.2521 to 1.7737) | -77.4598 (-92.1903 to -44.5114) | 1.6327 (1.0478 to 2.4146) | 0.3388 (0.1367 to 0.7716) | -79.2502 (-92.3145 to -52.0856) |  |
| Georgia | 0.0561 (0.0183 to 0.0928) | 0.0151 (0.0028 to 0.0313) | -73.0191 (-86.8774 to -40.1240) | 0.0243 (0.0076 to 0.0387) | 0.0066 (0.0011 to 0.0133) | -72.7503 (-87.7342 to -37.2041) |  |
| Germany | 0.0251 (0.0083 to 0.0447) | 0.0007 (0.0002 to 0.0037) | -97.2678 (-99.1028 to -87.9729) | 0.0063 (0.0021 to 0.0099) | 0.0001 (0.0001 to 0.0002) | -98.3622 (-99.1641 to -91.3829) |  |
| Ghana | 7.8626 (4.2243 to 15.0745) | 0.6522 (0.2918 to 1.3076) | -91.7047 (-96.3164 to -82.2009) | 4.5899 (2.5885 to 7.9212) | 0.3557 (0.1634 to 0.6671) | -92.2502 (-96.3843 to -82.6312) |  |
| Greece | 0.0422 (0.0248 to 0.0997) | 0.0192 (0.0064 to 0.0351) | -54.4258 (-89.6406 to 0.9042) | 0.0089 (0.0058 to 0.0147) | 0.0043 (0.0015 to 0.0072) | -51.7486 (-88.0276 to -2.0941) |  |
| Greenland | 0.0372 (0.0109 to 0.2061) | 0.0145 (0.0029 to 0.0362) | -61.0746 (-85.8913 to 46.1820) | 0.0080 (0.0036 to 0.0188) | 0.0043 (0.0009 to 0.0092) | -46.0698 (-83.1860 to 45.1399) |  |
| Grenada | 0.7145 (0.4720 to 1.2469) | 0.0235 (0.0104 to 0.1123) | -96.7123 (-98.6897 to -86.9401) | 0.1387 (0.0974 to 0.1893) | 0.0032 (0.0019 to 0.0055) | -97.7124 (-98.7694 to -96.0075) |  |
| Guam | 0.0287 (0.0058 to 0.1305) | 0.0170 (0.0069 to 0.0309) | -40.8253 (-89.0304 to 121.7529) | 0.0041 (0.0012 to 0.0129) | 0.0026 (0.0013 to 0.0044) | -36.0136 (-88.7575 to 103.4858) |  |
| Guatemala | 2.7099 (1.8110 to 3.8866) | 0.0772 (0.0380 to 0.1718) | -97.1523 (-98.5261 to -92.2331) | 1.6692 (1.1288 to 2.3522) | 0.0387 (0.0228 to 0.0707) | -97.6809 (-98.6756 to -95.5871) |  |
| Guinea | 11.4471 (5.9921 to 23.3404) | 1.4549 (0.5413 to 3.9132) | -87.2899 (-95.2581 to -67.0059) | 7.4544 (4.0426 to 12.5929) | 0.8502 (0.3578 to 1.8641) | -88.5952 (-95.3257 to -71.8678) |  |
| Guinea-Bissau | 19.3783 (8.6806 to 35.4522) | 2.9370 (1.3969 to 6.2615) | -84.8441 (-92.9437 to -60.6015) | 11.4863 (4.7928 to 19.3437) | 1.5271 (0.7423 to 2.8987) | -86.7050 (-93.6526 to -69.6959) |  |
| Guyana | 0.5889 (0.3248 to 0.9154) | 0.0384 (0.0065 to 0.1080) | -93.4761 (-98.4297 to -81.8176) | 0.1351 (0.0770 to 0.1935) | 0.0062 (0.0015 to 0.0165) | -95.3851 (-98.5153 to -86.7780) |  |
| Haiti | 15.2077 (6.8155 to 30.0132) | 1.3100 (0.4401 to 3.1310) | -91.3856 (-97.1842 to -72.5555) | 3.8342 (1.7687 to 6.5665) | 0.2733 (0.0978 to 0.5468) | -92.8714 (-97.3874 to -81.1338) |  |
| Honduras | 0.3771 (0.1349 to 0.6678) | 0.0515 (0.0124 to 0.1458) | -86.3364 (-96.6855 to -61.3025) | 0.2469 (0.0915 to 0.4207) | 0.0318 (0.0082 to 0.0781) | -87.1008 (-96.5159 to -68.6766) |  |
| Hungary | 0.2187 (0.0927 to 0.3274) | 0.0021 (0.0008 to 0.0086) | -99.0252 (-99.6861 to -94.1959) | 0.0945 (0.0405 to 0.1440) | 0.0007 (0.0004 to 0.0016) | -99.2766 (-99.6901 to -96.6891) |  |
| Iceland | 0.0060 (0.0022 to 0.0163) | 0.0150 (0.0005 to 0.0252) | 150.2524 (-81.4303 to 364.2688) | 0.0014 (0.0005 to 0.0024) | 0.0034 (0.0001 to 0.0056) | 145.9117 (-85.4454 to 358.8416) |  |
| India | 19.9609 (15.0904 to 26.6005) | 0.4453 (0.2233 to 0.8021) | -97.7691 (-98.7164 to -95.8952) | 11.0817 (8.8881 to 13.8743) | 0.2001 (0.1083 to 0.3530) | -98.1940 (-98.9742 to -96.7619) |  |
| Indonesia | 39.4947 (17.7255 to 76.6339) | 3.4025 (1.3272 to 7.5043) | -91.3849 (-96.9643 to -75.5149) | 12.0027 (5.8764 to 20.6404) | 0.8563 (0.3467 to 1.6754) | -92.8657 (-97.1561 to -81.1648) |  |
| Iran (Islamic Republic of) | 1.4256 (0.5963 to 2.7584) | 0.0270 (0.0120 to 0.0736) | -98.1095 (-99.3152 to -93.6639) | 0.6090 (0.2549 to 1.0723) | 0.0096 (0.0049 to 0.0184) | -98.4213 (-99.3239 to -95.1006) |  |
| Iraq | 2.2477 (0.8289 to 5.4074) | 0.1488 (0.0632 to 0.3430) | -93.3799 (-97.7779 to -75.2879) | 0.9772 (0.3912 to 1.9529) | 0.0540 (0.0254 to 0.1058) | -94.4719 (-97.9149 to -83.4310) |  |
| Ireland | 0.0101 (0.0046 to 0.0503) | 0.0030 (0.0003 to 0.0061) | -70.1042 (-95.7027 to -11.8153) | 0.0016 (0.0011 to 0.0023) | 0.0006 (0.0001 to 0.0011) | -62.2793 (-96.1729 to -18.8207) |  |
| Israel | 0.0023 (0.0008 to 0.0164) | 0.0007 (0.0002 to 0.0039) | -70.0084 (-90.9686 to -30.8913) | 0.0003 (0.0002 to 0.0005) | 0.0001 (0.0000 to 0.0002) | -62.9843 (-87.8761 to -31.5108) |  |
| Italy | 0.1577 (0.0941 to 0.3488) | 0.0296 (0.0169 to 0.0513) | -81.1966 (-92.9538 to -63.2246) | 0.0627 (0.0399 to 0.0941) | 0.0124 (0.0076 to 0.0184) | -80.1764 (-89.6179 to -63.2980) |  |
| Jamaica | 1.2216 (0.7022 to 1.8574) | 0.0305 (0.0138 to 0.0866) | -97.5062 (-99.0445 to -91.4956) | 0.2293 (0.1319 to 0.3373) | 0.0049 (0.0025 to 0.0099) | -97.8772 (-99.0767 to -94.5947) |  |
| Japan | 0.1570 (0.1042 to 0.2297) | 0.0398 (0.0217 to 0.0643) | -74.6573 (-88.2026 to -55.7144) | 0.0144 (0.0098 to 0.0200) | 0.0037 (0.0020 to 0.0055) | -74.4449 (-85.9701 to -55.5094) |  |
| Jordan | 0.2982 (0.1254 to 0.6874) | 0.0176 (0.0058 to 0.0528) | -94.1149 (-98.3130 to -82.0190) | 0.1304 (0.0535 to 0.2946) | 0.0056 (0.0020 to 0.0116) | -95.6976 (-98.5762 to -87.1976) |  |
| Kazakhstan | 0.0836 (0.0233 to 0.1624) | 0.0010 (0.0004 to 0.0079) | -98.7618 (-99.5412 to -95.7899) | 0.0338 (0.0115 to 0.0534) | 0.0002 (0.0001 to 0.0004) | -99.2824 (-99.5920 to -97.8889) |  |
| Kenya | 2.6282 (1.0922 to 4.9866) | 0.9949 (0.4826 to 1.7163) | -62.1457 (-83.8134 to -6.4789) | 1.5842 (0.6847 to 2.6566) | 0.6217 (0.3156 to 0.9735) | -60.7578 (-82.6712 to -7.2365) |  |
| Kiribati | 0.3315 (0.1187 to 1.3208) | 0.1220 (0.0334 to 0.4708) | -63.2005 (-86.8804 to 2.1981) | 0.0664 (0.0328 to 0.1148) | 0.0210 (0.0088 to 0.0418) | -68.3007 (-88.0154 to -20.4507) |  |
| Kuwait | 0.0022 (0.0006 to 0.0220) | 0.0007 (0.0002 to 0.0024) | -67.8326 (-92.9614 to 6.2581) | 0.0004 (0.0003 to 0.0007) | 0.0002 (0.0001 to 0.0003) | -52.9909 (-92.1584 to -9.1093) |  |
| Kyrgyzstan | 0.0101 (0.0011 to 0.0629) | 0.0008 (0.0002 to 0.0057) | -91.6455 (-97.1911 to -74.2554) | 0.0030 (0.0004 to 0.0045) | 0.0002 (0.0001 to 0.0004) | -94.1626 (-97.1432 to -83.1781) |  |
| Lao People's Democratic Republic | 403.6089 (181.4750 to 749.2993) | 18.7176 (7.7214 to 35.1969) | -95.3625 (-98.2843 to -86.8246) | 124.0015 (57.7047 to 212.0844) | 4.4354 (1.8924 to 8.0156) | -96.4231 (-98.5439 to -90.5407) |  |
| Latvia | 0.0017 (0.0006 to 0.0143) | 0.0088 (0.0001 to 0.0189) | 429.8477 (-92.7703 to 2,199.8452) | 0.0004 (0.0003 to 0.0008) | 0.0035 (0.0000 to 0.0067) | 737.3873 (-92.6634 to 1,911.3116) |  |
| Lebanon | 2.4160 (1.0420 to 4.4695) | 0.2389 (0.1079 to 0.4172) | -90.1109 (-95.4443 to -72.0716) | 1.0183 (0.4367 to 1.7336) | 0.0896 (0.0393 to 0.1463) | -91.2028 (-96.0647 to -75.4672) |  |
| Lesotho | 2.7532 (0.6762 to 7.9015) | 0.9269 (0.3027 to 2.0948) | -66.3342 (-87.8853 to 20.1669) | 0.9083 (0.2480 to 2.3191) | 0.3416 (0.1171 to 0.7715) | -62.3894 (-86.1918 to 25.4484) |  |
| Liberia | 6.3739 (3.0405 to 11.3137) | 0.7198 (0.1681 to 2.8006) | -88.7075 (-96.6329 to -63.3970) | 3.4280 (1.7330 to 5.6771) | 0.3056 (0.0953 to 0.8770) | -91.0848 (-96.7110 to -78.0403) |  |
| Libya | 0.1854 (0.0672 to 0.4786) | 0.0249 (0.0057 to 0.0732) | -86.5839 (-96.5588 to -57.2955) | 0.0772 (0.0320 to 0.1660) | 0.0092 (0.0026 to 0.0223) | -88.0889 (-96.2535 to -62.9259) |  |
| Lithuania | 0.0370 (0.0200 to 0.0634) | 0.0022 (0.0005 to 0.0046) | -94.1785 (-98.0542 to -84.6347) | 0.0167 (0.0084 to 0.0260) | 0.0008 (0.0002 to 0.0015) | -95.0026 (-98.0794 to -89.1997) |  |
| Luxembourg | 0.0092 (0.0045 to 0.0395) | 0.0120 (0.0015 to 0.0217) | 30.0609 (-92.4308 to 239.4577) | 0.0018 (0.0011 to 0.0032) | 0.0028 (0.0003 to 0.0049) | 60.1866 (-88.5521 to 232.8584) |  |
| Madagascar | 8.1279 (5.4538 to 11.7938) | 1.9784 (0.9436 to 4.7589) | -75.6596 (-86.9553 to -45.3743) | 4.9662 (3.5619 to 6.7416) | 1.1052 (0.5781 to 2.2050) | -77.7460 (-87.6840 to -55.5871) |  |
| Malawi | 8.7181 (3.9417 to 17.3567) | 1.7305 (0.8280 to 3.5784) | -80.1504 (-92.7171 to -42.7342) | 5.3709 (2.7318 to 10.5431) | 1.0048 (0.4966 to 1.8952) | -81.2920 (-92.7789 to -45.7424) |  |
| Malaysia | 4.2111 (1.5646 to 8.5222) | 0.3953 (0.1725 to 0.7340) | -90.6123 (-96.2422 to -72.4238) | 1.1113 (0.4492 to 2.0959) | 0.0868 (0.0408 to 0.1528) | -92.1935 (-96.6572 to -76.5126) |  |
| Maldives | 19.4963 (5.8810 to 45.3071) | 0.6390 (0.2605 to 1.4616) | -96.7224 (-98.8733 to -85.5250) | 4.5951 (1.4336 to 9.8470) | 0.1166 (0.0517 to 0.2455) | -97.4627 (-99.0855 to -90.0137) |  |
| Mali | 20.0895 (6.6756 to 44.9755) | 2.6115 (0.9575 to 6.0270) | -87.0008 (-93.6270 to -67.5651) | 10.4404 (3.8192 to 21.8866) | 1.2253 (0.5016 to 2.6046) | -88.2643 (-93.9581 to -70.7049) |  |
| Malta | 0.0790 (0.0201 to 0.1290) | 0.0021 (0.0005 to 0.0076) | -97.3550 (-98.6900 to -94.2660) | 0.0191 (0.0047 to 0.0286) | 0.0004 (0.0001 to 0.0008) | -97.7199 (-98.7740 to -95.5959) |  |
| Marshall Islands | 0.4625 (0.0790 to 1.9072) | 0.1648 (0.0442 to 0.6357) | -64.3746 (-83.8321 to 78.5053) | 0.0989 (0.0213 to 0.2455) | 0.0359 (0.0116 to 0.0792) | -63.7277 (-84.0036 to 39.7684) |  |
| Mauritania | 8.8934 (4.1230 to 19.6561) | 0.7068 (0.2581 to 1.8768) | -92.0530 (-96.7476 to -80.4015) | 4.6766 (2.2693 to 8.9076) | 0.3235 (0.1256 to 0.6938) | -93.0832 (-96.8766 to -82.2647) |  |
| Mauritius | 0.3572 (0.0898 to 0.5962) | 0.0034 (0.0005 to 0.0362) | -99.0382 (-99.8783 to -88.4086) | 0.0762 (0.0219 to 0.1155) | 0.0002 (0.0001 to 0.0004) | -99.7542 (-99.8741 to -98.9183) |  |
| Mexico | 1.0997 (0.7815 to 1.4843) | 0.0145 (0.0070 to 0.0657) | -98.6854 (-99.3737 to -93.9630) | 0.6530 (0.4566 to 0.8323) | 0.0066 (0.0041 to 0.0118) | -98.9948 (-99.3817 to -98.0043) |  |
| Micronesia (Federated States of) | 14.6400 (0.6329 to 36.5460) | 7.3487 (0.3893 to 19.5653) | -49.8044 (-88.3044 to 206.7323) | 3.2164 (0.1242 to 8.7189) | 1.4645 (0.0688 to 3.4820) | -54.4670 (-86.6008 to 177.7055) |  |
| Monaco | 0.0167 (0.0064 to 0.0425) | 0.0091 (0.0035 to 0.0246) | -45.3446 (-79.3821 to 60.4801) | 0.0036 (0.0016 to 0.0089) | 0.0021 (0.0009 to 0.0040) | -41.1789 (-78.1079 to 74.6457) |  |
| Mongolia | 0.0062 (0.0006 to 0.0779) | 0.0008 (0.0000 to 0.0137) | -86.7232 (-99.5843 to -69.1048) | 0.0008 (0.0003 to 0.0021) | 0.0000 (0.0000 to 0.0000) | -98.8408 (-99.6112 to -97.0219) |  |
| Montenegro | 0.0138 (0.0041 to 0.0327) | 0.0068 (0.0017 to 0.0231) | -50.6283 (-86.0307 to 93.9520) | 0.0055 (0.0017 to 0.0124) | 0.0025 (0.0007 to 0.0076) | -53.7929 (-87.1720 to 79.4019) |  |
| Morocco | 6.2905 (3.1366 to 12.0110) | 0.2304 (0.0712 to 0.8194) | -96.3376 (-99.0051 to -85.8242) | 2.8479 (1.5273 to 4.6359) | 0.0835 (0.0303 to 0.2168) | -97.0669 (-98.9413 to -92.9995) |  |
| Mozambique | 13.3556 (7.1944 to 24.3693) | 1.6426 (0.7900 to 2.7146) | -87.7008 (-94.1783 to -70.1898) | 7.6345 (4.2363 to 13.1984) | 0.8613 (0.4449 to 1.4559) | -88.7189 (-94.2829 to -74.7932) |  |
| Myanmar | 54.8598 (16.1258 to 112.3562) | 5.5268 (2.2205 to 14.6030) | -89.9256 (-95.7983 to -72.5960) | 17.0001 (5.3923 to 33.6265) | 1.2517 (0.4968 to 2.5445) | -92.6369 (-96.9385 to -81.1779) |  |
| Namibia | 5.0461 (2.0576 to 13.4508) | 0.3575 (0.1504 to 0.7581) | -92.9150 (-96.9629 to -79.2540) | 1.7649 (0.7236 to 3.9180) | 0.1147 (0.0513 to 0.2139) | -93.5038 (-97.2712 to -83.3582) |  |
| Nauru | 0.2317 (0.0488 to 1.1274) | 0.1016 (0.0260 to 0.4914) | -56.1558 (-83.4134 to 80.3538) | 0.0407 (0.0127 to 0.0820) | 0.0186 (0.0057 to 0.0434) | -54.2911 (-84.2426 to 50.0770) |  |
| Nepal | 60.8974 (29.7545 to 122.2354) | 1.5574 (0.5916 to 4.1431) | -97.4425 (-98.9192 to -93.2336) | 31.6029 (15.2888 to 56.7976) | 0.6373 (0.2688 to 1.3332) | -97.9833 (-99.1007 to -95.2053) |  |
| Netherlands | 0.0019 (0.0009 to 0.0102) | 0.0031 (0.0005 to 0.0052) | 61.5891 (-77.6093 to 303.8307) | 0.0004 (0.0002 to 0.0006) | 0.0008 (0.0001 to 0.0013) | 118.8318 (-69.8638 to 318.2632) |  |
| New Zealand | 0.0322 (0.0112 to 0.1137) | 0.0051 (0.0013 to 0.0172) | -84.0498 (-92.0717 to -68.2442) | 0.0026 (0.0009 to 0.0041) | 0.0004 (0.0001 to 0.0007) | -85.2522 (-92.5776 to -70.0714) |  |
| Nicaragua | 0.4495 (0.0469 to 0.7563) | 0.0054 (0.0005 to 0.0419) | -98.7963 (-99.9225 to -89.1975) | 0.2558 (0.0293 to 0.4307) | 0.0008 (0.0003 to 0.0020) | -99.6941 (-99.9191 to -96.4429) |  |
| Niger | 19.0127 (9.3764 to 37.5247) | 1.9836 (0.9676 to 3.7971) | -89.5668 (-95.8988 to -74.4707) | 10.0908 (5.1378 to 17.5456) | 1.0531 (0.5601 to 1.8555) | -89.5638 (-95.3647 to -76.4595) |  |
| Nigeria | 14.0753 (5.0345 to 26.0259) | 0.8791 (0.3725 to 1.7777) | -93.7542 (-97.5948 to -83.1130) | 6.8841 (2.4301 to 11.2686) | 0.4638 (0.2170 to 0.8555) | -93.2627 (-97.0708 to -82.5113) |  |
| Niue | 0.0816 (0.0199 to 0.3747) | 0.0413 (0.0116 to 0.1810) | -49.3508 (-80.2273 to 108.0424) | 0.0126 (0.0042 to 0.0249) | 0.0068 (0.0026 to 0.0130) | -46.0764 (-79.3137 to 95.9663) |  |
| North Macedonia | 0.0105 (0.0022 to 0.0263) | 0.0018 (0.0004 to 0.0092) | -83.3153 (-96.2761 to -21.3281) | 0.0038 (0.0009 to 0.0071) | 0.0004 (0.0002 to 0.0014) | -88.6983 (-96.1854 to -61.1591) |  |
| Northern Mariana Islands | 3.2338 (0.9828 to 13.1020) | 0.3824 (0.1619 to 0.7042) | -88.1742 (-96.1563 to -65.4249) | 0.5864 (0.1765 to 1.8233) | 0.0749 (0.0324 to 0.1284) | -87.2305 (-95.2627 to -64.8935) |  |
| Norway | 0.0048 (0.0024 to 0.0243) | 0.0193 (0.0019 to 0.0370) | 299.0773 (-81.9606 to 991.9770) | 0.0009 (0.0006 to 0.0015) | 0.0042 (0.0004 to 0.0076) | 354.0531 (-68.6804 to 828.4964) |  |
| Oman | 0.6654 (0.1455 to 2.0052) | 0.0179 (0.0066 to 0.0415) | -97.3034 (-99.3241 to -81.8223) | 0.2763 (0.0534 to 0.7124) | 0.0061 (0.0024 to 0.0124) | -97.8004 (-99.4454 to -86.5357) |  |
| Pakistan | 35.3755 (17.4862 to 63.6269) | 2.0939 (0.8448 to 4.7862) | -94.0810 (-97.5179 to -87.4934) | 11.6453 (6.0921 to 19.5653) | 0.7663 (0.3172 to 1.5696) | -93.4193 (-96.9179 to -86.4862) |  |
| Palau | 0.0280 (0.0006 to 0.2854) | 0.0115 (0.0003 to 0.1100) | -58.9883 (-93.4717 to 305.4982) | 0.0018 (0.0001 to 0.0072) | 0.0007 (0.0000 to 0.0030) | -59.5026 (-87.7069 to 58.5997) |  |
| Palestine | 0.2725 (0.0991 to 0.5944) | 0.0311 (0.0099 to 0.0819) | -88.5710 (-95.9256 to -56.7232) | 0.1279 (0.0495 to 0.2541) | 0.0112 (0.0039 to 0.0222) | -91.2056 (-96.4213 to -69.0524) |  |
| Panama | 0.3262 (0.0760 to 0.5607) | 0.0016 (0.0004 to 0.0091) | -99.4947 (-99.8977 to -95.2801) | 0.1757 (0.0426 to 0.2681) | 0.0004 (0.0002 to 0.0007) | -99.7724 (-99.9013 to -99.0923) |  |
| Papua New Guinea | 1.0760 (0.1256 to 4.2272) | 1.0489 (0.1199 to 4.3625) | -2.5241 (-67.3061 to 249.0237) | 0.2114 (0.0326 to 0.6007) | 0.1895 (0.0304 to 0.7300) | -10.3473 (-66.7813 to 164.0078) |  |
| Paraguay | 2.0022 (1.1789 to 2.8862) | 0.1045 (0.0315 to 0.3314) | -94.7784 (-98.7110 to -83.6729) | 0.8967 (0.5416 to 1.1864) | 0.0318 (0.0119 to 0.0862) | -96.4585 (-98.8960 to -89.9190) |  |
| Peru | 0.9924 (0.5482 to 1.5366) | 0.0582 (0.0199 to 0.2068) | -94.1356 (-98.2654 to -78.1681) | 0.3569 (0.2048 to 0.5149) | 0.0216 (0.0090 to 0.0578) | -93.9400 (-97.8473 to -85.1144) |  |
| Philippines | 9.4034 (5.1028 to 18.9143) | 2.5329 (1.0631 to 4.0826) | -73.0640 (-85.0820 to -45.7526) | 2.2046 (1.3981 to 3.2990) | 0.5215 (0.2409 to 0.8707) | -76.3429 (-86.9313 to -57.2552) |  |
| Poland | 0.1526 (0.0857 to 0.2242) | 0.0017 (0.0008 to 0.0069) | -98.8750 (-99.5479 to -93.4458) | 0.0817 (0.0454 to 0.1167) | 0.0007 (0.0004 to 0.0015) | -99.1556 (-99.5778 to -97.3331) |  |
| Portugal | 0.4823 (0.3003 to 0.6864) | 0.0019 (0.0004 to 0.0138) | -99.6007 (-99.9227 to -96.3705) | 0.1529 (0.0996 to 0.2113) | 0.0003 (0.0001 to 0.0006) | -99.8297 (-99.9280 to -99.4346) |  |
| Puerto Rico | 0.1147 (0.0679 to 0.2581) | 0.0335 (0.0081 to 0.0566) | -70.8039 (-95.5155 to -38.3680) | 0.0213 (0.0137 to 0.0335) | 0.0060 (0.0012 to 0.0103) | -71.6362 (-95.9582 to -42.6442) |  |
| Qatar | 0.2672 (0.1054 to 0.7448) | 0.0379 (0.0113 to 0.0799) | -85.8339 (-97.6476 to -55.3864) | 0.1100 (0.0508 to 0.2818) | 0.0142 (0.0048 to 0.0266) | -87.1381 (-97.6571 to -62.5128) |  |
| Republic of Korea | 0.5142 (0.2732 to 1.1815) | 0.0232 (0.0113 to 0.0490) | -95.4970 (-98.5327 to -88.5882) | 0.1371 (0.0824 to 0.2458) | 0.0047 (0.0025 to 0.0087) | -96.5419 (-98.4555 to -92.5791) |  |
| Republic of Moldova | 0.0047 (0.0017 to 0.0478) | 0.0006 (0.0001 to 0.0049) | -86.3394 (-94.6659 to -69.1566) | 0.0011 (0.0008 to 0.0018) | 0.0002 (0.0001 to 0.0005) | -83.9617 (-94.6819 to -68.5216) |  |
| Romania | 0.1068 (0.0651 to 0.2779) | 0.0127 (0.0057 to 0.0278) | -88.0759 (-96.6849 to -69.2610) | 0.0368 (0.0251 to 0.0555) | 0.0039 (0.0020 to 0.0070) | -89.4089 (-94.9322 to -76.2888) |  |
| Russian Federation | 0.0611 (0.0391 to 0.1085) | 0.0031 (0.0018 to 0.0060) | -94.9331 (-97.3087 to -90.4929) | 0.0273 (0.0184 to 0.0417) | 0.0011 (0.0007 to 0.0019) | -95.8484 (-97.6845 to -92.8403) |  |
| Rwanda | 8.9480 (4.0693 to 16.0856) | 0.8334 (0.3492 to 2.2212) | -90.6857 (-96.0568 to -75.6385) | 5.6532 (2.7217 to 9.5434) | 0.4417 (0.2099 to 0.9738) | -92.1868 (-96.2073 to -82.2131) |  |
| Saint Kitts and Nevis | 0.3344 (0.1288 to 0.5284) | 0.0134 (0.0045 to 0.0348) | -95.9822 (-98.2718 to -86.8504) | 0.0682 (0.0269 to 0.0985) | 0.0018 (0.0008 to 0.0033) | -97.3697 (-98.7010 to -94.1401) |  |
| Saint Lucia | 0.4783 (0.2442 to 0.7170) | 0.0140 (0.0059 to 0.0770) | -97.0755 (-98.8303 to -83.2874) | 0.0995 (0.0507 to 0.1489) | 0.0020 (0.0011 to 0.0035) | -98.0241 (-98.9622 to -93.8452) |  |
| Saint Vincent and the Grenadines | 0.0722 (0.0163 to 0.1936) | 0.0104 (0.0013 to 0.0452) | -85.5904 (-95.5094 to -64.1568) | 0.0132 (0.0037 to 0.0196) | 0.0014 (0.0002 to 0.0025) | -89.5639 (-95.2644 to -77.6461) |  |
| Samoa | 0.3952 (0.0877 to 1.4945) | 0.1721 (0.0452 to 0.5836) | -56.4508 (-80.1694 to 46.5098) | 0.0657 (0.0214 to 0.1450) | 0.0296 (0.0105 to 0.0666) | -54.9824 (-80.6692 to 34.7169) |  |
| San Marino | 0.0480 (0.0089 to 0.0965) | 0.0030 (0.0012 to 0.0137) | -93.7731 (-98.0961 to -65.3690) | 0.0111 (0.0020 to 0.0217) | 0.0005 (0.0003 to 0.0008) | -95.8519 (-98.1720 to -82.0363) |  |
| Sao Tome and Principe | 10.8983 (5.4319 to 19.4175) | 1.2021 (0.4820 to 3.2741) | -88.9695 (-95.9216 to -67.7527) | 5.3407 (2.7616 to 9.0876) | 0.5907 (0.2425 to 1.2179) | -88.9399 (-95.7927 to -71.9357) |  |
| Saudi Arabia | 2.2241 (1.0020 to 4.2144) | 0.1633 (0.0790 to 0.3131) | -92.6565 (-97.1310 to -76.8054) | 0.4453 (0.2079 to 0.7627) | 0.0291 (0.0160 to 0.0487) | -93.4743 (-97.2987 to -81.5615) |  |
| Senegal | 20.0879 (8.9054 to 36.4598) | 2.1619 (0.9960 to 4.7424) | -89.2379 (-94.8838 to -72.6068) | 5.1078 (2.1507 to 9.0957) | 0.4998 (0.2374 to 0.9539) | -90.2152 (-95.2857 to -76.1412) |  |
| Serbia | 0.1010 (0.0093 to 0.2377) | 0.0112 (0.0015 to 0.0251) | -88.9427 (-95.1106 to -59.3023) | 0.0463 (0.0040 to 0.0998) | 0.0049 (0.0006 to 0.0095) | -89.5097 (-95.2671 to -63.5946) |  |
| Seychelles | 1.9971 (0.9333 to 4.4706) | 0.5004 (0.1866 to 1.2573) | -74.9457 (-89.5978 to -9.5624) | 0.4543 (0.2221 to 0.9269) | 0.0937 (0.0404 to 0.1904) | -79.3848 (-90.9104 to -39.9366) |  |
| Sierra Leone | 18.5350 (9.6838 to 30.8377) | 2.9100 (1.4866 to 4.8502) | -84.2998 (-92.2217 to -63.1168) | 9.5117 (5.2261 to 15.0425) | 1.4350 (0.7927 to 2.3346) | -84.9138 (-92.3129 to -65.7893) |  |
| Singapore | 0.0083 (0.0009 to 0.1131) | 0.0018 (0.0000 to 0.0137) | -78.2111 (-97.5598 to 100.5988) | 0.0002 (0.0001 to 0.0003) | 0.0002 (0.0000 to 0.0004) | -11.3967 (-97.1044 to 75.5986) |  |
| Slovakia | 0.0033 (0.0011 to 0.0160) | 0.0012 (0.0002 to 0.0052) | -63.7051 (-89.6634 to -1.3739) | 0.0010 (0.0005 to 0.0019) | 0.0003 (0.0001 to 0.0008) | -64.5509 (-88.8740 to -13.6246) |  |
| Slovenia | 0.0443 (0.0122 to 0.0749) | 0.0164 (0.0007 to 0.0306) | -63.0679 (-95.1815 to -24.7207) | 0.0187 (0.0049 to 0.0307) | 0.0061 (0.0003 to 0.0114) | -67.1920 (-96.1851 to -36.5148) |  |
| Solomon Islands | 0.7294 (0.0733 to 2.4931) | 0.2908 (0.0259 to 0.8591) | -60.1302 (-86.0113 to 49.8884) | 0.1488 (0.0200 to 0.3971) | 0.0630 (0.0055 to 0.1797) | -57.6522 (-85.0116 to 38.9419) |  |
| Somalia | 33.0931 (4.9854 to 87.1912) | 15.2149 (4.1104 to 46.3046) | -54.0241 (-80.9799 to 79.4763) | 18.4728 (2.8887 to 41.5244) | 8.3055 (2.5476 to 19.9722) | -55.0393 (-78.4016 to 70.2305) |  |
| South Africa | 0.7173 (0.3721 to 1.3239) | 0.2332 (0.1113 to 0.6025) | -67.4918 (-84.0009 to -29.2742) | 0.2223 (0.1245 to 0.3767) | 0.0738 (0.0416 to 0.1268) | -66.7897 (-82.0044 to -30.3326) |  |
| South Sudan | 13.7202 (6.1618 to 29.6543) | 4.6838 (2.1575 to 10.5595) | -65.8620 (-85.3389 to 12.1037) | 7.0295 (3.4802 to 13.2085) | 2.4758 (1.2411 to 5.0101) | -64.7793 (-84.1262 to 2.9900) |  |
| Spain | 0.3902 (0.2624 to 0.5576) | 0.0062 (0.0024 to 0.0312) | -98.3984 (-99.3786 to -89.4990) | 0.0574 (0.0407 to 0.0756) | 0.0006 (0.0003 to 0.0013) | -98.9965 (-99.4494 to -97.6862) |  |
| Sri Lanka | 3.9866 (1.5873 to 7.9449) | 0.3869 (0.1673 to 0.7920) | -90.2944 (-96.4380 to -73.1208) | 0.8808 (0.3701 to 1.6339) | 0.0651 (0.0324 to 0.1219) | -92.6110 (-96.6760 to -80.3441) |  |
| Sudan | 4.5467 (1.3926 to 11.5785) | 0.1780 (0.0578 to 0.6626) | -96.0843 (-98.7097 to -86.5269) | 2.5859 (0.8239 to 5.1237) | 0.0824 (0.0299 to 0.1875) | -96.8119 (-98.7669 to -90.3346) |  |
| Suriname | 2.3026 (0.7966 to 3.7234) | 0.2751 (0.1122 to 0.8865) | -88.0535 (-96.1065 to -57.7743) | 0.5208 (0.1873 to 0.8186) | 0.0432 (0.0231 to 0.0830) | -91.7124 (-96.4722 to -76.8712) |  |
| Sweden | 0.0036 (0.0022 to 0.0122) | 0.0007 (0.0002 to 0.0022) | -79.3739 (-92.3847 to -62.2391) | 0.0006 (0.0004 to 0.0008) | 0.0001 (0.0000 to 0.0002) | -78.4627 (-92.2510 to -60.8626) |  |
| Switzerland | 0.0009 (0.0003 to 0.0048) | 0.0023 (0.0003 to 0.0044) | 160.0471 (-78.3990 to 698.3584) | 0.0002 (0.0001 to 0.0005) | 0.0008 (0.0001 to 0.0014) | 256.7677 (-70.9255 to 669.3977) |  |
| Syrian Arab Republic | 1.2987 (0.5175 to 2.9496) | 0.1142 (0.0340 to 0.3916) | -91.2047 (-97.5289 to -67.5822) | 0.5308 (0.2498 to 1.0725) | 0.0371 (0.0143 to 0.0894) | -93.0088 (-97.4520 to -79.4457) |  |
| Taiwan | 0.9432 (0.5003 to 1.3483) | 0.0053 (0.0016 to 0.0310) | -99.4419 (-99.8457 to -94.8772) | 0.1143 (0.0607 to 0.1538) | 0.0003 (0.0002 to 0.0007) | -99.7195 (-99.8715 to -99.0082) |  |
| Tajikistan | 0.0138 (0.0023 to 0.0671) | 0.0022 (0.0002 to 0.0209) | -83.7947 (-98.4704 to -44.4106) | 0.0043 (0.0010 to 0.0097) | 0.0001 (0.0001 to 0.0003) | -96.7960 (-98.3994 to -89.2847) |  |
| Thailand | 6.5459 (3.2487 to 13.1392) | 1.2021 (0.4349 to 2.2022) | -81.6361 (-92.1960 to -58.3547) | 0.9128 (0.4832 to 1.6164) | 0.1556 (0.0560 to 0.2691) | -82.9589 (-92.9104 to -62.8066) |  |
| Timor-Leste | 92.3526 (24.3347 to 203.6608) | 4.8994 (1.3658 to 12.6485) | -94.6949 (-97.6749 to -85.9699) | 25.8180 (8.1389 to 46.8757) | 1.0967 (0.3277 to 2.5613) | -95.7522 (-98.3406 to -90.1838) |  |
| Togo | 5.5747 (2.6828 to 10.0910) | 0.9359 (0.4077 to 2.1675) | -83.2108 (-92.7389 to -55.0199) | 3.3014 (1.6904 to 5.5101) | 0.5087 (0.2416 to 0.9798) | -84.5913 (-93.2610 to -63.4517) |  |
| Tokelau | 0.2309 (0.0523 to 0.8906) | 0.0403 (0.0124 to 0.1558) | -82.5661 (-93.6418 to -22.5231) | 0.0412 (0.0137 to 0.0955) | 0.0074 (0.0029 to 0.0149) | -81.9998 (-93.0840 to -32.0627) |  |
| Tonga | 0.0926 (0.0149 to 0.3426) | 0.0482 (0.0112 to 0.1559) | -47.9902 (-75.7398 to 97.0504) | 0.0170 (0.0036 to 0.0409) | 0.0092 (0.0025 to 0.0207) | -45.8856 (-75.1997 to 86.6152) |  |
| Trinidad and Tobago | 1.2959 (0.1780 to 2.0921) | 0.0151 (0.0051 to 0.0638) | -98.8333 (-99.6657 to -93.6718) | 0.2686 (0.0380 to 0.4109) | 0.0019 (0.0008 to 0.0045) | -99.3088 (-99.7540 to -95.8104) |  |
| Tunisia | 3.5899 (1.2092 to 7.5434) | 0.1367 (0.0686 to 0.2549) | -96.1933 (-98.3448 to -87.7908) | 1.4437 (0.4948 to 2.7464) | 0.0467 (0.0237 to 0.0732) | -96.7674 (-98.4788 to -90.5170) |  |
| Turkmenistan | 0.0535 (0.0311 to 0.1107) | 0.0311 (0.0073 to 0.0497) | -41.9107 (-78.6972 to 7.7206) | 0.0201 (0.0129 to 0.0280) | 0.0116 (0.0027 to 0.0166) | -42.3875 (-81.3416 to 2.0036) |  |
| Tuvalu | 0.5429 (0.0885 to 2.1605) | 0.1908 (0.0495 to 0.7326) | -64.8528 (-86.0060 to 44.7360) | 0.1137 (0.0248 to 0.2624) | 0.0379 (0.0112 to 0.0812) | -66.6876 (-87.0456 to 14.6701) |  |
| Türkiye | 1.1872 (0.4063 to 2.6758) | 0.0289 (0.0135 to 0.0849) | -97.5696 (-99.2401 to -90.1363) | 0.7413 (0.2519 to 1.6319) | 0.0149 (0.0077 to 0.0310) | -97.9894 (-99.2468 to -93.8016) |  |
| Uganda | 9.6761 (2.9866 to 19.2678) | 0.7584 (0.3405 to 1.7847) | -92.1621 (-97.0048 to -77.4646) | 5.1844 (1.8218 to 9.1729) | 0.4192 (0.2108 to 0.8868) | -91.9150 (-96.7877 to -78.2642) |  |
| Ukraine | 0.1308 (0.0922 to 0.1796) | 0.0075 (0.0035 to 0.0296) | -94.2997 (-97.4350 to -76.9868) | 0.0596 (0.0425 to 0.0825) | 0.0024 (0.0016 to 0.0056) | -96.0335 (-97.5752 to -91.1377) |  |
| United Arab Emirates | 7.0614 (0.8683 to 19.7717) | 0.1430 (0.0376 to 0.3051) | -97.9743 (-99.4076 to -90.0319) | 3.3846 (0.4074 to 10.3164) | 0.0715 (0.0157 to 0.1401) | -97.8860 (-99.4456 to -91.0575) |  |
| United Kingdom | 0.0162 (0.0094 to 0.0525) | 0.0038 (0.0021 to 0.0083) | -76.3251 (-91.1306 to -58.4766) | 0.0022 (0.0015 to 0.0043) | 0.0005 (0.0003 to 0.0008) | -76.6889 (-88.6987 to -61.3007) |  |
| United Republic of Tanzania | 5.2654 (2.2239 to 9.7955) | 0.6330 (0.3026 to 1.4699) | -87.9777 (-95.2191 to -69.4271) | 3.4419 (1.4578 to 5.8918) | 0.3884 (0.2047 to 0.7497) | -88.7169 (-94.9996 to -74.4659) |  |
| United States Virgin Islands | 0.0513 (0.0253 to 0.1306) | 0.1800 (0.0691 to 0.2952) | 250.5161 (-27.9370 to 716.6095) | 0.0090 (0.0054 to 0.0149) | 0.0305 (0.0129 to 0.0468) | 240.3388 (-1.5572 to 533.2104) |  |
| United States of America | 0.0198 (0.0131 to 0.0287) | 0.0034 (0.0019 to 0.0076) | -82.9076 (-89.6567 to -63.7430) | 0.0063 (0.0043 to 0.0090) | 0.0010 (0.0006 to 0.0016) | -83.5804 (-89.6630 to -71.0815) |  |
| Uruguay | 0.2309 (0.0398 to 0.4194) | 0.0030 (0.0009 to 0.0209) | -98.6928 (-99.6495 to -92.4161) | 0.0401 (0.0074 to 0.0650) | 0.0003 (0.0001 to 0.0005) | -99.3376 (-99.6777 to -96.4443) |  |
| Uzbekistan | 0.0471 (0.0096 to 0.0897) | 0.0036 (0.0005 to 0.0083) | -92.2627 (-96.1117 to -78.1843) | 0.0172 (0.0037 to 0.0273) | 0.0014 (0.0002 to 0.0025) | -91.7449 (-95.5179 to -83.2268) |  |
| Vanuatu | 0.8119 (0.1120 to 2.9375) | 0.3003 (0.0390 to 0.8590) | -63.0126 (-84.5001 to 34.4391) | 0.1559 (0.0286 to 0.4212) | 0.0601 (0.0093 to 0.1537) | -61.4743 (-84.8895 to 15.4008) |  |
| Venezuela (Bolivarian Republic of) | 0.7669 (0.5295 to 1.1056) | 0.1748 (0.0601 to 0.6703) | -77.2071 (-92.2687 to -11.3869) | 0.2974 (0.2148 to 0.4049) | 0.0513 (0.0186 to 0.1537) | -82.7607 (-94.0061 to -46.8909) |  |
| Viet Nam | 7.4449 (3.1814 to 14.1453) | 0.5376 (0.2258 to 1.1226) | -92.7795 (-97.5763 to -73.7773) | 2.2487 (0.9813 to 4.0298) | 0.1135 (0.0528 to 0.2202) | -94.9519 (-98.1405 to -86.0787) |  |
| Yemen | 11.5769 (1.7673 to 30.5006) | 0.5811 (0.1503 to 2.0484) | -94.9802 (-98.4602 to -76.0349) | 5.5299 (0.8840 to 12.6797) | 0.2255 (0.0626 to 0.5001) | -95.9229 (-98.7632 to -79.7928) |  |
| Zambia | 8.4536 (4.1559 to 16.4271) | 1.3141 (0.4858 to 3.2415) | -84.4551 (-94.9726 to -49.1717) | 4.7785 (2.3442 to 8.0716) | 0.7163 (0.2920 to 1.7130) | -85.0107 (-94.9602 to -54.7174) |  |
| Zimbabwe | 1.2268 (0.5247 to 2.1932) | 0.2674 (0.0759 to 0.8166) | -78.2070 (-95.1762 to -21.4410) | 0.3384 (0.1610 to 0.5843) | 0.0748 (0.0233 to 0.2032) | -77.8882 (-94.6867 to -35.6127) |  |

UI= uncertainty interval.

**Table S4.** Age-standardized prevalence rate (95% UI) per 100,000 population in GBD super region from 1990 to 2023.

| year | Global | High-income | Central Europe, Eastern Europe, and Central Asia | Latin America and Caribbean | North Africa and Middle East | South Asia | Southeast Asia, East Asia, and Oceania | Sub-Saharan Africa |
| --- | --- | --- | --- | --- | --- | --- | --- | --- |
| 1990 | 2.50 (1.91 to 3.12) | 0.01 (0.01 to 0.02) | 0.01 (0.00 to 0.01) | 0.34 (0.26 to 0.43) | 0.49 (0.35 to 0.71) | 4.89 (3.72 to 6.04) | 3.88 (2.93 to 4.94) | 2.42 (1.73 to 3.31) |
| 1991 | 2.44 (1.84 to 3.04) | 0.01 (0.01 to 0.02) | 0.01 (0.00 to 0.01) | 0.33 (0.25 to 0.41) | 0.48 (0.35 to 0.67) | 4.72 (3.58 to 5.83) | 3.78 (2.83 to 4.77) | 2.41 (1.71 to 3.27) |
| 1992 | 2.37 (1.78 to 2.94) | 0.01 (0.01 to 0.02) | 0.01 (0.00 to 0.01) | 0.31 (0.23 to 0.38) | 0.47 (0.34 to 0.63) | 4.53 (3.39 to 5.60) | 3.67 (2.72 to 4.61) | 2.40 (1.71 to 3.24) |
| 1993 | 2.29 (1.72 to 2.85) | 0.01 (0.01 to 0.02) | 0.01 (0.00 to 0.01) | 0.29 (0.22 to 0.36) | 0.46 (0.34 to 0.62) | 4.34 (3.25 to 5.35) | 3.54 (2.61 to 4.47) | 2.37 (1.70 to 3.20) |
| 1994 | 2.21 (1.65 to 2.75) | 0.01 (0.01 to 0.01) | 0.00 (0.00 to 0.01) | 0.27 (0.20 to 0.33) | 0.47 (0.34 to 0.61) | 4.14 (3.10 to 5.13) | 3.41 (2.51 to 4.33) | 2.35 (1.66 to 3.15) |
| 1995 | 2.14 (1.59 to 2.66) | 0.01 (0.01 to 0.01) | 0.00 (0.00 to 0.01) | 0.25 (0.19 to 0.31) | 0.46 (0.33 to 0.60) | 3.95 (2.96 to 4.92) | 3.29 (2.42 to 4.17) | 2.33 (1.63 to 3.11) |
| 1996 | 2.04 (1.51 to 2.55) | 0.01 (0.01 to 0.01) | 0.00 (0.00 to 0.01) | 0.23 (0.18 to 0.29) | 0.45 (0.32 to 0.59) | 3.73 (2.79 to 4.64) | 3.13 (2.29 to 3.98) | 2.28 (1.61 to 3.00) |
| 1997 | 1.90 (1.40 to 2.38) | 0.01 (0.01 to 0.01) | 0.00 (0.00 to 0.01) | 0.21 (0.16 to 0.27) | 0.43 (0.32 to 0.56) | 3.46 (2.58 to 4.31) | 2.89 (2.12 to 3.67) | 2.20 (1.57 to 2.91) |
| 1998 | 1.75 (1.29 to 2.18) | 0.01 (0.01 to 0.01) | 0.00 (0.00 to 0.00) | 0.19 (0.15 to 0.24) | 0.41 (0.30 to 0.54) | 3.17 (2.35 to 3.96) | 2.62 (1.93 to 3.32) | 2.10 (1.50 to 2.81) |
| 1999 | 1.60 (1.18 to 2.00) | 0.01 (0.01 to 0.01) | 0.00 (0.00 to 0.00) | 0.17 (0.13 to 0.22) | 0.39 (0.29 to 0.52) | 2.90 (2.14 to 3.63) | 2.35 (1.75 to 2.99) | 2.00 (1.43 to 2.71) |
| 2000 | 1.48 (1.10 to 1.86) | 0.01 (0.01 to 0.01) | 0.00 (0.00 to 0.00) | 0.16 (0.12 to 0.20) | 0.38 (0.28 to 0.50) | 2.69 (1.97 to 3.37) | 2.14 (1.61 to 2.72) | 1.91 (1.37 to 2.59) |
| 2001 | 1.38 (1.04 to 1.73) | 0.01 (0.01 to 0.01) | 0.00 (0.00 to 0.00) | 0.15 (0.11 to 0.18) | 0.36 (0.27 to 0.47) | 2.51 (1.85 to 3.15) | 1.97 (1.49 to 2.50) | 1.83 (1.33 to 2.46) |
| 2002 | 1.29 (0.98 to 1.60) | 0.01 (0.00 to 0.01) | 0.00 (0.00 to 0.00) | 0.14 (0.10 to 0.17) | 0.34 (0.26 to 0.45) | 2.34 (1.74 to 2.95) | 1.81 (1.36 to 2.27) | 1.74 (1.25 to 2.37) |
| 2003 | 1.20 (0.91 to 1.49) | 0.01 (0.00 to 0.01) | 0.00 (0.00 to 0.00) | 0.13 (0.10 to 0.16) | 0.33 (0.24 to 0.44) | 2.19 (1.63 to 2.76) | 1.65 (1.24 to 2.07) | 1.65 (1.19 to 2.24) |
| 2004 | 1.12 (0.84 to 1.39) | 0.01 (0.00 to 0.01) | 0.00 (0.00 to 0.00) | 0.12 (0.09 to 0.15) | 0.32 (0.24 to 0.42) | 2.05 (1.54 to 2.59) | 1.51 (1.14 to 1.89) | 1.57 (1.12 to 2.11) |
| 2005 | 1.05 (0.79 to 1.32) | 0.00 (0.00 to 0.01) | 0.00 (0.00 to 0.00) | 0.11 (0.09 to 0.14) | 0.31 (0.23 to 0.40) | 1.93 (1.45 to 2.45) | 1.40 (1.07 to 1.75) | 1.50 (1.07 to 1.99) |
| 2006 | 0.99 (0.75 to 1.24) | 0.00 (0.00 to 0.01) | 0.00 (0.00 to 0.00) | 0.11 (0.08 to 0.14) | 0.30 (0.22 to 0.39) | 1.82 (1.38 to 2.31) | 1.30 (0.99 to 1.63) | 1.43 (1.02 to 1.91) |
| 2007 | 0.94 (0.70 to 1.17) | 0.00 (0.00 to 0.01) | 0.00 (0.00 to 0.00) | 0.10 (0.08 to 0.13) | 0.29 (0.21 to 0.38) | 1.72 (1.30 to 2.17) | 1.20 (0.92 to 1.51) | 1.37 (0.98 to 1.83) |
| 2008 | 0.88 (0.66 to 1.10) | 0.00 (0.00 to 0.01) | 0.00 (0.00 to 0.00) | 0.10 (0.07 to 0.13) | 0.28 (0.21 to 0.36) | 1.63 (1.22 to 2.05) | 1.11 (0.84 to 1.40) | 1.31 (0.94 to 1.74) |
| 2009 | 0.83 (0.63 to 1.05) | 0.00 (0.00 to 0.01) | 0.00 (0.00 to 0.00) | 0.09 (0.07 to 0.12) | 0.27 (0.20 to 0.34) | 1.54 (1.15 to 1.94) | 1.03 (0.78 to 1.30) | 1.25 (0.89 to 1.65) |
| 2010 | 0.79 (0.60 to 1.00) | 0.00 (0.00 to 0.01) | 0.00 (0.00 to 0.00) | 0.09 (0.07 to 0.12) | 0.26 (0.20 to 0.34) | 1.47 (1.10 to 1.85) | 0.96 (0.73 to 1.22) | 1.20 (0.86 to 1.57) |
| 2011 | 0.76 (0.57 to 0.96) | 0.00 (0.00 to 0.01) | 0.00 (0.00 to 0.00) | 0.09 (0.07 to 0.12) | 0.25 (0.19 to 0.33) | 1.40 (1.05 to 1.77) | 0.91 (0.69 to 1.15) | 1.15 (0.83 to 1.51) |
| 2012 | 0.73 (0.55 to 0.91) | 0.00 (0.00 to 0.01) | 0.00 (0.00 to 0.00) | 0.09 (0.07 to 0.12) | 0.24 (0.18 to 0.32) | 1.34 (1.00 to 1.69) | 0.86 (0.66 to 1.09) | 1.10 (0.81 to 1.45) |
| 2013 | 0.70 (0.52 to 0.88) | 0.00 (0.00 to 0.00) | 0.00 (0.00 to 0.00) | 0.09 (0.06 to 0.12) | 0.24 (0.18 to 0.31) | 1.28 (0.96 to 1.62) | 0.82 (0.62 to 1.04) | 1.06 (0.78 to 1.37) |
| 2014 | 0.67 (0.50 to 0.85) | 0.00 (0.00 to 0.00) | 0.00 (0.00 to 0.00) | 0.09 (0.06 to 0.12) | 0.23 (0.17 to 0.30) | 1.23 (0.92 to 1.56) | 0.77 (0.59 to 0.99) | 1.01 (0.74 to 1.32) |
| 2015 | 0.64 (0.48 to 0.81) | 0.00 (0.00 to 0.00) | 0.00 (0.00 to 0.00) | 0.09 (0.06 to 0.12) | 0.23 (0.17 to 0.29) | 1.18 (0.89 to 1.50) | 0.73 (0.55 to 0.95) | 0.97 (0.71 to 1.28) |
| 2016 | 0.61 (0.46 to 0.78) | 0.00 (0.00 to 0.00) | 0.00 (0.00 to 0.00) | 0.08 (0.06 to 0.11) | 0.22 (0.16 to 0.29) | 1.14 (0.85 to 1.44) | 0.69 (0.51 to 0.89) | 0.92 (0.67 to 1.21) |
| 2017 | 0.58 (0.43 to 0.74) | 0.00 (0.00 to 0.00) | 0.00 (0.00 to 0.00) | 0.08 (0.06 to 0.11) | 0.21 (0.16 to 0.27) | 1.09 (0.82 to 1.37) | 0.64 (0.47 to 0.83) | 0.86 (0.62 to 1.13) |
| 2018 | 0.55 (0.41 to 0.70) | 0.00 (0.00 to 0.00) | 0.00 (0.00 to 0.00) | 0.08 (0.06 to 0.11) | 0.20 (0.15 to 0.26) | 1.04 (0.79 to 1.31) | 0.59 (0.44 to 0.77) | 0.81 (0.57 to 1.05) |
| 2019 | 0.52 (0.39 to 0.66) | 0.00 (0.00 to 0.00) | 0.00 (0.00 to 0.00) | 0.08 (0.06 to 0.11) | 0.20 (0.15 to 0.25) | 1.00 (0.76 to 1.25) | 0.56 (0.41 to 0.72) | 0.76 (0.53 to 0.98) |
| 2020 | 0.51 (0.38 to 0.65) | 0.00 (0.00 to 0.00) | 0.00 (0.00 to 0.00) | 0.08 (0.05 to 0.10) | 0.19 (0.15 to 0.25) | 0.97 (0.74 to 1.22) | 0.54 (0.40 to 0.70) | 0.72 (0.50 to 0.93) |
| 2021 | 0.49 (0.36 to 0.63) | 0.00 (0.00 to 0.00) | 0.00 (0.00 to 0.00) | 0.07 (0.05 to 0.10) | 0.19 (0.14 to 0.24) | 0.93 (0.69 to 1.17) | 0.54 (0.40 to 0.71) | 0.69 (0.48 to 0.90) |
| 2022 | 0.49 (0.35 to 0.63) | 0.00 (0.00 to 0.00) | 0.00 (0.00 to 0.00) | 0.07 (0.05 to 0.10) | 0.18 (0.14 to 0.24) | 0.89 (0.66 to 1.14) | 0.55 (0.40 to 0.72) | 0.67 (0.47 to 0.87) |
| 2023 | 0.49 (0.35 to 0.63) | 0.00 (0.00 to 0.00) | 0.00 (0.00 to 0.00) | 0.07 (0.05 to 0.10) | 0.18 (0.13 to 0.24) | 0.90 (0.66 to 1.15) | 0.54 (0.40 to 0.71) | 0.66 (0.46 to 0.87) |

GBD= Global Burden of Diseases, Injuries, and Risk Factors Study; UI= uncertainty interval.

**Table S5.** Age-standardized incidence rate (95% UI) per 100,000 population in GBD super region from 1990 to 2023.

| year | Global | High-income | Central Europe, Eastern Europe, and Central Asia | Latin America and Caribbean | North Africa and Middle East | South Asia | Southeast Asia, East Asia, and Oceania | Sub-Saharan Africa |
| --- | --- | --- | --- | --- | --- | --- | --- | --- |
| 1990 | 11.32 (7.98 to 16.36) | 0.13 (0.11 to 0.18) | 0.09 (0.07 to 0.12) | 1.93 (1.57 to 2.45) | 3.52 (1.68 to 6.52) | 23.38 (17.39 to 31.39) | 13.77 (8.53 to 20.91) | 14.83 (7.21 to 25.09) |
| 1991 | 10.23 (7.24 to 14.58) | 0.12 (0.10 to 0.16) | 0.08 (0.06 to 0.11) | 1.75 (1.42 to 2.22) | 3.21 (1.56 to 5.60) | 20.77 (15.54 to 26.87) | 12.28 (7.81 to 18.43) | 14.03 (6.88 to 23.72) |
| 1992 | 9.25 (6.72 to 12.94) | 0.11 (0.09 to 0.15) | 0.08 (0.06 to 0.10) | 1.57 (1.28 to 1.99) | 2.95 (1.42 to 5.06) | 18.44 (13.64 to 23.64) | 10.87 (6.99 to 16.06) | 13.32 (6.74 to 22.66) |
| 1993 | 8.37 (6.01 to 11.76) | 0.10 (0.08 to 0.14) | 0.07 (0.06 to 0.09) | 1.41 (1.15 to 1.77) | 2.90 (1.40 to 4.95) | 16.35 (12.18 to 21.23) | 9.61 (6.19 to 14.31) | 12.63 (6.53 to 21.93) |
| 1994 | 7.61 (5.39 to 10.75) | 0.09 (0.07 to 0.13) | 0.07 (0.05 to 0.09) | 1.26 (1.02 to 1.58) | 2.90 (1.40 to 4.75) | 14.56 (10.95 to 19.24) | 8.53 (5.51 to 12.71) | 12.05 (6.19 to 21.45) |
| 1995 | 7.00 (4.98 to 9.99) | 0.09 (0.06 to 0.12) | 0.06 (0.05 to 0.08) | 1.14 (0.90 to 1.43) | 2.79 (1.33 to 4.74) | 13.16 (9.82 to 17.92) | 7.69 (4.97 to 11.81) | 11.55 (5.92 to 20.81) |
| 1996 | 6.46 (4.64 to 9.06) | 0.08 (0.06 to 0.10) | 0.06 (0.04 to 0.07) | 1.02 (0.82 to 1.28) | 2.63 (1.36 to 4.28) | 11.96 (8.87 to 15.77) | 6.98 (4.51 to 10.57) | 11.14 (5.73 to 19.75) |
| 1997 | 5.94 (4.24 to 8.31) | 0.07 (0.05 to 0.09) | 0.05 (0.04 to 0.07) | 0.90 (0.72 to 1.14) | 2.46 (1.36 to 3.96) | 10.84 (7.98 to 14.10) | 6.31 (4.00 to 9.52) | 10.77 (5.71 to 18.75) |
| 1998 | 5.48 (3.95 to 7.59) | 0.06 (0.05 to 0.08) | 0.04 (0.03 to 0.06) | 0.79 (0.63 to 1.00) | 2.29 (1.35 to 3.75) | 9.85 (7.15 to 12.64) | 5.70 (3.63 to 8.90) | 10.44 (5.60 to 18.60) |
| 1999 | 5.06 (3.65 to 7.07) | 0.05 (0.04 to 0.07) | 0.04 (0.03 to 0.05) | 0.69 (0.55 to 0.89) | 2.12 (1.22 to 3.66) | 8.97 (6.39 to 11.65) | 5.17 (3.25 to 8.20) | 10.10 (5.45 to 18.36) |
| 2000 | 4.71 (3.40 to 6.72) | 0.05 (0.04 to 0.06) | 0.03 (0.02 to 0.05) | 0.61 (0.48 to 0.81) | 1.96 (1.07 to 3.40) | 8.25 (5.75 to 10.89) | 4.73 (2.91 to 7.68) | 9.72 (5.20 to 17.98) |
| 2001 | 4.37 (3.20 to 6.36) | 0.04 (0.03 to 0.06) | 0.03 (0.02 to 0.04) | 0.56 (0.44 to 0.74) | 1.76 (1.03 to 2.90) | 7.61 (5.32 to 10.13) | 4.36 (2.64 to 7.14) | 9.22 (4.84 to 16.77) |
| 2002 | 4.04 (2.93 to 5.99) | 0.04 (0.03 to 0.05) | 0.02 (0.02 to 0.04) | 0.51 (0.39 to 0.68) | 1.55 (0.94 to 2.50) | 6.97 (4.93 to 9.29) | 4.01 (2.37 to 6.56) | 8.65 (4.46 to 15.93) |
| 2003 | 3.72 (2.69 to 5.61) | 0.03 (0.02 to 0.04) | 0.02 (0.02 to 0.04) | 0.47 (0.35 to 0.63) | 1.44 (0.87 to 2.32) | 6.35 (4.52 to 8.39) | 3.68 (2.18 to 6.05) | 8.08 (4.11 to 14.74) |
| 2004 | 3.42 (2.42 to 5.22) | 0.03 (0.02 to 0.04) | 0.02 (0.01 to 0.03) | 0.43 (0.31 to 0.60) | 1.31 (0.80 to 2.16) | 5.76 (4.07 to 7.70) | 3.37 (1.98 to 5.76) | 7.50 (3.76 to 13.59) |
| 2005 | 3.15 (2.18 to 4.94) | 0.03 (0.02 to 0.04) | 0.02 (0.01 to 0.03) | 0.39 (0.28 to 0.57) | 1.16 (0.68 to 1.98) | 5.27 (3.70 to 7.09) | 3.10 (1.78 to 5.43) | 6.98 (3.48 to 13.14) |
| 2006 | 2.90 (1.98 to 4.47) | 0.02 (0.02 to 0.03) | 0.02 (0.01 to 0.03) | 0.36 (0.25 to 0.52) | 1.04 (0.62 to 1.74) | 4.82 (3.37 to 6.48) | 2.84 (1.61 to 4.91) | 6.49 (3.31 to 12.06) |
| 2007 | 2.64 (1.78 to 4.04) | 0.02 (0.02 to 0.03) | 0.01 (0.01 to 0.02) | 0.32 (0.23 to 0.47) | 0.93 (0.57 to 1.50) | 4.34 (2.99 to 5.85) | 2.58 (1.45 to 4.58) | 5.97 (3.06 to 10.92) |
| 2008 | 2.37 (1.61 to 3.80) | 0.02 (0.02 to 0.03) | 0.01 (0.01 to 0.02) | 0.28 (0.20 to 0.43) | 0.81 (0.48 to 1.28) | 3.86 (2.64 to 5.20) | 2.33 (1.31 to 4.17) | 5.44 (2.80 to 9.92) |
| 2009 | 2.14 (1.43 to 3.48) | 0.02 (0.01 to 0.03) | 0.01 (0.01 to 0.02) | 0.25 (0.18 to 0.40) | 0.72 (0.43 to 1.13) | 3.41 (2.29 to 4.66) | 2.12 (1.18 to 3.76) | 4.96 (2.49 to 9.08) |
| 2010 | 1.96 (1.26 to 3.12) | 0.02 (0.01 to 0.03) | 0.01 (0.01 to 0.02) | 0.23 (0.16 to 0.37) | 0.65 (0.38 to 1.07) | 3.04 (2.00 to 4.18) | 1.97 (1.10 to 3.55) | 4.57 (2.32 to 8.60) |
| 2011 | 1.80 (1.17 to 2.78) | 0.02 (0.01 to 0.03) | 0.01 (0.01 to 0.02) | 0.21 (0.15 to 0.34) | 0.59 (0.36 to 0.95) | 2.71 (1.81 to 3.67) | 1.84 (1.02 to 3.28) | 4.25 (2.19 to 7.95) |
| 2012 | 1.64 (1.09 to 2.60) | 0.02 (0.01 to 0.03) | 0.01 (0.01 to 0.02) | 0.20 (0.13 to 0.32) | 0.53 (0.33 to 0.89) | 2.39 (1.53 to 3.48) | 1.69 (0.94 to 3.01) | 3.94 (2.10 to 7.37) |
| 2013 | 1.49 (0.97 to 2.44) | 0.02 (0.01 to 0.03) | 0.01 (0.01 to 0.02) | 0.18 (0.13 to 0.30) | 0.48 (0.30 to 0.78) | 2.09 (1.26 to 3.12) | 1.56 (0.87 to 2.77) | 3.65 (1.98 to 6.63) |
| 2014 | 1.37 (0.88 to 2.29) | 0.01 (0.01 to 0.02) | 0.01 (0.01 to 0.02) | 0.17 (0.12 to 0.28) | 0.43 (0.26 to 0.72) | 1.83 (1.07 to 2.87) | 1.45 (0.80 to 2.55) | 3.38 (1.81 to 6.42) |
| 2015 | 1.26 (0.79 to 2.13) | 0.01 (0.01 to 0.02) | 0.01 (0.01 to 0.02) | 0.16 (0.11 to 0.26) | 0.40 (0.23 to 0.65) | 1.64 (0.93 to 2.71) | 1.36 (0.74 to 2.48) | 3.14 (1.68 to 6.32) |
| 2016 | 1.17 (0.73 to 1.97) | 0.01 (0.01 to 0.02) | 0.01 (0.01 to 0.02) | 0.16 (0.11 to 0.25) | 0.37 (0.22 to 0.62) | 1.50 (0.83 to 2.50) | 1.27 (0.68 to 2.25) | 2.91 (1.59 to 5.82) |
| 2017 | 1.08 (0.67 to 1.83) | 0.01 (0.01 to 0.02) | 0.01 (0.01 to 0.02) | 0.15 (0.10 to 0.24) | 0.35 (0.20 to 0.58) | 1.38 (0.75 to 2.31) | 1.18 (0.62 to 2.05) | 2.68 (1.50 to 5.11) |
| 2018 | 1.00 (0.61 to 1.68) | 0.01 (0.01 to 0.02) | 0.01 (0.00 to 0.02) | 0.14 (0.09 to 0.23) | 0.33 (0.18 to 0.54) | 1.26 (0.68 to 2.14) | 1.10 (0.57 to 1.95) | 2.46 (1.34 to 4.49) |
| 2019 | 0.93 (0.55 to 1.63) | 0.01 (0.01 to 0.02) | 0.01 (0.00 to 0.02) | 0.14 (0.09 to 0.23) | 0.31 (0.17 to 0.55) | 1.16 (0.62 to 1.99) | 1.03 (0.53 to 1.86) | 2.26 (1.19 to 4.37) |
| 2020 | 0.88 (0.50 to 1.62) | 0.01 (0.01 to 0.02) | 0.01 (0.00 to 0.02) | 0.14 (0.09 to 0.23) | 0.29 (0.15 to 0.55) | 1.06 (0.56 to 1.87) | 1.00 (0.49 to 1.83) | 2.09 (1.06 to 4.16) |
| 2021 | 0.82 (0.48 to 1.49) | 0.01 (0.01 to 0.02) | 0.01 (0.00 to 0.01) | 0.13 (0.08 to 0.22) | 0.27 (0.14 to 0.54) | 0.93 (0.49 to 1.63) | 0.99 (0.49 to 1.80) | 1.92 (0.95 to 3.74) |
| 2022 | 0.76 (0.44 to 1.37) | 0.01 (0.01 to 0.02) | 0.00 (0.00 to 0.01) | 0.13 (0.08 to 0.22) | 0.25 (0.12 to 0.52) | 0.81 (0.41 to 1.48) | 0.98 (0.48 to 1.86) | 1.75 (0.87 to 3.41) |
| 2023 | 0.70 (0.40 to 1.22) | 0.01 (0.01 to 0.02) | 0.00 (0.00 to 0.01) | 0.12 (0.08 to 0.20) | 0.22 (0.11 to 0.48) | 0.72 (0.36 to 1.34) | 0.92 (0.46 to 1.82) | 1.61 (0.80 to 3.26) |

GBD= Global Burden of Diseases, Injuries, and Risk Factors Study; UI= uncertainty interval.

**Table S6.** Age-standardized DALY rate (95% UI) per 100,000 population in GBD super region from 1990 to 2023.

| year | Global | High-income | | Central Europe, Eastern Europe, and Central Asia | | Latin America and Caribbean | | North Africa and Middle East | | South Asia | Southeast Asia, East Asia, and Oceania | | Sub-Saharan Africa |  |
| --- | --- | --- | --- | --- | --- | --- | --- | --- | --- | --- | --- | --- | --- | --- |
| 1990 | 386.60 (278.47 to 515.89) | 0.84 (0.66 to 1.01) | | 1.04 (0.80 to 1.29) | | 40.88 (33.47 to 49.05) | | 132.61 (58.70 to 225.85) | | 953.50 (757.86 to 1206.80) | 270.29 (171.45 to 393.24) | | 516.06 (241.29 to 778.90) |  |
| 1991 | 341.01 (245.06 to 460.05) | 0.74 (0.58 to 0.88) | | 0.98 (0.72 to 1.20) | | 35.31 (29.29 to 42.22) | | 116.60 (52.56 to 194.87) | | 813.09 (645.39 to 1021.27) | 235.67 (149.83 to 351.51) | | 488.62 (237.72 to 743.33) |  |
| 1992 | 309.19 (227.32 to 418.65) | 0.65 (0.52 to 0.79) | | 0.93 (0.66 to 1.13) | | 30.84 (25.52 to 37.60) | | 107.61 (48.83 to 181.23) | | 715.06 (567.67 to 889.25) | 208.70 (132.79 to 309.72) | | 466.77 (227.73 to 723.66) |  |
| 1993 | 284.14 (210.58 to 379.55) | 0.59 (0.47 to 0.72) | | 0.90 (0.61 to 1.09) | | 27.21 (22.58 to 33.73) | | 111.84 (52.07 to 198.02) | | 638.98 (511.42 to 794.28) | 185.07 (120.11 to 284.58) | | 445.31 (217.78 to 697.02) |  |
| 1994 | 263.91 (194.35 to 365.11) | 0.52 (0.42 to 0.64) | | 0.83 (0.56 to 1.01) | | 23.95 (19.92 to 29.62) | | 119.20 (54.96 to 206.79) | | 578.42 (462.71 to 714.09) | 165.34 (104.45 to 265.52) | | 426.84 (219.75 to 659.51) |  |
| 1995 | 243.39 (175.58 to 341.44) | 0.47 (0.37 to 0.58) | | 0.75 (0.51 to 0.91) | | 20.90 (17.45 to 25.75) | | 117.45 (52.48 to 198.00) | | 522.75 (423.54 to 641.33) | 146.70 (90.08 to 230.90) | | 405.19 (215.08 to 628.88) |  |
| 1996 | 221.24 (157.32 to 308.33) | 0.42 (0.33 to 0.53) | | 0.63 (0.46 to 0.77) | | 18.11 (15.13 to 22.49) | | 112.14 (52.53 to 193.20) | | 463.39 (364.54 to 563.15) | 129.69 (78.91 to 199.22) | | 382.81 (208.16 to 597.41) |  |
| 1997 | 201.10 (139.92 to 279.86) | 0.38 (0.30 to 0.47) | | 0.53 (0.40 to 0.63) | | 15.64 (12.99 to 19.12) | | 106.61 (51.83 to 190.67) | | 409.50 (317.98 to 505.55) | 115.19 (69.77 to 175.42) | | 363.90 (194.24 to 563.66) |  |
| 1998 | 185.54 (128.28 to 257.50) | 0.34 (0.27 to 0.42) | | 0.44 (0.35 to 0.52) | | 13.64 (11.26 to 17.14) | | 100.68 (51.23 to 179.22) | | 367.19 (284.25 to 457.38) | 103.39 (62.74 to 157.13) | | 351.73 (183.77 to 556.66) |  |
| 1999 | 171.90 (120.77 to 239.00) | 0.30 (0.24 to 0.36) | | 0.39 (0.32 to 0.47) | | 11.99 (9.69 to 15.32) | | 92.80 (49.88 to 167.70) | | 332.39 (255.73 to 415.91) | 92.90 (54.60 to 146.65) | | 339.54 (184.80 to 546.32) |  |
| 2000 | 158.73 (111.92 to 225.27) | 0.26 (0.21 to 0.32) | | 0.34 (0.28 to 0.41) | | 10.40 (8.27 to 13.56) | | 84.20 (46.31 to 145.60) | | 302.55 (236.65 to 384.90) | 83.33 (50.51 to 132.01) | | 323.52 (177.66 to 543.25) |  |
| 2001 | 145.56 (106.38 to 204.97) | 0.22 (0.18 to 0.28) | | 0.31 (0.25 to 0.37) | | 9.24 (7.20 to 12.31) | | 72.77 (41.13 to 122.75) | | 277.31 (222.11 to 356.95) | 75.39 (44.47 to 117.37) | | 299.43 (167.04 to 512.83) |  |
| 2002 | 133.68 (99.13 to 188.04) | 0.20 (0.16 to 0.26) | | 0.27 (0.22 to 0.33) | | 8.28 (6.35 to 11.21) | | 60.43 (34.70 to 101.30) | | 254.45 (202.86 to 330.50) | 67.80 (40.34 to 107.54) | | 278.74 (152.40 to 478.84) |  |
| 2003 | 123.45 (91.31 to 176.15) | 0.18 (0.14 to 0.23) | | 0.24 (0.19 to 0.30) | | 7.48 (5.66 to 10.40) | | 55.94 (32.57 to 93.96) | | 231.34 (185.08 to 294.05) | 61.26 (37.18 to 99.40) | | 262.60 (137.55 to 452.29) |  |
| 2004 | 113.21 (83.38 to 162.11) | 0.15 (0.12 to 0.20) | | 0.20 (0.16 to 0.24) | | 6.84 (5.12 to 9.73) | | 51.80 (28.96 to 87.90) | | 208.91 (170.21 to 257.94) | 55.96 (33.81 to 91.89) | | 243.14 (127.42 to 417.52) |  |
| 2005 | 102.21 (74.69 to 147.01) | 0.13 (0.11 to 0.17) | | 0.18 (0.15 to 0.22) | | 6.27 (4.63 to 9.02) | | 45.06 (24.61 to 78.36) | | 187.05 (148.38 to 233.49) | 49.65 (29.64 to 85.02) | | 222.29 (117.08 to 388.32) |  |
| 2006 | 91.99 (66.06 to 132.83) | 0.12 (0.10 to 0.15) | | 0.16 (0.13 to 0.19) | | 5.71 (4.12 to 8.53) | | 39.32 (21.77 to 70.14) | | 166.71 (129.06 to 206.53) | 44.21 (25.86 to 73.26) | | 204.21 (107.86 to 348.49) |  |
| 2007 | 82.53 (57.69 to 117.05) | 0.11 (0.09 to 0.13) | | 0.13 (0.11 to 0.16) | | 5.05 (3.74 to 7.62) | | 34.24 (18.99 to 59.89) | | 148.23 (112.03 to 184.07) | 39.51 (22.89 to 66.06) | | 186.21 (98.14 to 309.05) |  |
| 2008 | 73.68 (50.60 to 104.28) | 0.10 (0.08 to 0.13) | | 0.11 (0.09 to 0.14) | | 4.39 (3.20 to 6.73) | | 29.13 (16.05 to 49.35) | | 130.14 (96.86 to 163.70) | 35.72 (20.26 to 58.64) | | 169.04 (88.00 to 275.94) |  |
| 2009 | 64.43 (43.15 to 90.60) | 0.10 (0.08 to 0.12) | | 0.10 (0.08 to 0.12) | | 3.82 (2.74 to 5.71) | | 25.16 (14.47 to 41.99) | | 109.19 (81.48 to 141.98) | 31.74 (17.71 to 51.31) | | 153.09 (81.72 to 247.55) |  |
| 2010 | 57.02 (37.89 to 80.68) | 0.09 (0.07 to 0.11) | | 0.09 (0.07 to 0.11) | | 3.40 (2.43 to 5.00) | | 22.48 (12.84 to 37.39) | | 92.50 (69.08 to 121.27) | 28.58 (15.65 to 45.11) | | 138.73 (75.00 to 222.57) |  |
| 2011 | 51.16 (33.82 to 71.65) | 0.08 (0.07 to 0.10) | | 0.09 (0.08 to 0.11) | | 3.03 (2.15 to 4.54) | | 20.20 (11.36 to 33.95) | | 79.70 (58.19 to 105.88) | 25.86 (13.98 to 39.68) | | 127.22 (69.06 to 198.85) |  |
| 2012 | 46.24 (30.62 to 66.08) | 0.08 (0.06 to 0.10) | | 0.09 (0.07 to 0.11) | | 2.78 (1.97 to 4.20) | | 17.90 (10.24 to 29.34) | | 69.40 (50.91 to 92.65) | 23.12 (12.78 to 35.84) | | 118.82 (66.94 to 186.69) |  |
| 2013 | 42.13 (27.59 to 59.86) | 0.07 (0.06 to 0.09) | | 0.08 (0.07 to 0.10) | | 2.56 (1.82 to 4.10) | | 15.59 (8.91 to 25.32) | | 61.19 (43.63 to 83.61) | 20.96 (11.86 to 32.51) | | 110.96 (63.87 to 178.43) |  |
| 2014 | 38.48 (25.24 to 56.89) | 0.07 (0.05 to 0.09) | | 0.08 (0.07 to 0.10) | | 2.40 (1.70 to 3.86) | | 13.62 (7.85 to 22.08) | | 54.20 (37.11 to 76.82) | 19.18 (11.09 to 30.73) | | 103.22 (60.35 to 167.74) |  |
| 2015 | 35.01 (22.87 to 52.52) | 0.07 (0.05 to 0.09) | | 0.08 (0.07 to 0.10) | | 2.27 (1.60 to 3.63) | | 12.32 (7.32 to 20.31) | | 48.18 (32.03 to 70.75) | 17.69 (10.35 to 28.05) | | 95.11 (56.50 to 156.30) |  |
| 2016 | 31.75 (20.53 to 48.92) | 0.06 (0.05 to 0.09) | | 0.07 (0.06 to 0.09) | | 2.18 (1.52 to 3.50) | | 11.28 (6.68 to 18.69) | | 43.25 (27.80 to 66.04) | 15.90 (9.70 to 25.08) | | 87.01 (51.78 to 145.15) |  |
| 2017 | 29.17 (18.92 to 45.17) | 0.06 (0.05 to 0.08) | | 0.06 (0.05 to 0.08) | | 2.07 (1.45 to 3.40) | | 10.47 (6.05 to 17.36) | | 39.33 (24.83 to 61.11) | 14.70 (9.04 to 23.61) | | 79.40 (47.43 to 134.01) |  |
| 2018 | 26.75 (17.17 to 40.81) | 0.06 (0.05 to 0.08) | | 0.06 (0.05 to 0.08) | | 1.98 (1.36 to 3.22) | | 10.00 (5.64 to 17.30) | | 35.22 (21.89 to 55.48) | 13.68 (8.22 to 22.36) | | 72.30 (42.47 to 122.23) |  |
| 2019 | 24.71 (15.36 to 36.78) | 0.06 (0.04 to 0.08) | | 0.06 (0.05 to 0.07) | | 1.84 (1.27 to 2.96) | | 9.50 (5.26 to 17.00) | | 31.83 (19.82 to 50.15) | 12.90 (7.52 to 20.83) | | 65.85 (38.08 to 109.03) |  |
| 2020 | 23.61 (14.42 to 35.78) | 0.06 (0.04 to 0.08) | | 0.06 (0.04 to 0.07) | | 1.81 (1.22 to 2.91) | | 9.07 (4.86 to 16.65) | | 30.19 (18.40 to 46.43) | 12.72 (7.02 to 20.37) | | 61.11 (34.89 to 103.02) |  |
| 2021 | 22.07 (13.41 to 34.35) | 0.06 (0.04 to 0.07) | | 0.05 (0.04 to 0.07) | | 1.75 (1.19 to 2.85) | | 8.51 (4.49 to 15.89) | | 26.86 (16.26 to 41.84) | 13.21 (7.44 to 21.74) | | 56.22 (31.64 to 95.42) |  |
| 2022 | 20.04 (12.21 to 30.92) | 0.06 (0.04 to 0.07) | 0.05 (0.04 to 0.06) | | 1.69 (1.13 to 2.68) | | 7.61 (3.94 to 13.91) | | 23.58 (13.81 to 37.14) | | 12.36 (7.17 to 20.22) | 51.07 (28.90 to 87.57) | | |
| 2023 | 17.88 (10.86 to 27.38) | 0.05 (0.04 to 0.07) | 0.05 (0.04 to 0.06) | | 1.63 (1.07 to 2.56) | | 6.44 (3.38 to 11.78) | | 20.47 (11.99 to 32.50) | | 11.59 (6.62 to 19.15) | 46.07 (25.71 to 77.17) | | |

DALY= disability-adjusted life year; GBD= Global Burden of Diseases, Injuries, and Risk Factors Study; UI= uncertainty interval.

**Table S7.** Age-standardized mortality rate (95% UI) per 100,000 population in GBD super region from 1990 to 2023.

| year | Global | High-income | Central Europe, Eastern Europe, and Central Asia | Latin America and Caribbean | North Africa and Middle East | South Asia | Southeast Asia, East Asia, and Oceania | Sub-Saharan Africa |
| --- | --- | --- | --- | --- | --- | --- | --- | --- |
| 1990 | 4.94 (3.59 to 6.38) | 0.03 (0.02 to 0.03) | 0.04 (0.03 to 0.05) | 0.77 (0.64 to 0.90) | 1.76 (0.86 to 2.74) | 12.15 (9.60 to 15.17) | 3.84 (2.37 to 5.56) | 7.88 (3.76 to 12.26) |
| 1991 | 4.36 (3.20 to 5.69) | 0.02 (0.02 to 0.03) | 0.04 (0.03 to 0.04) | 0.67 (0.56 to 0.79) | 1.55 (0.73 to 2.50) | 10.42 (8.21 to 13.14) | 3.34 (2.09 to 4.82) | 7.45 (3.65 to 11.70) |
| 1992 | 3.96 (2.93 to 5.23) | 0.02 (0.02 to 0.03) | 0.03 (0.03 to 0.04) | 0.59 (0.50 to 0.69) | 1.42 (0.69 to 2.32) | 9.19 (7.21 to 11.61) | 2.96 (1.86 to 4.26) | 7.09 (3.47 to 11.56) |
| 1993 | 3.64 (2.70 to 4.84) | 0.02 (0.02 to 0.02) | 0.03 (0.02 to 0.04) | 0.52 (0.44 to 0.62) | 1.45 (0.72 to 2.46) | 8.21 (6.60 to 10.42) | 2.64 (1.67 to 3.84) | 6.77 (3.49 to 11.31) |
| 1994 | 3.38 (2.49 to 4.60) | 0.02 (0.01 to 0.02) | 0.03 (0.02 to 0.04) | 0.46 (0.39 to 0.55) | 1.52 (0.76 to 2.48) | 7.45 (5.91 to 9.22) | 2.37 (1.47 to 3.55) | 6.51 (3.38 to 10.59) |
| 1995 | 3.12 (2.28 to 4.27) | 0.02 (0.01 to 0.02) | 0.03 (0.02 to 0.03) | 0.41 (0.35 to 0.48) | 1.48 (0.73 to 2.39) | 6.76 (5.37 to 8.25) | 2.12 (1.33 to 3.09) | 6.22 (3.30 to 9.99) |
| 1996 | 2.85 (2.02 to 3.88) | 0.01 (0.01 to 0.02) | 0.02 (0.02 to 0.03) | 0.35 (0.30 to 0.42) | 1.41 (0.73 to 2.30) | 6.03 (4.71 to 7.31) | 1.88 (1.18 to 2.79) | 5.92 (3.16 to 9.52) |
| 1997 | 2.61 (1.85 to 3.56) | 0.01 (0.01 to 0.02) | 0.02 (0.01 to 0.02) | 0.31 (0.26 to 0.36) | 1.34 (0.70 to 2.25) | 5.39 (4.07 to 6.55) | 1.69 (1.04 to 2.46) | 5.69 (2.98 to 9.26) |
| 1998 | 2.42 (1.73 to 3.34) | 0.01 (0.01 to 0.01) | 0.02 (0.01 to 0.02) | 0.27 (0.23 to 0.31) | 1.26 (0.68 to 2.11) | 4.88 (3.73 to 5.99) | 1.53 (0.94 to 2.26) | 5.55 (3.02 to 8.92) |
| 1999 | 2.25 (1.59 to 3.12) | 0.01 (0.01 to 0.01) | 0.01 (0.01 to 0.02) | 0.23 (0.20 to 0.28) | 1.17 (0.66 to 1.96) | 4.44 (3.41 to 5.51) | 1.39 (0.87 to 2.11) | 5.41 (2.91 to 8.88) |
| 2000 | 2.09 (1.51 to 2.96) | 0.01 (0.01 to 0.01) | 0.01 (0.01 to 0.02) | 0.20 (0.17 to 0.24) | 1.06 (0.62 to 1.71) | 4.07 (3.13 to 5.17) | 1.26 (0.78 to 1.92) | 5.22 (2.87 to 8.66) |
| 2001 | 1.93 (1.41 to 2.74) | 0.01 (0.01 to 0.01) | 0.01 (0.01 to 0.01) | 0.18 (0.15 to 0.22) | 0.93 (0.56 to 1.45) | 3.75 (2.90 to 4.75) | 1.16 (0.72 to 1.77) | 4.90 (2.58 to 8.31) |
| 2002 | 1.78 (1.31 to 2.50) | 0.01 (0.01 to 0.01) | 0.01 (0.01 to 0.01) | 0.16 (0.13 to 0.20) | 0.78 (0.47 to 1.22) | 3.45 (2.68 to 4.38) | 1.06 (0.64 to 1.66) | 4.61 (2.41 to 7.92) |
| 2003 | 1.65 (1.21 to 2.32) | 0.01 (0.00 to 0.01) | 0.01 (0.01 to 0.01) | 0.14 (0.12 to 0.18) | 0.72 (0.44 to 1.16) | 3.14 (2.46 to 3.92) | 0.97 (0.58 to 1.53) | 4.38 (2.20 to 7.38) |
| 2004 | 1.52 (1.12 to 2.15) | 0.01 (0.00 to 0.01) | 0.01 (0.01 to 0.01) | 0.13 (0.11 to 0.17) | 0.67 (0.40 to 1.10) | 2.84 (2.19 to 3.48) | 0.90 (0.52 to 1.43) | 4.07 (2.04 to 6.99) |
| 2005 | 1.38 (1.02 to 1.99) | 0.00 (0.00 to 0.01) | 0.01 (0.01 to 0.01) | 0.12 (0.09 to 0.16) | 0.59 (0.35 to 0.96) | 2.54 (1.98 to 3.12) | 0.81 (0.47 to 1.29) | 3.74 (1.89 to 6.39) |
| 2006 | 1.25 (0.91 to 1.79) | 0.00 (0.00 to 0.01) | 0.01 (0.00 to 0.01) | 0.11 (0.08 to 0.15) | 0.52 (0.31 to 0.85) | 2.29 (1.79 to 2.85) | 0.72 (0.42 to 1.19) | 3.46 (1.75 to 5.78) |
| 2007 | 1.13 (0.81 to 1.59) | 0.00 (0.00 to 0.01) | 0.01 (0.00 to 0.01) | 0.10 (0.07 to 0.13) | 0.46 (0.27 to 0.77) | 2.05 (1.54 to 2.60) | 0.65 (0.38 to 1.05) | 3.17 (1.60 to 5.34) |
| 2008 | 1.01 (0.71 to 1.41) | 0.00 (0.00 to 0.00) | 0.00 (0.00 to 0.01) | 0.09 (0.07 to 0.11) | 0.40 (0.23 to 0.62) | 1.81 (1.34 to 2.35) | 0.60 (0.34 to 0.98) | 2.89 (1.49 to 4.91) |
| 2009 | 0.90 (0.61 to 1.23) | 0.00 (0.00 to 0.00) | 0.00 (0.00 to 0.00) | 0.08 (0.06 to 0.10) | 0.35 (0.21 to 0.53) | 1.54 (1.15 to 2.00) | 0.54 (0.30 to 0.87) | 2.64 (1.38 to 4.41) |
| 2010 | 0.80 (0.54 to 1.12) | 0.00 (0.00 to 0.00) | 0.00 (0.00 to 0.00) | 0.07 (0.05 to 0.09) | 0.31 (0.19 to 0.48) | 1.32 (0.97 to 1.72) | 0.49 (0.27 to 0.79) | 2.40 (1.27 to 3.99) |
| 2011 | 0.72 (0.48 to 1.01) | 0.00 (0.00 to 0.00) | 0.00 (0.00 to 0.00) | 0.06 (0.05 to 0.08) | 0.28 (0.17 to 0.44) | 1.14 (0.83 to 1.49) | 0.45 (0.25 to 0.72) | 2.21 (1.21 to 3.63) |
| 2012 | 0.65 (0.44 to 0.91) | 0.00 (0.00 to 0.00) | 0.00 (0.00 to 0.00) | 0.06 (0.04 to 0.08) | 0.25 (0.15 to 0.38) | 1.00 (0.71 to 1.33) | 0.41 (0.22 to 0.64) | 2.06 (1.15 to 3.38) |
| 2013 | 0.60 (0.40 to 0.85) | 0.00 (0.00 to 0.00) | 0.00 (0.00 to 0.00) | 0.05 (0.04 to 0.07) | 0.22 (0.13 to 0.34) | 0.88 (0.60 to 1.23) | 0.37 (0.21 to 0.60) | 1.94 (1.09 to 3.28) |
| 2014 | 0.55 (0.37 to 0.80) | 0.00 (0.00 to 0.00) | 0.00 (0.00 to 0.00) | 0.05 (0.04 to 0.07) | 0.20 (0.12 to 0.30) | 0.78 (0.52 to 1.12) | 0.34 (0.19 to 0.56) | 1.81 (1.01 to 3.06) |
| 2015 | 0.50 (0.33 to 0.76) | 0.00 (0.00 to 0.00) | 0.00 (0.00 to 0.00) | 0.05 (0.04 to 0.06) | 0.18 (0.11 to 0.29) | 0.70 (0.46 to 1.02) | 0.32 (0.18 to 0.53) | 1.68 (0.94 to 2.91) |
| 2016 | 0.46 (0.30 to 0.71) | 0.00 (0.00 to 0.00) | 0.00 (0.00 to 0.00) | 0.04 (0.03 to 0.06) | 0.17 (0.10 to 0.26) | 0.63 (0.41 to 0.94) | 0.29 (0.17 to 0.48) | 1.55 (0.89 to 2.76) |
| 2017 | 0.42 (0.28 to 0.67) | 0.00 (0.00 to 0.00) | 0.00 (0.00 to 0.00) | 0.04 (0.03 to 0.06) | 0.15 (0.10 to 0.24) | 0.57 (0.36 to 0.87) | 0.27 (0.15 to 0.45) | 1.43 (0.82 to 2.60) |
| 2018 | 0.39 (0.25 to 0.61) | 0.00 (0.00 to 0.00) | 0.00 (0.00 to 0.00) | 0.04 (0.03 to 0.06) | 0.15 (0.09 to 0.24) | 0.51 (0.31 to 0.78) | 0.25 (0.14 to 0.42) | 1.32 (0.76 to 2.40) |
| 2019 | 0.36 (0.22 to 0.56) | 0.00 (0.00 to 0.00) | 0.00 (0.00 to 0.00) | 0.04 (0.03 to 0.05) | 0.14 (0.08 to 0.23) | 0.46 (0.28 to 0.70) | 0.24 (0.13 to 0.40) | 1.21 (0.67 to 2.22) |
| 2020 | 0.34 (0.21 to 0.53) | 0.00 (0.00 to 0.00) | 0.00 (0.00 to 0.00) | 0.04 (0.03 to 0.05) | 0.13 (0.08 to 0.22) | 0.43 (0.26 to 0.67) | 0.23 (0.12 to 0.39) | 1.11 (0.61 to 2.04) |
| 2021 | 0.32 (0.20 to 0.49) | 0.00 (0.00 to 0.00) | 0.00 (0.00 to 0.00) | 0.04 (0.03 to 0.05) | 0.12 (0.07 to 0.21) | 0.39 (0.23 to 0.60) | 0.24 (0.12 to 0.41) | 1.02 (0.55 to 1.86) |
| 2022 | 0.29 (0.18 to 0.46) | 0.00 (0.00 to 0.00) | 0.00 (0.00 to 0.00) | 0.03 (0.03 to 0.05) | 0.11 (0.06 to 0.19) | 0.34 (0.19 to 0.54) | 0.22 (0.11 to 0.38) | 0.94 (0.51 to 1.67) |
| 2023 | 0.27 (0.16 to 0.41) | 0.00 (0.00 to 0.00) | 0.00 (0.00 to 0.00) | 0.03 (0.02 to 0.05) | 0.09 (0.05 to 0.16) | 0.30 (0.17 to 0.49) | 0.21 (0.11 to 0.35) | 0.86 (0.46 to 1.55) |

GBD= Global Burden of Diseases, Injuries, and Risk Factors Study; UI= uncertainty interval.

**Table S8.** Age-standardized YLD rate (95% UI) per 100,000 population from 1990 to 2023.

| **year** | **Global** | **High-income** | **Central Europe, Eastern Europe, and Central Asia** | **Latin America and Caribbean** | **North Africa and Middle East** | **South Asia** | **Southeast Asia, East Asia, and Oceania** | **Sub-Saharan Africa** |
| --- | --- | --- | --- | --- | --- | --- | --- | --- |
| **1990** | 0.1745 (0.1226 to 0.2290) | 0.0030 (0.0020 to 0.0040) | 0.0010 (0.0000 to 0.0010) | 0.0452 (0.0330 to 0.0590) | 0.0550 (0.0370 to 0.0790) | 0.3154 (0.2140 to 0.4239) | 0.2690 (0.1910 to 0.3514) | 0.1570 (0.0890 to 0.2460) |
| **1991** | 0.1665 (0.1183 to 0.2170) | 0.0020 (0.0010 to 0.0030) | 0.0010 (0.0000 to 0.0010) | 0.0420 (0.0310 to 0.0550) | 0.0530 (0.0350 to 0.0730) | 0.2970 (0.2010 to 0.3970) | 0.2570 (0.1830 to 0.3345) | 0.1520 (0.0880 to 0.2350) |
| **1992** | 0.1590 (0.1131 to 0.2070) | 0.0020 (0.0010 to 0.0030) | 0.0010 (0.0000 to 0.0010) | 0.0390 (0.0290 to 0.0510) | 0.0510 (0.0350 to 0.0670) | 0.2790 (0.1910 to 0.3720) | 0.2451 (0.1770 to 0.3190) | 0.1470 (0.0870 to 0.2220) |
| **1993** | 0.1510 (0.1082 to 0.1970) | 0.0020 (0.0010 to 0.0030) | 0.0010 (0.0000 to 0.0010) | 0.0370 (0.0270 to 0.0473) | 0.0504 (0.0340 to 0.0670) | 0.2620 (0.1811 to 0.3460) | 0.2340 (0.1710 to 0.3024) | 0.1420 (0.0860 to 0.2173) |
| **1994** | 0.1450 (0.1040 to 0.1870) | 0.0020 (0.0010 to 0.0030) | 0.0010 (0.0000 to 0.0010) | 0.0340 (0.0250 to 0.0440) | 0.0500 (0.0340 to 0.0680) | 0.2470 (0.1700 to 0.3230) | 0.2242 (0.1640 to 0.2900) | 0.1380 (0.0850 to 0.2154) |
| **1995** | 0.1390 (0.1001 to 0.1800) | 0.0010 (0.0010 to 0.0020) | 0.0010 (0.0000 to 0.0010) | 0.0320 (0.0230 to 0.0400) | 0.0490 (0.0330 to 0.0660) | 0.2340 (0.1610 to 0.3060) | 0.2160 (0.1580 to 0.2780) | 0.1340 (0.0820 to 0.2096) |
| **1996** | 0.1330 (0.0960 to 0.1720) | 0.0010 (0.0010 to 0.0020) | 0.0010 (0.0000 to 0.0010) | 0.0290 (0.0214 to 0.0370) | 0.0470 (0.0320 to 0.0640) | 0.2210 (0.1523 to 0.2860) | 0.2070 (0.1520 to 0.2680) | 0.1310 (0.0810 to 0.2050) |
| **1997** | 0.1260 (0.0910 to 0.1640) | 0.0010 (0.0010 to 0.0020) | 0.0000 (0.0000 to 0.0010) | 0.0270 (0.0190 to 0.0340) | 0.0460 (0.0310 to 0.0604) | 0.2080 (0.1421 to 0.2700) | 0.1970 (0.1448 to 0.2560) | 0.1270 (0.0800 to 0.1960) |
| **1998** | 0.1190 (0.0850 to 0.1530) | 0.0010 (0.0010 to 0.0020) | 0.0000 (0.0000 to 0.0010) | 0.0240 (0.0174 to 0.0300) | 0.0440 (0.0300 to 0.0570) | 0.1940 (0.1320 to 0.2520) | 0.1860 (0.1350 to 0.2423) | 0.1236 (0.0780 to 0.1890) |
| **1999** | 0.1130 (0.0800 to 0.1440) | 0.0010 (0.0010 to 0.0020) | 0.0000 (0.0000 to 0.0010) | 0.0220 (0.0160 to 0.0277) | 0.0420 (0.0290 to 0.0550) | 0.1810 (0.1236 to 0.2332) | 0.1763 (0.1290 to 0.2270) | 0.1200 (0.0760 to 0.1810) |
| **2000** | 0.1080 (0.0760 to 0.1380) | 0.0010 (0.0010 to 0.0020) | 0.0000 (0.0000 to 0.0010) | 0.0200 (0.0140 to 0.0260) | 0.0410 (0.0277 to 0.0541) | 0.1710 (0.1160 to 0.2216) | 0.1680 (0.1234 to 0.2137) | 0.1160 (0.0740 to 0.1790) |
| **2001** | 0.1029 (0.0740 to 0.1320) | 0.0010 (0.0010 to 0.0020) | 0.0000 (0.0000 to 0.0010) | 0.0190 (0.0133 to 0.0242) | 0.0390 (0.0269 to 0.0520) | 0.1630 (0.1128 to 0.2110) | 0.1610 (0.1177 to 0.2031) | 0.1120 (0.0710 to 0.1770) |
| **2002** | 0.0980 (0.0720 to 0.1260) | 0.0010 (0.0000 to 0.0010) | 0.0000 (0.0000 to 0.0010) | 0.0175 (0.0120 to 0.0220) | 0.0369 (0.0260 to 0.0499) | 0.1544 (0.1070 to 0.2010) | 0.1530 (0.1117 to 0.1920) | 0.1070 (0.0680 to 0.1665) |
| **2003** | 0.0930 (0.0680 to 0.1199) | 0.0010 (0.0000 to 0.0010) | 0.0000 (0.0000 to 0.0010) | 0.0160 (0.0120 to 0.0210) | 0.0360 (0.0250 to 0.0480) | 0.1470 (0.1030 to 0.1920) | 0.1460 (0.1060 to 0.1840) | 0.1020 (0.0650 to 0.1540) |
| **2004** | 0.0890 (0.0650 to 0.1150) | 0.0010 (0.0000 to 0.0010) | 0.0000 (0.0000 to 0.0000) | 0.0155 (0.0110 to 0.0200) | 0.0340 (0.0241 to 0.0449) | 0.1400 (0.0990 to 0.1840) | 0.1390 (0.1010 to 0.1740) | 0.0970 (0.0620 to 0.1460) |
| **2005** | 0.0850 (0.0620 to 0.1110) | 0.0010 (0.0000 to 0.0010) | 0.0000 (0.0000 to 0.0000) | 0.0150 (0.0100 to 0.0190) | 0.0330 (0.0240 to 0.0430) | 0.1341 (0.0950 to 0.1760) | 0.1318 (0.0970 to 0.1664) | 0.0930 (0.0596 to 0.1430) |
| **2006** | 0.0810 (0.0590 to 0.1050) | 0.0010 (0.0000 to 0.0010) | 0.0000 (0.0000 to 0.0000) | 0.0140 (0.0100 to 0.0180) | 0.0320 (0.0230 to 0.0410) | 0.1290 (0.0920 to 0.1690) | 0.1240 (0.0910 to 0.1560) | 0.0890 (0.0580 to 0.1360) |
| **2007** | 0.0760 (0.0550 to 0.0990) | 0.0000 (0.0000 to 0.0010) | 0.0000 (0.0000 to 0.0000) | 0.0140 (0.0100 to 0.0180) | 0.0310 (0.0230 to 0.0402) | 0.1240 (0.0880 to 0.1620) | 0.1140 (0.0850 to 0.1450) | 0.0850 (0.0560 to 0.1270) |
| **2008** | 0.0720 (0.0520 to 0.0930) | 0.0000 (0.0000 to 0.0010) | 0.0000 (0.0000 to 0.0000) | 0.0130 (0.0090 to 0.0170) | 0.0300 (0.0221 to 0.0390) | 0.1180 (0.0850 to 0.1560) | 0.1050 (0.0780 to 0.1340) | 0.0810 (0.0540 to 0.1190) |
| **2009** | 0.0680 (0.0480 to 0.0880) | 0.0000 (0.0000 to 0.0010) | 0.0000 (0.0000 to 0.0000) | 0.0130 (0.0090 to 0.0160) | 0.0298 (0.0220 to 0.0390) | 0.1140 (0.0820 to 0.1490) | 0.0960 (0.0720 to 0.1240) | 0.0770 (0.0527 to 0.1120) |
| **2010** | 0.0640 (0.0460 to 0.0850) | 0.0000 (0.0000 to 0.0010) | 0.0000 (0.0000 to 0.0000) | 0.0120 (0.0090 to 0.0158) | 0.0292 (0.0212 to 0.0377) | 0.1100 (0.0790 to 0.1430) | 0.0900 (0.0670 to 0.1167) | 0.0740 (0.0510 to 0.1090) |
| **2011** | 0.0620 (0.0450 to 0.0810) | 0.0000 (0.0000 to 0.0010) | 0.0000 (0.0000 to 0.0000) | 0.0120 (0.0090 to 0.0159) | 0.0286 (0.0210 to 0.0370) | 0.1060 (0.0770 to 0.1380) | 0.0850 (0.0630 to 0.1115) | 0.0720 (0.0500 to 0.1070) |
| **2012** | 0.0600 (0.0440 to 0.0770) | 0.0000 (0.0000 to 0.0010) | 0.0000 (0.0000 to 0.0000) | 0.0120 (0.0090 to 0.0160) | 0.0280 (0.0210 to 0.0360) | 0.1018 (0.0740 to 0.1310) | 0.0810 (0.0600 to 0.1070) | 0.0690 (0.0490 to 0.1050) |
| **2013** | 0.0575 (0.0420 to 0.0730) | 0.0000 (0.0000 to 0.0010) | 0.0000 (0.0000 to 0.0000) | 0.0123 (0.0090 to 0.0160) | 0.0280 (0.0203 to 0.0358) | 0.0980 (0.0710 to 0.1260) | 0.0780 (0.0578 to 0.1020) | 0.0670 (0.0470 to 0.1000) |
| **2014** | 0.0560 (0.0408 to 0.0710) | 0.0000 (0.0000 to 0.0010) | 0.0000 (0.0000 to 0.0000) | 0.0120 (0.0090 to 0.0160) | 0.0270 (0.0200 to 0.0350) | 0.0950 (0.0690 to 0.1220) | 0.0740 (0.0550 to 0.0980) | 0.0650 (0.0460 to 0.0950) |
| **2015** | 0.0540 (0.0390 to 0.0690) | 0.0000 (0.0000 to 0.0010) | 0.0000 (0.0000 to 0.0000) | 0.0120 (0.0090 to 0.0160) | 0.0270 (0.0190 to 0.0350) | 0.0930 (0.0680 to 0.1195) | 0.0710 (0.0520 to 0.0940) | 0.0630 (0.0437 to 0.0920) |
| **2016** | 0.0523 (0.0380 to 0.0670) | 0.0000 (0.0000 to 0.0010) | 0.0000 (0.0000 to 0.0000) | 0.0120 (0.0090 to 0.0160) | 0.0261 (0.0190 to 0.0340) | 0.0920 (0.0670 to 0.1200) | 0.0660 (0.0490 to 0.0880) | 0.0610 (0.0430 to 0.0880) |
| **2017** | 0.0510 (0.0370 to 0.0660) | 0.0000 (0.0000 to 0.0010) | 0.0000 (0.0000 to 0.0000) | 0.0110 (0.0080 to 0.0150) | 0.0260 (0.0180 to 0.0340) | 0.0920 (0.0670 to 0.1200) | 0.0610 (0.0450 to 0.0800) | 0.0600 (0.0420 to 0.0860) |
| **2018** | 0.0490 (0.0360 to 0.0640) | 0.0000 (0.0000 to 0.0010) | 0.0000 (0.0000 to 0.0000) | 0.0110 (0.0080 to 0.0142) | 0.0250 (0.0180 to 0.0330) | 0.0920 (0.0670 to 0.1196) | 0.0570 (0.0420 to 0.0730) | 0.0585 (0.0410 to 0.0830) |
| **2019** | 0.0480 (0.0350 to 0.0620) | 0.0000 (0.0000 to 0.0010) | 0.0000 (0.0000 to 0.0000) | 0.0100 (0.0070 to 0.0130) | 0.0240 (0.0175 to 0.0325) | 0.0910 (0.0670 to 0.1194) | 0.0529 (0.0388 to 0.0680) | 0.0570 (0.0400 to 0.0810) |
| **2020** | 0.0470 (0.0350 to 0.0614) | 0.0000 (0.0000 to 0.0010) | 0.0000 (0.0000 to 0.0000) | 0.0100 (0.0070 to 0.0130) | 0.0241 (0.0170 to 0.0317) | 0.0910 (0.0670 to 0.1190) | 0.0512 (0.0380 to 0.0650) | 0.0560 (0.0390 to 0.0790) |
| **2021** | 0.0450 (0.0330 to 0.0580) | 0.0000 (0.0000 to 0.0010) | 0.0000 (0.0000 to 0.0000) | 0.0080 (0.0060 to 0.0110) | 0.0240 (0.0170 to 0.0310) | 0.0810 (0.0590 to 0.1060) | 0.0520 (0.0380 to 0.0660) | 0.0530 (0.0380 to 0.0750) |
| **2022** | 0.0420 (0.0309 to 0.0547) | 0.0000 (0.0000 to 0.0010) | 0.0000 (0.0000 to 0.0000) | 0.0070 (0.0050 to 0.0090) | 0.0231 (0.0160 to 0.0300) | 0.0720 (0.0516 to 0.0930) | 0.0520 (0.0387 to 0.0680) | 0.0510 (0.0360 to 0.0710) |
| **2023** | 0.0420 (0.0310 to 0.0543) | 0.0000 (0.0000 to 0.0010) | 0.0000 (0.0000 to 0.0000) | 0.0060 (0.0040 to 0.0090) | 0.0230 (0.0160 to 0.0300) | 0.0740 (0.0535 to 0.0960) | 0.0507 (0.0370 to 0.0660) | 0.0490 (0.0350 to 0.0670) |

UI= uncertainty interval; YLD= years lived with disability.

**Table S9.** Age-standardized YLL rate (95% UI) per 100,000 population from 1990 to 2023.

| **year** | **Global** | **High-income** | **Central Europe, Eastern Europe, and Central Asia** | **Latin America and Caribbean** | **North Africa and Middle East** | **South Asia** | **Southeast Asia, East Asia, and Oceania** | **Sub-Saharan Africa** |
| --- | --- | --- | --- | --- | --- | --- | --- | --- |
| **1990** | 386.4229 (278.3304 to 515.6787) | 0.8416 (0.6614 to 1.0077) | 1.0408 (0.7994 to 1.2849) | 40.8395 (33.4366 to 48.9956) | 132.5530 (58.6523 to 225.7895) | 953.1829 (757.5692 to 1206.4421) | 270.0169 (171.1778 to 392.9315) | 515.9000 (241.1826 to 778.7219) |
| **1991** | 340.8438 (244.8947 to 459.8432) | 0.7339 (0.5739 to 0.8818) | 0.9757 (0.7186 to 1.1994) | 35.2708 (29.2481 to 42.1680) | 116.5518 (52.5185 to 194.8068) | 812.7980 (645.1221 to 1020.9893) | 235.4149 (149.5803 to 351.1891) | 488.4697 (237.6449 to 743.1205) |
| **1992** | 309.0312 (227.1726 to 418.5035) | 0.6511 (0.5182 to 0.7863) | 0.9315 (0.6619 to 1.1241) | 30.7985 (25.4886 to 37.5609) | 107.5597 (48.7844 to 181.1807) | 714.7803 (567.3899 to 888.9841) | 208.4526 (132.5680 to 309.4168) | 466.6240 (227.6383 to 723.4543) |
| **1993** | 283.9850 (210.4368 to 379.3630) | 0.5870 (0.4668 to 0.7144) | 0.9020 (0.6077 to 1.0885) | 27.1774 (22.5457 to 33.6945) | 111.7931 (52.0284 to 197.9605) | 638.7184 (511.1807 to 794.0460) | 184.8355 (119.8809 to 284.3286) | 445.1670 (217.6845 to 696.8550) |
| **1994** | 263.7702 (194.2085 to 364.9403) | 0.5215 (0.4182 to 0.6420) | 0.8332 (0.5640 to 1.0082) | 23.9152 (19.8901 to 29.5806) | 119.1493 (54.9178 to 206.7287) | 578.1750 (462.4731 to 713.9080) | 165.1115 (104.2667 to 265.3177) | 426.7037 (219.6567 to 659.3043) |
| **1995** | 243.2529 (175.4485 to 341.2652) | 0.4685 (0.3698 to 0.5749) | 0.7495 (0.5121 to 0.9139) | 20.8663 (17.4130 to 25.7201) | 117.4006 (52.4370 to 197.9416) | 522.5120 (423.3923 to 641.0870) | 146.4890 (89.8745 to 230.6947) | 405.0572 (214.9924 to 628.7112) |
| **1996** | 221.1079 (157.1887 to 308.1784) | 0.4229 (0.3309 to 0.5241) | 0.6340 (0.4559 to 0.7684) | 18.0848 (15.1060 to 22.4623) | 112.0894 (52.4795 to 193.1529) | 463.1713 (364.4029 to 562.8963) | 129.4809 (78.7098 to 199.0074) | 382.6797 (208.0507 to 597.2556) |
| **1997** | 200.9767 (139.7947 to 279.7388) | 0.3818 (0.3003 to 0.4728) | 0.5252 (0.4004 to 0.6291) | 15.6096 (12.9658 to 19.0810) | 106.5642 (51.7901 to 190.6158) | 409.2933 (317.8272 to 505.3100) | 114.9941 (69.6090 to 175.2230) | 363.7754 (194.1463 to 563.5165) |
| **1998** | 185.4234 (128.1744 to 257.3700) | 0.3381 (0.2656 to 0.4170) | 0.4405 (0.3460 to 0.5242) | 13.6169 (11.2291 to 17.1123) | 100.6361 (51.1940 to 179.1700) | 367.0014 (284.0492 to 457.1730) | 103.2002 (62.5430 to 156.9218) | 351.6045 (183.6796 to 556.5102) |
| **1999** | 171.7865 (120.6580 to 238.8866) | 0.2954 (0.2366 to 0.3640) | 0.3916 (0.3161 to 0.4653) | 11.9669 (9.6663 to 15.2941) | 92.7556 (49.8375 to 167.6499) | 332.2128 (255.5354 to 415.7337) | 92.7268 (54.4299 to 146.4645) | 339.4251 (184.7099 to 546.1743) |
| **2000** | 158.6241 (111.8265 to 225.1537) | 0.2549 (0.2041 to 0.3204) | 0.3423 (0.2764 to 0.4082) | 10.3846 (8.2573 to 13.5313) | 84.1596 (46.2776 to 145.5515) | 302.3792 (236.5000 to 384.6947) | 83.1618 (50.3687 to 131.8186) | 323.4055 (177.5755 to 543.0987) |
| **2001** | 145.4561 (106.2776 to 204.8634) | 0.2225 (0.1804 to 0.2817) | 0.3089 (0.2518 to 0.3682) | 9.2163 (7.1835 to 12.2802) | 72.7265 (41.1002 to 122.7070) | 277.1457 (221.9684 to 356.8004) | 75.2240 (44.3270 to 117.2118) | 299.3172 (166.9503 to 512.6471) |
| **2002** | 133.5860 (99.0411 to 187.9398) | 0.1988 (0.1608 to 0.2555) | 0.2734 (0.2221 to 0.3287) | 8.2620 (6.3352 to 11.1870) | 60.3950 (34.6651 to 101.2622) | 254.2990 (202.6892 to 330.3542) | 67.6450 (40.1943 to 107.3749) | 278.6282 (152.3096 to 478.7005) |
| **2003** | 123.3617 (91.2378 to 176.0425) | 0.1758 (0.1411 to 0.2272) | 0.2406 (0.1936 to 0.2952) | 7.4620 (5.6408 to 10.3843) | 55.9073 (32.5345 to 93.9161) | 231.1933 (184.9240 to 293.9050) | 61.1134 (37.0356 to 99.2645) | 262.4956 (137.4582 to 452.1547) |
| **2004** | 113.1245 (83.3117 to 162.0193) | 0.1524 (0.1219 to 0.1988) | 0.1991 (0.1621 to 0.2409) | 6.8233 (5.1025 to 9.7169) | 51.7613 (28.9323 to 87.8640) | 208.7742 (170.0644 to 257.8123) | 55.8216 (33.6785 to 91.7468) | 243.0405 (127.3378 to 417.3752) |
| **2005** | 102.1270 (74.6203 to 146.8902) | 0.1332 (0.1071 to 0.1729) | 0.1806 (0.1450 to 0.2175) | 6.2569 (4.6184 to 9.0056) | 45.0319 (24.5730 to 78.3280) | 186.9119 (148.2515 to 233.3657) | 49.5230 (29.5188 to 84.8873) | 222.1948 (117.0174 to 388.1989) |
| **2006** | 91.9061 (65.9984 to 132.7216) | 0.1173 (0.0950 to 0.1489) | 0.1566 (0.1255 to 0.1901) | 5.6923 (4.1052 to 8.5154) | 39.2858 (21.7345 to 70.1126) | 166.5838 (128.9349 to 206.4111) | 44.0861 (25.7488 to 73.1250) | 204.1227 (107.7723 to 348.3725) |
| **2007** | 82.4508 (57.6230 to 116.9679) | 0.1086 (0.0880 to 0.1344) | 0.1337 (0.1077 to 0.1625) | 5.0405 (3.7258 to 7.6070) | 34.2074 (18.9571 to 59.8570) | 148.1020 (111.8981 to 183.9102) | 39.3919 (22.7817 to 65.9575) | 186.1253 (98.0650 to 308.9508) |
| **2008** | 73.6088 (50.5348 to 104.1993) | 0.1014 (0.0810 to 0.1250) | 0.1129 (0.0930 to 0.1371) | 4.3805 (3.1873 to 6.7205) | 29.0950 (16.0194 to 49.3201) | 130.0264 (96.7470 to 163.5685) | 35.6184 (20.1610 to 58.5279) | 168.9597 (87.9170 to 275.8521) |
| **2009** | 64.3635 (43.0837 to 90.5414) | 0.1000 (0.0790 to 0.1225) | 0.0960 (0.0790 to 0.1196) | 3.8030 (2.7319 to 5.6984) | 25.1324 (14.4423 to 41.9524) | 109.0720 (81.3495 to 141.8646) | 31.6484 (17.6160 to 51.2165) | 153.0135 (81.6404 to 247.4639) |
| **2010** | 56.9590 (37.8365 to 80.6212) | 0.0920 (0.0720 to 0.1118) | 0.0900 (0.0740 to 0.1118) | 3.3869 (2.4196 to 4.9894) | 22.4485 (12.8203 to 37.3658) | 92.3938 (68.9637 to 121.1729) | 28.4951 (15.5719 to 45.0369) | 138.6532 (74.9227 to 222.4881) |
| **2011** | 51.1000 (33.7606 to 71.5860) | 0.0830 (0.0660 to 0.1008) | 0.0910 (0.0760 to 0.1123) | 3.0151 (2.1408 to 4.5278) | 20.1739 (11.3400 to 33.9190) | 79.5966 (58.1093 to 105.7657) | 25.7777 (13.9149 to 39.6018) | 127.1460 (69.0070 to 198.7559) |
| **2012** | 46.1820 (30.5613 to 66.0204) | 0.0770 (0.0605 to 0.0980) | 0.0880 (0.0740 to 0.1087) | 2.7697 (1.9567 to 4.1923) | 17.8682 (10.2104 to 29.3166) | 69.3030 (50.8300 to 92.5695) | 23.0371 (12.6940 to 35.7503) | 118.7490 (66.8855 to 186.6030) |
| **2013** | 42.0714 (27.5348 to 59.7954) | 0.0720 (0.0560 to 0.0940) | 0.0810 (0.0680 to 0.1002) | 2.5454 (1.8067 to 4.0913) | 15.5638 (8.8785 to 25.3006) | 61.0884 (43.5232 to 83.5360) | 20.8869 (11.7990 to 32.4302) | 110.8945 (63.7981 to 178.2967) |
| **2014** | 38.4244 (25.2006 to 56.8237) | 0.0680 (0.0520 to 0.0900) | 0.0790 (0.0660 to 0.0960) | 2.3899 (1.6926 to 3.8462) | 13.5896 (7.8247 to 22.0492) | 54.1043 (37.0400 to 76.7240) | 19.1036 (11.0129 to 30.6536) | 103.1545 (60.3023 to 167.6280) |
| **2015** | 34.9535 (22.8105 to 52.4703) | 0.0660 (0.0500 to 0.0880) | 0.0810 (0.0680 to 0.0980) | 2.2564 (1.5935 to 3.6190) | 12.2964 (7.2970 to 20.2854) | 48.0843 (31.9412 to 70.6516) | 17.6223 (10.2873 to 27.9502) | 95.0506 (56.4460 to 156.1976) |
| **2016** | 31.7009 (20.4892 to 48.8693) | 0.0640 (0.0481 to 0.0880) | 0.0740 (0.0630 to 0.0920) | 2.1643 (1.5031 to 3.4915) | 11.2499 (6.6644 to 18.6706) | 43.1543 (27.7154 to 65.9294) | 15.8299 (9.6340 to 24.9839) | 86.9464 (51.7348 to 145.0586) |
| **2017** | 29.1186 (18.8664 to 45.1102) | 0.0608 (0.0463 to 0.0830) | 0.0640 (0.0540 to 0.0810) | 2.0621 (1.4421 to 3.3838) | 10.4455 (6.0233 to 17.3319) | 39.2352 (24.7391 to 61.0110) | 14.6421 (8.9877 to 23.5167) | 79.3434 (47.3860 to 133.9306) |
| **2018** | 26.7044 (17.1206 to 40.7467) | 0.0590 (0.0451 to 0.0800) | 0.0620 (0.0500 to 0.0790) | 1.9656 (1.3546 to 3.2114) | 9.9794 (5.6212 to 17.2681) | 35.1254 (21.8005 to 55.3897) | 13.6196 (8.1662 to 22.2924) | 72.2449 (42.4229 to 122.1379) |
| **2019** | 24.6639 (15.3182 to 36.7226) | 0.0560 (0.0428 to 0.0760) | 0.0579 (0.0450 to 0.0720) | 1.8327 (1.2564 to 2.9503) | 9.4757 (5.2401 to 16.9760) | 31.7338 (19.7337 to 50.0586) | 12.8422 (7.4736 to 20.7475) | 65.7891 (38.0370 to 108.9180) |
| **2020** | 23.5637 (14.3783 to 35.7389) | 0.0560 (0.0420 to 0.0750) | 0.0570 (0.0440 to 0.0710) | 1.7985 (1.2164 to 2.8989) | 9.0471 (4.8363 to 16.6245) | 30.1015 (18.3103 to 46.3319) | 12.6638 (6.9700 to 20.3034) | 61.0586 (34.8371 to 102.9121) |
| **2021** | 22.0287 (13.3783 to 34.2996) | 0.0560 (0.0410 to 0.0740) | 0.0520 (0.0380 to 0.0660) | 1.7459 (1.1785 to 2.8407) | 8.4842 (4.4691 to 15.8617) | 26.7761 (16.1688 to 41.7617) | 13.1610 (7.3952 to 21.6560) | 56.1649 (31.5931 to 95.3159) |
| **2022** | 19.9937 (12.1738 to 30.8687) | 0.0555 (0.0400 to 0.0730) | 0.0500 (0.0380 to 0.0650) | 1.6861 (1.1215 to 2.6725) | 7.5915 (3.9216 to 13.8808) | 23.5073 (13.7393 to 37.0682) | 12.3086 (7.1284 to 20.1610) | 51.0199 (28.8509 to 87.4735) |
| **2023** | 17.8414 (10.8165 to 27.3394) | 0.0533 (0.0370 to 0.0690) | 0.0470 (0.0350 to 0.0610) | 1.6273 (1.0688 to 2.5488) | 6.4211 (3.3625 to 11.7603) | 20.3933 (11.9254 to 32.4223) | 11.5368 (6.5664 to 19.1004) | 46.0249 (25.6544 to 77.1038) |

UI= uncertainty interval; YLL= years of life lost.

**Table S10.** SDI and age-standardized prevalence, incidence, DALY, and mortality rate (95% UI) per 100,000 population from 1990 to 2023.

|  | year | Age-standardized prevalence rate | Age-standardized  incidence rate | Age-standardized  DALY rate | Age-standardized  mortality rate | SDI |
| --- | --- | --- | --- | --- | --- | --- |
| Global | 1990 | 2.50 (1.91 to 3.12) | 11.32 (7.98 to 16.36) | 386.60 (278.47 to 515.89) | 4.94 (3.59 to 6.38) | 0.532 |
| Global | 1991 | 2.44 (1.84 to 3.04) | 10.23 (7.24 to 14.58) | 341.01 (245.06 to 460.05) | 4.36 (3.20 to 5.69) | 0.536 |
| Global | 1992 | 2.37 (1.78 to 2.94) | 9.25 (6.72 to 12.94) | 309.19 (227.32 to 418.65) | 3.96 (2.93 to 5.23) | 0.540 |
| Global | 1993 | 2.29 (1.72 to 2.85) | 8.37 (6.01 to 11.76) | 284.14 (210.58 to 379.55) | 3.64 (2.70 to 4.84) | 0.544 |
| Global | 1994 | 2.21 (1.65 to 2.75) | 7.61 (5.39 to 10.75) | 263.91 (194.35 to 365.11) | 3.38 (2.49 to 4.60) | 0.548 |
| Global | 1995 | 2.14 (1.59 to 2.66) | 7.00 (4.98 to 9.99) | 243.39 (175.58 to 341.44) | 3.12 (2.28 to 4.27) | 0.552 |
| Global | 1996 | 2.04 (1.51 to 2.55) | 6.46 (4.64 to 9.06) | 221.24 (157.32 to 308.33) | 2.85 (2.02 to 3.88) | 0.556 |
| Global | 1997 | 1.90 (1.40 to 2.38) | 5.94 (4.24 to 8.31) | 201.10 (139.92 to 279.86) | 2.61 (1.85 to 3.56) | 0.561 |
| Global | 1998 | 1.75 (1.29 to 2.18) | 5.48 (3.95 to 7.59) | 185.54 (128.28 to 257.50) | 2.42 (1.73 to 3.34) | 0.565 |
| Global | 1999 | 1.60 (1.18 to 2.00) | 5.06 (3.65 to 7.07) | 171.90 (120.77 to 239.00) | 2.25 (1.59 to 3.12) | 0.569 |
| Global | 2000 | 1.48 (1.10 to 1.86) | 4.71 (3.40 to 6.72) | 158.73 (111.92 to 225.27) | 2.09 (1.51 to 2.96) | 0.573 |
| Global | 2001 | 1.38 (1.04 to 1.73) | 4.37 (3.20 to 6.36) | 145.56 (106.38 to 204.97) | 1.93 (1.41 to 2.74) | 0.577 |
| Global | 2002 | 1.29 (0.98 to 1.60) | 4.04 (2.93 to 5.99) | 133.68 (99.13 to 188.04) | 1.78 (1.31 to 2.50) | 0.581 |
| Global | 2003 | 1.20 (0.91 to 1.49) | 3.72 (2.69 to 5.61) | 123.45 (91.31 to 176.15) | 1.65 (1.21 to 2.32) | 0.585 |
| Global | 2004 | 1.12 (0.84 to 1.39) | 3.42 (2.42 to 5.22) | 113.21 (83.38 to 162.11) | 1.52 (1.12 to 2.15) | 0.590 |
| Global | 2005 | 1.05 (0.79 to 1.32) | 3.15 (2.18 to 4.94) | 102.21 (74.69 to 147.01) | 1.38 (1.02 to 1.99) | 0.594 |
| Global | 2006 | 0.99 (0.75 to 1.24) | 2.90 (1.98 to 4.47) | 91.99 (66.06 to 132.83) | 1.25 (0.91 to 1.79) | 0.599 |
| Global | 2007 | 0.94 (0.70 to 1.17) | 2.64 (1.78 to 4.04) | 82.53 (57.69 to 117.05) | 1.13 (0.81 to 1.59) | 0.604 |
| Global | 2008 | 0.88 (0.66 to 1.10) | 2.37 (1.61 to 3.80) | 73.68 (50.60 to 104.28) | 1.01 (0.71 to 1.41) | 0.608 |
| Global | 2009 | 0.83 (0.63 to 1.05) | 2.14 (1.43 to 3.48) | 64.43 (43.15 to 90.60) | 0.90 (0.61 to 1.23) | 0.613 |
| Global | 2010 | 0.79 (0.60 to 1.00) | 1.96 (1.26 to 3.12) | 57.02 (37.89 to 80.68) | 0.80 (0.54 to 1.12) | 0.618 |
| Global | 2011 | 0.76 (0.57 to 0.96) | 1.80 (1.17 to 2.78) | 51.16 (33.82 to 71.65) | 0.72 (0.48 to 1.01) | 0.623 |
| Global | 2012 | 0.73 (0.55 to 0.91) | 1.64 (1.09 to 2.60) | 46.24 (30.62 to 66.08) | 0.65 (0.44 to 0.91) | 0.627 |
| Global | 2013 | 0.70 (0.52 to 0.88) | 1.49 (0.97 to 2.44) | 42.13 (27.59 to 59.86) | 0.60 (0.40 to 0.85) | 0.631 |
| Global | 2014 | 0.67 (0.50 to 0.85) | 1.37 (0.88 to 2.29) | 38.48 (25.24 to 56.89) | 0.55 (0.37 to 0.80) | 0.635 |
| Global | 2015 | 0.64 (0.48 to 0.81) | 1.26 (0.79 to 2.13) | 35.01 (22.87 to 52.52) | 0.50 (0.33 to 0.76) | 0.639 |
| Global | 2016 | 0.61 (0.46 to 0.78) | 1.17 (0.73 to 1.97) | 31.75 (20.53 to 48.92) | 0.46 (0.30 to 0.71) | 0.644 |
| Global | 2017 | 0.58 (0.43 to 0.74) | 1.08 (0.67 to 1.83) | 29.17 (18.92 to 45.17) | 0.42 (0.28 to 0.67) | 0.649 |
| Global | 2018 | 0.55 (0.41 to 0.70) | 1.00 (0.61 to 1.68) | 26.75 (17.17 to 40.81) | 0.39 (0.25 to 0.61) | 0.655 |
| Global | 2019 | 0.52 (0.39 to 0.66) | 0.93 (0.55 to 1.63) | 24.71 (15.36 to 36.78) | 0.36 (0.22 to 0.56) | 0.661 |
| Global | 2020 | 0.51 (0.38 to 0.65) | 0.88 (0.50 to 1.62) | 23.61 (14.42 to 35.78) | 0.34 (0.21 to 0.53) | 0.666 |
| Global | 2021 | 0.49 (0.36 to 0.63) | 0.82 (0.48 to 1.49) | 22.07 (13.41 to 34.35) | 0.32 (0.20 to 0.49) | 0.671 |
| Global | 2022 | 0.49 (0.35 to 0.63) | 0.76 (0.44 to 1.37) | 20.04 (12.21 to 30.92) | 0.29 (0.18 to 0.46) | 0.676 |
| Global | 2023 | 0.49 (0.35 to 0.63) | 0.70 (0.40 to 1.22) | 17.88 (10.86 to 27.38) | 0.27 (0.16 to 0.41) | 0.680 |
| Andean Latin America | 1990 | 0.35 (0.27 to 0.45) | 2.41 (1.59 to 3.59) | 46.47 (30.35 to 65.21) | 0.93 (0.62 to 1.32) | 0.507 |
| Andean Latin America | 1991 | 0.34 (0.26 to 0.43) | 2.21 (1.44 to 3.19) | 42.42 (27.68 to 60.34) | 0.87 (0.57 to 1.22) | 0.508 |
| Andean Latin America | 1992 | 0.32 (0.25 to 0.41) | 2.01 (1.29 to 2.88) | 37.98 (24.95 to 53.56) | 0.79 (0.52 to 1.10) | 0.510 |
| Andean Latin America | 1993 | 0.31 (0.24 to 0.40) | 1.82 (1.16 to 2.57) | 33.69 (22.73 to 46.49) | 0.71 (0.49 to 0.99) | 0.513 |
| Andean Latin America | 1994 | 0.30 (0.23 to 0.38) | 1.65 (1.06 to 2.41) | 29.30 (20.31 to 40.89) | 0.62 (0.42 to 0.88) | 0.517 |
| Andean Latin America | 1995 | 0.29 (0.22 to 0.37) | 1.49 (0.97 to 2.28) | 25.70 (17.55 to 35.93) | 0.55 (0.37 to 0.78) | 0.522 |
| Andean Latin America | 1996 | 0.28 (0.21 to 0.35) | 1.33 (0.87 to 1.96) | 22.67 (15.38 to 32.29) | 0.49 (0.34 to 0.69) | 0.526 |
| Andean Latin America | 1997 | 0.26 (0.20 to 0.33) | 1.16 (0.77 to 1.70) | 19.41 (13.15 to 27.50) | 0.42 (0.30 to 0.60) | 0.531 |
| Andean Latin America | 1998 | 0.24 (0.18 to 0.31) | 0.99 (0.67 to 1.42) | 16.72 (11.40 to 23.09) | 0.37 (0.26 to 0.51) | 0.536 |
| Andean Latin America | 1999 | 0.22 (0.17 to 0.28) | 0.85 (0.59 to 1.22) | 14.14 (9.63 to 19.34) | 0.31 (0.22 to 0.44) | 0.541 |
| Andean Latin America | 2000 | 0.21 (0.16 to 0.27) | 0.75 (0.52 to 1.10) | 11.91 (8.22 to 16.44) | 0.27 (0.19 to 0.37) | 0.546 |
| Andean Latin America | 2001 | 0.20 (0.15 to 0.25) | 0.67 (0.46 to 1.00) | 10.17 (7.03 to 14.02) | 0.23 (0.16 to 0.32) | 0.552 |
| Andean Latin America | 2002 | 0.19 (0.15 to 0.24) | 0.60 (0.40 to 0.90) | 8.82 (5.84 to 12.47) | 0.20 (0.13 to 0.28) | 0.558 |
| Andean Latin America | 2003 | 0.18 (0.14 to 0.22) | 0.54 (0.36 to 0.82) | 8.01 (5.40 to 11.48) | 0.18 (0.12 to 0.26) | 0.563 |
| Andean Latin America | 2004 | 0.17 (0.13 to 0.21) | 0.49 (0.32 to 0.77) | 7.31 (5.07 to 10.58) | 0.17 (0.12 to 0.25) | 0.569 |
| Andean Latin America | 2005 | 0.16 (0.13 to 0.20) | 0.44 (0.29 to 0.74) | 6.58 (4.55 to 9.76) | 0.15 (0.10 to 0.23) | 0.574 |
| Andean Latin America | 2006 | 0.16 (0.12 to 0.20) | 0.40 (0.26 to 0.68) | 5.77 (3.93 to 8.83) | 0.13 (0.09 to 0.20) | 0.580 |
| Andean Latin America | 2007 | 0.15 (0.12 to 0.19) | 0.36 (0.24 to 0.63) | 5.16 (3.49 to 7.76) | 0.12 (0.08 to 0.18) | 0.586 |
| Andean Latin America | 2008 | 0.14 (0.11 to 0.18) | 0.33 (0.21 to 0.58) | 4.68 (3.15 to 7.01) | 0.11 (0.07 to 0.16) | 0.592 |
| Andean Latin America | 2009 | 0.14 (0.11 to 0.18) | 0.29 (0.18 to 0.53) | 4.21 (2.79 to 6.44) | 0.10 (0.06 to 0.15) | 0.598 |
| Andean Latin America | 2010 | 0.13 (0.11 to 0.17) | 0.27 (0.16 to 0.49) | 3.69 (2.34 to 5.71) | 0.09 (0.05 to 0.14) | 0.605 |
| Andean Latin America | 2011 | 0.13 (0.10 to 0.17) | 0.24 (0.15 to 0.43) | 3.16 (1.94 to 4.95) | 0.07 (0.04 to 0.12) | 0.612 |
| Andean Latin America | 2012 | 0.13 (0.10 to 0.16) | 0.21 (0.13 to 0.38) | 2.72 (1.62 to 4.36) | 0.06 (0.04 to 0.10) | 0.619 |
| Andean Latin America | 2013 | 0.12 (0.10 to 0.16) | 0.19 (0.11 to 0.33) | 2.46 (1.45 to 3.93) | 0.06 (0.03 to 0.09) | 0.626 |
| Andean Latin America | 2014 | 0.12 (0.09 to 0.16) | 0.17 (0.10 to 0.29) | 2.22 (1.31 to 3.51) | 0.05 (0.03 to 0.08) | 0.632 |
| Andean Latin America | 2015 | 0.12 (0.09 to 0.15) | 0.15 (0.09 to 0.28) | 2.06 (1.23 to 3.28) | 0.05 (0.03 to 0.08) | 0.638 |
| Andean Latin America | 2016 | 0.11 (0.09 to 0.15) | 0.14 (0.08 to 0.26) | 1.92 (1.18 to 3.04) | 0.05 (0.03 to 0.07) | 0.643 |
| Andean Latin America | 2017 | 0.11 (0.09 to 0.14) | 0.13 (0.07 to 0.24) | 1.81 (1.11 to 2.89) | 0.04 (0.03 to 0.07) | 0.648 |
| Andean Latin America | 2018 | 0.11 (0.08 to 0.14) | 0.12 (0.07 to 0.23) | 1.68 (1.02 to 2.72) | 0.04 (0.02 to 0.07) | 0.654 |
| Andean Latin America | 2019 | 0.10 (0.08 to 0.13) | 0.11 (0.06 to 0.22) | 1.45 (0.86 to 2.43) | 0.03 (0.02 to 0.06) | 0.660 |
| Andean Latin America | 2020 | 0.10 (0.08 to 0.13) | 0.11 (0.06 to 0.23) | 1.52 (0.83 to 2.59) | 0.04 (0.02 to 0.06) | 0.665 |
| Andean Latin America | 2021 | 0.10 (0.08 to 0.14) | 0.11 (0.06 to 0.23) | 1.52 (0.82 to 2.60) | 0.04 (0.02 to 0.06) | 0.670 |
| Andean Latin America | 2022 | 0.10 (0.08 to 0.14) | 0.12 (0.06 to 0.26) | 1.48 (0.84 to 2.60) | 0.04 (0.02 to 0.06) | 0.674 |
| Andean Latin America | 2023 | 0.10 (0.08 to 0.14) | 0.11 (0.05 to 0.25) | 1.49 (0.84 to 2.69) | 0.04 (0.02 to 0.07) | 0.679 |
| Australasia | 1990 | 0.00 (0.00 to 0.01) | 0.06 (0.03 to 0.12) | 0.07 (0.04 to 0.09) | 0.00 (0.00 to 0.01) | 0.734 |
| Australasia | 1991 | 0.00 (0.00 to 0.01) | 0.05 (0.03 to 0.11) | 0.06 (0.03 to 0.08) | 0.00 (0.00 to 0.01) | 0.738 |
| Australasia | 1992 | 0.00 (0.00 to 0.01) | 0.04 (0.02 to 0.10) | 0.06 (0.03 to 0.08) | 0.00 (0.00 to 0.00) | 0.742 |
| Australasia | 1993 | 0.00 (0.00 to 0.01) | 0.04 (0.02 to 0.09) | 0.04 (0.02 to 0.07) | 0.00 (0.00 to 0.00) | 0.746 |
| Australasia | 1994 | 0.00 (0.00 to 0.01) | 0.03 (0.02 to 0.09) | 0.04 (0.02 to 0.07) | 0.00 (0.00 to 0.00) | 0.750 |
| Australasia | 1995 | 0.00 (0.00 to 0.01) | 0.03 (0.02 to 0.08) | 0.03 (0.02 to 0.06) | 0.00 (0.00 to 0.00) | 0.754 |
| Australasia | 1996 | 0.00 (0.00 to 0.00) | 0.03 (0.01 to 0.08) | 0.03 (0.02 to 0.04) | 0.00 (0.00 to 0.00) | 0.758 |
| Australasia | 1997 | 0.00 (0.00 to 0.00) | 0.03 (0.01 to 0.07) | 0.03 (0.02 to 0.05) | 0.00 (0.00 to 0.00) | 0.763 |
| Australasia | 1998 | 0.00 (0.00 to 0.00) | 0.03 (0.01 to 0.07) | 0.03 (0.01 to 0.05) | 0.00 (0.00 to 0.00) | 0.767 |
| Australasia | 1999 | 0.00 (0.00 to 0.00) | 0.03 (0.01 to 0.07) | 0.03 (0.01 to 0.05) | 0.00 (0.00 to 0.00) | 0.771 |
| Australasia | 2000 | 0.00 (0.00 to 0.00) | 0.03 (0.01 to 0.07) | 0.03 (0.01 to 0.04) | 0.00 (0.00 to 0.00) | 0.775 |
| Australasia | 2001 | 0.00 (0.00 to 0.00) | 0.02 (0.01 to 0.06) | 0.02 (0.01 to 0.03) | 0.00 (0.00 to 0.00) | 0.779 |
| Australasia | 2002 | 0.00 (0.00 to 0.00) | 0.02 (0.01 to 0.06) | 0.01 (0.01 to 0.02) | 0.00 (0.00 to 0.00) | 0.784 |
| Australasia | 2003 | 0.00 (0.00 to 0.00) | 0.02 (0.01 to 0.05) | 0.01 (0.01 to 0.02) | 0.00 (0.00 to 0.00) | 0.788 |
| Australasia | 2004 | 0.00 (0.00 to 0.00) | 0.02 (0.01 to 0.05) | 0.01 (0.01 to 0.02) | 0.00 (0.00 to 0.00) | 0.791 |
| Australasia | 2005 | 0.00 (0.00 to 0.00) | 0.02 (0.01 to 0.04) | 0.02 (0.01 to 0.03) | 0.00 (0.00 to 0.00) | 0.794 |
| Australasia | 2006 | 0.00 (0.00 to 0.00) | 0.02 (0.01 to 0.04) | 0.02 (0.01 to 0.03) | 0.00 (0.00 to 0.00) | 0.795 |
| Australasia | 2007 | 0.00 (0.00 to 0.00) | 0.02 (0.01 to 0.04) | 0.03 (0.01 to 0.04) | 0.00 (0.00 to 0.00) | 0.797 |
| Australasia | 2008 | 0.00 (0.00 to 0.00) | 0.03 (0.01 to 0.05) | 0.03 (0.01 to 0.05) | 0.00 (0.00 to 0.00) | 0.799 |
| Australasia | 2009 | 0.00 (0.00 to 0.00) | 0.03 (0.01 to 0.05) | 0.03 (0.01 to 0.05) | 0.00 (0.00 to 0.00) | 0.803 |
| Australasia | 2010 | 0.00 (0.00 to 0.00) | 0.03 (0.01 to 0.06) | 0.03 (0.01 to 0.05) | 0.00 (0.00 to 0.00) | 0.807 |
| Australasia | 2011 | 0.00 (0.00 to 0.00) | 0.03 (0.01 to 0.05) | 0.03 (0.01 to 0.05) | 0.00 (0.00 to 0.00) | 0.811 |
| Australasia | 2012 | 0.00 (0.00 to 0.00) | 0.02 (0.01 to 0.04) | 0.03 (0.01 to 0.04) | 0.00 (0.00 to 0.00) | 0.815 |
| Australasia | 2013 | 0.00 (0.00 to 0.00) | 0.02 (0.00 to 0.03) | 0.02 (0.00 to 0.04) | 0.00 (0.00 to 0.00) | 0.820 |
| Australasia | 2014 | 0.00 (0.00 to 0.00) | 0.01 (0.00 to 0.03) | 0.02 (0.00 to 0.03) | 0.00 (0.00 to 0.00) | 0.824 |
| Australasia | 2015 | 0.00 (0.00 to 0.00) | 0.01 (0.00 to 0.03) | 0.01 (0.00 to 0.02) | 0.00 (0.00 to 0.00) | 0.828 |
| Australasia | 2016 | 0.00 (0.00 to 0.00) | 0.01 (0.00 to 0.02) | 0.01 (0.00 to 0.02) | 0.00 (0.00 to 0.00) | 0.832 |
| Australasia | 2017 | 0.00 (0.00 to 0.00) | 0.01 (0.00 to 0.02) | 0.01 (0.00 to 0.02) | 0.00 (0.00 to 0.00) | 0.836 |
| Australasia | 2018 | 0.00 (0.00 to 0.00) | 0.01 (0.00 to 0.02) | 0.02 (0.00 to 0.03) | 0.00 (0.00 to 0.00) | 0.839 |
| Australasia | 2019 | 0.00 (0.00 to 0.00) | 0.01 (0.00 to 0.02) | 0.01 (0.00 to 0.02) | 0.00 (0.00 to 0.00) | 0.844 |
| Australasia | 2020 | 0.00 (0.00 to 0.00) | 0.01 (0.00 to 0.02) | 0.01 (0.00 to 0.02) | 0.00 (0.00 to 0.00) | 0.847 |
| Australasia | 2021 | 0.00 (0.00 to 0.00) | 0.01 (0.00 to 0.02) | 0.01 (0.00 to 0.01) | 0.00 (0.00 to 0.00) | 0.850 |
| Australasia | 2022 | 0.00 (0.00 to 0.00) | 0.01 (0.00 to 0.01) | 0.00 (0.00 to 0.01) | 0.00 (0.00 to 0.00) | 0.853 |
| Australasia | 2023 | 0.00 (0.00 to 0.00) | 0.00 (0.00 to 0.01) | 0.00 (0.00 to 0.01) | 0.00 (0.00 to 0.00) | 0.856 |
| Caribbean | 1990 | 0.52 (0.36 to 0.72) | 4.91 (2.64 to 8.66) | 88.52 (42.93 to 158.46) | 1.16 (0.63 to 1.90) | 0.534 |
| Caribbean | 1991 | 0.52 (0.37 to 0.70) | 4.69 (2.70 to 7.81) | 81.00 (39.08 to 141.87) | 1.06 (0.58 to 1.72) | 0.538 |
| Caribbean | 1992 | 0.51 (0.37 to 0.69) | 4.46 (2.70 to 6.98) | 76.72 (38.04 to 131.73) | 1.00 (0.56 to 1.59) | 0.542 |
| Caribbean | 1993 | 0.51 (0.37 to 0.67) | 4.22 (2.58 to 6.78) | 73.53 (37.42 to 128.87) | 0.96 (0.55 to 1.55) | 0.545 |
| Caribbean | 1994 | 0.50 (0.37 to 0.67) | 3.99 (2.46 to 6.20) | 70.47 (36.30 to 118.86) | 0.92 (0.53 to 1.48) | 0.548 |
| Caribbean | 1995 | 0.49 (0.36 to 0.67) | 3.77 (2.24 to 6.01) | 65.59 (34.43 to 112.88) | 0.86 (0.50 to 1.36) | 0.551 |
| Caribbean | 1996 | 0.48 (0.36 to 0.64) | 3.54 (2.16 to 5.55) | 59.11 (31.35 to 98.25) | 0.78 (0.47 to 1.21) | 0.555 |
| Caribbean | 1997 | 0.46 (0.34 to 0.61) | 3.27 (2.03 to 5.12) | 53.60 (28.14 to 88.95) | 0.71 (0.43 to 1.10) | 0.558 |
| Caribbean | 1998 | 0.45 (0.33 to 0.59) | 3.02 (1.88 to 4.80) | 48.89 (25.62 to 85.69) | 0.65 (0.39 to 1.05) | 0.562 |
| Caribbean | 1999 | 0.43 (0.31 to 0.57) | 2.79 (1.71 to 4.58) | 45.69 (24.70 to 79.69) | 0.61 (0.37 to 0.99) | 0.567 |
| Caribbean | 2000 | 0.42 (0.30 to 0.56) | 2.60 (1.49 to 4.47) | 41.82 (21.54 to 80.90) | 0.55 (0.33 to 0.97) | 0.572 |
| Caribbean | 2001 | 0.41 (0.30 to 0.54) | 2.44 (1.42 to 4.16) | 38.59 (18.70 to 75.96) | 0.51 (0.29 to 0.90) | 0.578 |
| Caribbean | 2002 | 0.40 (0.29 to 0.52) | 2.30 (1.32 to 4.06) | 35.40 (17.25 to 64.66) | 0.47 (0.26 to 0.84) | 0.584 |
| Caribbean | 2003 | 0.39 (0.28 to 0.51) | 2.17 (1.19 to 3.87) | 32.86 (15.24 to 67.08) | 0.44 (0.23 to 0.79) | 0.590 |
| Caribbean | 2004 | 0.38 (0.27 to 0.50) | 2.04 (1.09 to 3.74) | 31.56 (15.57 to 64.50) | 0.42 (0.22 to 0.78) | 0.595 |
| Caribbean | 2005 | 0.36 (0.26 to 0.50) | 1.91 (0.97 to 3.71) | 30.51 (15.15 to 63.37) | 0.40 (0.22 to 0.76) | 0.600 |
| Caribbean | 2006 | 0.35 (0.25 to 0.48) | 1.76 (0.91 to 3.47) | 28.81 (13.75 to 61.26) | 0.38 (0.21 to 0.73) | 0.605 |
| Caribbean | 2007 | 0.34 (0.25 to 0.46) | 1.57 (0.86 to 3.10) | 25.47 (11.82 to 57.00) | 0.34 (0.18 to 0.67) | 0.608 |
| Caribbean | 2008 | 0.32 (0.24 to 0.44) | 1.39 (0.74 to 2.77) | 21.64 (9.72 to 48.68) | 0.29 (0.16 to 0.58) | 0.612 |
| Caribbean | 2009 | 0.31 (0.23 to 0.42) | 1.24 (0.62 to 2.54) | 18.17 (7.97 to 40.23) | 0.25 (0.13 to 0.50) | 0.615 |
| Caribbean | 2010 | 0.30 (0.22 to 0.42) | 1.13 (0.54 to 2.47) | 16.14 (7.05 to 35.33) | 0.22 (0.12 to 0.43) | 0.618 |
| Caribbean | 2011 | 0.30 (0.22 to 0.40) | 1.06 (0.52 to 2.47) | 14.81 (6.31 to 33.28) | 0.21 (0.10 to 0.40) | 0.623 |
| Caribbean | 2012 | 0.29 (0.21 to 0.39) | 1.02 (0.49 to 2.39) | 14.07 (6.04 to 30.72) | 0.20 (0.10 to 0.38) | 0.626 |
| Caribbean | 2013 | 0.29 (0.21 to 0.40) | 0.98 (0.47 to 2.27) | 13.24 (5.72 to 31.00) | 0.19 (0.10 to 0.38) | 0.630 |
| Caribbean | 2014 | 0.28 (0.21 to 0.39) | 0.95 (0.45 to 2.11) | 12.70 (5.53 to 30.11) | 0.18 (0.09 to 0.37) | 0.634 |
| Caribbean | 2015 | 0.28 (0.21 to 0.39) | 0.93 (0.44 to 2.04) | 12.12 (5.20 to 29.48) | 0.18 (0.09 to 0.36) | 0.638 |
| Caribbean | 2016 | 0.28 (0.21 to 0.39) | 0.90 (0.43 to 1.90) | 11.68 (4.90 to 28.10) | 0.17 (0.09 to 0.35) | 0.642 |
| Caribbean | 2017 | 0.28 (0.21 to 0.38) | 0.88 (0.42 to 1.82) | 11.26 (4.86 to 26.44) | 0.17 (0.08 to 0.34) | 0.645 |
| Caribbean | 2018 | 0.29 (0.21 to 0.38) | 0.86 (0.40 to 1.81) | 10.86 (4.82 to 26.34) | 0.16 (0.08 to 0.32) | 0.648 |
| Caribbean | 2019 | 0.29 (0.21 to 0.39) | 0.84 (0.39 to 1.76) | 10.18 (4.54 to 24.01) | 0.15 (0.08 to 0.29) | 0.651 |
| Caribbean | 2020 | 0.29 (0.21 to 0.39) | 0.81 (0.37 to 1.69) | 10.12 (4.48 to 23.25) | 0.15 (0.08 to 0.28) | 0.654 |
| Caribbean | 2021 | 0.29 (0.21 to 0.38) | 0.78 (0.36 to 1.62) | 9.83 (4.08 to 22.88) | 0.15 (0.07 to 0.28) | 0.657 |
| Caribbean | 2022 | 0.28 (0.21 to 0.38) | 0.74 (0.34 to 1.57) | 9.46 (3.91 to 20.34) | 0.14 (0.07 to 0.25) | 0.660 |
| Caribbean | 2023 | 0.28 (0.21 to 0.37) | 0.69 (0.31 to 1.47) | 8.90 (3.61 to 19.48) | 0.13 (0.06 to 0.24) | 0.664 |
| Central Asia | 1990 | 0.00 (0.00 to 0.01) | 0.05 (0.02 to 0.10) | 0.75 (0.26 to 1.02) | 0.02 (0.01 to 0.03) | 0.560 |
| Central Asia | 1991 | 0.00 (0.00 to 0.01) | 0.05 (0.02 to 0.10) | 0.73 (0.25 to 0.98) | 0.02 (0.01 to 0.03) | 0.561 |
| Central Asia | 1992 | 0.00 (0.00 to 0.01) | 0.05 (0.02 to 0.10) | 0.71 (0.24 to 0.94) | 0.02 (0.01 to 0.03) | 0.563 |
| Central Asia | 1993 | 0.00 (0.00 to 0.01) | 0.05 (0.02 to 0.10) | 0.71 (0.24 to 0.93) | 0.02 (0.01 to 0.03) | 0.566 |
| Central Asia | 1994 | 0.00 (0.00 to 0.01) | 0.05 (0.02 to 0.09) | 0.68 (0.23 to 0.90) | 0.02 (0.01 to 0.03) | 0.568 |
| Central Asia | 1995 | 0.00 (0.00 to 0.01) | 0.05 (0.02 to 0.09) | 0.64 (0.21 to 0.86) | 0.02 (0.01 to 0.03) | 0.571 |
| Central Asia | 1996 | 0.00 (0.00 to 0.01) | 0.04 (0.02 to 0.09) | 0.52 (0.19 to 0.68) | 0.02 (0.01 to 0.02) | 0.573 |
| Central Asia | 1997 | 0.00 (0.00 to 0.01) | 0.04 (0.02 to 0.07) | 0.38 (0.17 to 0.51) | 0.01 (0.00 to 0.02) | 0.576 |
| Central Asia | 1998 | 0.00 (0.00 to 0.00) | 0.03 (0.01 to 0.06) | 0.29 (0.15 to 0.39) | 0.01 (0.00 to 0.01) | 0.578 |
| Central Asia | 1999 | 0.00 (0.00 to 0.00) | 0.02 (0.01 to 0.05) | 0.20 (0.12 to 0.28) | 0.01 (0.00 to 0.01) | 0.582 |
| Central Asia | 2000 | 0.00 (0.00 to 0.00) | 0.01 (0.01 to 0.04) | 0.17 (0.11 to 0.23) | 0.00 (0.00 to 0.01) | 0.586 |
| Central Asia | 2001 | 0.00 (0.00 to 0.00) | 0.01 (0.01 to 0.04) | 0.12 (0.08 to 0.16) | 0.00 (0.00 to 0.00) | 0.590 |
| Central Asia | 2002 | 0.00 (0.00 to 0.00) | 0.01 (0.01 to 0.03) | 0.09 (0.07 to 0.12) | 0.00 (0.00 to 0.00) | 0.595 |
| Central Asia | 2003 | 0.00 (0.00 to 0.00) | 0.01 (0.00 to 0.03) | 0.08 (0.06 to 0.11) | 0.00 (0.00 to 0.00) | 0.600 |
| Central Asia | 2004 | 0.00 (0.00 to 0.00) | 0.01 (0.00 to 0.02) | 0.06 (0.04 to 0.08) | 0.00 (0.00 to 0.00) | 0.605 |
| Central Asia | 2005 | 0.00 (0.00 to 0.00) | 0.01 (0.00 to 0.02) | 0.06 (0.04 to 0.08) | 0.00 (0.00 to 0.00) | 0.611 |
| Central Asia | 2006 | 0.00 (0.00 to 0.00) | 0.01 (0.00 to 0.02) | 0.05 (0.04 to 0.07) | 0.00 (0.00 to 0.00) | 0.616 |
| Central Asia | 2007 | 0.00 (0.00 to 0.00) | 0.01 (0.00 to 0.02) | 0.05 (0.03 to 0.07) | 0.00 (0.00 to 0.00) | 0.622 |
| Central Asia | 2008 | 0.00 (0.00 to 0.00) | 0.00 (0.00 to 0.02) | 0.04 (0.03 to 0.06) | 0.00 (0.00 to 0.00) | 0.628 |
| Central Asia | 2009 | 0.00 (0.00 to 0.00) | 0.00 (0.00 to 0.02) | 0.04 (0.03 to 0.05) | 0.00 (0.00 to 0.00) | 0.632 |
| Central Asia | 2010 | 0.00 (0.00 to 0.00) | 0.00 (0.00 to 0.01) | 0.05 (0.03 to 0.06) | 0.00 (0.00 to 0.00) | 0.637 |
| Central Asia | 2011 | 0.00 (0.00 to 0.00) | 0.00 (0.00 to 0.01) | 0.05 (0.03 to 0.07) | 0.00 (0.00 to 0.00) | 0.641 |
| Central Asia | 2012 | 0.00 (0.00 to 0.00) | 0.01 (0.00 to 0.01) | 0.04 (0.03 to 0.05) | 0.00 (0.00 to 0.00) | 0.645 |
| Central Asia | 2013 | 0.00 (0.00 to 0.00) | 0.01 (0.00 to 0.01) | 0.04 (0.03 to 0.06) | 0.00 (0.00 to 0.00) | 0.648 |
| Central Asia | 2014 | 0.00 (0.00 to 0.00) | 0.01 (0.00 to 0.01) | 0.07 (0.03 to 0.08) | 0.00 (0.00 to 0.00) | 0.652 |
| Central Asia | 2015 | 0.00 (0.00 to 0.00) | 0.01 (0.00 to 0.01) | 0.09 (0.04 to 0.11) | 0.00 (0.00 to 0.00) | 0.654 |
| Central Asia | 2016 | 0.00 (0.00 to 0.00) | 0.01 (0.00 to 0.01) | 0.09 (0.03 to 0.12) | 0.00 (0.00 to 0.00) | 0.656 |
| Central Asia | 2017 | 0.00 (0.00 to 0.00) | 0.01 (0.00 to 0.01) | 0.09 (0.03 to 0.12) | 0.00 (0.00 to 0.00) | 0.658 |
| Central Asia | 2018 | 0.00 (0.00 to 0.00) | 0.01 (0.00 to 0.01) | 0.08 (0.03 to 0.11) | 0.00 (0.00 to 0.00) | 0.660 |
| Central Asia | 2019 | 0.00 (0.00 to 0.00) | 0.01 (0.00 to 0.01) | 0.08 (0.03 to 0.10) | 0.00 (0.00 to 0.00) | 0.661 |
| Central Asia | 2020 | 0.00 (0.00 to 0.00) | 0.01 (0.00 to 0.01) | 0.07 (0.03 to 0.09) | 0.00 (0.00 to 0.00) | 0.662 |
| Central Asia | 2021 | 0.00 (0.00 to 0.00) | 0.01 (0.00 to 0.01) | 0.07 (0.02 to 0.09) | 0.00 (0.00 to 0.00) | 0.664 |
| Central Asia | 2022 | 0.00 (0.00 to 0.00) | 0.01 (0.00 to 0.01) | 0.06 (0.02 to 0.08) | 0.00 (0.00 to 0.00) | 0.667 |
| Central Asia | 2023 | 0.00 (0.00 to 0.00) | 0.01 (0.00 to 0.01) | 0.05 (0.02 to 0.07) | 0.00 (0.00 to 0.00) | 0.670 |
| Central Europe | 1990 | 0.01 (0.00 to 0.01) | 0.12 (0.07 to 0.15) | 1.26 (0.78 to 1.53) | 0.05 (0.03 to 0.06) | 0.634 |
| Central Europe | 1991 | 0.01 (0.00 to 0.01) | 0.11 (0.07 to 0.14) | 1.15 (0.72 to 1.39) | 0.05 (0.03 to 0.06) | 0.640 |
| Central Europe | 1992 | 0.01 (0.00 to 0.01) | 0.10 (0.06 to 0.13) | 1.07 (0.66 to 1.29) | 0.04 (0.03 to 0.05) | 0.646 |
| Central Europe | 1993 | 0.01 (0.00 to 0.01) | 0.09 (0.06 to 0.12) | 1.02 (0.61 to 1.25) | 0.04 (0.02 to 0.05) | 0.651 |
| Central Europe | 1994 | 0.01 (0.00 to 0.01) | 0.09 (0.05 to 0.11) | 0.90 (0.53 to 1.13) | 0.04 (0.02 to 0.05) | 0.658 |
| Central Europe | 1995 | 0.00 (0.00 to 0.01) | 0.08 (0.04 to 0.10) | 0.80 (0.48 to 1.00) | 0.03 (0.02 to 0.04) | 0.665 |
| Central Europe | 1996 | 0.00 (0.00 to 0.01) | 0.07 (0.04 to 0.09) | 0.69 (0.42 to 0.87) | 0.03 (0.02 to 0.04) | 0.671 |
| Central Europe | 1997 | 0.00 (0.00 to 0.01) | 0.06 (0.04 to 0.08) | 0.58 (0.35 to 0.73) | 0.02 (0.01 to 0.03) | 0.677 |
| Central Europe | 1998 | 0.00 (0.00 to 0.00) | 0.05 (0.03 to 0.07) | 0.47 (0.31 to 0.60) | 0.02 (0.01 to 0.02) | 0.683 |
| Central Europe | 1999 | 0.00 (0.00 to 0.00) | 0.04 (0.03 to 0.05) | 0.41 (0.27 to 0.51) | 0.02 (0.01 to 0.02) | 0.689 |
| Central Europe | 2000 | 0.00 (0.00 to 0.00) | 0.04 (0.03 to 0.05) | 0.35 (0.23 to 0.44) | 0.01 (0.01 to 0.02) | 0.695 |
| Central Europe | 2001 | 0.00 (0.00 to 0.00) | 0.03 (0.02 to 0.04) | 0.32 (0.21 to 0.41) | 0.01 (0.01 to 0.02) | 0.702 |
| Central Europe | 2002 | 0.00 (0.00 to 0.00) | 0.03 (0.02 to 0.04) | 0.29 (0.18 to 0.37) | 0.01 (0.01 to 0.02) | 0.709 |
| Central Europe | 2003 | 0.00 (0.00 to 0.00) | 0.03 (0.02 to 0.04) | 0.26 (0.17 to 0.34) | 0.01 (0.01 to 0.01) | 0.715 |
| Central Europe | 2004 | 0.00 (0.00 to 0.00) | 0.03 (0.02 to 0.04) | 0.24 (0.15 to 0.31) | 0.01 (0.01 to 0.01) | 0.720 |
| Central Europe | 2005 | 0.00 (0.00 to 0.00) | 0.02 (0.02 to 0.03) | 0.24 (0.15 to 0.31) | 0.01 (0.01 to 0.01) | 0.726 |
| Central Europe | 2006 | 0.00 (0.00 to 0.00) | 0.02 (0.02 to 0.03) | 0.22 (0.14 to 0.30) | 0.01 (0.01 to 0.01) | 0.731 |
| Central Europe | 2007 | 0.00 (0.00 to 0.00) | 0.02 (0.01 to 0.03) | 0.20 (0.12 to 0.27) | 0.01 (0.01 to 0.01) | 0.736 |
| Central Europe | 2008 | 0.00 (0.00 to 0.00) | 0.02 (0.01 to 0.02) | 0.18 (0.10 to 0.25) | 0.01 (0.00 to 0.01) | 0.741 |
| Central Europe | 2009 | 0.00 (0.00 to 0.00) | 0.02 (0.01 to 0.02) | 0.17 (0.09 to 0.23) | 0.01 (0.00 to 0.01) | 0.747 |
| Central Europe | 2010 | 0.00 (0.00 to 0.00) | 0.02 (0.01 to 0.02) | 0.15 (0.08 to 0.20) | 0.01 (0.00 to 0.01) | 0.753 |
| Central Europe | 2011 | 0.00 (0.00 to 0.00) | 0.01 (0.01 to 0.02) | 0.13 (0.08 to 0.18) | 0.01 (0.00 to 0.01) | 0.759 |
| Central Europe | 2012 | 0.00 (0.00 to 0.00) | 0.01 (0.01 to 0.02) | 0.12 (0.07 to 0.16) | 0.00 (0.00 to 0.01) | 0.763 |
| Central Europe | 2013 | 0.00 (0.00 to 0.00) | 0.01 (0.01 to 0.01) | 0.10 (0.07 to 0.14) | 0.00 (0.00 to 0.01) | 0.767 |
| Central Europe | 2014 | 0.00 (0.00 to 0.00) | 0.01 (0.01 to 0.01) | 0.08 (0.06 to 0.11) | 0.00 (0.00 to 0.00) | 0.770 |
| Central Europe | 2015 | 0.00 (0.00 to 0.00) | 0.01 (0.01 to 0.01) | 0.08 (0.06 to 0.10) | 0.00 (0.00 to 0.00) | 0.774 |
| Central Europe | 2016 | 0.00 (0.00 to 0.00) | 0.01 (0.01 to 0.01) | 0.07 (0.05 to 0.08) | 0.00 (0.00 to 0.00) | 0.777 |
| Central Europe | 2017 | 0.00 (0.00 to 0.00) | 0.01 (0.00 to 0.01) | 0.06 (0.04 to 0.08) | 0.00 (0.00 to 0.00) | 0.780 |
| Central Europe | 2018 | 0.00 (0.00 to 0.00) | 0.01 (0.00 to 0.01) | 0.05 (0.04 to 0.08) | 0.00 (0.00 to 0.00) | 0.785 |
| Central Europe | 2019 | 0.00 (0.00 to 0.00) | 0.01 (0.00 to 0.01) | 0.06 (0.03 to 0.08) | 0.00 (0.00 to 0.00) | 0.790 |
| Central Europe | 2020 | 0.00 (0.00 to 0.00) | 0.01 (0.00 to 0.01) | 0.06 (0.03 to 0.08) | 0.00 (0.00 to 0.00) | 0.794 |
| Central Europe | 2021 | 0.00 (0.00 to 0.00) | 0.01 (0.00 to 0.01) | 0.05 (0.03 to 0.08) | 0.00 (0.00 to 0.00) | 0.799 |
| Central Europe | 2022 | 0.00 (0.00 to 0.00) | 0.01 (0.00 to 0.01) | 0.05 (0.03 to 0.08) | 0.00 (0.00 to 0.00) | 0.804 |
| Central Europe | 2023 | 0.00 (0.00 to 0.00) | 0.01 (0.00 to 0.01) | 0.05 (0.03 to 0.07) | 0.00 (0.00 to 0.00) | 0.808 |
| Central Latin America | 1990 | 0.22 (0.17 to 0.28) | 1.06 (0.81 to 1.33) | 29.89 (23.55 to 34.21) | 0.60 (0.47 to 0.72) | 0.497 |
| Central Latin America | 1991 | 0.20 (0.15 to 0.26) | 0.90 (0.70 to 1.13) | 24.17 (19.46 to 27.74) | 0.50 (0.40 to 0.59) | 0.500 |
| Central Latin America | 1992 | 0.19 (0.14 to 0.24) | 0.74 (0.58 to 0.93) | 19.23 (15.94 to 22.15) | 0.40 (0.32 to 0.48) | 0.505 |
| Central Latin America | 1993 | 0.17 (0.13 to 0.22) | 0.62 (0.49 to 0.78) | 15.64 (13.02 to 18.14) | 0.33 (0.27 to 0.40) | 0.510 |
| Central Latin America | 1994 | 0.15 (0.11 to 0.20) | 0.51 (0.41 to 0.65) | 12.91 (10.78 to 15.06) | 0.28 (0.22 to 0.34) | 0.516 |
| Central Latin America | 1995 | 0.14 (0.10 to 0.18) | 0.43 (0.33 to 0.55) | 10.65 (9.00 to 12.47) | 0.23 (0.19 to 0.29) | 0.522 |
| Central Latin America | 1996 | 0.13 (0.09 to 0.17) | 0.37 (0.29 to 0.48) | 8.96 (7.58 to 10.57) | 0.20 (0.16 to 0.24) | 0.528 |
| Central Latin America | 1997 | 0.11 (0.08 to 0.15) | 0.32 (0.26 to 0.41) | 7.56 (6.24 to 9.00) | 0.17 (0.14 to 0.21) | 0.533 |
| Central Latin America | 1998 | 0.10 (0.07 to 0.13) | 0.28 (0.23 to 0.36) | 6.55 (5.40 to 7.99) | 0.15 (0.12 to 0.18) | 0.539 |
| Central Latin America | 1999 | 0.09 (0.06 to 0.11) | 0.25 (0.20 to 0.32) | 5.74 (4.75 to 7.01) | 0.13 (0.11 to 0.16) | 0.545 |
| Central Latin America | 2000 | 0.08 (0.06 to 0.10) | 0.22 (0.17 to 0.29) | 5.06 (4.20 to 6.19) | 0.12 (0.09 to 0.14) | 0.550 |
| Central Latin America | 2001 | 0.07 (0.05 to 0.09) | 0.20 (0.16 to 0.26) | 4.48 (3.69 to 5.50) | 0.10 (0.08 to 0.13) | 0.555 |
| Central Latin America | 2002 | 0.06 (0.05 to 0.08) | 0.18 (0.14 to 0.24) | 4.08 (3.36 to 5.06) | 0.09 (0.08 to 0.12) | 0.560 |
| Central Latin America | 2003 | 0.06 (0.04 to 0.08) | 0.16 (0.13 to 0.22) | 3.68 (3.02 to 4.60) | 0.08 (0.07 to 0.11) | 0.565 |
| Central Latin America | 2004 | 0.05 (0.04 to 0.07) | 0.15 (0.12 to 0.21) | 3.26 (2.66 to 4.03) | 0.07 (0.06 to 0.10) | 0.570 |
| Central Latin America | 2005 | 0.05 (0.03 to 0.07) | 0.13 (0.10 to 0.19) | 2.91 (2.37 to 3.61) | 0.07 (0.05 to 0.09) | 0.575 |
| Central Latin America | 2006 | 0.05 (0.03 to 0.07) | 0.12 (0.09 to 0.17) | 2.52 (2.04 to 3.14) | 0.06 (0.05 to 0.08) | 0.580 |
| Central Latin America | 2007 | 0.04 (0.03 to 0.06) | 0.10 (0.08 to 0.15) | 2.13 (1.74 to 2.66) | 0.05 (0.04 to 0.06) | 0.585 |
| Central Latin America | 2008 | 0.04 (0.03 to 0.06) | 0.09 (0.07 to 0.13) | 1.75 (1.43 to 2.22) | 0.04 (0.03 to 0.05) | 0.590 |
| Central Latin America | 2009 | 0.04 (0.03 to 0.06) | 0.07 (0.06 to 0.12) | 1.48 (1.21 to 1.91) | 0.03 (0.03 to 0.05) | 0.594 |
| Central Latin America | 2010 | 0.04 (0.02 to 0.06) | 0.07 (0.05 to 0.11) | 1.32 (1.07 to 1.70) | 0.03 (0.02 to 0.04) | 0.599 |
| Central Latin America | 2011 | 0.04 (0.02 to 0.06) | 0.06 (0.04 to 0.10) | 1.12 (0.89 to 1.44) | 0.03 (0.02 to 0.04) | 0.604 |
| Central Latin America | 2012 | 0.04 (0.02 to 0.05) | 0.05 (0.04 to 0.09) | 1.03 (0.81 to 1.32) | 0.02 (0.02 to 0.03) | 0.610 |
| Central Latin America | 2013 | 0.03 (0.02 to 0.05) | 0.05 (0.04 to 0.08) | 0.92 (0.71 to 1.18) | 0.02 (0.02 to 0.03) | 0.616 |
| Central Latin America | 2014 | 0.03 (0.02 to 0.05) | 0.05 (0.03 to 0.08) | 0.88 (0.68 to 1.14) | 0.02 (0.02 to 0.03) | 0.621 |
| Central Latin America | 2015 | 0.03 (0.02 to 0.05) | 0.04 (0.03 to 0.08) | 0.84 (0.64 to 1.12) | 0.02 (0.01 to 0.03) | 0.627 |
| Central Latin America | 2016 | 0.03 (0.02 to 0.05) | 0.04 (0.03 to 0.08) | 0.82 (0.63 to 1.11) | 0.02 (0.01 to 0.03) | 0.633 |
| Central Latin America | 2017 | 0.03 (0.02 to 0.05) | 0.04 (0.03 to 0.08) | 0.77 (0.59 to 1.05) | 0.02 (0.01 to 0.03) | 0.639 |
| Central Latin America | 2018 | 0.03 (0.02 to 0.05) | 0.04 (0.03 to 0.08) | 0.74 (0.57 to 1.03) | 0.02 (0.01 to 0.03) | 0.645 |
| Central Latin America | 2019 | 0.03 (0.02 to 0.05) | 0.04 (0.03 to 0.08) | 0.69 (0.53 to 0.97) | 0.02 (0.01 to 0.03) | 0.650 |
| Central Latin America | 2020 | 0.03 (0.02 to 0.05) | 0.04 (0.02 to 0.08) | 0.67 (0.50 to 0.98) | 0.02 (0.01 to 0.03) | 0.654 |
| Central Latin America | 2021 | 0.03 (0.02 to 0.05) | 0.04 (0.02 to 0.08) | 0.68 (0.49 to 1.02) | 0.02 (0.01 to 0.03) | 0.659 |
| Central Latin America | 2022 | 0.03 (0.02 to 0.05) | 0.04 (0.02 to 0.09) | 0.65 (0.45 to 0.99) | 0.02 (0.01 to 0.03) | 0.663 |
| Central Latin America | 2023 | 0.03 (0.02 to 0.05) | 0.04 (0.02 to 0.10) | 0.63 (0.42 to 1.04) | 0.02 (0.01 to 0.03) | 0.667 |
| Central Sub-Saharan Africa | 1990 | 0.71 (0.41 to 1.23) | 6.78 (2.17 to 15.15) | 199.42 (96.82 to 340.85) | 4.40 (1.46 to 8.46) | 0.303 |
| Central Sub-Saharan Africa | 1991 | 0.72 (0.42 to 1.27) | 6.80 (2.24 to 15.85) | 200.17 (98.80 to 345.28) | 4.44 (1.53 to 8.91) | 0.306 |
| Central Sub-Saharan Africa | 1992 | 0.73 (0.42 to 1.31) | 6.86 (2.20 to 16.60) | 195.73 (99.49 to 334.89) | 4.35 (1.46 to 8.80) | 0.309 |
| Central Sub-Saharan Africa | 1993 | 0.74 (0.43 to 1.34) | 7.01 (2.18 to 16.56) | 197.24 (98.89 to 342.76) | 4.41 (1.47 to 9.00) | 0.311 |
| Central Sub-Saharan Africa | 1994 | 0.75 (0.43 to 1.34) | 7.15 (2.23 to 16.48) | 200.09 (97.38 to 353.12) | 4.52 (1.48 to 9.39) | 0.312 |
| Central Sub-Saharan Africa | 1995 | 0.75 (0.43 to 1.33) | 7.21 (2.16 to 16.30) | 203.52 (94.77 to 357.65) | 4.62 (1.50 to 9.37) | 0.313 |
| Central Sub-Saharan Africa | 1996 | 0.76 (0.43 to 1.36) | 7.27 (2.13 to 16.43) | 204.20 (93.85 to 355.17) | 4.64 (1.46 to 9.69) | 0.315 |
| Central Sub-Saharan Africa | 1997 | 0.76 (0.44 to 1.41) | 7.39 (2.09 to 17.05) | 205.84 (91.42 to 381.12) | 4.71 (1.44 to 10.44) | 0.317 |
| Central Sub-Saharan Africa | 1998 | 0.77 (0.43 to 1.43) | 7.53 (2.13 to 18.52) | 211.25 (93.13 to 398.68) | 4.87 (1.47 to 11.07) | 0.319 |
| Central Sub-Saharan Africa | 1999 | 0.77 (0.43 to 1.43) | 7.67 (2.21 to 19.15) | 215.26 (90.22 to 421.80) | 4.98 (1.44 to 11.68) | 0.321 |
| Central Sub-Saharan Africa | 2000 | 0.77 (0.43 to 1.44) | 7.62 (2.08 to 19.71) | 213.54 (85.92 to 423.51) | 4.96 (1.33 to 11.77) | 0.323 |
| Central Sub-Saharan Africa | 2001 | 0.75 (0.42 to 1.40) | 7.33 (1.94 to 18.69) | 202.08 (77.60 to 406.83) | 4.68 (1.21 to 10.60) | 0.326 |
| Central Sub-Saharan Africa | 2002 | 0.73 (0.41 to 1.35) | 6.82 (1.81 to 17.34) | 189.09 (69.18 to 373.43) | 4.38 (1.09 to 9.77) | 0.330 |
| Central Sub-Saharan Africa | 2003 | 0.69 (0.40 to 1.19) | 6.29 (1.67 to 15.91) | 180.31 (67.77 to 356.10) | 4.10 (1.00 to 8.88) | 0.334 |
| Central Sub-Saharan Africa | 2004 | 0.66 (0.39 to 1.14) | 5.70 (1.55 to 14.26) | 168.99 (68.53 to 329.91) | 3.73 (1.00 to 8.05) | 0.339 |
| Central Sub-Saharan Africa | 2005 | 0.63 (0.38 to 1.06) | 5.25 (1.48 to 13.41) | 160.48 (67.39 to 312.91) | 3.44 (0.98 to 7.52) | 0.346 |
| Central Sub-Saharan Africa | 2006 | 0.61 (0.38 to 1.00) | 4.87 (1.46 to 12.19) | 151.85 (61.91 to 295.86) | 3.17 (0.93 to 6.77) | 0.353 |
| Central Sub-Saharan Africa | 2007 | 0.58 (0.37 to 0.95) | 4.45 (1.38 to 10.80) | 139.85 (58.94 to 268.67) | 2.86 (0.86 to 5.81) | 0.361 |
| Central Sub-Saharan Africa | 2008 | 0.56 (0.36 to 0.89) | 4.08 (1.28 to 9.40) | 128.63 (54.96 to 241.55) | 2.61 (0.78 to 5.28) | 0.370 |
| Central Sub-Saharan Africa | 2009 | 0.54 (0.35 to 0.84) | 3.78 (1.14 to 8.30) | 121.06 (51.88 to 226.17) | 2.45 (0.75 to 4.86) | 0.378 |
| Central Sub-Saharan Africa | 2010 | 0.53 (0.34 to 0.81) | 3.56 (1.08 to 7.74) | 114.63 (51.07 to 212.33) | 2.33 (0.72 to 4.60) | 0.386 |
| Central Sub-Saharan Africa | 2011 | 0.51 (0.34 to 0.79) | 3.33 (1.02 to 7.45) | 106.62 (49.68 to 192.87) | 2.17 (0.69 to 4.36) | 0.395 |
| Central Sub-Saharan Africa | 2012 | 0.50 (0.32 to 0.77) | 3.11 (0.97 to 7.30) | 98.98 (44.89 to 175.42) | 2.03 (0.65 to 4.14) | 0.404 |
| Central Sub-Saharan Africa | 2013 | 0.48 (0.32 to 0.75) | 2.97 (0.93 to 6.96) | 93.96 (42.75 to 167.97) | 1.95 (0.62 to 4.08) | 0.413 |
| Central Sub-Saharan Africa | 2014 | 0.46 (0.31 to 0.73) | 2.83 (0.88 to 6.66) | 88.31 (39.53 to 158.19) | 1.86 (0.58 to 3.88) | 0.421 |
| Central Sub-Saharan Africa | 2015 | 0.45 (0.30 to 0.70) | 2.67 (0.82 to 6.34) | 81.00 (36.47 to 144.13) | 1.73 (0.57 to 3.71) | 0.429 |
| Central Sub-Saharan Africa | 2016 | 0.43 (0.29 to 0.67) | 2.57 (0.81 to 6.21) | 76.17 (34.69 to 139.78) | 1.67 (0.56 to 3.65) | 0.436 |
| Central Sub-Saharan Africa | 2017 | 0.42 (0.28 to 0.65) | 2.50 (0.78 to 6.30) | 72.70 (33.24 to 134.03) | 1.62 (0.51 to 3.62) | 0.443 |
| Central Sub-Saharan Africa | 2018 | 0.40 (0.27 to 0.62) | 2.38 (0.71 to 6.19) | 67.72 (30.59 to 125.85) | 1.53 (0.47 to 3.40) | 0.450 |
| Central Sub-Saharan Africa | 2019 | 0.39 (0.26 to 0.60) | 2.25 (0.66 to 6.07) | 62.47 (28.76 to 117.52) | 1.43 (0.45 to 3.14) | 0.456 |
| Central Sub-Saharan Africa | 2020 | 0.37 (0.25 to 0.58) | 2.13 (0.58 to 5.93) | 57.86 (27.64 to 110.21) | 1.33 (0.43 to 2.88) | 0.462 |
| Central Sub-Saharan Africa | 2021 | 0.36 (0.24 to 0.56) | 1.96 (0.51 to 5.43) | 52.75 (24.87 to 99.53) | 1.22 (0.37 to 2.65) | 0.467 |
| Central Sub-Saharan Africa | 2022 | 0.34 (0.23 to 0.54) | 1.77 (0.44 to 5.18) | 46.47 (20.64 to 86.33) | 1.09 (0.32 to 2.38) | 0.473 |
| Central Sub-Saharan Africa | 2023 | 0.33 (0.22 to 0.51) | 1.64 (0.39 to 4.89) | 42.09 (18.34 to 81.40) | 0.99 (0.29 to 2.15) | 0.479 |
| East Asia | 1990 | 3.32 (2.47 to 4.21) | 7.33 (4.72 to 11.05) | 131.72 (82.51 to 198.58) | 1.74 (1.14 to 2.48) | 0.475 |
| East Asia | 1991 | 3.24 (2.40 to 4.13) | 6.59 (4.30 to 9.41) | 116.00 (73.64 to 166.87) | 1.54 (1.00 to 2.17) | 0.483 |
| East Asia | 1992 | 3.14 (2.32 to 3.99) | 5.88 (3.86 to 8.04) | 103.30 (67.44 to 147.88) | 1.38 (0.94 to 1.88) | 0.491 |
| East Asia | 1993 | 3.03 (2.23 to 3.82) | 5.22 (3.53 to 7.39) | 91.06 (60.61 to 126.74) | 1.22 (0.83 to 1.62) | 0.499 |
| East Asia | 1994 | 2.91 (2.13 to 3.64) | 4.61 (3.20 to 6.74) | 80.97 (55.96 to 109.27) | 1.09 (0.76 to 1.44) | 0.507 |
| East Asia | 1995 | 2.78 (2.03 to 3.48) | 4.05 (2.85 to 6.02) | 69.67 (49.05 to 92.13) | 0.94 (0.68 to 1.27) | 0.516 |
| East Asia | 1996 | 2.60 (1.91 to 3.28) | 3.51 (2.56 to 5.01) | 58.00 (42.21 to 78.70) | 0.79 (0.57 to 1.08) | 0.526 |
| East Asia | 1997 | 2.37 (1.73 to 2.99) | 2.97 (2.13 to 3.98) | 48.06 (34.78 to 65.32) | 0.66 (0.49 to 0.87) | 0.536 |
| East Asia | 1998 | 2.10 (1.54 to 2.67) | 2.46 (1.74 to 3.32) | 39.30 (30.18 to 52.08) | 0.54 (0.42 to 0.70) | 0.544 |
| East Asia | 1999 | 1.85 (1.36 to 2.36) | 2.04 (1.46 to 2.75) | 32.26 (24.50 to 41.58) | 0.45 (0.34 to 0.58) | 0.553 |
| East Asia | 2000 | 1.64 (1.21 to 2.11) | 1.72 (1.23 to 2.34) | 27.19 (20.98 to 34.30) | 0.39 (0.30 to 0.50) | 0.561 |
| East Asia | 2001 | 1.47 (1.10 to 1.88) | 1.50 (1.07 to 2.02) | 23.86 (18.36 to 30.29) | 0.34 (0.26 to 0.45) | 0.567 |
| East Asia | 2002 | 1.31 (0.98 to 1.66) | 1.30 (0.93 to 1.72) | 20.50 (16.15 to 25.86) | 0.30 (0.23 to 0.40) | 0.573 |
| East Asia | 2003 | 1.16 (0.87 to 1.46) | 1.13 (0.79 to 1.48) | 17.46 (13.77 to 21.88) | 0.26 (0.20 to 0.34) | 0.580 |
| East Asia | 2004 | 1.02 (0.77 to 1.29) | 0.98 (0.67 to 1.31) | 14.56 (11.44 to 18.27) | 0.22 (0.18 to 0.28) | 0.588 |
| East Asia | 2005 | 0.92 (0.69 to 1.15) | 0.85 (0.59 to 1.15) | 11.69 (9.23 to 14.37) | 0.18 (0.14 to 0.24) | 0.597 |
| East Asia | 2006 | 0.83 (0.62 to 1.05) | 0.72 (0.51 to 0.96) | 8.84 (6.93 to 10.99) | 0.14 (0.11 to 0.18) | 0.607 |
| East Asia | 2007 | 0.75 (0.56 to 0.95) | 0.59 (0.41 to 0.81) | 6.76 (5.36 to 8.43) | 0.11 (0.09 to 0.15) | 0.617 |
| East Asia | 2008 | 0.67 (0.49 to 0.86) | 0.46 (0.32 to 0.68) | 5.19 (4.07 to 6.45) | 0.09 (0.07 to 0.12) | 0.626 |
| East Asia | 2009 | 0.60 (0.44 to 0.78) | 0.36 (0.24 to 0.56) | 4.03 (3.12 to 5.09) | 0.07 (0.05 to 0.10) | 0.635 |
| East Asia | 2010 | 0.55 (0.39 to 0.72) | 0.30 (0.19 to 0.50) | 3.02 (2.25 to 3.84) | 0.06 (0.04 to 0.08) | 0.647 |
| East Asia | 2011 | 0.51 (0.36 to 0.67) | 0.26 (0.17 to 0.45) | 2.39 (1.74 to 3.13) | 0.05 (0.03 to 0.07) | 0.656 |
| East Asia | 2012 | 0.47 (0.34 to 0.63) | 0.23 (0.15 to 0.40) | 2.02 (1.49 to 2.65) | 0.04 (0.03 to 0.06) | 0.661 |
| East Asia | 2013 | 0.44 (0.32 to 0.59) | 0.21 (0.13 to 0.36) | 1.85 (1.35 to 2.41) | 0.04 (0.02 to 0.05) | 0.665 |
| East Asia | 2014 | 0.41 (0.30 to 0.56) | 0.19 (0.11 to 0.32) | 1.72 (1.24 to 2.24) | 0.04 (0.02 to 0.05) | 0.669 |
| East Asia | 2015 | 0.39 (0.28 to 0.53) | 0.17 (0.10 to 0.29) | 1.63 (1.15 to 2.11) | 0.03 (0.02 to 0.05) | 0.671 |
| East Asia | 2016 | 0.35 (0.25 to 0.48) | 0.16 (0.09 to 0.26) | 1.54 (1.06 to 2.05) | 0.03 (0.02 to 0.04) | 0.674 |
| East Asia | 2017 | 0.31 (0.22 to 0.43) | 0.15 (0.08 to 0.25) | 1.38 (0.93 to 1.88) | 0.03 (0.02 to 0.04) | 0.682 |
| East Asia | 2018 | 0.28 (0.19 to 0.39) | 0.13 (0.07 to 0.23) | 1.23 (0.79 to 1.72) | 0.03 (0.01 to 0.04) | 0.691 |
| East Asia | 2019 | 0.25 (0.17 to 0.36) | 0.12 (0.06 to 0.22) | 1.09 (0.67 to 1.59) | 0.02 (0.01 to 0.04) | 0.700 |
| East Asia | 2020 | 0.24 (0.16 to 0.35) | 0.12 (0.05 to 0.23) | 0.99 (0.59 to 1.48) | 0.02 (0.01 to 0.04) | 0.709 |
| East Asia | 2021 | 0.25 (0.17 to 0.36) | 0.11 (0.05 to 0.22) | 0.91 (0.53 to 1.44) | 0.02 (0.01 to 0.03) | 0.717 |
| East Asia | 2022 | 0.26 (0.17 to 0.38) | 0.11 (0.05 to 0.22) | 0.87 (0.49 to 1.47) | 0.02 (0.01 to 0.04) | 0.724 |
| East Asia | 2023 | 0.26 (0.17 to 0.38) | 0.10 (0.04 to 0.19) | 0.78 (0.43 to 1.31) | 0.02 (0.01 to 0.03) | 0.731 |
| Eastern Europe | 1990 | 0.01 (0.00 to 0.01) | 0.08 (0.06 to 0.11) | 0.97 (0.74 to 1.24) | 0.03 (0.03 to 0.04) | 0.671 |
| Eastern Europe | 1991 | 0.01 (0.00 to 0.01) | 0.07 (0.06 to 0.10) | 0.92 (0.72 to 1.18) | 0.03 (0.03 to 0.04) | 0.678 |
| Eastern Europe | 1992 | 0.01 (0.00 to 0.01) | 0.07 (0.05 to 0.09) | 0.89 (0.68 to 1.13) | 0.03 (0.03 to 0.04) | 0.686 |
| Eastern Europe | 1993 | 0.00 (0.00 to 0.01) | 0.06 (0.05 to 0.08) | 0.87 (0.65 to 1.08) | 0.03 (0.02 to 0.04) | 0.691 |
| Eastern Europe | 1994 | 0.00 (0.00 to 0.01) | 0.06 (0.05 to 0.08) | 0.82 (0.62 to 1.03) | 0.03 (0.02 to 0.03) | 0.694 |
| Eastern Europe | 1995 | 0.00 (0.00 to 0.01) | 0.06 (0.04 to 0.07) | 0.75 (0.56 to 0.94) | 0.03 (0.02 to 0.03) | 0.697 |
| Eastern Europe | 1996 | 0.00 (0.00 to 0.01) | 0.05 (0.04 to 0.07) | 0.63 (0.50 to 0.76) | 0.02 (0.02 to 0.03) | 0.701 |
| Eastern Europe | 1997 | 0.00 (0.00 to 0.01) | 0.05 (0.04 to 0.07) | 0.53 (0.43 to 0.64) | 0.02 (0.01 to 0.02) | 0.703 |
| Eastern Europe | 1998 | 0.00 (0.00 to 0.00) | 0.04 (0.03 to 0.06) | 0.46 (0.37 to 0.55) | 0.02 (0.01 to 0.02) | 0.705 |
| Eastern Europe | 1999 | 0.00 (0.00 to 0.00) | 0.03 (0.03 to 0.06) | 0.42 (0.34 to 0.52) | 0.01 (0.01 to 0.02) | 0.707 |
| Eastern Europe | 2000 | 0.00 (0.00 to 0.00) | 0.03 (0.02 to 0.06) | 0.38 (0.29 to 0.48) | 0.01 (0.01 to 0.02) | 0.709 |
| Eastern Europe | 2001 | 0.00 (0.00 to 0.00) | 0.03 (0.02 to 0.05) | 0.34 (0.27 to 0.45) | 0.01 (0.01 to 0.02) | 0.711 |
| Eastern Europe | 2002 | 0.00 (0.00 to 0.00) | 0.02 (0.02 to 0.05) | 0.30 (0.23 to 0.40) | 0.01 (0.01 to 0.02) | 0.714 |
| Eastern Europe | 2003 | 0.00 (0.00 to 0.00) | 0.02 (0.01 to 0.04) | 0.26 (0.20 to 0.34) | 0.01 (0.01 to 0.01) | 0.719 |
| Eastern Europe | 2004 | 0.00 (0.00 to 0.00) | 0.02 (0.01 to 0.04) | 0.21 (0.16 to 0.28) | 0.01 (0.01 to 0.01) | 0.726 |
| Eastern Europe | 2005 | 0.00 (0.00 to 0.00) | 0.01 (0.01 to 0.03) | 0.17 (0.13 to 0.24) | 0.01 (0.00 to 0.01) | 0.733 |
| Eastern Europe | 2006 | 0.00 (0.00 to 0.00) | 0.01 (0.01 to 0.03) | 0.14 (0.11 to 0.20) | 0.01 (0.00 to 0.01) | 0.739 |
| Eastern Europe | 2007 | 0.00 (0.00 to 0.00) | 0.01 (0.01 to 0.03) | 0.11 (0.09 to 0.18) | 0.00 (0.00 to 0.01) | 0.746 |
| Eastern Europe | 2008 | 0.00 (0.00 to 0.00) | 0.01 (0.01 to 0.02) | 0.08 (0.06 to 0.14) | 0.00 (0.00 to 0.01) | 0.752 |
| Eastern Europe | 2009 | 0.00 (0.00 to 0.00) | 0.01 (0.00 to 0.02) | 0.07 (0.05 to 0.11) | 0.00 (0.00 to 0.00) | 0.758 |
| Eastern Europe | 2010 | 0.00 (0.00 to 0.00) | 0.01 (0.00 to 0.02) | 0.06 (0.05 to 0.10) | 0.00 (0.00 to 0.00) | 0.763 |
| Eastern Europe | 2011 | 0.00 (0.00 to 0.00) | 0.01 (0.00 to 0.02) | 0.07 (0.06 to 0.11) | 0.00 (0.00 to 0.00) | 0.767 |
| Eastern Europe | 2012 | 0.00 (0.00 to 0.00) | 0.01 (0.00 to 0.02) | 0.08 (0.07 to 0.11) | 0.00 (0.00 to 0.00) | 0.771 |
| Eastern Europe | 2013 | 0.00 (0.00 to 0.00) | 0.01 (0.00 to 0.02) | 0.08 (0.06 to 0.11) | 0.00 (0.00 to 0.00) | 0.776 |
| Eastern Europe | 2014 | 0.00 (0.00 to 0.00) | 0.01 (0.00 to 0.02) | 0.07 (0.06 to 0.10) | 0.00 (0.00 to 0.00) | 0.781 |
| Eastern Europe | 2015 | 0.00 (0.00 to 0.00) | 0.01 (0.00 to 0.02) | 0.07 (0.06 to 0.10) | 0.00 (0.00 to 0.00) | 0.785 |
| Eastern Europe | 2016 | 0.00 (0.00 to 0.00) | 0.01 (0.00 to 0.02) | 0.07 (0.05 to 0.10) | 0.00 (0.00 to 0.00) | 0.790 |
| Eastern Europe | 2017 | 0.00 (0.00 to 0.00) | 0.01 (0.00 to 0.02) | 0.05 (0.04 to 0.09) | 0.00 (0.00 to 0.00) | 0.795 |
| Eastern Europe | 2018 | 0.00 (0.00 to 0.00) | 0.01 (0.00 to 0.02) | 0.05 (0.04 to 0.09) | 0.00 (0.00 to 0.00) | 0.800 |
| Eastern Europe | 2019 | 0.00 (0.00 to 0.00) | 0.01 (0.00 to 0.02) | 0.05 (0.04 to 0.08) | 0.00 (0.00 to 0.00) | 0.804 |
| Eastern Europe | 2020 | 0.00 (0.00 to 0.00) | 0.00 (0.00 to 0.01) | 0.05 (0.04 to 0.07) | 0.00 (0.00 to 0.00) | 0.806 |
| Eastern Europe | 2021 | 0.00 (0.00 to 0.00) | 0.00 (0.00 to 0.01) | 0.04 (0.03 to 0.06) | 0.00 (0.00 to 0.00) | 0.810 |
| Eastern Europe | 2022 | 0.00 (0.00 to 0.00) | 0.00 (0.00 to 0.01) | 0.04 (0.03 to 0.06) | 0.00 (0.00 to 0.00) | 0.812 |
| Eastern Europe | 2023 | 0.00 (0.00 to 0.00) | 0.00 (0.00 to 0.01) | 0.04 (0.03 to 0.06) | 0.00 (0.00 to 0.00) | 0.815 |
| Eastern Sub-Saharan Africa | 1990 | 2.52 (1.75 to 3.57) | 22.55 (11.04 to 37.22) | 804.72 (384.86 to 1214.13) | 12.24 (6.00 to 18.71) | 0.233 |
| Eastern Sub-Saharan Africa | 1991 | 2.49 (1.74 to 3.48) | 21.16 (10.44 to 35.47) | 762.80 (371.03 to 1148.25) | 11.58 (5.86 to 18.13) | 0.236 |
| Eastern Sub-Saharan Africa | 1992 | 2.46 (1.72 to 3.43) | 19.97 (10.08 to 34.30) | 730.99 (354.93 to 1123.07) | 11.05 (5.56 to 17.81) | 0.239 |
| Eastern Sub-Saharan Africa | 1993 | 2.42 (1.69 to 3.43) | 18.78 (9.81 to 32.82) | 691.73 (335.95 to 1082.03) | 10.44 (5.32 to 16.92) | 0.241 |
| Eastern Sub-Saharan Africa | 1994 | 2.38 (1.66 to 3.30) | 17.73 (9.29 to 31.13) | 654.25 (327.80 to 1019.33) | 9.89 (5.22 to 16.00) | 0.243 |
| Eastern Sub-Saharan Africa | 1995 | 2.34 (1.64 to 3.19) | 16.85 (9.02 to 30.06) | 608.26 (317.91 to 934.94) | 9.24 (4.89 to 14.98) | 0.246 |
| Eastern Sub-Saharan Africa | 1996 | 2.29 (1.63 to 3.12) | 16.06 (8.89 to 28.37) | 560.53 (302.18 to 859.45) | 8.59 (4.57 to 13.87) | 0.248 |
| Eastern Sub-Saharan Africa | 1997 | 2.22 (1.58 to 3.04) | 15.31 (8.53 to 27.01) | 521.40 (279.56 to 808.91) | 8.09 (4.32 to 13.15) | 0.252 |
| Eastern Sub-Saharan Africa | 1998 | 2.14 (1.52 to 2.91) | 14.65 (8.49 to 26.23) | 496.91 (269.62 to 780.68) | 7.77 (4.24 to 12.71) | 0.256 |
| Eastern Sub-Saharan Africa | 1999 | 2.06 (1.45 to 2.80) | 14.00 (7.93 to 26.05) | 477.01 (263.16 to 771.91) | 7.54 (4.17 to 12.09) | 0.260 |
| Eastern Sub-Saharan Africa | 2000 | 1.98 (1.42 to 2.75) | 13.34 (7.38 to 25.78) | 453.94 (261.46 to 734.00) | 7.29 (4.17 to 12.08) | 0.264 |
| Eastern Sub-Saharan Africa | 2001 | 1.91 (1.36 to 2.67) | 12.60 (7.14 to 23.90) | 415.91 (243.23 to 695.20) | 6.81 (3.95 to 11.31) | 0.269 |
| Eastern Sub-Saharan Africa | 2002 | 1.84 (1.30 to 2.59) | 11.92 (6.64 to 22.32) | 388.72 (220.17 to 651.19) | 6.45 (3.56 to 10.95) | 0.274 |
| Eastern Sub-Saharan Africa | 2003 | 1.76 (1.24 to 2.50) | 11.31 (6.16 to 21.07) | 372.86 (206.69 to 621.94) | 6.26 (3.46 to 10.59) | 0.279 |
| Eastern Sub-Saharan Africa | 2004 | 1.69 (1.19 to 2.38) | 10.65 (5.67 to 19.80) | 346.04 (190.39 to 578.41) | 5.87 (3.10 to 10.00) | 0.284 |
| Eastern Sub-Saharan Africa | 2005 | 1.62 (1.14 to 2.26) | 10.01 (5.17 to 19.25) | 316.68 (175.94 to 532.09) | 5.43 (2.85 to 9.13) | 0.290 |
| Eastern Sub-Saharan Africa | 2006 | 1.55 (1.09 to 2.13) | 9.31 (4.86 to 17.76) | 291.30 (159.82 to 486.60) | 5.06 (2.71 to 8.65) | 0.297 |
| Eastern Sub-Saharan Africa | 2007 | 1.47 (1.05 to 2.00) | 8.54 (4.61 to 16.07) | 266.47 (148.84 to 446.33) | 4.66 (2.55 to 8.10) | 0.304 |
| Eastern Sub-Saharan Africa | 2008 | 1.39 (1.01 to 1.87) | 7.72 (4.31 to 14.26) | 241.86 (132.05 to 403.11) | 4.25 (2.32 to 7.32) | 0.312 |
| Eastern Sub-Saharan Africa | 2009 | 1.32 (0.96 to 1.76) | 6.96 (3.81 to 12.70) | 217.18 (122.38 to 358.62) | 3.84 (2.15 to 6.51) | 0.320 |
| Eastern Sub-Saharan Africa | 2010 | 1.26 (0.91 to 1.67) | 6.33 (3.39 to 11.44) | 193.33 (108.31 to 318.59) | 3.43 (1.98 to 5.87) | 0.328 |
| Eastern Sub-Saharan Africa | 2011 | 1.20 (0.88 to 1.58) | 5.82 (3.17 to 10.74) | 174.77 (97.24 to 282.44) | 3.10 (1.79 to 5.26) | 0.336 |
| Eastern Sub-Saharan Africa | 2012 | 1.14 (0.84 to 1.51) | 5.34 (2.98 to 10.03) | 162.91 (95.44 to 260.33) | 2.89 (1.74 to 4.90) | 0.344 |
| Eastern Sub-Saharan Africa | 2013 | 1.08 (0.79 to 1.45) | 4.91 (2.77 to 9.34) | 151.22 (90.67 to 251.74) | 2.69 (1.64 to 4.67) | 0.352 |
| Eastern Sub-Saharan Africa | 2014 | 1.03 (0.75 to 1.37) | 4.53 (2.59 to 9.03) | 140.24 (85.21 to 238.42) | 2.50 (1.51 to 4.38) | 0.360 |
| Eastern Sub-Saharan Africa | 2015 | 0.97 (0.71 to 1.30) | 4.19 (2.36 to 8.84) | 129.80 (78.51 to 226.06) | 2.33 (1.40 to 4.14) | 0.367 |
| Eastern Sub-Saharan Africa | 2016 | 0.92 (0.67 to 1.22) | 3.86 (2.18 to 8.17) | 118.38 (72.25 to 203.16) | 2.14 (1.31 to 3.86) | 0.375 |
| Eastern Sub-Saharan Africa | 2017 | 0.87 (0.63 to 1.15) | 3.57 (1.98 to 7.17) | 108.11 (65.05 to 189.05) | 1.97 (1.18 to 3.60) | 0.383 |
| Eastern Sub-Saharan Africa | 2018 | 0.81 (0.58 to 1.08) | 3.30 (1.78 to 6.22) | 99.23 (57.97 to 173.55) | 1.82 (1.04 to 3.34) | 0.391 |
| Eastern Sub-Saharan Africa | 2019 | 0.77 (0.55 to 1.02) | 3.06 (1.63 to 5.93) | 91.48 (52.71 to 153.71) | 1.68 (0.97 to 3.01) | 0.399 |
| Eastern Sub-Saharan Africa | 2020 | 0.73 (0.53 to 0.97) | 2.88 (1.48 to 5.83) | 86.73 (48.34 to 146.76) | 1.58 (0.89 to 2.81) | 0.407 |
| Eastern Sub-Saharan Africa | 2021 | 0.70 (0.50 to 0.95) | 2.65 (1.34 to 5.35) | 80.40 (45.18 to 135.87) | 1.45 (0.79 to 2.63) | 0.415 |
| Eastern Sub-Saharan Africa | 2022 | 0.68 (0.48 to 0.91) | 2.42 (1.20 to 4.78) | 74.09 (42.10 to 128.34) | 1.34 (0.73 to 2.43) | 0.423 |
| Eastern Sub-Saharan Africa | 2023 | 0.67 (0.47 to 0.88) | 2.23 (1.10 to 4.71) | 66.51 (37.54 to 115.90) | 1.23 (0.66 to 2.21) | 0.431 |
| High-income Asia Pacific | 1990 | 0.03 (0.03 to 0.04) | 0.23 (0.16 to 0.34) | 1.66 (1.20 to 2.16) | 0.04 (0.03 to 0.05) | 0.774 |
| High-income Asia Pacific | 1991 | 0.03 (0.02 to 0.04) | 0.20 (0.15 to 0.29) | 1.43 (1.05 to 1.85) | 0.03 (0.02 to 0.04) | 0.779 |
| High-income Asia Pacific | 1992 | 0.03 (0.02 to 0.04) | 0.17 (0.13 to 0.26) | 1.22 (0.90 to 1.58) | 0.03 (0.02 to 0.04) | 0.785 |
| High-income Asia Pacific | 1993 | 0.03 (0.02 to 0.04) | 0.15 (0.11 to 0.21) | 1.01 (0.76 to 1.34) | 0.02 (0.02 to 0.03) | 0.790 |
| High-income Asia Pacific | 1994 | 0.03 (0.02 to 0.03) | 0.13 (0.09 to 0.18) | 0.85 (0.64 to 1.14) | 0.02 (0.02 to 0.03) | 0.794 |
| High-income Asia Pacific | 1995 | 0.02 (0.02 to 0.03) | 0.12 (0.08 to 0.17) | 0.71 (0.54 to 0.96) | 0.02 (0.01 to 0.02) | 0.799 |
| High-income Asia Pacific | 1996 | 0.02 (0.02 to 0.03) | 0.10 (0.08 to 0.16) | 0.61 (0.46 to 0.81) | 0.01 (0.01 to 0.02) | 0.804 |
| High-income Asia Pacific | 1997 | 0.02 (0.02 to 0.03) | 0.09 (0.07 to 0.14) | 0.55 (0.42 to 0.74) | 0.01 (0.01 to 0.02) | 0.808 |
| High-income Asia Pacific | 1998 | 0.02 (0.02 to 0.03) | 0.09 (0.07 to 0.13) | 0.50 (0.38 to 0.66) | 0.01 (0.01 to 0.02) | 0.811 |
| High-income Asia Pacific | 1999 | 0.02 (0.02 to 0.03) | 0.08 (0.06 to 0.12) | 0.45 (0.35 to 0.59) | 0.01 (0.01 to 0.01) | 0.815 |
| High-income Asia Pacific | 2000 | 0.02 (0.01 to 0.03) | 0.07 (0.05 to 0.11) | 0.42 (0.32 to 0.55) | 0.01 (0.01 to 0.01) | 0.818 |
| High-income Asia Pacific | 2001 | 0.02 (0.01 to 0.03) | 0.07 (0.05 to 0.10) | 0.39 (0.29 to 0.51) | 0.01 (0.01 to 0.01) | 0.821 |
| High-income Asia Pacific | 2002 | 0.02 (0.01 to 0.02) | 0.06 (0.04 to 0.09) | 0.34 (0.26 to 0.45) | 0.01 (0.01 to 0.01) | 0.825 |
| High-income Asia Pacific | 2003 | 0.02 (0.01 to 0.02) | 0.05 (0.04 to 0.08) | 0.29 (0.23 to 0.39) | 0.01 (0.01 to 0.01) | 0.828 |
| High-income Asia Pacific | 2004 | 0.02 (0.01 to 0.02) | 0.05 (0.03 to 0.07) | 0.25 (0.19 to 0.34) | 0.01 (0.00 to 0.01) | 0.831 |
| High-income Asia Pacific | 2005 | 0.01 (0.01 to 0.02) | 0.04 (0.03 to 0.07) | 0.22 (0.17 to 0.30) | 0.01 (0.00 to 0.01) | 0.834 |
| High-income Asia Pacific | 2006 | 0.01 (0.01 to 0.02) | 0.04 (0.03 to 0.06) | 0.19 (0.15 to 0.26) | 0.01 (0.00 to 0.01) | 0.836 |
| High-income Asia Pacific | 2007 | 0.01 (0.01 to 0.02) | 0.04 (0.03 to 0.06) | 0.17 (0.13 to 0.23) | 0.00 (0.00 to 0.01) | 0.839 |
| High-income Asia Pacific | 2008 | 0.01 (0.01 to 0.02) | 0.04 (0.03 to 0.06) | 0.16 (0.12 to 0.21) | 0.00 (0.00 to 0.01) | 0.842 |
| High-income Asia Pacific | 2009 | 0.01 (0.01 to 0.02) | 0.04 (0.03 to 0.06) | 0.15 (0.12 to 0.19) | 0.00 (0.00 to 0.01) | 0.844 |
| High-income Asia Pacific | 2010 | 0.01 (0.01 to 0.01) | 0.04 (0.03 to 0.06) | 0.14 (0.11 to 0.19) | 0.00 (0.00 to 0.01) | 0.847 |
| High-income Asia Pacific | 2011 | 0.01 (0.01 to 0.01) | 0.04 (0.03 to 0.06) | 0.13 (0.10 to 0.17) | 0.00 (0.00 to 0.01) | 0.849 |
| High-income Asia Pacific | 2012 | 0.01 (0.01 to 0.01) | 0.03 (0.02 to 0.05) | 0.12 (0.09 to 0.15) | 0.00 (0.00 to 0.00) | 0.852 |
| High-income Asia Pacific | 2013 | 0.01 (0.01 to 0.01) | 0.03 (0.02 to 0.05) | 0.10 (0.08 to 0.14) | 0.00 (0.00 to 0.00) | 0.855 |
| High-income Asia Pacific | 2014 | 0.01 (0.01 to 0.01) | 0.03 (0.02 to 0.05) | 0.10 (0.08 to 0.13) | 0.00 (0.00 to 0.00) | 0.858 |
| High-income Asia Pacific | 2015 | 0.01 (0.01 to 0.01) | 0.03 (0.02 to 0.05) | 0.10 (0.07 to 0.13) | 0.00 (0.00 to 0.00) | 0.860 |
| High-income Asia Pacific | 2016 | 0.01 (0.00 to 0.01) | 0.03 (0.02 to 0.05) | 0.09 (0.07 to 0.13) | 0.00 (0.00 to 0.00) | 0.863 |
| High-income Asia Pacific | 2017 | 0.01 (0.00 to 0.01) | 0.03 (0.02 to 0.05) | 0.09 (0.07 to 0.12) | 0.00 (0.00 to 0.00) | 0.866 |
| High-income Asia Pacific | 2018 | 0.01 (0.00 to 0.01) | 0.03 (0.02 to 0.05) | 0.09 (0.07 to 0.12) | 0.00 (0.00 to 0.00) | 0.869 |
| High-income Asia Pacific | 2019 | 0.01 (0.00 to 0.01) | 0.03 (0.02 to 0.05) | 0.09 (0.06 to 0.11) | 0.00 (0.00 to 0.00) | 0.872 |
| High-income Asia Pacific | 2020 | 0.01 (0.00 to 0.01) | 0.03 (0.02 to 0.05) | 0.09 (0.06 to 0.12) | 0.00 (0.00 to 0.00) | 0.874 |
| High-income Asia Pacific | 2021 | 0.01 (0.00 to 0.01) | 0.03 (0.02 to 0.05) | 0.10 (0.07 to 0.13) | 0.00 (0.00 to 0.00) | 0.877 |
| High-income Asia Pacific | 2022 | 0.01 (0.01 to 0.01) | 0.03 (0.02 to 0.05) | 0.10 (0.06 to 0.13) | 0.00 (0.00 to 0.00) | 0.880 |
| High-income Asia Pacific | 2023 | 0.01 (0.00 to 0.01) | 0.03 (0.02 to 0.05) | 0.10 (0.06 to 0.13) | 0.00 (0.00 to 0.01) | 0.882 |
| High-income North America | 1990 | 0.00 (0.00 to 0.00) | 0.02 (0.01 to 0.03) | 0.17 (0.12 to 0.23) | 0.01 (0.00 to 0.01) | 0.776 |
| High-income North America | 1991 | 0.00 (0.00 to 0.00) | 0.02 (0.01 to 0.02) | 0.15 (0.11 to 0.20) | 0.01 (0.00 to 0.01) | 0.779 |
| High-income North America | 1992 | 0.00 (0.00 to 0.00) | 0.01 (0.01 to 0.02) | 0.13 (0.10 to 0.17) | 0.00 (0.00 to 0.01) | 0.782 |
| High-income North America | 1993 | 0.00 (0.00 to 0.00) | 0.01 (0.01 to 0.02) | 0.12 (0.09 to 0.16) | 0.00 (0.00 to 0.01) | 0.785 |
| High-income North America | 1994 | 0.00 (0.00 to 0.00) | 0.01 (0.01 to 0.02) | 0.10 (0.08 to 0.13) | 0.00 (0.00 to 0.00) | 0.788 |
| High-income North America | 1995 | 0.00 (0.00 to 0.00) | 0.01 (0.01 to 0.02) | 0.08 (0.06 to 0.11) | 0.00 (0.00 to 0.00) | 0.791 |
| High-income North America | 1996 | 0.00 (0.00 to 0.00) | 0.01 (0.01 to 0.02) | 0.06 (0.05 to 0.09) | 0.00 (0.00 to 0.00) | 0.794 |
| High-income North America | 1997 | 0.00 (0.00 to 0.00) | 0.01 (0.01 to 0.01) | 0.07 (0.05 to 0.09) | 0.00 (0.00 to 0.00) | 0.796 |
| High-income North America | 1998 | 0.00 (0.00 to 0.00) | 0.01 (0.01 to 0.01) | 0.07 (0.05 to 0.10) | 0.00 (0.00 to 0.00) | 0.798 |
| High-income North America | 1999 | 0.00 (0.00 to 0.00) | 0.01 (0.01 to 0.01) | 0.07 (0.05 to 0.09) | 0.00 (0.00 to 0.00) | 0.801 |
| High-income North America | 2000 | 0.00 (0.00 to 0.00) | 0.01 (0.01 to 0.01) | 0.06 (0.05 to 0.08) | 0.00 (0.00 to 0.00) | 0.804 |
| High-income North America | 2001 | 0.00 (0.00 to 0.00) | 0.01 (0.00 to 0.01) | 0.06 (0.04 to 0.08) | 0.00 (0.00 to 0.00) | 0.809 |
| High-income North America | 2002 | 0.00 (0.00 to 0.00) | 0.01 (0.00 to 0.01) | 0.05 (0.04 to 0.07) | 0.00 (0.00 to 0.00) | 0.812 |
| High-income North America | 2003 | 0.00 (0.00 to 0.00) | 0.01 (0.00 to 0.01) | 0.05 (0.04 to 0.07) | 0.00 (0.00 to 0.00) | 0.815 |
| High-income North America | 2004 | 0.00 (0.00 to 0.00) | 0.00 (0.00 to 0.01) | 0.04 (0.03 to 0.06) | 0.00 (0.00 to 0.00) | 0.817 |
| High-income North America | 2005 | 0.00 (0.00 to 0.00) | 0.00 (0.00 to 0.01) | 0.04 (0.03 to 0.05) | 0.00 (0.00 to 0.00) | 0.818 |
| High-income North America | 2006 | 0.00 (0.00 to 0.00) | 0.00 (0.00 to 0.01) | 0.04 (0.03 to 0.05) | 0.00 (0.00 to 0.00) | 0.819 |
| High-income North America | 2007 | 0.00 (0.00 to 0.00) | 0.00 (0.00 to 0.01) | 0.04 (0.03 to 0.05) | 0.00 (0.00 to 0.00) | 0.821 |
| High-income North America | 2008 | 0.00 (0.00 to 0.00) | 0.00 (0.00 to 0.01) | 0.04 (0.03 to 0.05) | 0.00 (0.00 to 0.00) | 0.826 |
| High-income North America | 2009 | 0.00 (0.00 to 0.00) | 0.00 (0.00 to 0.01) | 0.04 (0.03 to 0.06) | 0.00 (0.00 to 0.00) | 0.831 |
| High-income North America | 2010 | 0.00 (0.00 to 0.00) | 0.00 (0.00 to 0.01) | 0.04 (0.03 to 0.05) | 0.00 (0.00 to 0.00) | 0.836 |
| High-income North America | 2011 | 0.00 (0.00 to 0.00) | 0.00 (0.00 to 0.01) | 0.04 (0.03 to 0.05) | 0.00 (0.00 to 0.00) | 0.840 |
| High-income North America | 2012 | 0.00 (0.00 to 0.00) | 0.00 (0.00 to 0.01) | 0.04 (0.03 to 0.05) | 0.00 (0.00 to 0.00) | 0.844 |
| High-income North America | 2013 | 0.00 (0.00 to 0.00) | 0.00 (0.00 to 0.01) | 0.03 (0.02 to 0.05) | 0.00 (0.00 to 0.00) | 0.847 |
| High-income North America | 2014 | 0.00 (0.00 to 0.00) | 0.00 (0.00 to 0.01) | 0.03 (0.02 to 0.04) | 0.00 (0.00 to 0.00) | 0.850 |
| High-income North America | 2015 | 0.00 (0.00 to 0.00) | 0.00 (0.00 to 0.01) | 0.03 (0.02 to 0.04) | 0.00 (0.00 to 0.00) | 0.854 |
| High-income North America | 2016 | 0.00 (0.00 to 0.00) | 0.00 (0.00 to 0.01) | 0.03 (0.02 to 0.04) | 0.00 (0.00 to 0.00) | 0.857 |
| High-income North America | 2017 | 0.00 (0.00 to 0.00) | 0.00 (0.00 to 0.01) | 0.03 (0.02 to 0.04) | 0.00 (0.00 to 0.00) | 0.861 |
| High-income North America | 2018 | 0.00 (0.00 to 0.00) | 0.00 (0.00 to 0.01) | 0.03 (0.02 to 0.04) | 0.00 (0.00 to 0.00) | 0.864 |
| High-income North America | 2019 | 0.00 (0.00 to 0.00) | 0.00 (0.00 to 0.01) | 0.03 (0.02 to 0.04) | 0.00 (0.00 to 0.00) | 0.868 |
| High-income North America | 2020 | 0.00 (0.00 to 0.00) | 0.00 (0.00 to 0.01) | 0.03 (0.02 to 0.04) | 0.00 (0.00 to 0.00) | 0.870 |
| High-income North America | 2021 | 0.00 (0.00 to 0.00) | 0.00 (0.00 to 0.01) | 0.03 (0.02 to 0.04) | 0.00 (0.00 to 0.00) | 0.873 |
| High-income North America | 2022 | 0.00 (0.00 to 0.00) | 0.00 (0.00 to 0.01) | 0.03 (0.02 to 0.04) | 0.00 (0.00 to 0.00) | 0.875 |
| High-income North America | 2023 | 0.00 (0.00 to 0.00) | 0.00 (0.00 to 0.01) | 0.03 (0.02 to 0.04) | 0.00 (0.00 to 0.00) | 0.878 |
| North Africa and Middle East | 1990 | 0.49 (0.35 to 0.71) | 3.52 (1.68 to 6.52) | 132.61 (58.70 to 225.85) | 1.76 (0.86 to 2.74) | 0.445 |
| North Africa and Middle East | 1990 | 0.49 (0.35 to 0.71) | 3.52 (1.68 to 6.52) | 132.61 (58.70 to 225.85) | 1.76 (0.86 to 2.74) | 0.445 |
| North Africa and Middle East | 1991 | 0.48 (0.35 to 0.67) | 3.21 (1.56 to 5.60) | 116.60 (52.56 to 194.87) | 1.55 (0.73 to 2.50) | 0.455 |
| North Africa and Middle East | 1991 | 0.48 (0.35 to 0.67) | 3.21 (1.56 to 5.60) | 116.60 (52.56 to 194.87) | 1.55 (0.73 to 2.50) | 0.455 |
| North Africa and Middle East | 1992 | 0.47 (0.34 to 0.63) | 2.95 (1.42 to 5.06) | 107.61 (48.83 to 181.23) | 1.42 (0.69 to 2.32) | 0.464 |
| North Africa and Middle East | 1992 | 0.47 (0.34 to 0.63) | 2.95 (1.42 to 5.06) | 107.61 (48.83 to 181.23) | 1.42 (0.69 to 2.32) | 0.464 |
| North Africa and Middle East | 1993 | 0.46 (0.34 to 0.62) | 2.90 (1.40 to 4.95) | 111.84 (52.07 to 198.02) | 1.45 (0.72 to 2.46) | 0.472 |
| North Africa and Middle East | 1993 | 0.46 (0.34 to 0.62) | 2.90 (1.40 to 4.95) | 111.84 (52.07 to 198.02) | 1.45 (0.72 to 2.46) | 0.472 |
| North Africa and Middle East | 1994 | 0.47 (0.34 to 0.61) | 2.90 (1.40 to 4.75) | 119.20 (54.96 to 206.79) | 1.52 (0.76 to 2.48) | 0.478 |
| North Africa and Middle East | 1994 | 0.47 (0.34 to 0.61) | 2.90 (1.40 to 4.75) | 119.20 (54.96 to 206.79) | 1.52 (0.76 to 2.48) | 0.478 |
| North Africa and Middle East | 1995 | 0.46 (0.33 to 0.60) | 2.79 (1.33 to 4.74) | 117.45 (52.48 to 198.00) | 1.48 (0.73 to 2.39) | 0.486 |
| North Africa and Middle East | 1995 | 0.46 (0.33 to 0.60) | 2.79 (1.33 to 4.74) | 117.45 (52.48 to 198.00) | 1.48 (0.73 to 2.39) | 0.486 |
| North Africa and Middle East | 1996 | 0.45 (0.32 to 0.59) | 2.63 (1.36 to 4.28) | 112.14 (52.53 to 193.20) | 1.41 (0.73 to 2.30) | 0.494 |
| North Africa and Middle East | 1996 | 0.45 (0.32 to 0.59) | 2.63 (1.36 to 4.28) | 112.14 (52.53 to 193.20) | 1.41 (0.73 to 2.30) | 0.494 |
| North Africa and Middle East | 1997 | 0.43 (0.32 to 0.56) | 2.46 (1.36 to 3.96) | 106.61 (51.83 to 190.67) | 1.34 (0.70 to 2.25) | 0.502 |
| North Africa and Middle East | 1997 | 0.43 (0.32 to 0.56) | 2.46 (1.36 to 3.96) | 106.61 (51.83 to 190.67) | 1.34 (0.70 to 2.25) | 0.502 |
| North Africa and Middle East | 1998 | 0.41 (0.30 to 0.54) | 2.29 (1.35 to 3.75) | 100.68 (51.23 to 179.22) | 1.26 (0.68 to 2.11) | 0.510 |
| North Africa and Middle East | 1998 | 0.41 (0.30 to 0.54) | 2.29 (1.35 to 3.75) | 100.68 (51.23 to 179.22) | 1.26 (0.68 to 2.11) | 0.510 |
| North Africa and Middle East | 1999 | 0.39 (0.29 to 0.52) | 2.12 (1.22 to 3.66) | 92.80 (49.88 to 167.70) | 1.17 (0.66 to 1.96) | 0.518 |
| North Africa and Middle East | 1999 | 0.39 (0.29 to 0.52) | 2.12 (1.22 to 3.66) | 92.80 (49.88 to 167.70) | 1.17 (0.66 to 1.96) | 0.518 |
| North Africa and Middle East | 2000 | 0.38 (0.28 to 0.50) | 1.96 (1.07 to 3.40) | 84.20 (46.31 to 145.60) | 1.06 (0.62 to 1.71) | 0.527 |
| North Africa and Middle East | 2000 | 0.38 (0.28 to 0.50) | 1.96 (1.07 to 3.40) | 84.20 (46.31 to 145.60) | 1.06 (0.62 to 1.71) | 0.527 |
| North Africa and Middle East | 2001 | 0.36 (0.27 to 0.47) | 1.76 (1.03 to 2.90) | 72.77 (41.13 to 122.75) | 0.93 (0.56 to 1.45) | 0.535 |
| North Africa and Middle East | 2001 | 0.36 (0.27 to 0.47) | 1.76 (1.03 to 2.90) | 72.77 (41.13 to 122.75) | 0.93 (0.56 to 1.45) | 0.535 |
| North Africa and Middle East | 2002 | 0.34 (0.26 to 0.45) | 1.55 (0.94 to 2.50) | 60.43 (34.70 to 101.30) | 0.78 (0.47 to 1.22) | 0.543 |
| North Africa and Middle East | 2002 | 0.34 (0.26 to 0.45) | 1.55 (0.94 to 2.50) | 60.43 (34.70 to 101.30) | 0.78 (0.47 to 1.22) | 0.543 |
| North Africa and Middle East | 2003 | 0.33 (0.24 to 0.44) | 1.44 (0.87 to 2.32) | 55.94 (32.57 to 93.96) | 0.72 (0.44 to 1.16) | 0.550 |
| North Africa and Middle East | 2003 | 0.33 (0.24 to 0.44) | 1.44 (0.87 to 2.32) | 55.94 (32.57 to 93.96) | 0.72 (0.44 to 1.16) | 0.550 |
| North Africa and Middle East | 2004 | 0.32 (0.24 to 0.42) | 1.31 (0.80 to 2.16) | 51.80 (28.96 to 87.90) | 0.67 (0.40 to 1.10) | 0.558 |
| North Africa and Middle East | 2004 | 0.32 (0.24 to 0.42) | 1.31 (0.80 to 2.16) | 51.80 (28.96 to 87.90) | 0.67 (0.40 to 1.10) | 0.558 |
| North Africa and Middle East | 2005 | 0.31 (0.23 to 0.40) | 1.16 (0.68 to 1.98) | 45.06 (24.61 to 78.36) | 0.59 (0.35 to 0.96) | 0.565 |
| North Africa and Middle East | 2005 | 0.31 (0.23 to 0.40) | 1.16 (0.68 to 1.98) | 45.06 (24.61 to 78.36) | 0.59 (0.35 to 0.96) | 0.565 |
| North Africa and Middle East | 2006 | 0.30 (0.22 to 0.39) | 1.04 (0.62 to 1.74) | 39.32 (21.77 to 70.14) | 0.52 (0.31 to 0.85) | 0.573 |
| North Africa and Middle East | 2006 | 0.30 (0.22 to 0.39) | 1.04 (0.62 to 1.74) | 39.32 (21.77 to 70.14) | 0.52 (0.31 to 0.85) | 0.573 |
| North Africa and Middle East | 2007 | 0.29 (0.21 to 0.38) | 0.93 (0.57 to 1.50) | 34.24 (18.99 to 59.89) | 0.46 (0.27 to 0.77) | 0.580 |
| North Africa and Middle East | 2007 | 0.29 (0.21 to 0.38) | 0.93 (0.57 to 1.50) | 34.24 (18.99 to 59.89) | 0.46 (0.27 to 0.77) | 0.580 |
| North Africa and Middle East | 2008 | 0.28 (0.21 to 0.36) | 0.81 (0.48 to 1.28) | 29.13 (16.05 to 49.35) | 0.40 (0.23 to 0.62) | 0.588 |
| North Africa and Middle East | 2008 | 0.28 (0.21 to 0.36) | 0.81 (0.48 to 1.28) | 29.13 (16.05 to 49.35) | 0.40 (0.23 to 0.62) | 0.588 |
| North Africa and Middle East | 2009 | 0.27 (0.20 to 0.34) | 0.72 (0.43 to 1.13) | 25.16 (14.47 to 41.99) | 0.35 (0.21 to 0.53) | 0.594 |
| North Africa and Middle East | 2009 | 0.27 (0.20 to 0.34) | 0.72 (0.43 to 1.13) | 25.16 (14.47 to 41.99) | 0.35 (0.21 to 0.53) | 0.594 |
| North Africa and Middle East | 2010 | 0.26 (0.20 to 0.34) | 0.65 (0.38 to 1.07) | 22.48 (12.84 to 37.39) | 0.31 (0.19 to 0.48) | 0.599 |
| North Africa and Middle East | 2010 | 0.26 (0.20 to 0.34) | 0.65 (0.38 to 1.07) | 22.48 (12.84 to 37.39) | 0.31 (0.19 to 0.48) | 0.599 |
| North Africa and Middle East | 2011 | 0.25 (0.19 to 0.33) | 0.59 (0.36 to 0.95) | 20.20 (11.36 to 33.95) | 0.28 (0.17 to 0.44) | 0.605 |
| North Africa and Middle East | 2011 | 0.25 (0.19 to 0.33) | 0.59 (0.36 to 0.95) | 20.20 (11.36 to 33.95) | 0.28 (0.17 to 0.44) | 0.605 |
| North Africa and Middle East | 2012 | 0.24 (0.18 to 0.32) | 0.53 (0.33 to 0.89) | 17.90 (10.24 to 29.34) | 0.25 (0.15 to 0.38) | 0.611 |
| North Africa and Middle East | 2012 | 0.24 (0.18 to 0.32) | 0.53 (0.33 to 0.89) | 17.90 (10.24 to 29.34) | 0.25 (0.15 to 0.38) | 0.611 |
| North Africa and Middle East | 2013 | 0.24 (0.18 to 0.31) | 0.48 (0.30 to 0.78) | 15.59 (8.91 to 25.32) | 0.22 (0.13 to 0.34) | 0.616 |
| North Africa and Middle East | 2013 | 0.24 (0.18 to 0.31) | 0.48 (0.30 to 0.78) | 15.59 (8.91 to 25.32) | 0.22 (0.13 to 0.34) | 0.616 |
| North Africa and Middle East | 2014 | 0.23 (0.17 to 0.30) | 0.43 (0.26 to 0.72) | 13.62 (7.85 to 22.08) | 0.20 (0.12 to 0.30) | 0.622 |
| North Africa and Middle East | 2014 | 0.23 (0.17 to 0.30) | 0.43 (0.26 to 0.72) | 13.62 (7.85 to 22.08) | 0.20 (0.12 to 0.30) | 0.622 |
| North Africa and Middle East | 2015 | 0.23 (0.17 to 0.29) | 0.40 (0.23 to 0.65) | 12.32 (7.32 to 20.31) | 0.18 (0.11 to 0.29) | 0.628 |
| North Africa and Middle East | 2015 | 0.23 (0.17 to 0.29) | 0.40 (0.23 to 0.65) | 12.32 (7.32 to 20.31) | 0.18 (0.11 to 0.29) | 0.628 |
| North Africa and Middle East | 2016 | 0.22 (0.16 to 0.29) | 0.37 (0.22 to 0.62) | 11.28 (6.68 to 18.69) | 0.17 (0.10 to 0.26) | 0.635 |
| North Africa and Middle East | 2016 | 0.22 (0.16 to 0.29) | 0.37 (0.22 to 0.62) | 11.28 (6.68 to 18.69) | 0.17 (0.10 to 0.26) | 0.635 |
| North Africa and Middle East | 2017 | 0.21 (0.16 to 0.27) | 0.35 (0.20 to 0.58) | 10.47 (6.05 to 17.36) | 0.15 (0.10 to 0.24) | 0.642 |
| North Africa and Middle East | 2017 | 0.21 (0.16 to 0.27) | 0.35 (0.20 to 0.58) | 10.47 (6.05 to 17.36) | 0.15 (0.10 to 0.24) | 0.642 |
| North Africa and Middle East | 2018 | 0.20 (0.15 to 0.26) | 0.33 (0.18 to 0.54) | 10.00 (5.64 to 17.30) | 0.15 (0.09 to 0.24) | 0.649 |
| North Africa and Middle East | 2018 | 0.20 (0.15 to 0.26) | 0.33 (0.18 to 0.54) | 10.00 (5.64 to 17.30) | 0.15 (0.09 to 0.24) | 0.649 |
| North Africa and Middle East | 2019 | 0.20 (0.15 to 0.25) | 0.31 (0.17 to 0.55) | 9.50 (5.26 to 17.00) | 0.14 (0.08 to 0.23) | 0.656 |
| North Africa and Middle East | 2019 | 0.20 (0.15 to 0.25) | 0.31 (0.17 to 0.55) | 9.50 (5.26 to 17.00) | 0.14 (0.08 to 0.23) | 0.656 |
| North Africa and Middle East | 2020 | 0.19 (0.15 to 0.25) | 0.29 (0.15 to 0.55) | 9.07 (4.86 to 16.65) | 0.13 (0.08 to 0.22) | 0.662 |
| North Africa and Middle East | 2020 | 0.19 (0.15 to 0.25) | 0.29 (0.15 to 0.55) | 9.07 (4.86 to 16.65) | 0.13 (0.08 to 0.22) | 0.662 |
| North Africa and Middle East | 2021 | 0.19 (0.14 to 0.24) | 0.27 (0.14 to 0.54) | 8.51 (4.49 to 15.89) | 0.12 (0.07 to 0.21) | 0.669 |
| North Africa and Middle East | 2021 | 0.19 (0.14 to 0.24) | 0.27 (0.14 to 0.54) | 8.51 (4.49 to 15.89) | 0.12 (0.07 to 0.21) | 0.669 |
| North Africa and Middle East | 2022 | 0.18 (0.14 to 0.24) | 0.25 (0.12 to 0.52) | 7.61 (3.94 to 13.91) | 0.11 (0.06 to 0.19) | 0.676 |
| North Africa and Middle East | 2022 | 0.18 (0.14 to 0.24) | 0.25 (0.12 to 0.52) | 7.61 (3.94 to 13.91) | 0.11 (0.06 to 0.19) | 0.676 |
| North Africa and Middle East | 2023 | 0.18 (0.13 to 0.24) | 0.22 (0.11 to 0.48) | 6.44 (3.38 to 11.78) | 0.09 (0.05 to 0.16) | 0.684 |
| North Africa and Middle East | 2023 | 0.18 (0.13 to 0.24) | 0.22 (0.11 to 0.48) | 6.44 (3.38 to 11.78) | 0.09 (0.05 to 0.16) | 0.684 |
| Oceania | 1990 | 0.08 (0.04 to 0.18) | 0.91 (0.23 to 2.89) | 4.00 (1.46 to 8.82) | 0.18 (0.05 to 0.44) | 0.405 |
| Oceania | 1991 | 0.08 (0.04 to 0.18) | 0.85 (0.22 to 2.66) | 3.73 (1.36 to 8.34) | 0.17 (0.04 to 0.43) | 0.409 |
| Oceania | 1992 | 0.08 (0.04 to 0.19) | 0.81 (0.21 to 2.49) | 3.55 (1.32 to 7.66) | 0.16 (0.04 to 0.41) | 0.412 |
| Oceania | 1993 | 0.08 (0.04 to 0.17) | 0.76 (0.19 to 2.36) | 3.37 (1.25 to 7.19) | 0.15 (0.04 to 0.37) | 0.416 |
| Oceania | 1994 | 0.07 (0.04 to 0.16) | 0.73 (0.19 to 2.25) | 3.18 (1.10 to 6.51) | 0.14 (0.04 to 0.36) | 0.420 |
| Oceania | 1995 | 0.07 (0.04 to 0.14) | 0.71 (0.18 to 2.18) | 3.02 (1.02 to 6.13) | 0.14 (0.04 to 0.34) | 0.424 |
| Oceania | 1996 | 0.07 (0.04 to 0.14) | 0.69 (0.19 to 2.12) | 2.92 (1.06 to 6.05) | 0.14 (0.04 to 0.34) | 0.428 |
| Oceania | 1997 | 0.07 (0.04 to 0.14) | 0.68 (0.19 to 2.08) | 2.88 (1.07 to 5.92) | 0.14 (0.04 to 0.33) | 0.432 |
| Oceania | 1998 | 0.07 (0.04 to 0.14) | 0.67 (0.19 to 2.04) | 2.91 (1.08 to 5.99) | 0.14 (0.04 to 0.33) | 0.436 |
| Oceania | 1999 | 0.07 (0.04 to 0.14) | 0.66 (0.19 to 2.03) | 2.86 (1.08 to 5.91) | 0.13 (0.04 to 0.32) | 0.439 |
| Oceania | 2000 | 0.07 (0.04 to 0.15) | 0.65 (0.18 to 2.00) | 2.73 (1.04 to 5.57) | 0.13 (0.04 to 0.31) | 0.441 |
| Oceania | 2001 | 0.07 (0.04 to 0.14) | 0.63 (0.18 to 1.92) | 2.55 (0.95 to 5.29) | 0.12 (0.03 to 0.30) | 0.443 |
| Oceania | 2002 | 0.06 (0.04 to 0.14) | 0.60 (0.17 to 1.81) | 2.40 (0.89 to 5.05) | 0.12 (0.03 to 0.29) | 0.445 |
| Oceania | 2003 | 0.06 (0.03 to 0.13) | 0.58 (0.16 to 1.75) | 2.28 (0.85 to 4.80) | 0.11 (0.03 to 0.28) | 0.446 |
| Oceania | 2004 | 0.06 (0.03 to 0.12) | 0.55 (0.14 to 1.66) | 2.22 (0.82 to 4.83) | 0.11 (0.03 to 0.28) | 0.447 |
| Oceania | 2005 | 0.06 (0.03 to 0.12) | 0.54 (0.13 to 1.59) | 2.17 (0.82 to 4.93) | 0.11 (0.03 to 0.29) | 0.449 |
| Oceania | 2006 | 0.06 (0.03 to 0.12) | 0.55 (0.14 to 1.59) | 2.17 (0.81 to 5.24) | 0.11 (0.03 to 0.32) | 0.451 |
| Oceania | 2007 | 0.06 (0.03 to 0.12) | 0.56 (0.14 to 1.59) | 2.15 (0.79 to 5.22) | 0.11 (0.03 to 0.32) | 0.452 |
| Oceania | 2008 | 0.06 (0.03 to 0.12) | 0.57 (0.15 to 1.71) | 2.21 (0.83 to 5.43) | 0.12 (0.03 to 0.35) | 0.454 |
| Oceania | 2009 | 0.06 (0.03 to 0.13) | 0.59 (0.15 to 1.92) | 2.19 (0.81 to 5.29) | 0.12 (0.03 to 0.34) | 0.455 |
| Oceania | 2010 | 0.06 (0.03 to 0.13) | 0.61 (0.15 to 2.04) | 2.24 (0.79 to 5.61) | 0.12 (0.03 to 0.35) | 0.457 |
| Oceania | 2011 | 0.06 (0.03 to 0.13) | 0.63 (0.15 to 2.12) | 2.27 (0.72 to 5.96) | 0.13 (0.03 to 0.38) | 0.459 |
| Oceania | 2012 | 0.06 (0.03 to 0.15) | 0.65 (0.14 to 2.19) | 2.29 (0.73 to 6.05) | 0.13 (0.03 to 0.38) | 0.461 |
| Oceania | 2013 | 0.06 (0.03 to 0.16) | 0.67 (0.14 to 2.26) | 2.25 (0.72 to 5.97) | 0.13 (0.03 to 0.38) | 0.463 |
| Oceania | 2014 | 0.06 (0.03 to 0.16) | 0.70 (0.14 to 2.45) | 2.29 (0.72 to 6.33) | 0.13 (0.03 to 0.41) | 0.466 |
| Oceania | 2015 | 0.06 (0.03 to 0.16) | 0.72 (0.13 to 2.44) | 2.34 (0.73 to 6.37) | 0.14 (0.03 to 0.44) | 0.469 |
| Oceania | 2016 | 0.06 (0.03 to 0.17) | 0.73 (0.13 to 2.64) | 2.35 (0.69 to 6.54) | 0.14 (0.03 to 0.44) | 0.472 |
| Oceania | 2017 | 0.06 (0.03 to 0.17) | 0.75 (0.13 to 2.75) | 2.35 (0.69 to 6.63) | 0.14 (0.03 to 0.44) | 0.475 |
| Oceania | 2018 | 0.06 (0.03 to 0.17) | 0.77 (0.13 to 2.94) | 2.39 (0.65 to 6.83) | 0.15 (0.03 to 0.47) | 0.477 |
| Oceania | 2019 | 0.06 (0.03 to 0.18) | 0.78 (0.13 to 3.25) | 2.44 (0.64 to 7.04) | 0.15 (0.03 to 0.48) | 0.480 |
| Oceania | 2020 | 0.06 (0.03 to 0.18) | 0.80 (0.13 to 3.42) | 2.41 (0.66 to 7.18) | 0.15 (0.03 to 0.49) | 0.481 |
| Oceania | 2021 | 0.07 (0.03 to 0.19) | 0.81 (0.14 to 3.35) | 2.41 (0.67 to 7.29) | 0.15 (0.03 to 0.49) | 0.481 |
| Oceania | 2022 | 0.07 (0.03 to 0.19) | 0.82 (0.14 to 3.28) | 2.38 (0.64 to 7.43) | 0.15 (0.03 to 0.50) | 0.481 |
| Oceania | 2023 | 0.06 (0.03 to 0.16) | 0.77 (0.14 to 2.75) | 2.21 (0.62 to 6.99) | 0.14 (0.03 to 0.47) | 0.482 |
| South Asia | 1990 | 4.89 (3.72 to 6.04) | 23.38 (17.39 to 31.39) | 953.50 (757.86 to 1206.80) | 12.15 (9.60 to 15.17) | 0.328 |
| South Asia | 1990 | 4.89 (3.72 to 6.04) | 23.38 (17.39 to 31.39) | 953.50 (757.86 to 1206.80) | 12.15 (9.60 to 15.17) | 0.328 |
| South Asia | 1991 | 4.72 (3.58 to 5.83) | 20.77 (15.54 to 26.87) | 813.09 (645.39 to 1021.27) | 10.42 (8.21 to 13.14) | 0.334 |
| South Asia | 1991 | 4.72 (3.58 to 5.83) | 20.77 (15.54 to 26.87) | 813.09 (645.39 to 1021.27) | 10.42 (8.21 to 13.14) | 0.334 |
| South Asia | 1992 | 4.53 (3.39 to 5.60) | 18.44 (13.64 to 23.64) | 715.06 (567.67 to 889.25) | 9.19 (7.21 to 11.61) | 0.340 |
| South Asia | 1992 | 4.53 (3.39 to 5.60) | 18.44 (13.64 to 23.64) | 715.06 (567.67 to 889.25) | 9.19 (7.21 to 11.61) | 0.340 |
| South Asia | 1993 | 4.34 (3.25 to 5.35) | 16.35 (12.18 to 21.23) | 638.98 (511.42 to 794.28) | 8.21 (6.60 to 10.42) | 0.345 |
| South Asia | 1993 | 4.34 (3.25 to 5.35) | 16.35 (12.18 to 21.23) | 638.98 (511.42 to 794.28) | 8.21 (6.60 to 10.42) | 0.345 |
| South Asia | 1994 | 4.14 (3.10 to 5.13) | 14.56 (10.95 to 19.24) | 578.42 (462.71 to 714.09) | 7.45 (5.91 to 9.22) | 0.351 |
| South Asia | 1994 | 4.14 (3.10 to 5.13) | 14.56 (10.95 to 19.24) | 578.42 (462.71 to 714.09) | 7.45 (5.91 to 9.22) | 0.351 |
| South Asia | 1995 | 3.95 (2.96 to 4.92) | 13.16 (9.82 to 17.92) | 522.75 (423.54 to 641.33) | 6.76 (5.37 to 8.25) | 0.358 |
| South Asia | 1995 | 3.95 (2.96 to 4.92) | 13.16 (9.82 to 17.92) | 522.75 (423.54 to 641.33) | 6.76 (5.37 to 8.25) | 0.358 |
| South Asia | 1996 | 3.73 (2.79 to 4.64) | 11.96 (8.87 to 15.77) | 463.39 (364.54 to 563.15) | 6.03 (4.71 to 7.31) | 0.364 |
| South Asia | 1996 | 3.73 (2.79 to 4.64) | 11.96 (8.87 to 15.77) | 463.39 (364.54 to 563.15) | 6.03 (4.71 to 7.31) | 0.364 |
| South Asia | 1997 | 3.46 (2.58 to 4.31) | 10.84 (7.98 to 14.10) | 409.50 (317.98 to 505.55) | 5.39 (4.07 to 6.55) | 0.371 |
| South Asia | 1997 | 3.46 (2.58 to 4.31) | 10.84 (7.98 to 14.10) | 409.50 (317.98 to 505.55) | 5.39 (4.07 to 6.55) | 0.371 |
| South Asia | 1998 | 3.17 (2.35 to 3.96) | 9.85 (7.15 to 12.64) | 367.19 (284.25 to 457.38) | 4.88 (3.73 to 5.99) | 0.378 |
| South Asia | 1998 | 3.17 (2.35 to 3.96) | 9.85 (7.15 to 12.64) | 367.19 (284.25 to 457.38) | 4.88 (3.73 to 5.99) | 0.378 |
| South Asia | 1999 | 2.90 (2.14 to 3.63) | 8.97 (6.39 to 11.65) | 332.39 (255.73 to 415.91) | 4.44 (3.41 to 5.51) | 0.384 |
| South Asia | 1999 | 2.90 (2.14 to 3.63) | 8.97 (6.39 to 11.65) | 332.39 (255.73 to 415.91) | 4.44 (3.41 to 5.51) | 0.384 |
| South Asia | 2000 | 2.69 (1.97 to 3.37) | 8.25 (5.75 to 10.89) | 302.55 (236.65 to 384.90) | 4.07 (3.13 to 5.17) | 0.391 |
| South Asia | 2000 | 2.69 (1.97 to 3.37) | 8.25 (5.75 to 10.89) | 302.55 (236.65 to 384.90) | 4.07 (3.13 to 5.17) | 0.391 |
| South Asia | 2001 | 2.51 (1.85 to 3.15) | 7.61 (5.32 to 10.13) | 277.31 (222.11 to 356.95) | 3.75 (2.90 to 4.75) | 0.397 |
| South Asia | 2001 | 2.51 (1.85 to 3.15) | 7.61 (5.32 to 10.13) | 277.31 (222.11 to 356.95) | 3.75 (2.90 to 4.75) | 0.397 |
| South Asia | 2002 | 2.34 (1.74 to 2.95) | 6.97 (4.93 to 9.29) | 254.45 (202.86 to 330.50) | 3.45 (2.68 to 4.38) | 0.403 |
| South Asia | 2002 | 2.34 (1.74 to 2.95) | 6.97 (4.93 to 9.29) | 254.45 (202.86 to 330.50) | 3.45 (2.68 to 4.38) | 0.403 |
| South Asia | 2003 | 2.19 (1.63 to 2.76) | 6.35 (4.52 to 8.39) | 231.34 (185.08 to 294.05) | 3.14 (2.46 to 3.92) | 0.408 |
| South Asia | 2003 | 2.19 (1.63 to 2.76) | 6.35 (4.52 to 8.39) | 231.34 (185.08 to 294.05) | 3.14 (2.46 to 3.92) | 0.408 |
| South Asia | 2004 | 2.05 (1.54 to 2.59) | 5.76 (4.07 to 7.70) | 208.91 (170.21 to 257.94) | 2.84 (2.19 to 3.48) | 0.415 |
| South Asia | 2004 | 2.05 (1.54 to 2.59) | 5.76 (4.07 to 7.70) | 208.91 (170.21 to 257.94) | 2.84 (2.19 to 3.48) | 0.415 |
| South Asia | 2005 | 1.93 (1.45 to 2.45) | 5.27 (3.70 to 7.09) | 187.05 (148.38 to 233.49) | 2.54 (1.98 to 3.12) | 0.422 |
| South Asia | 2005 | 1.93 (1.45 to 2.45) | 5.27 (3.70 to 7.09) | 187.05 (148.38 to 233.49) | 2.54 (1.98 to 3.12) | 0.422 |
| South Asia | 2006 | 1.82 (1.38 to 2.31) | 4.82 (3.37 to 6.48) | 166.71 (129.06 to 206.53) | 2.29 (1.79 to 2.85) | 0.429 |
| South Asia | 2006 | 1.82 (1.38 to 2.31) | 4.82 (3.37 to 6.48) | 166.71 (129.06 to 206.53) | 2.29 (1.79 to 2.85) | 0.429 |
| South Asia | 2007 | 1.72 (1.30 to 2.17) | 4.34 (2.99 to 5.85) | 148.23 (112.03 to 184.07) | 2.05 (1.54 to 2.60) | 0.437 |
| South Asia | 2007 | 1.72 (1.30 to 2.17) | 4.34 (2.99 to 5.85) | 148.23 (112.03 to 184.07) | 2.05 (1.54 to 2.60) | 0.437 |
| South Asia | 2008 | 1.63 (1.22 to 2.05) | 3.86 (2.64 to 5.20) | 130.14 (96.86 to 163.70) | 1.81 (1.34 to 2.35) | 0.444 |
| South Asia | 2008 | 1.63 (1.22 to 2.05) | 3.86 (2.64 to 5.20) | 130.14 (96.86 to 163.70) | 1.81 (1.34 to 2.35) | 0.444 |
| South Asia | 2009 | 1.54 (1.15 to 1.94) | 3.41 (2.29 to 4.66) | 109.19 (81.48 to 141.98) | 1.54 (1.15 to 2.00) | 0.453 |
| South Asia | 2009 | 1.54 (1.15 to 1.94) | 3.41 (2.29 to 4.66) | 109.19 (81.48 to 141.98) | 1.54 (1.15 to 2.00) | 0.453 |
| South Asia | 2010 | 1.47 (1.10 to 1.85) | 3.04 (2.00 to 4.18) | 92.50 (69.08 to 121.27) | 1.32 (0.97 to 1.72) | 0.462 |
| South Asia | 2010 | 1.47 (1.10 to 1.85) | 3.04 (2.00 to 4.18) | 92.50 (69.08 to 121.27) | 1.32 (0.97 to 1.72) | 0.462 |
| South Asia | 2011 | 1.40 (1.05 to 1.77) | 2.71 (1.81 to 3.67) | 79.70 (58.19 to 105.88) | 1.14 (0.83 to 1.49) | 0.471 |
| South Asia | 2011 | 1.40 (1.05 to 1.77) | 2.71 (1.81 to 3.67) | 79.70 (58.19 to 105.88) | 1.14 (0.83 to 1.49) | 0.471 |
| South Asia | 2012 | 1.34 (1.00 to 1.69) | 2.39 (1.53 to 3.48) | 69.40 (50.91 to 92.65) | 1.00 (0.71 to 1.33) | 0.481 |
| South Asia | 2012 | 1.34 (1.00 to 1.69) | 2.39 (1.53 to 3.48) | 69.40 (50.91 to 92.65) | 1.00 (0.71 to 1.33) | 0.481 |
| South Asia | 2013 | 1.28 (0.96 to 1.62) | 2.09 (1.26 to 3.12) | 61.19 (43.63 to 83.61) | 0.88 (0.60 to 1.23) | 0.492 |
| South Asia | 2013 | 1.28 (0.96 to 1.62) | 2.09 (1.26 to 3.12) | 61.19 (43.63 to 83.61) | 0.88 (0.60 to 1.23) | 0.492 |
| South Asia | 2014 | 1.23 (0.92 to 1.56) | 1.83 (1.07 to 2.87) | 54.20 (37.11 to 76.82) | 0.78 (0.52 to 1.12) | 0.503 |
| South Asia | 2014 | 1.23 (0.92 to 1.56) | 1.83 (1.07 to 2.87) | 54.20 (37.11 to 76.82) | 0.78 (0.52 to 1.12) | 0.503 |
| South Asia | 2015 | 1.18 (0.89 to 1.50) | 1.64 (0.93 to 2.71) | 48.18 (32.03 to 70.75) | 0.70 (0.46 to 1.02) | 0.514 |
| South Asia | 2015 | 1.18 (0.89 to 1.50) | 1.64 (0.93 to 2.71) | 48.18 (32.03 to 70.75) | 0.70 (0.46 to 1.02) | 0.514 |
| South Asia | 2016 | 1.14 (0.85 to 1.44) | 1.50 (0.83 to 2.50) | 43.25 (27.80 to 66.04) | 0.63 (0.41 to 0.94) | 0.525 |
| South Asia | 2016 | 1.14 (0.85 to 1.44) | 1.50 (0.83 to 2.50) | 43.25 (27.80 to 66.04) | 0.63 (0.41 to 0.94) | 0.525 |
| South Asia | 2017 | 1.09 (0.82 to 1.37) | 1.38 (0.75 to 2.31) | 39.33 (24.83 to 61.11) | 0.57 (0.36 to 0.87) | 0.536 |
| South Asia | 2017 | 1.09 (0.82 to 1.37) | 1.38 (0.75 to 2.31) | 39.33 (24.83 to 61.11) | 0.57 (0.36 to 0.87) | 0.536 |
| South Asia | 2018 | 1.04 (0.79 to 1.31) | 1.26 (0.68 to 2.14) | 35.22 (21.89 to 55.48) | 0.51 (0.31 to 0.78) | 0.546 |
| South Asia | 2018 | 1.04 (0.79 to 1.31) | 1.26 (0.68 to 2.14) | 35.22 (21.89 to 55.48) | 0.51 (0.31 to 0.78) | 0.546 |
| South Asia | 2019 | 1.00 (0.76 to 1.25) | 1.16 (0.62 to 1.99) | 31.83 (19.82 to 50.15) | 0.46 (0.28 to 0.70) | 0.555 |
| South Asia | 2019 | 1.00 (0.76 to 1.25) | 1.16 (0.62 to 1.99) | 31.83 (19.82 to 50.15) | 0.46 (0.28 to 0.70) | 0.555 |
| South Asia | 2020 | 0.97 (0.74 to 1.22) | 1.06 (0.56 to 1.87) | 30.19 (18.40 to 46.43) | 0.43 (0.26 to 0.67) | 0.562 |
| South Asia | 2020 | 0.97 (0.74 to 1.22) | 1.06 (0.56 to 1.87) | 30.19 (18.40 to 46.43) | 0.43 (0.26 to 0.67) | 0.562 |
| South Asia | 2021 | 0.93 (0.69 to 1.17) | 0.93 (0.49 to 1.63) | 26.86 (16.26 to 41.84) | 0.39 (0.23 to 0.60) | 0.570 |
| South Asia | 2021 | 0.93 (0.69 to 1.17) | 0.93 (0.49 to 1.63) | 26.86 (16.26 to 41.84) | 0.39 (0.23 to 0.60) | 0.570 |
| South Asia | 2022 | 0.89 (0.66 to 1.14) | 0.81 (0.41 to 1.48) | 23.58 (13.81 to 37.14) | 0.34 (0.19 to 0.54) | 0.578 |
| South Asia | 2022 | 0.89 (0.66 to 1.14) | 0.81 (0.41 to 1.48) | 23.58 (13.81 to 37.14) | 0.34 (0.19 to 0.54) | 0.578 |
| South Asia | 2023 | 0.90 (0.66 to 1.15) | 0.72 (0.36 to 1.34) | 20.47 (11.99 to 32.50) | 0.30 (0.17 to 0.49) | 0.585 |
| South Asia | 2023 | 0.90 (0.66 to 1.15) | 0.72 (0.36 to 1.34) | 20.47 (11.99 to 32.50) | 0.30 (0.17 to 0.49) | 0.585 |
| Southeast Asia | 1990 | 5.40 (4.01 to 7.26) | 30.47 (14.63 to 53.43) | 578.66 (271.92 to 930.69) | 9.09 (4.49 to 15.07) | 0.469 |
| Southeast Asia | 1991 | 5.22 (3.88 to 6.93) | 26.89 (13.13 to 47.02) | 495.77 (246.30 to 800.91) | 7.78 (3.86 to 12.55) | 0.476 |
| Southeast Asia | 1992 | 5.04 (3.74 to 6.63) | 23.57 (11.71 to 40.36) | 432.63 (219.71 to 717.70) | 6.80 (3.38 to 10.97) | 0.484 |
| Southeast Asia | 1993 | 4.88 (3.60 to 6.38) | 20.68 (10.02 to 34.13) | 380.81 (191.74 to 642.79) | 6.01 (2.95 to 9.63) | 0.491 |
| Southeast Asia | 1994 | 4.73 (3.50 to 6.22) | 18.32 (8.65 to 31.80) | 337.81 (162.46 to 582.22) | 5.38 (2.70 to 8.83) | 0.499 |
| Southeast Asia | 1995 | 4.62 (3.40 to 6.04) | 16.64 (7.87 to 30.20) | 300.74 (144.51 to 515.13) | 4.84 (2.43 to 7.84) | 0.507 |
| Southeast Asia | 1996 | 4.45 (3.30 to 5.84) | 15.40 (7.65 to 27.51) | 269.15 (132.12 to 453.99) | 4.38 (2.27 to 7.07) | 0.515 |
| Southeast Asia | 1997 | 4.19 (3.11 to 5.43) | 14.29 (7.34 to 25.16) | 242.24 (122.67 to 395.87) | 4.00 (2.07 to 6.45) | 0.522 |
| Southeast Asia | 1998 | 3.88 (2.88 to 4.97) | 13.30 (7.04 to 24.07) | 221.73 (109.91 to 366.70) | 3.70 (1.99 to 5.97) | 0.528 |
| Southeast Asia | 1999 | 3.57 (2.67 to 4.54) | 12.41 (6.68 to 23.33) | 202.67 (100.38 to 339.06) | 3.43 (1.86 to 5.68) | 0.533 |
| Southeast Asia | 2000 | 3.33 (2.49 to 4.22) | 11.64 (6.21 to 22.52) | 184.02 (93.75 to 311.76) | 3.17 (1.71 to 5.29) | 0.538 |
| Southeast Asia | 2001 | 3.13 (2.33 to 3.94) | 10.91 (5.77 to 21.05) | 167.79 (86.74 to 277.04) | 2.94 (1.56 to 4.86) | 0.542 |
| Southeast Asia | 2002 | 2.95 (2.20 to 3.69) | 10.17 (5.22 to 19.54) | 152.71 (80.45 to 257.55) | 2.73 (1.43 to 4.59) | 0.546 |
| Southeast Asia | 2003 | 2.78 (2.07 to 3.50) | 9.47 (4.64 to 18.23) | 140.07 (72.59 to 240.22) | 2.54 (1.29 to 4.32) | 0.551 |
| Southeast Asia | 2004 | 2.62 (1.96 to 3.29) | 8.82 (4.19 to 16.85) | 130.58 (68.34 to 222.15) | 2.40 (1.20 to 4.11) | 0.556 |
| Southeast Asia | 2005 | 2.47 (1.85 to 3.12) | 8.25 (3.84 to 16.01) | 118.60 (59.89 to 209.52) | 2.20 (1.07 to 3.79) | 0.561 |
| Southeast Asia | 2006 | 2.34 (1.75 to 2.93) | 7.71 (3.62 to 15.13) | 108.81 (54.61 to 188.08) | 2.04 (0.97 to 3.50) | 0.565 |
| Southeast Asia | 2007 | 2.20 (1.64 to 2.74) | 7.18 (3.38 to 14.20) | 99.63 (50.93 to 165.68) | 1.88 (0.86 to 3.20) | 0.571 |
| Southeast Asia | 2008 | 2.08 (1.55 to 2.58) | 6.66 (3.04 to 12.75) | 92.12 (47.49 to 149.53) | 1.76 (0.83 to 3.03) | 0.576 |
| Southeast Asia | 2009 | 1.96 (1.46 to 2.42) | 6.18 (2.75 to 11.78) | 82.72 (42.56 to 135.62) | 1.61 (0.74 to 2.66) | 0.582 |
| Southeast Asia | 2010 | 1.85 (1.37 to 2.29) | 5.78 (2.54 to 11.14) | 74.41 (37.16 to 119.30) | 1.47 (0.67 to 2.48) | 0.588 |
| Southeast Asia | 2011 | 1.76 (1.32 to 2.18) | 5.43 (2.45 to 10.45) | 68.09 (34.85 to 108.25) | 1.36 (0.62 to 2.32) | 0.593 |
| Southeast Asia | 2012 | 1.68 (1.26 to 2.08) | 5.08 (2.35 to 9.70) | 62.50 (31.54 to 99.34) | 1.25 (0.59 to 2.11) | 0.599 |
| Southeast Asia | 2013 | 1.60 (1.20 to 1.98) | 4.75 (2.23 to 8.77) | 57.43 (29.84 to 92.41) | 1.16 (0.56 to 1.95) | 0.606 |
| Southeast Asia | 2014 | 1.52 (1.14 to 1.89) | 4.45 (2.11 to 8.33) | 53.04 (28.12 to 87.93) | 1.08 (0.53 to 1.82) | 0.612 |
| Southeast Asia | 2015 | 1.45 (1.09 to 1.81) | 4.20 (1.99 to 7.90) | 49.83 (27.62 to 80.40) | 1.01 (0.51 to 1.75) | 0.618 |
| Southeast Asia | 2016 | 1.38 (1.05 to 1.72) | 3.95 (1.87 to 7.32) | 45.44 (25.35 to 74.83) | 0.93 (0.48 to 1.62) | 0.624 |
| Southeast Asia | 2017 | 1.30 (0.99 to 1.63) | 3.67 (1.72 to 6.76) | 41.97 (23.45 to 69.68) | 0.86 (0.44 to 1.52) | 0.630 |
| Southeast Asia | 2018 | 1.23 (0.94 to 1.55) | 3.40 (1.58 to 6.36) | 38.59 (21.22 to 63.70) | 0.80 (0.40 to 1.40) | 0.636 |
| Southeast Asia | 2019 | 1.17 (0.88 to 1.49) | 3.16 (1.46 to 6.05) | 35.76 (18.57 to 61.45) | 0.74 (0.36 to 1.31) | 0.642 |
| Southeast Asia | 2020 | 1.13 (0.86 to 1.45) | 3.01 (1.36 to 5.87) | 34.28 (16.66 to 58.92) | 0.71 (0.34 to 1.25) | 0.647 |
| Southeast Asia | 2021 | 1.11 (0.84 to 1.45) | 2.95 (1.34 to 5.88) | 34.99 (17.12 to 61.54) | 0.71 (0.34 to 1.30) | 0.652 |
| Southeast Asia | 2022 | 1.10 (0.83 to 1.43) | 2.90 (1.32 to 5.78) | 32.09 (16.49 to 55.21) | 0.67 (0.31 to 1.21) | 0.657 |
| Southeast Asia | 2023 | 1.08 (0.82 to 1.42) | 2.68 (1.16 to 5.49) | 29.59 (15.49 to 52.33) | 0.61 (0.28 to 1.11) | 0.661 |
| Southern Latin America | 1990 | 0.06 (0.05 to 0.09) | 0.64 (0.44 to 1.04) | 3.85 (2.92 to 4.98) | 0.12 (0.09 to 0.17) | 0.606 |
| Southern Latin America | 1991 | 0.06 (0.05 to 0.08) | 0.58 (0.42 to 0.92) | 3.26 (2.49 to 4.36) | 0.10 (0.07 to 0.15) | 0.611 |
| Southern Latin America | 1992 | 0.06 (0.04 to 0.08) | 0.53 (0.38 to 0.81) | 2.95 (2.21 to 3.86) | 0.09 (0.07 to 0.13) | 0.617 |
| Southern Latin America | 1993 | 0.05 (0.04 to 0.07) | 0.48 (0.34 to 0.70) | 2.72 (2.06 to 3.52) | 0.08 (0.06 to 0.11) | 0.622 |
| Southern Latin America | 1994 | 0.05 (0.04 to 0.06) | 0.44 (0.31 to 0.62) | 2.47 (1.82 to 3.21) | 0.07 (0.05 to 0.10) | 0.628 |
| Southern Latin America | 1995 | 0.05 (0.04 to 0.06) | 0.40 (0.28 to 0.54) | 2.33 (1.73 to 3.05) | 0.07 (0.05 to 0.09) | 0.633 |
| Southern Latin America | 1996 | 0.04 (0.03 to 0.06) | 0.36 (0.25 to 0.50) | 2.21 (1.64 to 2.90) | 0.07 (0.05 to 0.09) | 0.638 |
| Southern Latin America | 1997 | 0.04 (0.03 to 0.05) | 0.32 (0.23 to 0.45) | 1.98 (1.46 to 2.60) | 0.06 (0.04 to 0.08) | 0.644 |
| Southern Latin America | 1998 | 0.03 (0.03 to 0.04) | 0.29 (0.21 to 0.40) | 1.75 (1.28 to 2.29) | 0.05 (0.04 to 0.07) | 0.650 |
| Southern Latin America | 1999 | 0.03 (0.02 to 0.04) | 0.25 (0.18 to 0.37) | 1.54 (1.13 to 2.02) | 0.05 (0.03 to 0.06) | 0.655 |
| Southern Latin America | 2000 | 0.03 (0.02 to 0.04) | 0.23 (0.16 to 0.34) | 1.29 (0.96 to 1.74) | 0.04 (0.03 to 0.05) | 0.660 |
| Southern Latin America | 2001 | 0.02 (0.02 to 0.03) | 0.20 (0.15 to 0.31) | 1.17 (0.88 to 1.61) | 0.04 (0.03 to 0.05) | 0.664 |
| Southern Latin America | 2002 | 0.02 (0.02 to 0.03) | 0.18 (0.13 to 0.28) | 1.12 (0.82 to 1.54) | 0.03 (0.02 to 0.05) | 0.668 |
| Southern Latin America | 2003 | 0.02 (0.01 to 0.03) | 0.16 (0.12 to 0.26) | 1.00 (0.74 to 1.40) | 0.03 (0.02 to 0.05) | 0.670 |
| Southern Latin America | 2004 | 0.02 (0.01 to 0.02) | 0.15 (0.10 to 0.24) | 0.87 (0.63 to 1.25) | 0.03 (0.02 to 0.04) | 0.673 |
| Southern Latin America | 2005 | 0.02 (0.01 to 0.02) | 0.13 (0.09 to 0.23) | 0.72 (0.51 to 1.04) | 0.02 (0.02 to 0.03) | 0.678 |
| Southern Latin America | 2006 | 0.01 (0.01 to 0.02) | 0.12 (0.08 to 0.21) | 0.57 (0.41 to 0.85) | 0.02 (0.01 to 0.03) | 0.681 |
| Southern Latin America | 2007 | 0.01 (0.01 to 0.02) | 0.10 (0.07 to 0.19) | 0.51 (0.36 to 0.78) | 0.02 (0.01 to 0.03) | 0.684 |
| Southern Latin America | 2008 | 0.01 (0.01 to 0.02) | 0.09 (0.06 to 0.18) | 0.44 (0.31 to 0.69) | 0.01 (0.01 to 0.02) | 0.687 |
| Southern Latin America | 2009 | 0.01 (0.01 to 0.02) | 0.08 (0.05 to 0.16) | 0.43 (0.31 to 0.67) | 0.01 (0.01 to 0.02) | 0.691 |
| Southern Latin America | 2010 | 0.01 (0.01 to 0.02) | 0.07 (0.05 to 0.15) | 0.38 (0.27 to 0.59) | 0.01 (0.01 to 0.02) | 0.696 |
| Southern Latin America | 2011 | 0.01 (0.01 to 0.02) | 0.07 (0.04 to 0.15) | 0.31 (0.22 to 0.49) | 0.01 (0.01 to 0.02) | 0.701 |
| Southern Latin America | 2012 | 0.01 (0.01 to 0.01) | 0.06 (0.04 to 0.14) | 0.29 (0.20 to 0.47) | 0.01 (0.01 to 0.02) | 0.705 |
| Southern Latin America | 2013 | 0.01 (0.01 to 0.01) | 0.06 (0.04 to 0.13) | 0.32 (0.22 to 0.50) | 0.01 (0.01 to 0.02) | 0.709 |
| Southern Latin America | 2014 | 0.01 (0.01 to 0.01) | 0.06 (0.03 to 0.12) | 0.31 (0.21 to 0.48) | 0.01 (0.01 to 0.02) | 0.713 |
| Southern Latin America | 2015 | 0.01 (0.01 to 0.01) | 0.05 (0.03 to 0.11) | 0.28 (0.18 to 0.43) | 0.01 (0.01 to 0.01) | 0.720 |
| Southern Latin America | 2016 | 0.01 (0.01 to 0.01) | 0.05 (0.03 to 0.11) | 0.25 (0.17 to 0.39) | 0.01 (0.01 to 0.01) | 0.727 |
| Southern Latin America | 2017 | 0.01 (0.00 to 0.01) | 0.04 (0.03 to 0.11) | 0.20 (0.13 to 0.33) | 0.01 (0.00 to 0.01) | 0.734 |
| Southern Latin America | 2018 | 0.01 (0.00 to 0.01) | 0.03 (0.02 to 0.10) | 0.16 (0.11 to 0.28) | 0.00 (0.00 to 0.01) | 0.743 |
| Southern Latin America | 2019 | 0.01 (0.00 to 0.01) | 0.03 (0.02 to 0.09) | 0.13 (0.08 to 0.23) | 0.00 (0.00 to 0.01) | 0.753 |
| Southern Latin America | 2020 | 0.01 (0.00 to 0.01) | 0.02 (0.01 to 0.09) | 0.11 (0.07 to 0.21) | 0.00 (0.00 to 0.01) | 0.759 |
| Southern Latin America | 2021 | 0.01 (0.00 to 0.01) | 0.02 (0.01 to 0.10) | 0.09 (0.06 to 0.19) | 0.00 (0.00 to 0.01) | 0.764 |
| Southern Latin America | 2022 | 0.01 (0.00 to 0.01) | 0.02 (0.01 to 0.10) | 0.10 (0.06 to 0.20) | 0.00 (0.00 to 0.01) | 0.768 |
| Southern Latin America | 2023 | 0.01 (0.00 to 0.01) | 0.02 (0.01 to 0.09) | 0.09 (0.06 to 0.19) | 0.00 (0.00 to 0.01) | 0.770 |
| Southern Sub-Saharan Africa | 1990 | 0.22 (0.17 to 0.30) | 0.99 (0.52 to 1.82) | 16.30 (9.40 to 25.44) | 0.31 (0.18 to 0.52) | 0.514 |
| Southern Sub-Saharan Africa | 1991 | 0.22 (0.16 to 0.29) | 0.92 (0.49 to 1.72) | 14.51 (8.47 to 23.04) | 0.28 (0.16 to 0.46) | 0.520 |
| Southern Sub-Saharan Africa | 1992 | 0.21 (0.16 to 0.28) | 0.85 (0.47 to 1.53) | 13.24 (7.87 to 20.83) | 0.25 (0.15 to 0.42) | 0.525 |
| Southern Sub-Saharan Africa | 1993 | 0.21 (0.16 to 0.27) | 0.80 (0.44 to 1.47) | 12.22 (7.47 to 18.92) | 0.24 (0.14 to 0.39) | 0.531 |
| Southern Sub-Saharan Africa | 1994 | 0.20 (0.15 to 0.26) | 0.75 (0.43 to 1.45) | 11.92 (7.58 to 18.77) | 0.23 (0.15 to 0.39) | 0.536 |
| Southern Sub-Saharan Africa | 1995 | 0.20 (0.15 to 0.26) | 0.73 (0.42 to 1.41) | 11.53 (7.58 to 17.92) | 0.23 (0.14 to 0.38) | 0.541 |
| Southern Sub-Saharan Africa | 1996 | 0.19 (0.15 to 0.25) | 0.71 (0.42 to 1.37) | 11.49 (7.75 to 18.26) | 0.23 (0.15 to 0.39) | 0.547 |
| Southern Sub-Saharan Africa | 1997 | 0.19 (0.15 to 0.24) | 0.70 (0.42 to 1.35) | 11.44 (7.79 to 18.40) | 0.24 (0.15 to 0.40) | 0.552 |
| Southern Sub-Saharan Africa | 1998 | 0.18 (0.14 to 0.23) | 0.69 (0.42 to 1.36) | 11.19 (7.76 to 18.29) | 0.23 (0.15 to 0.40) | 0.557 |
| Southern Sub-Saharan Africa | 1999 | 0.18 (0.14 to 0.23) | 0.68 (0.42 to 1.39) | 10.51 (7.25 to 17.26) | 0.22 (0.15 to 0.38) | 0.561 |
| Southern Sub-Saharan Africa | 2000 | 0.17 (0.14 to 0.23) | 0.68 (0.41 to 1.41) | 10.04 (6.83 to 16.59) | 0.21 (0.14 to 0.37) | 0.566 |
| Southern Sub-Saharan Africa | 2001 | 0.17 (0.13 to 0.23) | 0.68 (0.43 to 1.45) | 9.93 (6.68 to 16.56) | 0.21 (0.14 to 0.37) | 0.571 |
| Southern Sub-Saharan Africa | 2002 | 0.17 (0.13 to 0.22) | 0.69 (0.44 to 1.52) | 9.98 (6.69 to 17.04) | 0.21 (0.14 to 0.38) | 0.574 |
| Southern Sub-Saharan Africa | 2003 | 0.17 (0.13 to 0.22) | 0.70 (0.43 to 1.54) | 10.05 (6.75 to 17.26) | 0.21 (0.14 to 0.38) | 0.578 |
| Southern Sub-Saharan Africa | 2004 | 0.17 (0.13 to 0.22) | 0.71 (0.42 to 1.56) | 10.20 (6.78 to 17.51) | 0.21 (0.14 to 0.38) | 0.582 |
| Southern Sub-Saharan Africa | 2005 | 0.16 (0.13 to 0.23) | 0.71 (0.41 to 1.53) | 10.33 (6.97 to 17.62) | 0.21 (0.14 to 0.38) | 0.585 |
| Southern Sub-Saharan Africa | 2006 | 0.16 (0.13 to 0.22) | 0.70 (0.42 to 1.52) | 10.27 (7.11 to 17.30) | 0.21 (0.14 to 0.38) | 0.589 |
| Southern Sub-Saharan Africa | 2007 | 0.16 (0.13 to 0.22) | 0.69 (0.42 to 1.50) | 10.04 (6.92 to 16.72) | 0.21 (0.14 to 0.36) | 0.594 |
| Southern Sub-Saharan Africa | 2008 | 0.16 (0.13 to 0.22) | 0.68 (0.41 to 1.49) | 10.09 (6.95 to 16.89) | 0.21 (0.14 to 0.37) | 0.599 |
| Southern Sub-Saharan Africa | 2009 | 0.16 (0.13 to 0.21) | 0.66 (0.40 to 1.45) | 9.93 (6.81 to 16.63) | 0.21 (0.14 to 0.37) | 0.603 |
| Southern Sub-Saharan Africa | 2010 | 0.16 (0.13 to 0.21) | 0.65 (0.39 to 1.41) | 9.53 (6.35 to 16.16) | 0.20 (0.13 to 0.36) | 0.607 |
| Southern Sub-Saharan Africa | 2011 | 0.16 (0.12 to 0.21) | 0.63 (0.38 to 1.37) | 9.09 (6.01 to 15.43) | 0.19 (0.12 to 0.34) | 0.612 |
| Southern Sub-Saharan Africa | 2012 | 0.15 (0.12 to 0.20) | 0.61 (0.36 to 1.32) | 8.60 (5.65 to 14.41) | 0.19 (0.12 to 0.32) | 0.618 |
| Southern Sub-Saharan Africa | 2013 | 0.15 (0.12 to 0.20) | 0.58 (0.35 to 1.26) | 8.08 (5.29 to 13.32) | 0.17 (0.11 to 0.30) | 0.623 |
| Southern Sub-Saharan Africa | 2014 | 0.15 (0.12 to 0.19) | 0.55 (0.31 to 1.25) | 7.63 (5.04 to 12.52) | 0.17 (0.10 to 0.28) | 0.629 |
| Southern Sub-Saharan Africa | 2015 | 0.14 (0.12 to 0.19) | 0.53 (0.29 to 1.23) | 7.10 (4.63 to 11.69) | 0.16 (0.10 to 0.26) | 0.634 |
| Southern Sub-Saharan Africa | 2016 | 0.14 (0.11 to 0.18) | 0.50 (0.27 to 1.16) | 6.64 (4.24 to 11.03) | 0.15 (0.09 to 0.25) | 0.639 |
| Southern Sub-Saharan Africa | 2017 | 0.14 (0.11 to 0.17) | 0.44 (0.24 to 1.03) | 5.97 (3.86 to 9.92) | 0.13 (0.08 to 0.22) | 0.644 |
| Southern Sub-Saharan Africa | 2018 | 0.13 (0.11 to 0.17) | 0.39 (0.21 to 0.89) | 5.47 (3.55 to 9.05) | 0.12 (0.07 to 0.20) | 0.649 |
| Southern Sub-Saharan Africa | 2019 | 0.13 (0.11 to 0.16) | 0.34 (0.18 to 0.75) | 4.97 (3.27 to 8.24) | 0.11 (0.07 to 0.18) | 0.653 |
| Southern Sub-Saharan Africa | 2020 | 0.13 (0.11 to 0.15) | 0.30 (0.16 to 0.68) | 4.61 (3.02 to 7.60) | 0.09 (0.06 to 0.16) | 0.656 |
| Southern Sub-Saharan Africa | 2021 | 0.12 (0.10 to 0.15) | 0.28 (0.14 to 0.62) | 4.55 (2.85 to 7.47) | 0.09 (0.05 to 0.16) | 0.659 |
| Southern Sub-Saharan Africa | 2022 | 0.12 (0.09 to 0.14) | 0.27 (0.13 to 0.59) | 4.16 (2.59 to 6.82) | 0.09 (0.05 to 0.15) | 0.663 |
| Southern Sub-Saharan Africa | 2023 | 0.12 (0.10 to 0.14) | 0.26 (0.13 to 0.61) | 3.87 (2.42 to 6.30) | 0.08 (0.05 to 0.14) | 0.666 |
| Tropical Latin America | 1990 | 0.43 (0.33 to 0.55) | 2.07 (1.64 to 2.83) | 41.70 (34.25 to 51.47) | 0.83 (0.68 to 1.05) | 0.512 |
| Tropical Latin America | 1991 | 0.41 (0.31 to 0.52) | 1.88 (1.50 to 2.56) | 36.54 (30.08 to 44.87) | 0.73 (0.61 to 0.92) | 0.519 |
| Tropical Latin America | 1992 | 0.38 (0.29 to 0.49) | 1.71 (1.37 to 2.29) | 32.72 (27.09 to 40.32) | 0.66 (0.55 to 0.83) | 0.524 |
| Tropical Latin America | 1993 | 0.36 (0.27 to 0.46) | 1.54 (1.24 to 2.06) | 29.20 (24.39 to 36.09) | 0.60 (0.50 to 0.75) | 0.530 |
| Tropical Latin America | 1994 | 0.33 (0.25 to 0.43) | 1.38 (1.11 to 1.88) | 25.56 (21.52 to 31.49) | 0.53 (0.44 to 0.66) | 0.535 |
| Tropical Latin America | 1995 | 0.31 (0.23 to 0.40) | 1.23 (0.98 to 1.71) | 22.05 (18.29 to 27.26) | 0.46 (0.38 to 0.58) | 0.541 |
| Tropical Latin America | 1996 | 0.28 (0.21 to 0.36) | 1.09 (0.86 to 1.50) | 18.75 (15.64 to 23.21) | 0.40 (0.33 to 0.50) | 0.546 |
| Tropical Latin America | 1997 | 0.25 (0.19 to 0.32) | 0.93 (0.74 to 1.26) | 15.83 (13.15 to 19.54) | 0.34 (0.28 to 0.43) | 0.551 |
| Tropical Latin America | 1998 | 0.22 (0.17 to 0.28) | 0.78 (0.62 to 1.06) | 13.43 (11.18 to 16.54) | 0.29 (0.24 to 0.36) | 0.556 |
| Tropical Latin America | 1999 | 0.19 (0.15 to 0.25) | 0.66 (0.52 to 0.88) | 11.38 (9.34 to 13.90) | 0.25 (0.20 to 0.30) | 0.561 |
| Tropical Latin America | 2000 | 0.17 (0.13 to 0.22) | 0.57 (0.44 to 0.76) | 9.41 (7.67 to 11.54) | 0.20 (0.16 to 0.25) | 0.567 |
| Tropical Latin America | 2001 | 0.16 (0.12 to 0.20) | 0.50 (0.39 to 0.67) | 8.16 (6.63 to 10.15) | 0.18 (0.14 to 0.22) | 0.573 |
| Tropical Latin America | 2002 | 0.14 (0.11 to 0.18) | 0.45 (0.35 to 0.60) | 7.16 (5.76 to 8.91) | 0.16 (0.13 to 0.20) | 0.579 |
| Tropical Latin America | 2003 | 0.13 (0.10 to 0.17) | 0.40 (0.31 to 0.54) | 6.29 (5.06 to 7.75) | 0.14 (0.11 to 0.17) | 0.584 |
| Tropical Latin America | 2004 | 0.12 (0.10 to 0.16) | 0.36 (0.27 to 0.49) | 5.56 (4.47 to 6.85) | 0.13 (0.10 to 0.16) | 0.590 |
| Tropical Latin America | 2005 | 0.12 (0.09 to 0.15) | 0.33 (0.24 to 0.45) | 4.91 (3.89 to 6.09) | 0.11 (0.09 to 0.14) | 0.597 |
| Tropical Latin America | 2006 | 0.11 (0.09 to 0.14) | 0.30 (0.22 to 0.40) | 4.42 (3.46 to 5.46) | 0.10 (0.08 to 0.13) | 0.603 |
| Tropical Latin America | 2007 | 0.11 (0.08 to 0.14) | 0.27 (0.20 to 0.36) | 3.98 (3.10 to 5.01) | 0.09 (0.07 to 0.12) | 0.609 |
| Tropical Latin America | 2008 | 0.10 (0.08 to 0.13) | 0.24 (0.18 to 0.33) | 3.58 (2.76 to 4.56) | 0.09 (0.06 to 0.11) | 0.616 |
| Tropical Latin America | 2009 | 0.10 (0.07 to 0.13) | 0.22 (0.16 to 0.30) | 3.20 (2.45 to 4.10) | 0.08 (0.06 to 0.10) | 0.622 |
| Tropical Latin America | 2010 | 0.10 (0.07 to 0.13) | 0.20 (0.14 to 0.28) | 2.90 (2.23 to 3.68) | 0.07 (0.05 to 0.09) | 0.628 |
| Tropical Latin America | 2011 | 0.09 (0.07 to 0.13) | 0.18 (0.13 to 0.25) | 2.59 (2.02 to 3.23) | 0.06 (0.05 to 0.08) | 0.635 |
| Tropical Latin America | 2012 | 0.10 (0.07 to 0.13) | 0.17 (0.12 to 0.23) | 2.32 (1.80 to 2.89) | 0.06 (0.04 to 0.07) | 0.640 |
| Tropical Latin America | 2013 | 0.10 (0.07 to 0.13) | 0.15 (0.11 to 0.21) | 2.11 (1.65 to 2.67) | 0.05 (0.04 to 0.07) | 0.645 |
| Tropical Latin America | 2014 | 0.10 (0.07 to 0.13) | 0.14 (0.10 to 0.19) | 1.92 (1.54 to 2.40) | 0.05 (0.04 to 0.06) | 0.650 |
| Tropical Latin America | 2015 | 0.10 (0.07 to 0.13) | 0.13 (0.09 to 0.19) | 1.78 (1.43 to 2.30) | 0.05 (0.04 to 0.06) | 0.654 |
| Tropical Latin America | 2016 | 0.09 (0.07 to 0.13) | 0.12 (0.09 to 0.18) | 1.69 (1.33 to 2.25) | 0.04 (0.03 to 0.06) | 0.657 |
| Tropical Latin America | 2017 | 0.09 (0.07 to 0.12) | 0.11 (0.08 to 0.17) | 1.58 (1.24 to 2.16) | 0.04 (0.03 to 0.06) | 0.661 |
| Tropical Latin America | 2018 | 0.08 (0.06 to 0.11) | 0.11 (0.08 to 0.17) | 1.46 (1.10 to 2.04) | 0.04 (0.03 to 0.05) | 0.664 |
| Tropical Latin America | 2019 | 0.08 (0.06 to 0.10) | 0.10 (0.07 to 0.17) | 1.35 (1.01 to 1.96) | 0.04 (0.03 to 0.05) | 0.668 |
| Tropical Latin America | 2020 | 0.07 (0.05 to 0.10) | 0.10 (0.06 to 0.18) | 1.25 (0.92 to 1.89) | 0.03 (0.02 to 0.05) | 0.671 |
| Tropical Latin America | 2021 | 0.06 (0.04 to 0.09) | 0.09 (0.06 to 0.19) | 1.15 (0.83 to 1.81) | 0.03 (0.02 to 0.05) | 0.675 |
| Tropical Latin America | 2022 | 0.05 (0.03 to 0.09) | 0.09 (0.06 to 0.20) | 1.09 (0.79 to 1.75) | 0.03 (0.02 to 0.05) | 0.679 |
| Tropical Latin America | 2023 | 0.05 (0.03 to 0.09) | 0.08 (0.05 to 0.18) | 1.07 (0.77 to 1.73) | 0.03 (0.02 to 0.05) | 0.684 |
| Western Europe | 1990 | 0.01 (0.01 to 0.01) | 0.13 (0.09 to 0.17) | 0.67 (0.45 to 0.84) | 0.03 (0.02 to 0.04) | 0.754 |
| Western Europe | 1991 | 0.01 (0.01 to 0.01) | 0.12 (0.09 to 0.16) | 0.60 (0.41 to 0.76) | 0.03 (0.02 to 0.03) | 0.759 |
| Western Europe | 1992 | 0.01 (0.01 to 0.01) | 0.11 (0.08 to 0.15) | 0.55 (0.37 to 0.70) | 0.02 (0.02 to 0.03) | 0.765 |
| Western Europe | 1993 | 0.01 (0.01 to 0.01) | 0.11 (0.07 to 0.14) | 0.52 (0.33 to 0.66) | 0.02 (0.02 to 0.03) | 0.770 |
| Western Europe | 1994 | 0.01 (0.01 to 0.01) | 0.10 (0.06 to 0.14) | 0.47 (0.30 to 0.60) | 0.02 (0.01 to 0.03) | 0.775 |
| Western Europe | 1995 | 0.01 (0.00 to 0.01) | 0.09 (0.06 to 0.13) | 0.44 (0.28 to 0.57) | 0.02 (0.01 to 0.02) | 0.779 |
| Western Europe | 1996 | 0.01 (0.00 to 0.01) | 0.08 (0.05 to 0.12) | 0.40 (0.25 to 0.52) | 0.02 (0.01 to 0.02) | 0.783 |
| Western Europe | 1997 | 0.01 (0.00 to 0.01) | 0.07 (0.05 to 0.10) | 0.36 (0.23 to 0.47) | 0.02 (0.01 to 0.02) | 0.786 |
| Western Europe | 1998 | 0.00 (0.00 to 0.01) | 0.06 (0.04 to 0.08) | 0.31 (0.20 to 0.40) | 0.01 (0.01 to 0.02) | 0.790 |
| Western Europe | 1999 | 0.00 (0.00 to 0.01) | 0.05 (0.04 to 0.07) | 0.25 (0.17 to 0.33) | 0.01 (0.01 to 0.02) | 0.793 |
| Western Europe | 2000 | 0.00 (0.00 to 0.00) | 0.04 (0.03 to 0.06) | 0.21 (0.15 to 0.28) | 0.01 (0.01 to 0.01) | 0.796 |
| Western Europe | 2001 | 0.00 (0.00 to 0.00) | 0.04 (0.03 to 0.05) | 0.17 (0.12 to 0.23) | 0.01 (0.01 to 0.01) | 0.800 |
| Western Europe | 2002 | 0.00 (0.00 to 0.00) | 0.03 (0.02 to 0.05) | 0.15 (0.11 to 0.20) | 0.01 (0.01 to 0.01) | 0.803 |
| Western Europe | 2003 | 0.00 (0.00 to 0.00) | 0.03 (0.02 to 0.04) | 0.13 (0.10 to 0.19) | 0.01 (0.00 to 0.01) | 0.806 |
| Western Europe | 2004 | 0.00 (0.00 to 0.00) | 0.02 (0.02 to 0.03) | 0.12 (0.09 to 0.17) | 0.01 (0.00 to 0.01) | 0.809 |
| Western Europe | 2005 | 0.00 (0.00 to 0.00) | 0.02 (0.01 to 0.03) | 0.11 (0.08 to 0.15) | 0.01 (0.00 to 0.01) | 0.811 |
| Western Europe | 2006 | 0.00 (0.00 to 0.00) | 0.02 (0.01 to 0.03) | 0.10 (0.07 to 0.13) | 0.00 (0.00 to 0.01) | 0.814 |
| Western Europe | 2007 | 0.00 (0.00 to 0.00) | 0.02 (0.01 to 0.03) | 0.09 (0.07 to 0.12) | 0.00 (0.00 to 0.01) | 0.817 |
| Western Europe | 2008 | 0.00 (0.00 to 0.00) | 0.02 (0.01 to 0.03) | 0.09 (0.06 to 0.12) | 0.00 (0.00 to 0.01) | 0.819 |
| Western Europe | 2009 | 0.00 (0.00 to 0.00) | 0.02 (0.01 to 0.02) | 0.09 (0.06 to 0.12) | 0.00 (0.00 to 0.01) | 0.822 |
| Western Europe | 2010 | 0.00 (0.00 to 0.00) | 0.01 (0.01 to 0.02) | 0.08 (0.06 to 0.10) | 0.00 (0.00 to 0.01) | 0.825 |
| Western Europe | 2011 | 0.00 (0.00 to 0.00) | 0.01 (0.01 to 0.02) | 0.07 (0.05 to 0.09) | 0.00 (0.00 to 0.00) | 0.829 |
| Western Europe | 2012 | 0.00 (0.00 to 0.00) | 0.01 (0.01 to 0.02) | 0.07 (0.05 to 0.09) | 0.00 (0.00 to 0.00) | 0.832 |
| Western Europe | 2013 | 0.00 (0.00 to 0.00) | 0.01 (0.01 to 0.02) | 0.06 (0.04 to 0.08) | 0.00 (0.00 to 0.00) | 0.835 |
| Western Europe | 2014 | 0.00 (0.00 to 0.00) | 0.01 (0.01 to 0.02) | 0.06 (0.04 to 0.07) | 0.00 (0.00 to 0.00) | 0.838 |
| Western Europe | 2015 | 0.00 (0.00 to 0.00) | 0.01 (0.01 to 0.02) | 0.06 (0.04 to 0.08) | 0.00 (0.00 to 0.00) | 0.840 |
| Western Europe | 2016 | 0.00 (0.00 to 0.00) | 0.01 (0.01 to 0.02) | 0.06 (0.04 to 0.08) | 0.00 (0.00 to 0.00) | 0.843 |
| Western Europe | 2017 | 0.00 (0.00 to 0.00) | 0.01 (0.01 to 0.02) | 0.06 (0.04 to 0.08) | 0.00 (0.00 to 0.00) | 0.847 |
| Western Europe | 2018 | 0.00 (0.00 to 0.00) | 0.01 (0.01 to 0.02) | 0.06 (0.04 to 0.08) | 0.00 (0.00 to 0.00) | 0.850 |
| Western Europe | 2019 | 0.00 (0.00 to 0.00) | 0.01 (0.01 to 0.02) | 0.06 (0.04 to 0.08) | 0.00 (0.00 to 0.00) | 0.853 |
| Western Europe | 2020 | 0.00 (0.00 to 0.00) | 0.01 (0.01 to 0.02) | 0.06 (0.04 to 0.08) | 0.00 (0.00 to 0.00) | 0.856 |
| Western Europe | 2021 | 0.00 (0.00 to 0.00) | 0.01 (0.01 to 0.02) | 0.06 (0.03 to 0.08) | 0.00 (0.00 to 0.00) | 0.859 |
| Western Europe | 2022 | 0.00 (0.00 to 0.00) | 0.01 (0.01 to 0.02) | 0.06 (0.03 to 0.08) | 0.00 (0.00 to 0.00) | 0.862 |
| Western Europe | 2023 | 0.00 (0.00 to 0.00) | 0.01 (0.01 to 0.02) | 0.05 (0.03 to 0.08) | 0.00 (0.00 to 0.00) | 0.865 |
| Western Sub-Saharan Africa | 1990 | 3.41 (2.43 to 4.55) | 13.32 (6.21 to 23.90) | 438.91 (198.73 to 703.69) | 6.63 (3.17 to 10.70) | 0.271 |
| Western Sub-Saharan Africa | 1991 | 3.42 (2.44 to 4.53) | 12.62 (6.11 to 22.01) | 409.88 (188.90 to 661.33) | 6.18 (2.94 to 10.24) | 0.275 |
| Western Sub-Saharan Africa | 1992 | 3.41 (2.44 to 4.50) | 11.96 (5.82 to 20.84) | 386.32 (181.86 to 628.27) | 5.81 (2.85 to 9.82) | 0.279 |
| Western Sub-Saharan Africa | 1993 | 3.39 (2.42 to 4.45) | 11.37 (5.62 to 19.58) | 370.75 (178.29 to 606.65) | 5.57 (2.83 to 9.07) | 0.283 |
| Western Sub-Saharan Africa | 1994 | 3.36 (2.38 to 4.42) | 10.88 (5.14 to 18.90) | 360.89 (172.49 to 594.25) | 5.43 (2.73 to 8.84) | 0.287 |
| Western Sub-Saharan Africa | 1995 | 3.34 (2.34 to 4.42) | 10.51 (4.84 to 18.52) | 351.26 (166.57 to 584.71) | 5.31 (2.72 to 8.49) | 0.290 |
| Western Sub-Saharan Africa | 1996 | 3.27 (2.29 to 4.32) | 10.22 (4.94 to 18.07) | 341.71 (166.67 to 562.27) | 5.20 (2.64 to 8.43) | 0.294 |
| Western Sub-Saharan Africa | 1997 | 3.13 (2.22 to 4.15) | 9.97 (4.93 to 17.97) | 331.54 (165.85 to 536.44) | 5.08 (2.46 to 8.28) | 0.297 |
| Western Sub-Saharan Africa | 1998 | 2.95 (2.11 to 3.90) | 9.74 (4.63 to 17.65) | 323.35 (157.73 to 545.98) | 4.98 (2.36 to 8.31) | 0.301 |
| Western Sub-Saharan Africa | 1999 | 2.77 (1.97 to 3.67) | 9.49 (4.47 to 17.26) | 311.81 (154.09 to 536.79) | 4.83 (2.33 to 8.29) | 0.304 |
| Western Sub-Saharan Africa | 2000 | 2.62 (1.86 to 3.48) | 9.18 (4.38 to 17.52) | 295.63 (143.43 to 522.26) | 4.63 (2.30 to 7.98) | 0.308 |
| Western Sub-Saharan Africa | 2001 | 2.49 (1.77 to 3.32) | 8.74 (4.20 to 16.85) | 276.62 (141.20 to 491.33) | 4.37 (2.19 to 7.56) | 0.312 |
| Western Sub-Saharan Africa | 2002 | 2.35 (1.66 to 3.12) | 8.12 (4.04 to 15.72) | 255.85 (126.81 to 460.28) | 4.08 (2.02 to 7.16) | 0.317 |
| Western Sub-Saharan Africa | 2003 | 2.21 (1.55 to 2.92) | 7.43 (3.62 to 14.25) | 234.16 (111.38 to 416.47) | 3.76 (1.80 to 6.55) | 0.323 |
| Western Sub-Saharan Africa | 2004 | 2.07 (1.46 to 2.75) | 6.77 (3.22 to 12.88) | 215.30 (104.68 to 381.35) | 3.47 (1.69 to 6.09) | 0.328 |
| Western Sub-Saharan Africa | 2005 | 1.97 (1.39 to 2.62) | 6.22 (2.91 to 11.87) | 194.68 (96.09 to 350.02) | 3.15 (1.57 to 5.42) | 0.335 |
| Western Sub-Saharan Africa | 2006 | 1.87 (1.32 to 2.47) | 5.77 (2.90 to 11.19) | 177.20 (91.01 to 311.17) | 2.89 (1.48 to 4.88) | 0.341 |
| Western Sub-Saharan Africa | 2007 | 1.79 (1.26 to 2.34) | 5.31 (2.63 to 10.56) | 160.23 (83.55 to 276.43) | 2.62 (1.33 to 4.39) | 0.348 |
| Western Sub-Saharan Africa | 2008 | 1.71 (1.21 to 2.22) | 4.87 (2.34 to 9.79) | 144.75 (72.37 to 242.93) | 2.39 (1.19 to 4.04) | 0.354 |
| Western Sub-Saharan Africa | 2009 | 1.63 (1.16 to 2.13) | 4.48 (2.11 to 8.95) | 131.24 (66.73 to 210.26) | 2.18 (1.10 to 3.67) | 0.361 |
| Western Sub-Saharan Africa | 2010 | 1.57 (1.11 to 2.04) | 4.17 (1.98 to 8.65) | 120.44 (62.39 to 190.91) | 2.02 (1.02 to 3.40) | 0.368 |
| Western Sub-Saharan Africa | 2011 | 1.51 (1.07 to 1.97) | 3.91 (1.92 to 7.95) | 112.02 (58.28 to 176.58) | 1.88 (0.98 to 3.18) | 0.375 |
| Western Sub-Saharan Africa | 2012 | 1.45 (1.04 to 1.91) | 3.64 (1.85 to 7.25) | 104.96 (57.41 to 160.99) | 1.77 (0.94 to 2.97) | 0.382 |
| Western Sub-Saharan Africa | 2013 | 1.40 (1.01 to 1.85) | 3.38 (1.76 to 6.58) | 98.32 (53.35 to 154.54) | 1.67 (0.90 to 2.77) | 0.389 |
| Western Sub-Saharan Africa | 2014 | 1.35 (0.97 to 1.76) | 3.13 (1.64 to 6.01) | 91.46 (50.99 to 144.45) | 1.56 (0.86 to 2.60) | 0.396 |
| Western Sub-Saharan Africa | 2015 | 1.29 (0.93 to 1.68) | 2.89 (1.53 to 5.45) | 83.81 (47.53 to 136.07) | 1.43 (0.79 to 2.44) | 0.404 |
| Western Sub-Saharan Africa | 2016 | 1.22 (0.88 to 1.58) | 2.67 (1.45 to 4.87) | 76.40 (43.27 to 127.63) | 1.32 (0.73 to 2.31) | 0.411 |
| Western Sub-Saharan Africa | 2017 | 1.14 (0.82 to 1.48) | 2.43 (1.34 to 4.32) | 68.83 (39.42 to 115.21) | 1.20 (0.68 to 2.09) | 0.418 |
| Western Sub-Saharan Africa | 2018 | 1.05 (0.75 to 1.37) | 2.19 (1.18 to 3.78) | 61.63 (35.29 to 101.92) | 1.08 (0.61 to 1.90) | 0.425 |
| Western Sub-Saharan Africa | 2019 | 0.98 (0.70 to 1.28) | 1.97 (1.03 to 3.35) | 55.00 (31.33 to 91.66) | 0.98 (0.52 to 1.67) | 0.433 |
| Western Sub-Saharan Africa | 2020 | 0.93 (0.66 to 1.21) | 1.77 (0.89 to 3.09) | 49.54 (26.93 to 81.67) | 0.88 (0.45 to 1.48) | 0.439 |
| Western Sub-Saharan Africa | 2021 | 0.89 (0.62 to 1.16) | 1.61 (0.79 to 2.79) | 45.17 (24.13 to 75.47) | 0.81 (0.41 to 1.36) | 0.446 |
| Western Sub-Saharan Africa | 2022 | 0.86 (0.60 to 1.14) | 1.46 (0.72 to 2.59) | 40.52 (21.80 to 67.66) | 0.72 (0.37 to 1.23) | 0.453 |
| Western Sub-Saharan Africa | 2023 | 0.85 (0.60 to 1.13) | 1.35 (0.67 to 2.46) | 36.75 (19.55 to 61.39) | 0.66 (0.35 to 1.13) | 0.460 |

DALY= disability-adjusted life year; SDI=Socio-demographic Index; UI= uncertainty interval.

**Table S11.** Age-specific prevalence and incidence rate (95% UI) per 100,000 population by sex in 1990 and 2023.

|  | **Age-specific prevalence rate (95% UI)** | | | | **Age-specific incidence rate (95% UI)** | | |
| --- | --- | --- | --- | --- | --- | --- | --- |
|  | | 1990 | 2023 | Total percentage change (%) | 1990 | 2023 | Total percentage change (%) |
| **Male** | |  |  |  |  |  |  |
| <28 days | | 233.39 (136.87 to 386.99) | 6.94 (3.07 to 15.26) | -97.0 (-99.0 to -93.0) | 4,187.69 (2,568.50 to 6,853.34) | 123.50 (54.62 to 280.79) | -97.0 (-99.0 to -93.0) |
| 1-5 months | | 7.79 (4.60 to 14.52) | 0.85 (0.64 to 1.16) | -89.0 (-94.0 to -82.0) | 95.26 (39.89 to 215.51) | 2.75 (0.94 to 6.66) | -97.0 (-99.0 to -91.0) |
| 6-11 months | | 4.68 (3.15 to 7.41) | 0.76 (0.56 to 0.98) | -84.0 (-89.0 to -77.0) | 39.62 (17.85 to 85.87) | 1.26 (0.44 to 3.12) | -97.0 (-99.0 to -90.0) |
| 12-23 months | | 2.66 (1.89 to 3.46) | 0.69 (0.50 to 0.89) | -74.0 (-78.0 to -71.0) | 3.56 (0.74 to 10.42) | 0.31 (0.04 to 1.34) | -91.0 (-99.0 to -51.0) |
| 2-4 years | | 2.80 (1.93 to 3.86) | 0.68 (0.49 to 0.89) | -76.0 (-81.0 to -71.0) | 6.29 (1.33 to 18.73) | 0.54 (0.07 to 2.29) | -91.0 (-99.0 to -50.0) |
| 5-9 years | | 2.79 (1.98 to 3.53) | 0.65 (0.47 to 0.84) | -77.0 (-80.0 to -74.0) | 6.07 (2.30 to 12.40) | 0.51 (0.15 to 1.22) | -92.0 (-97.0 to -77.0) |
| 10-14 years | | 2.60 (1.85 to 3.29) | 0.63 (0.46 to 0.82) | -76.0 (-78.0 to -73.0) | 3.67 (1.38 to 9.30) | 0.46 (0.15 to 1.11) | -87.0 (-96.0 to -66.0) |
| 15-19 years | | 2.58 (1.82 to 3.30) | 0.62 (0.45 to 0.80) | -76.0 (-78.0 to -73.0) | 3.13 (1.13 to 7.02) | 0.45 (0.15 to 1.05) | -86.0 (-95.0 to -62.0) |
| 20-24 years | | 2.54 (1.82 to 3.27) | 0.59 (0.42 to 0.77) | -77.0 (-79.0 to -74.0) | 2.46 (0.92 to 5.50) | 0.36 (0.11 to 0.83) | -85.0 (-95.0 to -61.0) |
| 25-29 years | | 2.42 (1.75 to 3.12) | 0.56 (0.40 to 0.73) | -77.0 (-79.0 to -74.0) | 2.59 (0.98 to 6.11) | 0.44 (0.14 to 1.10) | -83.0 (-94.0 to -55.0) |
| 30-34 years | | 2.38 (1.72 to 3.14) | 0.52 (0.39 to 0.68) | -78.0 (-80.0 to -75.0) | 3.50 (1.10 to 9.20) | 0.53 (0.19 to 1.57) | -85.0 (-94.0 to -56.0) |
| 35-39 years | | 2.44 (1.78 to 3.11) | 0.50 (0.36 to 0.64) | -80.0 (-82.0 to -77.0) | 3.85 (1.40 to 8.98) | 0.55 (0.18 to 1.29) | -86.0 (-95.0 to -59.0) |
| 40-44 years | | 2.40 (1.73 to 3.16) | 0.49 (0.36 to 0.65) | -80.0 (-82.0 to -77.0) | 4.78 (1.60 to 10.87) | 0.70 (0.23 to 1.60) | -85.0 (-94.0 to -59.0) |
| 45-49 years | | 2.52 (1.83 to 3.36) | 0.48 (0.36 to 0.64) | -81.0 (-84.0 to -77.0) | 6.76 (2.19 to 15.30) | 0.99 (0.31 to 2.29) | -85.0 (-94.0 to -59.0) |
| 50-54 years | | 2.44 (1.77 to 3.29) | 0.45 (0.32 to 0.60) | -82.0 (-85.0 to -78.0) | 7.32 (2.45 to 19.27) | 0.97 (0.31 to 2.30) | -87.0 (-95.0 to -66.0) |
| 55-59 years | | 2.49 (1.80 to 3.33) | 0.43 (0.30 to 0.59) | -83.0 (-86.0 to -79.0) | 8.66 (2.71 to 22.24) | 1.11 (0.34 to 2.69) | -87.0 (-95.0 to -63.0) |
| 60-64 years | | 2.35 (1.74 to 3.15) | 0.40 (0.28 to 0.54) | -83.0 (-87.0 to -79.0) | 8.40 (3.10 to 19.92) | 1.03 (0.39 to 2.08) | -88.0 (-95.0 to -63.0) |
| 65-69 years | | 2.36 (1.67 to 3.31) | 0.38 (0.26 to 0.53) | -84.0 (-87.0 to -80.0) | 9.66 (3.53 to 26.73) | 1.14 (0.47 to 2.92) | -88.0 (-95.0 to -70.0) |
| 70-74 years | | 2.41 (1.74 to 3.47) | 0.35 (0.24 to 0.51) | -85.0 (-89.0 to -81.0) | 11.35 (4.20 to 29.04) | 1.20 (0.46 to 2.87) | -89.0 (-96.0 to -71.0) |
| 75-79 years | | 2.15 (1.58 to 3.09) | 0.34 (0.23 to 0.49) | -84.0 (-88.0 to -80.0) | 11.39 (3.83 to 26.08) | 1.32 (0.47 to 3.13) | -88.0 (-96.0 to -69.0) |
| 80-84 years | | 2.00 (1.43 to 2.77) | 0.31 (0.21 to 0.44) | -84.0 (-88.0 to -80.0) | 10.87 (4.62 to 22.57) | 1.21 (0.43 to 2.57) | -89.0 (-96.0 to -72.0) |
| 85+ years | | 1.98 (1.40 to 2.83) | 0.30 (0.20 to 0.43) | -85.0 (-89.0 to -79.0) | 13.68 (6.07 to 31.02) | 1.66 (0.72 to 3.22) | -88.0 (-95.0 to -68.0) |
| **Female** | |  |  |  |  |  |  |
| <28 days | | 172.67 (104.80 to 289.61) | 5.55 (2.54 to 10.19) | -97.0 (-99.0 to -93.0) | 3,156.17 (1,968.39 to 5,095.12) | 104.23 (45.79 to 202.41) | -97.0 (-99.0 to -93.0) |
| 1-5 months | | 5.82 (3.33 to 10.82) | 0.63 (0.48 to 0.83) | -89.0 (-94.0 to -81.0) | 72.41 (29.12 to 165.40) | 2.27 (0.83 to 5.45) | -97.0 (-99.0 to -90.0) |
| 6-11 months | | 3.82 (2.54 to 6.37) | 0.56 (0.43 to 0.71) | -85.0 (-91.0 to -79.0) | 36.64 (14.79 to 76.09) | 1.11 (0.42 to 2.46) | -97.0 (-99.0 to -91.0) |
| 12-23 months | | 2.02 (1.43 to 2.61) | 0.51 (0.37 to 0.66) | -75.0 (-79.0 to -71.0) | 4.26 (1.34 to 11.17) | 0.36 (0.09 to 0.88) | -92.0 (-98.0 to -71.0) |
| 2-4 years | | 2.07 (1.46 to 2.63) | 0.50 (0.36 to 0.64) | -76.0 (-80.0 to -73.0) | 5.34 (1.67 to 11.53) | 0.36 (0.10 to 0.90) | -93.0 (-98.0 to -76.0) |
| 5-9 years | | 2.05 (1.47 to 2.67) | 0.48 (0.34 to 0.61) | -77.0 (-80.0 to -75.0) | 4.95 (1.55 to 9.92) | 0.36 (0.09 to 1.02) | -93.0 (-98.0 to -80.0) |
| 10-14 years | | 1.90 (1.35 to 2.48) | 0.47 (0.34 to 0.60) | -75.0 (-78.0 to -73.0) | 2.75 (1.14 to 6.56) | 0.28 (0.08 to 0.71) | -90.0 (-97.0 to -71.0) |
| 15-19 years | | 1.91 (1.34 to 2.50) | 0.47 (0.33 to 0.61) | -75.0 (-79.0 to -73.0) | 3.52 (1.24 to 9.48) | 0.42 (0.11 to 1.19) | -88.0 (-97.0 to -64.0) |
| 20-24 years | | 1.83 (1.31 to 2.39) | 0.46 (0.33 to 0.59) | -75.0 (-77.0 to -73.0) | 2.60 (1.02 to 5.87) | 0.30 (0.09 to 0.83) | -88.0 (-96.0 to -68.0) |
| 25-29 years | | 1.72 (1.24 to 2.22) | 0.44 (0.32 to 0.56) | -75.0 (-77.0 to -73.0) | 2.14 (0.82 to 4.68) | 0.22 (0.07 to 0.55) | -90.0 (-97.0 to -72.0) |
| 30-34 years | | 1.66 (1.20 to 2.19) | 0.40 (0.29 to 0.52) | -76.0 (-78.0 to -74.0) | 2.47 (1.06 to 5.53) | 0.25 (0.09 to 0.64) | -90.0 (-96.0 to -72.0) |
| 35-39 years | | 1.62 (1.16 to 2.08) | 0.37 (0.27 to 0.49) | -77.0 (-79.0 to -75.0) | 1.97 (0.91 to 4.00) | 0.16 (0.06 to 0.40) | -92.0 (-97.0 to -78.0) |
| 40-44 years | | 1.60 (1.12 to 2.09) | 0.37 (0.27 to 0.48) | -77.0 (-80.0 to -75.0) | 2.64 (1.02 to 6.29) | 0.24 (0.08 to 0.60) | -91.0 (-96.0 to -75.0) |
| 45-49 years | | 1.63 (1.14 to 2.13) | 0.35 (0.25 to 0.46) | -78.0 (-81.0 to -76.0) | 3.20 (1.21 to 8.15) | 0.25 (0.08 to 0.60) | -92.0 (-97.0 to -78.0) |
| 50-54 years | | 1.57 (1.12 to 2.03) | 0.33 (0.23 to 0.43) | -79.0 (-82.0 to -76.0) | 4.02 (1.56 to 9.48) | 0.32 (0.10 to 0.78) | -92.0 (-97.0 to -77.0) |
| 55-59 years | | 1.53 (1.08 to 2.03) | 0.31 (0.22 to 0.41) | -80.0 (-83.0 to -77.0) | 4.13 (1.73 to 9.27) | 0.32 (0.11 to 0.73) | -92.0 (-97.0 to -79.0) |
| 60-64 years | | 1.45 (1.02 to 1.89) | 0.29 (0.21 to 0.39) | -80.0 (-83.0 to -77.0) | 5.02 (2.10 to 10.61) | 0.42 (0.15 to 0.95) | -92.0 (-96.0 to -77.0) |
| 65-69 years | | 1.42 (0.98 to 1.87) | 0.27 (0.20 to 0.37) | -81.0 (-85.0 to -77.0) | 6.04 (2.40 to 13.22) | 0.54 (0.21 to 1.27) | -91.0 (-96.0 to -76.0) |
| 70-74 years | | 1.47 (1.02 to 2.09) | 0.25 (0.18 to 0.34) | -83.0 (-87.0 to -79.0) | 7.71 (3.14 to 18.14) | 0.68 (0.26 to 1.60) | -91.0 (-97.0 to -77.0) |
| 75-79 years | | 1.23 (0.86 to 1.75) | 0.23 (0.16 to 0.31) | -81.0 (-86.0 to -76.0) | 7.20 (2.88 to 15.75) | 0.75 (0.27 to 1.74) | -90.0 (-96.0 to -73.0) |
| 80-84 years | | 1.10 (0.76 to 1.60) | 0.20 (0.14 to 0.27) | -82.0 (-87.0 to -77.0) | 6.69 (2.73 to 14.25) | 0.68 (0.25 to 1.80) | -90.0 (-96.0 to -72.0) |
| 85+ years | | 1.10 (0.73 to 1.70) | 0.18 (0.12 to 0.26) | -83.0 (-89.0 to -76.0) | 8.85 (3.76 to 18.70) | 1.06 (0.42 to 2.22) | -88.0 (-95.0 to -67.0) |

UI= uncertainty interval.

**Table S12.** Age-specific DALY and mortality rate (95% UI) per 100,000 population by sex in 1990 and 2023.

|  | **Age-specific DALY rate (95% UI)** | | | **Age-specific mortality rate (95% UI)** | | | |  |
| --- | --- | --- | --- | --- | --- | --- | --- | --- |
|  | 1990 | 2023 | Total percentage change (%) | | 1990 | 2023 | Total percentage change (%) | |
| **Male** |  |  |  | |  |  |  | |
| <28 days | 180,024.98 (115,197.24 to 273,425.86) | 5,437.79 (2,358.66 to 12,171.52) | -97.0 (-99.0 to -93.0) | | 2,000.52 (1,280.15 to 3,038.45) | 60.43 (26.21 to 135.26) | -97.0 (-99.0 to -93.0) | |
| 1-5 months | 3,778.37 (1,484.66 to 8,144.57) | 109.57 (37.36 to 250.66) | -97.0 (-99.0 to -91.0) | | 42.08 (16.54 to 90.72) | 1.22 (0.42 to 2.79) | -97.0 (-99.0 to -91.0) | |
| 6-11 months | 1,530.54 (591.07 to 3,079.05) | 47.00 (16.76 to 101.03) | -97.0 (-99.0 to -91.0) | | 17.13 (6.61 to 34.46) | 0.53 (0.19 to 1.13) | -97.0 (-99.0 to -91.0) | |
| 12-23 months | 120.88 (27.67 to 328.57) | 9.16 (1.36 to 27.28) | -92.0 (-99.0 to -71.0) | | 1.36 (0.31 to 3.71) | 0.10 (0.01 to 0.31) | -92.0 (-99.0 to -71.0) | |
| 2-4 years | 167.90 (40.14 to 478.14) | 13.32 (2.09 to 41.55) | -92.0 (-99.0 to -67.0) | | 1.93 (0.46 to 5.51) | 0.15 (0.02 to 0.48) | -92.0 (-99.0 to -67.0) | |
| 5-9 years | 129.06 (58.30 to 235.60) | 12.23 (3.92 to 27.47) | -91.0 (-97.0 to -76.0) | | 1.55 (0.70 to 2.83) | 0.15 (0.05 to 0.33) | -91.0 (-97.0 to -76.0) | |
| 10-14 years | 71.79 (33.65 to 147.80) | 10.93 (4.18 to 25.62) | -85.0 (-95.0 to -65.0) | | 0.92 (0.43 to 1.90) | 0.14 (0.05 to 0.33) | -85.0 (-95.0 to -65.0) | |
| 15-19 years | 64.92 (29.35 to 127.79) | 10.62 (3.87 to 23.73) | -84.0 (-94.0 to -62.0) | | 0.89 (0.40 to 1.76) | 0.15 (0.05 to 0.33) | -84.0 (-94.0 to -62.0) | |
| 20-24 years | 46.95 (22.79 to 90.34) | 7.23 (2.50 to 15.98) | -85.0 (-94.0 to -62.0) | | 0.69 (0.33 to 1.33) | 0.11 (0.04 to 0.24) | -85.0 (-94.0 to -62.0) | |
| 25-29 years | 46.96 (21.18 to 90.92) | 8.26 (3.14 to 18.40) | -82.0 (-94.0 to -54.0) | | 0.75 (0.34 to 1.45) | 0.13 (0.05 to 0.29) | -82.0 (-94.0 to -54.0) | |
| 30-34 years | 52.12 (22.32 to 109.21) | 7.71 (3.10 to 19.24) | -85.0 (-94.0 to -62.0) | | 0.90 (0.39 to 1.89) | 0.13 (0.05 to 0.33) | -85.0 (-95.0 to -62.0) | |
| 35-39 years | 54.15 (25.94 to 111.22) | 7.48 (2.91 to 15.58) | -86.0 (-94.0 to -67.0) | | 1.02 (0.49 to 2.10) | 0.14 (0.05 to 0.29) | -86.0 (-94.0 to -67.0) | |
| 40-44 years | 65.70 (28.72 to 131.61) | 9.28 (3.42 to 19.75) | -86.0 (-94.0 to -64.0) | | 1.37 (0.60 to 2.75) | 0.19 (0.07 to 0.41) | -86.0 (-94.0 to -64.0) | |
| 45-49 years | 92.83 (36.52 to 191.34) | 12.58 (5.04 to 25.36) | -86.0 (-95.0 to -67.0) | | 2.16 (0.85 to 4.45) | 0.29 (0.12 to 0.59) | -86.0 (-95.0 to -67.0) | |
| 50-54 years | 91.64 (37.83 to 185.31) | 10.85 (4.48 to 22.49) | -88.0 (-95.0 to -70.0) | | 2.40 (0.99 to 4.84) | 0.28 (0.12 to 0.59) | -88.0 (-95.0 to -70.0) | |
| 55-59 years | 98.51 (40.05 to 200.11) | 11.34 (4.41 to 23.58) | -88.0 (-95.0 to -69.0) | | 2.94 (1.19 to 5.97) | 0.34 (0.13 to 0.70) | -89.0 (-95.0 to -69.0) | |
| 60-64 years | 84.89 (38.58 to 164.38) | 9.11 (3.57 to 18.93) | -89.0 (-96.0 to -72.0) | | 2.93 (1.33 to 5.68) | 0.31 (0.12 to 0.65) | -89.0 (-96.0 to -72.0) | |
| 65-69 years | 85.66 (36.95 to 204.19) | 8.96 (3.98 to 19.40) | -90.0 (-96.0 to -74.0) | | 3.51 (1.51 to 8.37) | 0.37 (0.16 to 0.79) | -90.0 (-96.0 to -74.0) | |
| 70-74 years | 89.16 (37.66 to 190.70) | 8.54 (3.43 to 17.60) | -90.0 (-96.0 to -75.0) | | 4.44 (1.87 to 9.49) | 0.42 (0.17 to 0.88) | -90.0 (-96.0 to -75.0) | |
| 75-79 years | 73.21 (30.32 to 175.62) | 8.21 (3.34 to 16.90) | -89.0 (-96.0 to -70.0) | | 4.55 (1.89 to 10.92) | 0.51 (0.21 to 1.05) | -89.0 (-96.0 to -70.0) | |
| 80-84 years | 54.98 (25.55 to 107.63) | 5.96 (2.38 to 11.78) | -89.0 (-96.0 to -75.0) | | 4.37 (2.03 to 8.53) | 0.47 (0.19 to 0.94) | -89.0 (-96.0 to -75.0) | |
| 85+ years | 48.48 (22.21 to 90.24) | 5.35 (2.38 to 10.06) | -89.0 (-95.0 to -74.0) | | 5.04 (2.32 to 9.42) | 0.56 (0.25 to 1.06) | -89.0 (-95.0 to -74.0) | |
| **Female** |  |  |  | |  |  |  | |
| <28 days | 153,793.19 (105,732.25 to 240,215.28) | 5,034.54 (2,331.05 to 9,566.43) | -97.0 (-99.0 to -93.0) | | 1,709.06 (1,174.90 to 2,669.40) | 55.95 (25.90 to 106.31) | -97.0 (-99.0 to -93.0) | |
| 1-5 months | 3,313.69 (1,282.81 to 7,404.51) | 102.06 (36.59 to 222.49) | -97.0 (-99.0 to -89.0) | | 36.91 (14.29 to 82.47) | 1.14 (0.41 to 2.48) | -97.0 (-99.0 to -89.0) | |
| 6-11 months | 1,638.70 (626.16 to 3,384.73) | 49.95 (19.83 to 99.38) | -97.0 (-99.0 to -91.0) | | 18.34 (7.01 to 37.89) | 0.56 (0.22 to 1.11) | -97.0 (-99.0 to -91.0) | |
| 12-23 months | 173.32 (56.90 to 387.71) | 15.96 (4.25 to 37.35) | -91.0 (-98.0 to -71.0) | | 1.96 (0.64 to 4.37) | 0.18 (0.05 to 0.42) | -91.0 (-98.0 to -71.0) | |
| 2-4 years | 177.50 (65.37 to 390.02) | 12.80 (3.46 to 30.09) | -93.0 (-98.0 to -78.0) | | 2.04 (0.75 to 4.49) | 0.15 (0.04 to 0.35) | -93.0 (-98.0 to -78.0) | |
| 5-9 years | 133.34 (46.25 to 252.05) | 10.45 (2.84 to 23.72) | -92.0 (-97.0 to -82.0) | | 1.60 (0.56 to 3.03) | 0.13 (0.03 to 0.29) | -92.0 (-97.0 to -82.0) | |
| 10-14 years | 68.40 (31.70 to 139.69) | 7.48 (2.23 to 17.11) | -89.0 (-96.0 to -73.0) | | 0.88 (0.41 to 1.79) | 0.10 (0.03 to 0.22) | -89.0 (-96.0 to -73.0) | |
| 15-19 years | 86.93 (33.60 to 203.06) | 11.01 (3.08 to 28.12) | -87.0 (-97.0 to -64.0) | | 1.20 (0.46 to 2.80) | 0.15 (0.04 to 0.39) | -87.0 (-97.0 to -64.0) | |
| 20-24 years | 68.00 (28.76 to 141.53) | 7.91 (2.55 to 18.77) | -88.0 (-96.0 to -71.0) | | 1.00 (0.42 to 2.09) | 0.12 (0.04 to 0.28) | -88.0 (-96.0 to -71.0) | |
| 25-29 years | 48.32 (22.04 to 96.02) | 5.00 (1.78 to 10.49) | -90.0 (-96.0 to -74.0) | | 0.77 (0.35 to 1.53) | 0.08 (0.03 to 0.17) | -90.0 (-96.0 to -74.0) | |
| 30-34 years | 52.39 (23.94 to 101.73) | 5.47 (2.12 to 11.64) | -90.0 (-96.0 to -74.0) | | 0.90 (0.41 to 1.76) | 0.09 (0.04 to 0.20) | -90.0 (-96.0 to -74.0) | |
| 35-39 years | 39.59 (20.59 to 73.16) | 3.49 (1.35 to 7.46) | -91.0 (-96.0 to -78.0) | | 0.75 (0.39 to 1.38) | 0.07 (0.02 to 0.14) | -91.0 (-96.0 to -78.0) | |
| 40-44 years | 49.07 (23.46 to 96.43) | 4.49 (1.71 to 9.75) | -91.0 (-96.0 to -76.0) | | 1.02 (0.49 to 2.01) | 0.09 (0.04 to 0.20) | -91.0 (-96.0 to -76.0) | |
| 45-49 years | 55.50 (23.83 to 108.45) | 4.21 (1.54 to 9.30) | -92.0 (-97.0 to -80.0) | | 1.29 (0.55 to 2.52) | 0.10 (0.04 to 0.22) | -92.0 (-97.0 to -80.0) | |
| 50-54 years | 63.27 (23.97 to 123.16) | 4.82 (1.75 to 10.62) | -92.0 (-97.0 to -81.0) | | 1.65 (0.62 to 3.22) | 0.13 (0.04 to 0.28) | -92.0 (-97.0 to -81.0) | |
| 55-59 years | 56.53 (27.78 to 108.08) | 4.07 (1.62 to 8.62) | -93.0 (-97.0 to -81.0) | | 1.68 (0.83 to 3.22) | 0.12 (0.05 to 0.26) | -93.0 (-97.0 to -81.0) | |
| 60-64 years | 59.82 (27.87 to 107.54) | 4.39 (1.75 to 8.94) | -93.0 (-97.0 to -82.0) | | 2.07 (0.96 to 3.72) | 0.15 (0.06 to 0.31) | -93.0 (-97.0 to -82.0) | |
| 65-69 years | 61.66 (28.68 to 112.85) | 5.09 (2.07 to 11.06) | -92.0 (-97.0 to -79.0) | | 2.53 (1.18 to 4.63) | 0.21 (0.08 to 0.45) | -92.0 (-97.0 to -79.0) | |
| 70-74 years | 69.43 (30.43 to 146.00) | 5.60 (2.32 to 12.22) | -92.0 (-97.0 to -79.0) | | 3.46 (1.52 to 7.28) | 0.28 (0.12 to 0.61) | -92.0 (-97.0 to -79.0) | |
| 75-79 years | 48.82 (23.86 to 93.73) | 4.75 (1.91 to 9.94) | -90.0 (-96.0 to -75.0) | | 3.05 (1.49 to 5.85) | 0.30 (0.12 to 0.62) | -90.0 (-96.0 to -75.0) | |
| 80-84 years | 33.74 (14.75 to 61.54) | 3.27 (1.30 to 6.74) | -90.0 (-96.0 to -76.0) | | 2.69 (1.17 to 4.90) | 0.26 (0.10 to 0.54) | -90.0 (-96.0 to -76.0) | |
| 85+ years | 31.46 (13.57 to 55.92) | 3.20 (1.26 to 6.28) | -90.0 (-96.0 to -77.0) | | 3.29 (1.44 to 5.84) | 0.34 (0.14 to 0.67) | -90.0 (-96.0 to -76.0) | |

DALY= disability-adjusted life year; UI= uncertainty interval.
