# Supplementary material for: Global, regional, and national burden of tetanus in 204 countries and territories, 1990–2023: a systematic analysis for the Global Burden of Disease Study 2023
Source: eClinicalMedicine. 2026 Sep 28;100:104202. doi: 10.1016/j.eclinm.2026.104202 (PMC13639606; doi:10.1016/j.eclinm.2026.104202)
Supplement: Supplementary file 2 [file mmc2.docx]

**Appendix 2: Authorship appendix to “Global, regional, and national burden of tetanus in 204 countries and territories, 1990-2023: a systematic analysis for the Global Burden of Disease Study 2023”**

This appendix provides further authorship detail for “Global, regional, and national burden of tetanus in 204 countries and territories, 1990-2023: a systematic analysis for the Global Burden of Disease Study 2023.”

**Table of Contents**

**GBD 2023 Tetanus Collaborators	2**

**Affiliations	4**

**Authors’ Contributions	11**

Providing data or critical feedback on data sources	11

Developing methods or computational machinery	11

Providing critical feedback on methods or results	11

Drafting the work or revising it critically for important intellectual content	13

Managing the estimation or publications process	14

**GBD 2023 Tetanus Collaborators**

Demelash Areda*, Hyunjee Kim*, Jiseung Kang*, Noga Shalev, Ishu Poudyal, Min Seo Kim, Benn Sartorius, Hasan Aalruz, Nadin M I Abdel Razeq, Sherief Abd-Elsalam, Meriem Abdoun, Jeza Muhamad Abdul Aziz, Abdulrakib Abdulrahim, Mesfin Abebe, Olifan Zewdie Abil, Richard Gyan Aboagye, Khaled M Aboshanab, Amr Selim Abu Lila, Nermeen Abu-Elala, Aishah Fadila Adamu, Tajudeen Adesanmi Adebisi, Ayo Stephen Adebowale, Daniel Adeyemi Adepoju, Belete Muluadam Admassie, Qorinah Estiningtyas Sakilah Adnani, Aanuoluwapo Adeyimika Afolabi, Muhammad Sohail Afzal, Saira Afzal, Gizachew Beykaso Agafari, Percival Delali Agordoh, Salome Wumpini Agordoh, Abdul-Raheem Folorunsho Ahmad, Khabir Ahmad, Muayyad M Ahmad, Ali Ahmed, Ayman Ahmed, Gasha Salih Ahmed, Meqdad Saleh Ahmed, Mohamed Mustaf Mustaf Ahmed, Oluwasefunmi Akeju, Akil Akhtar, Muhammad Nadeem Akhtar, Olumide Odunayo Akinniyi, Karolina Akinosoglou, Damilare Michael Akintunde, Salah Al Awaidy, Muath A Alammar, Mohammed Albashtawy, Ali M Alfalki, Abdelazeem M Algammal, Mohammad Daud Ali, Mohammed Usman Ali, Mohammed Alissa, Alaa A. A. Aljabali, Sameer Abdulmalik Alkubati, Mohammed Z. Allouh, Joseph Uy Almazan, Mohammed Almunef, Khaldoon Aied Alnawafleh, Mushtak Talib Salih Al-Ouqaili, Mohammad R Alqudimat, Ahmad Rajeh Al-Qudimat, Najim Z Alshahrani, Ali Salman Al-Shami, Abdalkarem Fedgash Alsharari, Zaid Altaany, Awais Altaf, Kassaye D. Demeke Altaye, Nelson Alvis-Guzman, Hany Aly, Raed M Al-Zoubi, Nada A. Alzunaidy, Abhishek Anil, Mohammad Azam Ansari, Boluwatife Stephen Anuoluwa, Razique Anwer, Mosab Arafat, Muhammad Shahzad Aslam, Maha Moh'd Wahbi Atout, Alok Atreya, Matteo Augello, Usman Ayub Awan, Kemal Lemnuro Awol, Habtamu Molla Ayele, AKM Azad, Ahmed Y Azzam, Syed Babar Jamal Bacha, Riadh Badraoui, Nayereh Baghcheghi, Khlood K Baghlaf, Senthilkumar Balakrishnan, Aleksandra Barac, Dennis Bardoe, Hiba Jawdat Barqawi, Shazia Batool, Ravi Batra, Nebiyou Simegnew Bayleyegn, Narasimha M Beeraka, Desalegn Weldebrhan Bekru, Asnake Gashaw Belayneh, Samiun Nazrin Bente Kamal Tune, Pankaj Bhardwaj, Sonu Bhaskar, Jasvinder Singh Bhatti, Md Mehedi Hasan Bhuiyan, Can Bilgin, Trupti Bodhare, Eyob Ketema Bogale, Souad Bouaoud, Christopher Boxe, Sayed Muhammad Ata Ullah Shah Bukhari, Yasser Bustanji, Zahid A Butt, Bindiya C C Gowda, Sandip Chakraborty, Imane Chamkhi, Rama Mohan Chandika, Vijay Kumar Chattu, Lam Duc Chau, Akhilanand Chaurasia, Haiyan Chen, Patrick R Ching, Arrigo Francesco Giuseppe Cicero, Bashir Dabo, Omid Dadras, Xiaochen Dai, Fernando Pio De la Hoz, Bryan Gervais de Liyis, Sindhura Deekonda, Ivan Delgado-Enciso, Vinoth Gnana Chellaiyan Devanbu, Devananda Devegowda, Syed Masudur Rahman Dewan, Giuseppe Di Martino, Marcello Di Pumpo, Ojas Prakashbhai Doshi, Robert Kokou Dowou, Hasan Durmuş, Veli DurmuŞ, Oyewole Christopher Durojaiye, Siddhartha Dutta, Venkata Ravikanth Eddula, Martin Herbas Ekat, Rabie Adel El Arab, Muhammed Elhadi, Yasir Ahmed Mohammed Elhadi, Yasser S. El-Sayed, Chadi Eltaha, Abdelgawad Salah Eltahawy, Simachew Getaneh Endalamew, Collins Nduka Esomchi, Peter Maduabuchi Eze, Adeniyi Francis Fagbamigbe, Ayesha Fahim, Ebenezer Gezahegn Fanta, Aisha Farhana, Wafa Fatima, Olufunmilayo Ibitola Fawole, Getahun Fetensa, Tommaso Filippini, Claudio Fiorilla, Florian Fischer, Aemon Berhane Fissha, Federica Fogacci, Takeshi Fukumoto, Dominic Dormenyo Gadeka, Márió Gajdács, Ezhilkugan Ganessane, Jacopo Garlasco, Rupesh K Gautam, Mebin George Mathew, Ali Gerami Matin, Shumiye Shiferaw Gessess, Ramy Mohamed Ghazy, Sama Ghoba, Syed Abdullah Gilani, Najam Gohar, Mahaveer Golechha, Yitayal Ayalew Goshu, Bashar Haruna Gulumbe, Sapna Gupta, Awoke Derbie Habteyohannes, Hideharu Hagiya, Teklehaimanot Gereziher Haile, Pritam Halder, Raz Mohammed Hama Salih, Mohamed H. Hamad, Fatimah Othman Hamarasool, Samer Hamidi, Abdelrahman M Hamouda, Didem Han Yekdeş, Arief Hargono, Sara Harsini, Abdul Haseeb, Md Saquib Hasnain, Ibrahim Nagmeldin Hassan, Simon I Hay, Zaw Myo Hein, Abdelaziz Hendy, Md Sabbir Hossain, MD Talib Hussain, Segun Emmanuel Ibitoye, Sumaira Idrees, Ann Oluchi Ikeabbah, Adalia Ikiroma, Olayinka Stephen Ilesanmi, Irena M Ilic, Milena D Ilic, Korakrit Imwattana, Md. Mojahedul Islam, Aloysia Ispriantari, Mihajlo Jakovljevic, Arshad Jamal, Jazlan Jamaluddin, Jerin James, Yovanthi Anurangi Jayasinghe, Bijay Mukesh Jeswani, Nitin Joseph, Charity Ehimwenma Joshua, Fozi Musa Jundi, Dhinesh Babu K, Divya K L, Saltanat Kamenova, Oleksandr Kamyshnyi, Kehinde Kazeem Kanmodi, Suthanthira Kannan S, Reddy Praneeth Karnam, Tomasz M Karpiński, Awoke Amare Kebede, Himanshu Khajuria, Sidra Khalid, Hayat Ahmad Khan, Muhammad Arslan Khan, Raees Khan, Ramsha Mushtaq Khan, Yusuf Saleem Khan, Khaled Khatab, Feriha Fatima Khidri, Daniel Gebrehawaria Kinfe, Shivakumar KM Kondlahalli, Aida Kondybayeva, Hamid Reza Koohestani, Vladimir Andreevich Korshunov, Kewal Krishan, Mukhtar Kulimbet, Shweta Kulshreshtha, Dewesh Kumar, Pradeep Kumar, Rakesh Kumar, Abigail Kusi Amponsah, Dian Kusuma, Ville Kytö, Chandrakant Lahariya, Yemane Leake, Caterina Ledda, Hoi Leong Lee, Hwamin Lee, Seung Won Lee, Ambreen Liaqat, Anqi Lin, Madeeha Shahzad Lodhi, José Francisco López-Gil, Peng Luo, Ellina Lytvyak, Franciele Maboni Siqueira, Mahmoud Mabrok, Monika Machoy-Rakoczy, Máté Mackei, Edward Augustine Magwe, Sasikumar Mahalingam, Nozad Hussein Mahmood, Tabarak Malik, Aseer Manilal, Kaleem Maqsood, Bernardo Alfonso Martinez-Guerra, Medha Mathur, Fernanda Penido Matozinhos, Prosanjit Mazumder, Uwakwe C Mba, Agezegn Asegid Mekonnen, Hadush Negash Meles, Tomislav Mestrovic, Muhammad Agus Mikrajab, Bhagyashree Mishra, Nouh Saad Mohamed, Khabab Abbasher Hussien Mohamed Ahmed, Yahaya Mohammed, Ali H Mokdad, Lorenzo Monasta, Rohith Motappa, Sumaira Mubarik, Christopher J L Murray, Ghulam Mustafa, Hastyar Hama Rashid Najmuldeen, Zuhair S Natto, Biswa Prakash Nayak, Nini Asfaw Negash, Charles Richard James Newton, Cuong Tat Nguyen, Trang Nguyen, Vikram Niranjan, Ali Aahil Noorali, Emira Noumi, Chisom Adaobi Nri-Ezedi, Fred Nugen, Alex Odoom, Felix Akpojene Ogbo, Foluso Olamide Olamide Ojo, Osaretin Christabel Okonji, Michael Chinyem Okonkwo, Lenz Nwachinemere Okoro, Morufat Oluwatosin Olaitan, Atef Oreiby, Thompson Osei, Olutosin Ademola Otekunrin, Amel Ouyahia, Mayowa O Owolabi, Kehinde Adewole Oyeniran, Ilker Ozsahin, Jagadish Rao Padubidri, Chulwoo Park, Sameena Parveen, Shankargouda Patil, Mohamed Anas Patni, Iuliia Pavlova, Zahra Zahid Piracha, Amit Porwal, Xiang Qi, Igor Radenkov, Fakher Rahim, Bilal Ahmad Rahimi, Md Sazedur Rahman, Mosiur Rahman, Jeffrey Pradeep Raj, Gunaseelan Rajendran, Mahmoud Mohammed Ramadan, Kadar Ramadhan, Lakshmi Ramamoorthy, Nemanja Rancic, Mithun Rao, Elrashdy M Redwan, Abdul Rehman, Wajiha Rehman, Yohanes Andy Rias, Luca Ronfani, Parimal Roy, Aly M A Saad, Umar Saeed, Ashok Kumar Sah, Indranil Saha, Fatemeh Saheb Sharif-Askari, Narjes Saheb Sharif-Askari, Amirhossein Sahebkar, Joseph W Sakshaug, Samreen Saleem, Mohamed A Saleh, Malik Adebayo Sanusi, Aswini Saravanan, Brijesh Sathian, Mukesh Kumar Sathya Narayanan, Subramanian Senthilkumaran, Yashendra Sethi, Muhammad Shabbir, Disha Sanjivkumar Shah, Liloma Shah, Syed Ali Raza Shah, Shazlin Shaharudin, Sunder Sham, Mohammed Shannawaz, Amit Baran Sharangi, Nadim Sharif, Sumaira Sharif, Avimanu Sharma, Bhoopesh Kumar Sharma, Kamlesh Sharma, Ravi Kumar Sharma, Bereket Beyene Shashamo, Mahabalesh Shetty, Pavanchand Shetty, Premalatha K Shetty, Aminu Shittu, Km Shivangi, Seyed Afshin Shorofi, Suleiman Adeiza Shuaibu, Harpreet Singh, Jasvinder A Singh, Abhijeet Prasad Sinha, Mejdi Snoussi, Yavuzalp solak, Ahmed M Soliman, Yizhe Song, Aayushi Sood, Anna Maria Spagnolo, Marjia Sultana, Joshua Sumankuuro, Tarun Kumar Suvvari, Ramin Tabibi, Zanan Mohammed-Ameen Taha, Jianye Tan, Rekha Thiruvengadam, Christina S Thornton, Marcos Roberto Tovani-Palone, Nguyen Tran Minh Duc, Amritha Trikkur Madom Seetharaman, Munkhtuya Tumurkhuu, Md Jamal Uddin, Irfan Ullah, Lawan Umar, Deepadarshan Urs, Hande Uzunçıbuk, Giacomo Pietro Vigezzi, Vignesh Vikram, Tuan Vinh, Yasir Waheed, Xingxin Wang, Tanveer Wani, Nuwan Darshana Wickramasinghe, Mieszko Wieckiewicz, Camillus Abawiera Wongnaa, Yihun Miskir Miskir Wubie, Galal Yahya, Hanwen Yang, Vahit Yiğit, Naohiro Yonemoto, Fathiah Zakham, Giulia Zamagni, Aurora Zanghì, Seema Zargar, Mohammed G M Zeariya, Basit Zeshan, Mohamed Ali Zoromba, Abdulhakim Mussema Zuber, Ashley A Harris, Jonathan F Mosser^, and Dong Keon Yon^.

**Joint first authors*

*^Corresponding authors*

**Affiliations**

College of Art and Science (D Areda PhD), Ottawa University, Surprise, AZ, USA; Center for Digital Health, Medical Science Research Institute (H Kim), Kyung Hee University College of Medicine, Seoul, South Korea; School of Health and Environmental Science (Prof. J Kang PhD), College of Medicine (M Elhadi MD), Department of Biomedical Informatics (Prof. H Lee PhD), Korea University, Seoul, South Korea; Department of Anesthesia, Critical Care and Pain Medicine (Prof. J Kang PhD), Massachusetts General Hospital, Boston, MA, USA (M Kim MD); Institute for Health Metrics and Evaluation (N Shalev MD, I Poudyal BA, X Dai PhD, S Ghoba MS, Prof. S I Hay DSc, T Mestrovic PhD, Prof. A H Mokdad PhD, Prof. C J L Murray DPhil, A A Harris MPH, J F Mosser MD), Department of Health Metrics Sciences, School of Medicine (B Sartorius PhD, X Dai PhD, Prof. S I Hay DSc, Prof. A H Mokdad PhD, Prof. C J L Murray DPhil), University of Washington, Seattle, WA, USA; Cardiovascular Disease Initiative (M Kim MD), Broad Institute of MIT and Harvard, Cambridge, MA, USA; Faculty of Medicine (B Sartorius PhD), Faculty of Health, Medicine and Behavioural Sciences (M Rahman PhD), The University of Queensland, Brisbane, QLD, Australia; Nuffield Department of Medicine (B Sartorius PhD), Oxford Vaccine Group (O Akeju MPH), Department of Psychiatry (Prof. C R J Newton MD), Radcliffe Department of Medicine (T Vinh BS), University of Oxford, Oxford, England; Department of Nursing (H Aalruz PhD), Al Zaytoonah University of Jordan, Amman, Jordan; School of Nursing (Prof. N M I Abdel Razeq PhD), The University of Jordan, Amman, Jordan; Department of Tropical Medicine and Infectious Diseases (S Abd-Elsalam PhD), Tanta University, Tanta, Egypt; Department of Medicine (Prof. M Abdoun PhD), Department of Health (Prof. M Abdoun PhD), University of Setif Algeria, Sétif, Algeria; Department of Medical Laboratory Sciences (A Manilal PhD), Komar University of Science and Technology, Sulaymaniyah, Iraq (J M Abdul Aziz MSc); Baxshin Hospital (J M Abdul Aziz MSc), Baxshin Research Center, Sulaymaniyah, Iraq; Department of Medical Microbiology (A Abdulrahim BSc), Universiti Putra Malaysia, Serdang, Malaysia; Department of Midwifery (M Abebe MSc), Dilla University, Dilla, Ethiopia; Department of Immunology (O Z Abil PhD), Tulane University, Covington, LA, USA; College of Medicine and Health Sciences (O Z Abil PhD), Ambo University, Ambo, Ethiopia; Department of Family and Community Health (R G Aboagye MPH), School of Medicine (A F Adamu MD), Department of Nutrition and Dietetics (P D Agordoh MPhil), Department of Epidemiology and Biostatistics (R K Dowou MPhil), University of Health and Allied Sciences, Ho, Ghana; School of Population Health (R G Aboagye MPH), University of New South Wales, Sydney, NSW, Australia; Faculty of Pharmacy (Prof. K M Aboshanab PhD), Faculty of Nursing (A Hendy PhD), Ain Shams University, Cairo, Egypt; Department of Pharmaceutics (Prof. A S Abu Lila PhD), Department of Medical Surgical Nursing (S A Alkubati PhD), Department of Biology (Prof. R Badraoui PhD), Pharmacology and Toxicology (M Hussain PhD), Biology Department (A Jamal PhD, Prof. E Noumi PhD, Prof. M Snoussi PhD), College of Medicine (Y S Khan MD), Department of Health Management (R Kumar PhD), Department of Public Health (M G M Zeariya PhD), University of Hail, Hail, Saudi Arabia; Faculty of Veterinary Medicine (Prof. N Abu-Elala PhD), Cairo University, Cairo, Egypt; Faculty of Veterinary Medicine (Prof. N Abu-Elala PhD), King Salman International University, Rus Sudr, Egypt; Department of Microbiology (T A Adebisi BSc), Ladoke Akintola University, Osogbo, Nigeria; NMC Healthcare (T A Adebisi BSc), Independent Consultant, Sharjah, United Arab Emirates; Faculty of Public Health (Prof. A S Adebowale PhD), Department of Veterinary Medicine (O O Akinniyi MSc), Department of Epidemiology and Medical Statistics (A F Fagbamigbe PhD, Prof. O I Fawole PhD), Department of Health Promotion and Education (S Ibitoye PhD), Department of Medicine (Prof. M O Owolabi DrM), University of Ibadan, Ibadan, Nigeria; Department of Health Informatics (D A Adepoju MD), Indiana University Indianapolis, Indianapolis, IN, USA; Department of Anesthesia (B M Admassie MSc), Department of Emergency and Critical Care Nursing (A G Belayneh MSc), Department of Health Promotion and Behavioural Science (E K Bogale MPH), Department of Veterinary Epidemiology and Public health (S Endalamew MPH), department of midwifery (S S Gessess MSc), Department of Medical Microbiology (A D Habteyohannes PhD), Department of Public Health Nutrition and Nursing Related (A A Kebede MSc), Department of Emergency Medicine and Critical Care Nursing (Y M Wubie MPH), Bahir Dar University, Bahir Dar, Ethiopia; Department of Public Health (Q Adnani PhD), Universitas Padjadjaran (Padjadjaran University), Bandung, Indonesia; Technical Services Directorate (A A Afolabi MPH), MSI Nigeria Reproductive Choices, Abuja, Nigeria; Department of Life Sciences (M S Afzal PhD), University of Management and Technology, Lahore, Pakistan; Department of Community Medicine (Prof. S Afzal PhD), King Edward Memorial Hospital, Lahore, Pakistan; Department of Public Health (Prof. S Afzal PhD), Public Health Institute, Lahore, Pakistan; Department of Public Health (G B Agafari PhD), Wachemo University, Hossana, Ethiopia; Operations Department (S W Agordoh MPH), World Vision, Kete Krachi, Ghana; Department of Biochemistry (A F Ahmad MSc), University of Lagos, Ilesa, Nigeria; Public Health and Epidemiology (A F Ahmad MSc), Ministry of Health, Osogbo, Nigeria; King Khaled Eye Specialist Hospital and Research Center, Riyadh, Saudi Arabia (Prof. K Ahmad PhD); School of Nursing (Prof. M M Ahmad PhD), University of Jordan, Amman, Jordan; Department of Pharmacy Practice (A Ahmed PhD), Riphah Institute of Pharmaceutical Sciences, Islamabad, Pakistan; Division of Infectious Diseases and Global Public Health (IDGPH) (A Ahmed PhD), University of California San Diego, San Diego, CA, USA; Institute of Endemic Diseases (A Ahmed PhD), Department of Medicine (I Hassan MD), Faculty of Medicine (K Mohamed Ahmed MD), University of Khartoum, Khartoum, Sudan; Pan-African One Health Institute (PAOHI), Kigali, Rwanda (A Ahmed PhD); Medical Laboratory Science Department (G S Ahmed MSc), University of Human Development, Sulaymaniyah, Iraq; Department of Pathology and Microbiology (M S Ahmed PhD), Duhok Research Centre (Z M Taha PhD), University of Duhok, Duhok, Iraq; Institute for Global Health (M M Ahmed MD), SIMAD University, Mogadishu, Somalia; Emory University School of Medicine (A Akhtar PhD), Emory University, Atlanta, GA, USA; University Institute of Diet and Nutritional Sciences (M Akhtar PhD), Institute of Molecular Biology and Biotechnology (IMBB) (A Altaf PhD, M S Lodhi PhD, S Shah PhD, S Sharif PhD), University Institute of Public Health (W Fatima PhD), The University of Lahore, Lahore, Pakistan; Department of Internal Medicine (K Akinosoglou PhD), University of Patras, Patras, Greece; Department of Internal Medicine and Infectious Diseases (K Akinosoglou PhD), University General Hospital of Patras, Patras, Greece; Medecins Sans Frontieres - Holland (D M Akintunde MPH), Doctors Without Borders, Omdurman, Sudan; Department of Community Medicine (D M Akintunde MPH), Ahmadu Bello University, Zaria, Nigeria; Department of Communicable Diseases (S Al Awaidy MSc), Ministry of Health, Muscat, Oman; Middle East, Eurasia, and Africa Influenza Stakeholders Network, Muscat, Oman (S Al Awaidy MSc); Department of Family Medicine (Prof. M A Alammar PhD), College of Medicine (Prof. G Mustafa MD), College of Medicine at Shaqra (M Shabbir FCPS), Shaqra University, Shaqra, Saudi Arabia; Department of Community and Mental Health (Prof. M Albashtawy PhD), Al al-Bayt University, Mafraq, Jordan; Department of Epidemiology and Biostatistics (A M Alfalki MPH), University of South Carolina, Columbia, SC, USA; Department of Bacteriology, Immunology, and Mycology (Prof. A M Algammal PhD), Faculty of Veterinary Medicine (M Mabrok PhD), Suez Canal University, Ismailia, Egypt; Department of Pharmacy (M Ali PhD), Mohammed Al-Mana College for Medical Sciences, Dammam, Saudi Arabia (R A El Arab PhD); Department of Medical Rehabilitation (Physiotherapy) (M U Ali PhD), University of Maiduguri, Maiduguri, Nigeria; Nethersole School of Nursing (M U Ali PhD), The Chinese University of Hong Kong, Hong Kong, China; Department of Medical Laboratory (M Alissa PhD), College of Nursing (M Zoromba PhD), Prince Sattam bin Abdulaziz University, Al-Kharj, Saudi Arabia; Faculty of Pharmacy (Prof. A A A Aljabali PhD), Yarmouk University, Irbid, Jordan; Department of Anatomy (Prof. M Z Allouh PhD), Institute of Public Health (Y A M Elhadi PhD), Department of Veterinary Medicine (A Oreiby PhD), United Arab Emirates University, Al Ain, United Arab Emirates; Faculty of Medicine (Prof. M Z Allouh PhD), Jordan University of Science and Technology, Irbid, Jordan; Department of Medicine (J U Almazan PhD), Nazarbayev University, Astana, Kazakhstan; Department of Pharmacy Practice, College of Pharmacy (M Almunef PhD), Department of Food Science and Human Nutrition (N A Alzunaidy PhD), Department of Maternal and Child Health Nursing (A Hendy PhD), Qassim University, Buraydah, Saudi Arabia; Nursing Faculty (K A Alnawafleh PhD), Fahad Bin Sultan University, Tabuk, Saudi Arabia; Department of Microbiology, College of Medicine (Prof. M Al-Ouqaili PhD), University of Anbar, Ramadi, Iraq; American University of the Middle East, Egaila, Kuwait (M R Alqudimat PhD); Surgical Research Section (A R Al-Qudimat MPH), Department of Surgery (Prof. R Al-Zoubi PhD), Hamad Medical Corporation, Doha, Qatar; Department of Family and Community Medicine (N Z Alshahrani MD), University of Jeddah, Jeddah, Saudi Arabia; Department of Pharmacology (A S Al-Shami MS), Sana'a University, Sanaa, Yemen; Department of Basic Medical Sciences (A S Al-Shami MS), Universiti Malaysia Sarawak, Kota Samarahan, Malaysia; College of Nursing (Prof. A F Alsharari PhD), Department of Clinical Laboratory Sciences (A Farhana PhD), Jouf University, Sakaka, Saudi Arabia; Yarmouk University (Z Altaany PhD), Faculty of Medicine, Irbid, Jordan; Higher Colleges of Technology (Z Altaany PhD), Higher Colleges of Technology, Abu Dhabi, United Arab Emirates; Faculty of Health Sciences (A Altaf PhD), Equator University of Science and Technology, Uganda, Masaka, Uganda; University of Gondar (K D Altaye MD), College of Medicine and Health Sciences (N A Negash MSc), University of Gondar, Gondar, Ethiopia; Research Group in Health Economics (Prof. N Alvis-Guzman PhD), Universidad de Cartagena (University of Cartagena), Cartagena, Colombia; Research Group in Hospital Management and Health Policies (Prof. N Alvis-Guzman PhD), Universidad de la Costa (University of the Coast), Barranquilla, Colombia; Department of Pediatrics (Prof. H Aly MD), Cleveland Clinic, Cleveland, OH, USA; Department of Health Sciences (Prof. R Al-Zoubi PhD), Qatar University, Doha, Qatar; Department of Pharmacology (A Anil MD), All India Institute of Medical Sciences, Bhubaneswar, India; Department of Epidemic Diseases Research (Prof. M A Ansari PhD), Imam Abdulrahman Bin Faisal University, Dammam, Saudi Arabia; Department of Environmental and Occupational Health (B S Anuoluwa PhD), University of Medical Sciences, Ondo, Ondo, Nigeria; College of Medicine (R Anwer PhD), Department of Mathematics & Statistics (A Azad PhD), Imam Mohammad Ibn Saud Islamic University, Riyadh, Saudi Arabia; Al Ain University, Abu Dhabi, United Arab Emirates (Prof. M Arafat PhD); School of Traditional Chinese Medicine (M Aslam PhD), Xiamen University Malaysia, Sepang, Malaysia; Faculty of Nursing (M M W Atout PhD), Philadelphia University, Amman, Jordan; Department of Forensic Medicine (A Atreya MD), Lumbini Medical College, Palpa, Nepal; Department of Health Sciences (M Augello MD), University of Milan, Milan, Italy; Clinic of Infectious Diseases and Tropical Medicine (M Augello MD), ASST Santi Paolo e Carlo, Milan, Italy; Department of Medical Laboratory Technology (U Awan PhD), The University of Haripur, Haripur, Pakistan; Internal Medicine Department (K L Awol MD), St Paul's Hospital Millennium Medical College, Addis Ababa, Ethiopia; Bloomberg School of Public Health (K L Awol MD, E G Fanta MD), School of Medicine (B M Jeswani MBBS), Department of Medicine (A Noorali MD), Johns Hopkins University, Baltimore, MD, USA; Africa Centre for Disease Control and Prevention, Addis Ababa, Ethiopia (H Ayele MPH); Maternal and Child Health Directorate (H Ayele MPH), Federal Ministry of Health, Addis Ababa, Ethiopia; ASIDE Healthcare, Lewes, DE, USA (A Y Azzam MD); Faculty of Medicine (A Y Azzam MD), October 6 University, 6th of October City, Egypt; Department of Biological Sciences (S B J Bacha PhD, S Bukhari MS, R Khan PhD), Biological Sciences Department (L Shah Mphil), National University of Medical Sciences (NUMS), Rawalpindi, Pakistan; Faculty of Medicine of Tunis (Prof. R Badraoui PhD), University of Tunis El Manar, Tunis, Tunisia; Department of Nursing (N Baghcheghi PhD), Social Determinants of Health Research Center (H Koohestani PhD), Saveh University of Medical Sciences, Saveh, Iran; Pediatric Dentistry Department (K K Baghlaf PhD), Department of Dental Public Health (Prof. Z S Natto DrPH), King Fahd Medical Research Center (KFMRC) (Prof. Z S Natto DrPH), King Abdulaziz University, Jeddah, Saudi Arabia; Division of Biological Sciences (S Balakrishnan PhD), Tamil Nadu State Council for Science and Technology, Chennai, India; Clinic for Infectious and Tropical Disease (A Barac PhD), Clinical Center of Serbia, Belgrade, Serbia; Faculty of Medicine (A Barac PhD, I M Ilic PhD), University of Belgrade, Belgrade, Serbia; Department of Public Health Education (D Bardoe MPhil), University of Skills Training and Entrepreneurial Development, Mampong, Ghana; Clinical Sciences Department (H J Barqawi MPhil, N Saheb Sharif-Askari PhD), Department of Basic Biomedical Sciences (Prof. Y Bustanji PhD), Department of Clinical Sciences (Prof. M M Ramadan PhD), Sharjah Institute of Medical Sciences (F Saheb Sharif-Askari PhD), College of Medicine (Prof. M A Saleh PhD), University of Sharjah, Sharjah, United Arab Emirates; Institute of Chemical Sciences (S Batool MS), Bahauddin Zakariya University, Multan, Pakistan; School of Public Health (R Batra MS), University of Nevada Las Vegas, Las Vegas, NV, USA; IT Department (R Batra MS), Coforge, Georgia, GA, USA; Department of Surgery (N S Bayleyegn MD), Institute of Health (Prof. T Malik PhD), Jimma University, Jimma, Ethiopia; Department of Human Anatomy and Histology (Prof. N M Beeraka PhD), Department of Epidemiology and Evidence-Based Medicine (V A Korshunov PhD), I.M. Sechenov First Moscow State Medical University, Moscow, Russia; Faculty of Midwifery and Nursing (Department of Adult Health Nursing) (D W Bekru MSc), Mekelle University, Mekelle, Ethiopia; BRAC James P Grant School of Public Health (S Bente Kamal Tune MPH), BRAC University, Dhaka, Bangladesh; National Institute for Implementation Research on Non Communicable Diseases (Prof. P Bhardwaj MD), Indian Council of Medical Research, Jodhpur, India; Global Health Neurology Lab (S Bhaskar MD), NSW Brain Clot Bank, Sydney, NSW, Australia; Division of Cerebrovascular Medicine and Neurology (S Bhaskar MD), National Cerebral and Cardiovascular Center, Suita, Japan; Department of Human Genetics and Molecular Medicine (Prof. J S Bhatti PhD), Central University of Punjab, Bathinda, India; Department of Statistics and Data Science (M Bhuiyan MS), University of Central Florida, Orlando, FL, USA; TAF Uludag Winter Training Center (C Bilgin MD), Turkish Ministry of Defence, Bursa, Turkiye; Department of Community and Family Medicine (Prof. T Bodhare MD), All India Institute of Medical Sciences, Ramanathapuram, India; Department of Medicine (Prof. S Bouaoud DrPH), Faculty of Medicine (Prof. A Ouyahia PhD), University Ferhat Abbas of Setif, Sétif, Algeria; Department of Epidemiology and Preventive Medicine (Prof. S Bouaoud DrPH), University Hospital Saadna Abdenour, Sétif, Algeria; Department of Earth, Environment, and Equity (C Boxe PhD), Howard University, Washington, DC, USA; School of Pharmacy (Prof. Y Bustanji PhD), The University of Jordan, Amman, Jordan; School of Public Health Sciences (Z A Butt PhD), University of Waterloo, Waterloo, ON, Canada; Al Shifa School of Public Health (Z A Butt PhD), Al Shifa Trust Eye Hospital, Rawalpindi, Pakistan; Department of Community Medicine (B C C Gowda MD), Employees' State Insurance Model Hospital, Hyderabad, India; Department of Community Medicine and Family Medicine (B C C Gowda MD), Department of Pharmacology (A Saravanan MD), All India Institute of Medical Sciences, Jodhpur, India; State Disease Investigation Laboratory (S Chakraborty M.V.Sc), Animal Resources Development Department, Agartala, India; Scientific Institute (Prof. I Chamkhi PhD), University Mohammed V, Rabat, Morocco; Clinical Nutrition Department (R M Chandika PhD), Department of Public Health, Health Informatics Program (S Idrees MS), Department of Maxillofacial Surgery and Diagnostic Sciences (S Parveen PhD), Department of Prosthetic Dental Sciences (A Porwal PhD), Department of Public Health (W Rehman MS), Jazan University, Jazan, Saudi Arabia; Department of Public Health and Health Sciences (Prof. V Chattu PhD), Tennessee State University (TSU), Nashville, TN, USA; Department of Community Medicine (Prof. V Chattu PhD), Datta Meghe Institute of Medical Sciences, Sawangi, India; Department of Epidemiology (L D Chau BA), The University of Texas Health Science Center at Houston (UTHealth Houston), Houston, TX, USA; Department of Oral Medicine and Radiology (Prof. A Chaurasia MD), King George's Medical University, Lucknow, India; Clinical Project Management Office (H Chen MPH), National Clinical Research Center for Infectious Diseases, Shenzhen, Shenzhen, China; Division of Infectious Diseases (P R Ching MD), Virginia Commonwealth University, Richmond, VA, USA; Hypertension and Cardiovascular Risk Research Group (Prof. A F G Cicero PhD), Hypertension and Cardiovascular Risk Research Center (F Fogacci MDc), University of Bologna, Bologna, Italy; Heart-Chest-Vascular Department (Prof. A F G Cicero PhD), IRCCS Azienda Ospedaliero-Universitaria di Bologna, Bologna, Italy; Department of Epidemiology and Biostatistics (B Dabo MSPH), University of South Florida, Tampa, FL, USA; Department of Medical Microbiology and Parasitology (B Dabo MSPH), Bayero University, Kano, Nigeria; Center for Immigrant and Refugee Health (O Dadras PhD), Public Health Institute, Los Angeles, CA, USA; Department of Public Health (Prof. F P De la Hoz PhD), National University of Colombia, Bogota, Colombia; Department of Neurosurgery (B G de Liyis MD), National Brain Center in Indonesia, Jakarta, Indonesia; Department of Pediatrics (S Deekonda MD), Brookdale University Hospital Medical Center, Brooklyn, NY, USA; School of Medicine (I Delgado-Enciso DSc), University of Colima, Colima, Mexico; Department of Research (I Delgado-Enciso DSc), State Cancerology Institute of Colima, IMSS‑BIENESTAR, Colima, Mexico; Chettinad Hospital & Research Institute (Prof. V Devanbu MD), Chettinad Academy of Research and Education, Chennai, India; JSS Medical College (D Devegowda PhD), School of Public Health (D K L MPH), Jagadguru Sri Shivarathreeswara Academy of Health Education and Research, Mysuru, India; Department of Pharmacy (S Dewan PhD), United International University, Dhaka, Bangladesh; Pharmacology Division (S Dewan PhD), Center for Life Sciences Research Bangladesh, Dhaka, Bangladesh; Department of Medicine and Aging Sciences (Prof. G Di Martino MD), "G. d'Annunzio" University of Chieti-Pescara, Chieti, Italy; Unit of Epidemiology and Health Statistics (Prof. G Di Martino MD), Local Health Authority of Pescara, Pescara, Italy; Department of Life Science and Public Health (M Di Pumpo DrPH), Università Cattolica del Sacro Cuore (Catholic University of the Sacred Heart), Rome, Italy; Independent Consultant, Bridgewater, NJ, USA (O P Doshi MS); Department of Public Health (H Durmuş MD), Erciyes University, Kayseri, Turkiye; Health Management Department (Prof. V DurmuŞ PhD), Kütahya Health Sciences University, Kütahya, Turkiye; Department of Infection and Tropical Medicine (O C Durojaiye MPH), University of Sheffield, Sheffield, England; Department of Pharmacology (S Dutta MD), All India Institute of Medical Sciences Rajkot, Rajkot, India; Department of Dermatology, Venereology, and Leprology (V Eddula MD), Apollo Institute of Medical Sciences and Research Chittoor, Chittoor, India; Infectious Diseases Unit (M Ekat MD), Service des Maladies Infectieuses, Brazzaville, Congo; Faculté des Sciences de la Santé (M Ekat MD), Marien Ngouabi University, Brazzaville, Congo; Department of Healthcare Policy and Financing (Y A M Elhadi PhD), Global Health Focus, Khartoum, Sudan; Faculty of Veterinary Medicine (Prof. Y S El-Sayed PhD), Damanhour University, Damanhour, Egypt; Department of Pediatrics (C Eltaha MD), University of Texas, Dallas, TX, USA; Faculty of Veterinary Medicine (Prof. A S Eltahawy PhD), Damanhour University, Damanhur, Egypt; Department of Human Anatomy (C N Esomchi MSc), State University of Medical and Applied Sciences, Igbo Eno, Nigeria; Department of Anatomy (C N Esomchi MSc), Alex Ekwueme Federal University Ndufu Alike Ikwo, Ikwo, Nigeria; Department of Environmental Health Science (P M Eze PhD), Department of Paediatrics (C A Nri-Ezedi PhD), Nnamdi Azikiwe University, Awka, Nigeria; School of Biological Sciences (P M Eze PhD), Queen's University Belfast, Belfast, UK; Research Centre for Healthcare and Community (A F Fagbamigbe PhD), Coventry University, Coventry, UK; Tanner College of Dental Medicine (Prof. A Fahim PhD), University of Pikeville, Pikeville, KY, USA; Department of Surgery, and Clinical and Public Health Research (E G Fanta MD), MyungSung Medical College, Addis Ababa, Ethiopia; Department of Public Health (W Fatima PhD), Afro-Asian Institute, Lahore, Pakistan; Department of Nursing (G Fetensa MSc), Wollega University, Nekemte, Ethiopia; Department of Biomedical, Metabolic and Neural Sciences (Prof. T Filippini PhD), University of Modena and Reggio Emilia, Modena, Italy; School of Public Health (Prof. T Filippini PhD), School of Information (F Nugen PhD), University of California Berkeley, Berkeley, CA, USA; Department of Public Health (C Fiorilla MD), University of Naples "Federico II", Naples, Italy; Institute of Public Health (F Fischer PhD), Charité Universitätsmedizin Berlin (Charité Medical University Berlin), Berlin, Germany; Institute of Public Health (A B Fissha MD), Washington University in St. Louis, St Louis, MO, USA; School of Medicine (A B Fissha MD), Addis Ababa University, Addis Ababa, Ethiopia; Department of Medical Pharmacology (F Fogacci MDc), Ataturk University, Erzurum, Turkiye; Department of Dermatology (Prof. T Fukumoto PhD), Kyoto Prefectural University of Medicine, Kyoto, Japan; School of Public Health (D Gadeka PhD), University of Ghana, Legon-Accra, Ghana; Department of Public Health (M Gajdács PhD), University of Szeged, Szeged, Hungary; Trauma and Emergency Medicine (E Ganessane MD), All India Institute of Medical Sciences (AIIMS), Madurai, Madurai, India; Infectious Diseases Unit (J Garlasco MD), University of Verona, Verona, Italy; Amity Institute of Pharmacy (Prof. R K Gautam PhD), Amity University Uttar Pradesh, Noida, India; Christian Dental College (M George Mathew PhD), Christian Medical College and Hospital (CMC), Ludhiana, India; School of Engineering and Applied Science (A Gerami Matin PhD), George Washington University, Washington, DC, USA; Tropical Health Department (R M Ghazy PhD), Alexandria University, Alexandria, Egypt; Family and Community Medicine Department (R M Ghazy PhD), King Khalid University, Abha, Saudi Arabia; Department of Biological Sciences and Chemistry (DBSC) (S A Gilani PhD), University of Nizwa, Nizwa, Oman; Department of Medicine (N Gohar MBBS), Ameer-ud-Din Medical College, Lahore, Pakistan; Department of Health Systems and Policy Research (Prof. M Golechha PhD), Indian Institute of Public Health, Gandhinagar, India; Midwifery Department (Y A Goshu MSc), Debre Tabor University, Debre Tabor, Ethiopia; Department of Microbiology (B H Gulumbe PhD), Federal University Birnin Kebbi, Birnin Kebbi, Nigeria; Department of Toxicology (S Gupta PhD), Shriram Institute for Industrial Research, Delhi, India; Department of Infectious Diseases (H Hagiya PhD), Okayama University, Okayama, Japan; Department of Maternity and Neonatal Nursing (T G Haile MSc), Department of Internal Medicine (D Kinfe MD), College of Medicine and Health Sciences (Y Leake MD), Aksum University, Aksum, Ethiopia; Department of Community Medicine (P Halder MD), Post Graduate Institute of Medical Education and Research, Chandigarh, India; Centre for Community Medicine (P Halder MD), All India Institute of Medical Sciences, New Delhi, India; Kurdistan Higher Council of Medical Specialties, Sulaymaniyah, Iraq (R M Hama Salih PhD); Faculty of Veterinary Medicine (M H Hamad PhD), Cardiovascular Department (Prof. A M A Saad MD), Department of Microbiology and Immunology (G Yahya PhD), Zagazig University, Zagazig, Egypt; Department of Animal Diseases (M H Hamad PhD), King Salman International University, Ras Sudr, Egypt; Department of Statistics and Informatics (F O Hamarasool MSc), Salahaddin University-Erbil, Erbil, Iraq; School of Health and Environmental Studies (Prof. S Hamidi DrPH), Hamdan Bin Mohammed Smart University, Dubai, United Arab Emirates; Department of Neurosurgery (A M Hamouda MD), Mayo Clinic, Rochester, NY, USA; Edirne Public Health Center (D Han Yekdeş MD), Edirne Provincial Health Directorate, Edirne, Turkiye; Department of Epidemiology, Biostatistics, Population Studies and Health Promotion (A Hargono Dr), Faculty of Public Health, Universitas Airlangga, Surabaya, Indonesia; Department of Medical Imaging (S Harsini MD), University of Toronto, Toronto, ON, Canada; Faculty of Pharmacy (A Haseeb PhD), University of Tabuk, Tabuk, Saudi Arabia; Department of Pharmaceutical Technology (Prof. M S Hasnain PhD), Adamas University, Kolkata, India; College of Medicine (Z Hein PhD), Department of Pathological Sciences (A Rehman PhD), Ajman University, Ajman, United Arab Emirates; Department of Statistics (M Hossain MSc, M Islam BSc), Shahjalal University of Science and Technology, Sylhet, Bangladesh; Institute of Child Care (A O Ikeabbah FWACP), Nnamdi Azikiwe University teaching hospital Nnewi, Nigeria, Nnewi, Nigeria; Collaborative Alliance Research and Education (CARE) Programme (A Ikiroma PhD), Episcope Research, Aberdeen, Scotland; West Africa RCC (O S Ilesanmi PhD), Africa Centre for Disease Control and Prevention, Abuja, Nigeria; Department of Community Medicine (O S Ilesanmi PhD), Department of Medicine (Prof. M O Owolabi DrM), University College Hospital, Ibadan, Ibadan, Nigeria; Faculty of Medical Sciences (Prof. M D Ilic PhD), University of Kragujevac, Kragujevac, Serbia; Department of Microbiology (K Imwattana PhD), Mahidol University, Bangkok, Thailand; Department of Pathology & Immunology (K Imwattana PhD), School of Medicine (Prof. J A Singh MD), Baylor College of Medicine, Houston, TX, USA; Nursing Department (A Ispriantari PhD), Institute of Technology and Health Science RS dr Soepraoen, Malang, Indonesia; Section of Economic & Social Sciences, Humanities & Arts (Prof. M Jakovljevic PhD), The World Academy of Sciences UNESCO-TWAS, Trieste, Italy; Shaanxi University of Technology, Hanzhong, China (Prof. M Jakovljevic PhD); Department of Primary Care Medicine (J Jamaluddin MMed), Universiti Malaya, Kuala Lumpur, Malaysia; SRM Medical College Hospital and Research Centre (J James MD), Sri Ramaswamy Memorial Institute of Science and Technology, Kattankulathur, India; Centre for Evidence Synthesis and Implementation Research (Y A Jayasinghe BSc), Cephas Health Research Initiative, Inc, Ibadan, Nigeria; Jindal School of Public Health and Human Development (Y A Jayasinghe BSc), O.P. Jindal Global University, Sonipat, India; Department of Community Medicine (N Joseph MD, R Motappa MD), Department of Forensic Medicine and Toxicology (Prof. J Padubidri MD), Manipal Academy of Higher Education, Mangalore, India; Department of Economics (C E Joshua BSc), National Open University, Benin City, Nigeria; Master of public health (F M Jundi MPH), Haramaya University, Harar, Ethiopia; Department of Emergency Medicine (D K MS), Karpaga Vinayaga Educational Lore for Learning (Deemed-to-be University), Madhuranthagam, India; Department of General Medical Practice No. 2 (Prof. S Kamenova DMedSc), Scientific and Educational Center for Neurology and Applied Neuroscience (A Kondybayeva PhD), Kazakh National Medical University, Almaty, Kazakhstan; Microbiology, Virology and Immunology Department (Prof. O Kamyshnyi DSc), I. Horbachevsky Ternopil National Medical University, Ternopil, Ukraine; Office of the Executive Director (Prof. K K Kanmodi MPH), Cephas Health Research Initiative Inc., Ibadan, Nigeria; Department of Public Health (Prof. K K Kanmodi MPH), Thomas Adewumi University, Oko, Nigeria; Department of Community Medicine (S Kannan S MD), ESIC Medical College and Hospital Chennai, Chennai, India; School of Management (Hospital and Health Care Management (R Karnam PhD), The Apollo University, Chittoor, A.P, Chittoor, India; Chair and Department of Medical Microbiology (Prof. T M Karpiński DDS), Poznan University of Medical Sciences, Poznan, Poland; Amity Institute of Forensic Sciences (H Khajuria PhD, B P Nayak PhD), Amity Institute of Public Health and Hospital Administration (M Shannawaz PhD), Amity University, Noida, India; Lahore Medical Research Center (S Khalid PhD), Lahore Medical Research Center, Lahore, Pakistan; Dr Sulaiman Al Habib Hospital (H A Khan MCHorthopedics), Sheri Kashmir Institute of Medical Sciences, Riyadh, Saudi Arabia; Department of Pharmacy (M Khan MPhil), Minhaj University Lahore, Lahore, Pakistan; Department of Community and Preventive Medicine (R Khan MD), King Edward Medical University, Lahore, Pakistan; College of Health, Wellbeing and Life Sciences (Prof. K Khatab PhD), Sheffield Hallam University, Sheffield, UK; College of Arts and Sciences (Prof. K Khatab PhD), Ohio University, Zanesville, OH, USA; Department of Biochemistry (F Khidri PhD), Liaquat University Of Medical and Health Sciences, Jamshoro, Pakistan; Department of Public Health Dentistry (Prof. S KM Kondlahalli PhD), Krishna Vishwa Vidyapeeth Deemed to be University, Karad, India; Department of Anthropology (Prof. K Krishan PhD), Panjab University, Chandigarh, India; Department of Research Management (M Kulimbet MSc), Research Institute of Cardiology and Internal Diseases, Almaty, Kazakhstan; Health Research Institute (M Kulimbet MSc), Al Farabi Kazakh National University, Almaty, Kazakhstan; Amity Institute of Biotechnology (S Kulshreshtha PhD, A Sharma PhD), Amity Centre for Water Studies and Research (S Kulshreshtha PhD), Amity University Rajasthan, Jaipur, India; Department of Community Medicine (D Kumar MD), Rajendra Institute of Medical Sciences, Ranchi, India; Wits Advanced Drug Delivery Platform Research Unit (Prof. P Kumar PhD), Wits Infectious Diseases and Oncology Research Institute (Prof. P Kumar PhD), University of the Witwatersrand, Johannesburg, South Africa; Department of Public Health Nursing (A Kusi Amponsah PhD), Department of Agricultural Economics, Agribusiness and Extension (Prof. C Wongnaa PhD), Kwame Nkrumah University of Science and Technology, Kumasi, Ghana; Department of Public Health and Epidemiology (D Kusuma DSc), Khalifa University of Science and Technology, Abu Dhabi, United Arab Emirates; Faculty of Public Health (D Kusuma DSc), University of Indonesia, Depok, Indonesia; Clinical Research Center (V Kytö MD), Turku University Hospital, Turku, Finland; Heart Center (V Kytö MD), University of Turku and Turku University Hospital, Turku, Finland; Division of Evidence Synthesis (C Lahariya MD), Foundation for People-centric Health Systems, New Delhi, India; Division of Lifestyle Medicine (C Lahariya MD), Centre for Health: The Specialty Practice, New Delhi, India; Department of Clinical and Experimental Medicine (Prof. C Ledda PhD), University of Catania, Catania, Italy; Faculty of Electronic Engineering and Technology (H Lee PhD), Universiti Malaysia Perlis, Arau, Malaysia; Department of Precision Medicine (Prof. S Lee MD), Sungkyunkwan University, Suwon-si, South Korea; Clinical Sciences Department (A Liaqat FCPS), Shaqra University, Shaqra, Saudi Arabia; Faculty of Health Sciences (A Lin MD), University of Macau, Macau, China; School of Medicine (J López-Gil PhD), Universidad Espíritu Santo, Samborondón, Ecuador; Vicerrectoría de Investigación y Postgrado (J López-Gil PhD), Universidad de Los Lagos, Osorno, Chile; Shantou University Medical College (P Luo MD), Shantou University Medical College, Shantou, China; Department of Medicine (E Lytvyak MD), University of Alberta, Edmonton, AB, Canada; Tulane School of Public Health and Tropical Medicine (E Lytvyak MD), Tulane University, New Orleans, LA, USA; Veterinary Pathology Department (Prof. F Maboni Siqueira PhD), Federal University of Rio Grande do Sul, Porto Alegre, Brazil; Department of Microbiology and Parasitology (M Mabrok PhD), King Salman International University, South Sinai, Egypt; Department of Periodontology (Prof. M Machoy-Rakoczy PhD), Pomeranian Medical University, Szczecin, Poland; Department of Physiology and Bioc Sulaiman hemistry (M Mackei PhD), University of Veterinary Medicine Budapest, Budapest, Hungary; Department of Pharmaceutical Sciences (E Magwe PhD), Ruaha Catholic University (RUCU), Iringa, Tanzania; Department of Emergency Medicine and Trauma (S Mahalingam MD), Karpaga Vinayaga Institute of Medical Sciences and Research Center, Karpaga Vinayaga Educational Lore for Learning (Deemed-to-be-University), Maduranthagam Taluk, Chengalpattu District, TamilNadu, Madhuranthagam, India; Research Center (N H Mahmood PhD), Cihan University-Sulaimaniya, Sulaymaniyah, Iraq; Division of Research & Development (Prof. T Malik PhD), Lovely Professional University, Phagwara, India; Department of Biology (K Maqsood PhD), Lahore Garrison University, Lahore, Pakistan; Department of Infectious Diseases (B A Martinez-Guerra MSc), Instituto Nacional de Nutrición Salvador Zubirán (Salvador Zubiran National Institute of Medical Sciences and Nutrition), Mexico City, Mexico; Department of Community Medicine (M Mathur MD), Geetanjali Medical College and Hospital in Udaipur India, Udaipur, India; Department of Maternal-Child Nursing and Public Health (Prof. F P Matozinhos PhD), Federal University of Minas Gerais, Belo Horizonte, Brazil; Sir Salimullah Medical College & Mitford hospital (P Mazumder MSc), Institute of Nutrition and Food Science (M Sultana PhD), University of Dhaka, Dhaka, Bangladesh; Department of Surgery (U C Mba FMCS), Enugu State University of Science and Technology, Enugu, Nigeria; Department of Surgery (U C Mba FMCS), ESUT Teaching Hospital, Enugu, Nigeria; College of Medicine and Health Science (A A Mekonnen PhD), Department of Medical Laboratory Science (A M Zuber MSc), Wachemo University, Hosanna, Ethiopia; College of Medicine and Department of Nursing (A A Mekonnen PhD), Komar University of Science and Technology, Kurdistan Sulemaniya, Iraq; Department of Medical Laboratory Sciences (H N Meles MSc), Adigrat University, Adigrat, Ethiopia; University Centre Varazdin (T Mestrovic PhD), University North, Varazdin, Croatia; Research Center for Public Health and Nutrition (M Mikrajab MPH), National Research and Innovation Agency, Bogor District, Indonesia; Division of Allied Health Sciences, School of Health Sciences (B Mishra MSc), The Apollo University, Chittoor, India; Molecular Biology Unit (N S Mohamed MSc), Bio-Statistical and Molecular Biology Department (N S Mohamed MSc), Sirius Training and Research Centre, Khartoum, Sudan; Department of Pathophysiology (K Mohamed Ahmed MD), St. George's University, St. George’s, Grenada; Medical Microbiology Department (Prof. Y Mohammed FWACP), Department of Veterinary Public Health and Preventive Medicine (A Shittu MSc), Department of Pharmaceutical Microbiology and Biotechnology (S Shuaibu PhD), Usmanu Danfodiyo University, Sokoto, Sokoto, Nigeria; Medical Microbiology Department (Prof. Y Mohammed FWACP), Usmanu Danfodiyo University Teaching Hospital, Sokoto, Nigeria; Clinical Epidemiology and Public Health Research Unit (L Monasta DSc, L Ronfani PhD, G Zamagni MSc), Burlo Garofolo Institute for Maternal and Child Health, Trieste, Italy; Unit of Pharmacotherapy, Epidemiology and Economics (Prof. S Mubarik PhD), University of Groningen (Rijksuniversiteit Groningen), Groningen, Netherlands; Department of Epidemiology and Biostatistics (Prof. S Mubarik PhD), Wuhan University, Wuhan, China; Department of Pediatrics & Pediatric Pulmonology (Prof. G Mustafa MD), Institute of Mother & Child Care, Multan, Pakistan; College of Health Sciences (H H R Najmuldeen PhD), Cihan University Sulaimaniya, Sulaimaniya, Iraq; University of Sulaimani, Sulaymaniyah, Iraq (H H R Najmuldeen PhD); Department of Neurosciences (Prof. C R J Newton MD), Kenya Medical Research Institute/Wellcome Trust Research Programme, Kilifi, Kenya; Institute for Global Health Innovations (C T Nguyen MPH), Duy Tan University, Da Nang, Viet Nam; Faculty of Public Health (T Nguyen PhD), VNU University of Medicine and Pharmacy, Hanoi, Viet Nam; Faculty of Medicine (T Nguyen PhD), Duy Tan University, Da Nang, Viet Nam; School of Medicine, Health Research Institute (V Niranjan PhD), University of Limerick, Limerick, Ireland; Department of Radiology (F Nugen PhD), Mayo Clinic, Rochester, MN, USA; Department of Medical Microbiology, University of Ghana Medical School (A Odoom BSc), University of Ghana, Accra, Ghana; Translational Health Research Institute (F A Ogbo PhD), Western Sydney University, Sydney, NSW, Australia; Department of Human Anatomy (F O Ojo PhD), University of Ilesa, Ilesa Osun State, Ilesa, Nigeria; School of Pharmacy (O C Okonji MSc), University of the Western Cape, Cape Town, South Africa; Department of Pharmaceutical Health Outcomes and Policy (M C Okonkwo BPharm), University of Houston, Texas, Houston, TX, USA; Department of Community Medicine (L N Okoro MBBS), David Umahi Federal University Teaching Hospital, Uburu, Nigeria; Department of Community Medicine (L N Okoro MBBS), David Umahi Federal University of Health Sciences, Uburu, Nigeria; Department of Biology, Microbiology and Science Laboratory Technology (M O Olaitan MSc), Nile University of Nigeria, Abuja, Nigeria; Department of Animal Medicine (A Oreiby PhD), Kafrelsheikh University, Kafr El Sheikh, Egypt; Public Health Department (T Osei Mphil), Akenten Appiah-Menka University of Skills Training and Entrepreneurial Development, Kumasi, Ghana, Kumasi, Ghana; Department of Agribusiness (O A Otekunrin PhD), Federal University of Agriculture and Development Studies, Iragbiji (FUADSI), Nigeria, Iragbiji, Nigeria; Disaster Management Training and Education Centre for Africa (DiMTEC) (O A Otekunrin PhD), University of the Free State, Bloemfontein, South Africa; Division of Infectious Diseases (Prof. A Ouyahia PhD), University Hospital of Setif, Sétif, Algeria; Department of Biological Sciences (K A Oyeniran PhD), Bamidele Olumilua University of Education Science & Technology, Ikere-Ekiti, Nigeria; Plant Systems Biology (K A Oyeniran PhD), International Center for Genetic Engineering & Biotechnology (ICGEB), Cape Town, South Africa; Irfan Suat Gunsel Operational Research Institute (I Ozsahin PhD, Prof. U Saeed PhD, Prof. Y Waheed PhD), Near East University, Nicosia, Turkiye; Department of Sociology, Anthropology, and Public Health (C Park DrPH), University of Maryland Baltimore County, Baltimore, MD, USA; College of Dental Medicine (Prof. S Patil PhD), Roseman University of Health Sciences, South Jordan, UT, USA; Department of Community Medicine (M Patni MD), RAK Medical and Health Sciences University, UAE, Ras Al Khaimah, United Arab Emirates; Lviv State University of Physical Culture, Lviv, Ukraine (Prof. I Pavlova DSc); Helsinki Collegium of Advanced Studies (Prof. I Pavlova DSc), University of Helsinki, Helsinki, Finland; Széchenyi István University, Győr, Hungary (Z Z Piracha PhD); International Center of Medical Sciences Research (ICMSR), Islamabad, Pakistan (Z Z Piracha PhD); Rory Meyers College of Nursing (X Qi PhD), New York University, New York, NY, USA; St. Cyril and Methodius University- Skopje, North Macedonia, Skopje, North Macedonia (I Radenkov MD); Department of Medical Biology (Prof. F Rahim PhD), Kocaeli University, Izmit, Turkiye; Internal Medical Faculty (Prof. F Rahim PhD), Osh state university, Osh, Kyrgyzstan; Department of Public Health (Prof. B Rahimi PhD), Afghan International Islamic University in Kabul, Afghanistan, Kabul, Afghanistan; Department of Pediatrics (Prof. B Rahimi PhD), Kandahar University in Kandahar, Afghanistan, Kandahar, Afghanistan; Department of Population Science and Human Resource Development (Prof. M Rahman DrPH), University of Rajshahi, Rajshahi, Bangladesh; Division of Clinical Pharmacology (J P Raj DM), Kasturba Medical College Mangalore (M Rao MD), Manipal College of Dental Sciences, Mangalore (Prof. P K Shetty MDS), Manipal Academy of Higher Education, Manipal, India; Trauma and Emergency Medicine Department (G Rajendran MD), All India Institute of Medical Sciences (AIIMS), Madurai, India; Department of Cardiology (Prof. M M Ramadan PhD), Faculty of Pharmacy (Prof. M A Saleh PhD), Faculty of Nursing (M Zoromba PhD), Mansoura University, Mansoura, Egypt; Department of Midwifery (K Ramadhan MPH), Ministry of Health of the Republic of Indonesia, Palu, Indonesia; College of Nursing (L Ramamoorthy PhD), Jawaharlal Institute of Postgraduate Medical Education and Research, puducherry, India; Centre for Clinical Pharmacology (Prof. N Rancic PhD), Military Medical Academy, Belgrade, Serbia; Department of Biological Sciences (Prof. E M Redwan PhD), King Abdulaziz University, Jeddah, Saudi Arabia; Department of Protein Research (Prof. E M Redwan PhD), Research and Academic Institution, Alexandria, Egypt; The School of Pharmaceutical Sciences (W Rehman MS), University of Science Malaysia, Penang, Malaysia; College of Nursing (Y Rias MNS), Institut Ilmu Kesehatan Bhakti Wiyata Kediri, Kediri, Indonesia; Department of Veterinary Microbiology (P Roy PhD), College of Veterinary Science and Animal Husbandry, Agartala, India; Széchenyi István University, Győr, Hungary (Prof. U Saeed PhD); College of Applied and Health Sciences (A Sah PhD), A' Sharqiyah University, Ibra, Oman; Department of Epidemiology (Prof. I Saha PhD), Indian Council of Medical Research, Kolkata, India; Biotechnology Research Center (Prof. A Sahebkar PhD), Mashhad University of Medical Sciences, Mashhad, Iran; Centre for Research Impact and Outcome (Prof. A Sahebkar PhD), Chitkara University, Rajpura, India; LMU-Munich, Munich, Germany (J W Sakshaug PhD); Institute for Employment Research, Nuremberg, Germany (J W Sakshaug PhD); Department of Human Nutrition & Dietetics (S Saleem PhD), Health Services Academy, Islamabad, Pakistan; Department of Biochemistry and Molecular Biology (M A Sanusi MSc), Obafemi Awolowo University, Ile-Ife, Nigeria; Indira Gandhi Medical College and Research Institute, Puducherry, India (A Saravanan MD); Faculty of Health & Social Sciences (B Sathian PhD), Bournemouth University, Bournemouth, England; Department of Epidemiology (M Sathya Narayanan MBBS), National Institute for Research in Tuberculosis, Chennai, India; Emergency Department (S Senthilkumaran PhD), Manian Medical Centre, Erode, India; Department of Medicine (Y Sethi MD), Swami Vivekanand Subharti University, Meerut, India; Ashok & Rita Patel Institute of Physiotherapy (D S Shah MPT), Charotar University of Science and Technology, Changa, India; School of Health Sciences (S Shaharudin PhD), Universiti Sains Malaysia, Kota Bharu, Malaysia; National Institute of Education (S Shaharudin PhD), Nanyang Technological University, Singapore, Singapore; Department of Pathology and Laboratory Medicine (S Sham MD), Northwell Health, New York, NY, USA; Department of Plantation Spices Medicinal and Aromatic Crops (Prof. A B Sharangi PhD), Bidhan Chandra Krishi Viswavidyalaya Agricultural University in India, Mohanpur, India; School of Agriculture, Food and Ecosystem Sciences (Prof. A B Sharangi PhD), University of Melbourne, Burnley, VIC, Australia; Department of Microbiology and Molecular Genetics (N Sharif MSc), University of Vermont, Burlington, VT, USA; Department of Microbiology (N Sharif MSc), Jahangirnagar University, Dhaka, Bangladesh; Department of Forensic Science (Prof. B K Sharma PhD), Shree Guru Gobind Singh Tricentenary University, Gurugram, India; Division of Physical Sciences (Prof. K Sharma PhD), Karunya Institute of Technology and Sciences, Tamil Nadu, India; Department of University Institute of Biotechnology (R Sharma PhD), Chandigarh University, Mohali, India; Department of Nursing (B B Shashamo MSc), Arba Minch University, Arba Minch, Ethiopia; Alva's Institute of Medical Sciences & Research Centre (Prof. M Shetty MD), Rajiv Gandhi University of Health Sciences, Moodubidire, India; KS Hegde Medical Academy (Prof. P Shetty MD), Nitte University, Mangalore, India; School of Biotechnology (K Shivangi MSc), Gautam Buddha University, Gautam Buddha Nagar, India; Department of Medical-Surgical Nursing (S Shorofi PhD), Mazandaran University of Medical Sciences, Sari, Iran; Department of Nursing and Health Sciences (S Shorofi PhD), Flinders University, Adelaide, SA, Australia; School of Pharmaceutical Sciences (H Singh PhD), IFTM University, Moradabad, India; Department of Medicine Service (Prof. J A Singh MD), US Department of Veterans Affairs (VA), Houston, TX, USA; Amity Institute of Public Health and Hospital Administration (A P Sinha PhD), Amity University Noida, Delhi, India; Çubuk District Health Directorate (Y solak PhD), Republic of Türkiye Ministry of Health, Ankara, Turkiye; Faculty of Pharmacy (A M Soliman PhD), Kafrelsheikh University, Kafr El-Sheikh, Egypt; Faculty of Science (A M Soliman PhD), University of Regina, Regina, SK, Canada; Department of Medicine (Y Song MD), Washington University in St. Louis, Saint Louis, MO, USA; Department of Endocrinology (A Sood MD), Case Western Reserve University, Cleveland, OH, USA; Department of Health Sciences (Prof. A Spagnolo PhD), University of Genoa, Genoa, Italy; Department of Public Policy and Management (J Sumankuuro PhD), University of Business and Integrated Development Studies, Wa, Ghana; School of Nursing (J Sumankuuro PhD), Charles Sturt University, Bathurst, NSW, Australia; Squad Medicine and Research (SMR) (T Suvvari MD), Indian Council of Medical Research, Amadalavalasa, India; Department of Health, Safety, and Environmental Management (R Tabibi PhD), Abadan University of Medical Sciences, Abadan, Iran; Zhujiang Hospital (J Tan MD), Southern Medical University, Guangzhou, China; The First Affiliated Hospital of Chongqing Medical University (J Tan MD), Chongqing Medical University, Chongqing, China; Department of Neonatology (R Thiruvengadam PhD), Saveetha Dental College and Hospitals (M Tovani-Palone PhD), Saveetha University, Chennai, India; Department of Medicine (C S Thornton MD), University of Calgary, Calgary, AB, Canada; Research and Advocacy Initiative (N Tran Minh Duc MD), ALS Viet Nam, Quang Ngai, Viet Nam; Hamilton Eye Institute (A Trikkur Madom Seetharaman PhD), University of Tennessee, Memphis, TN, USA; Department of Internal Medicine (M Tumurkhuu PhD), Wake Forest University, Winston-Salem, NC, USA; Department of Statistics (Prof. M Uddin PhD), Shahjalal University of Science and Technology (SUST), Sylhet, Bangladesh; Faculty of Graduate Studies (Prof. M Uddin PhD), Daffodil International University (DIU), Dhaka, Bangladesh; University Hospitals Cleveland Medical Center, Cleveland, OH, USA (I Ullah MBBS); Department of Physiotherapy (L Umar PhD), Federal Ministry of Health, Gusau, Nigeria; Department of Studies and Research in Biochemistry (D Urs PhD), Mangalore University, Kodagu, India; Department of Orthodontics (H Uzunçıbuk PhD), University of Trakya, Edirne, Turkiye; Department of Public Health, Experimental and Forensic Medicine (G Vigezzi DrPH), Università degli Studi di Pavia, Pavia, Italy; Henry M.Goldman School of Dental Medicine (V Vikram MDS), Boston University, Boston, MA, USA; VIZJA University, Warsaw, Poland (Prof. Y Waheed PhD); Shandong Provincial Hospital Affiliated to Shandong First Medical University, Jinan, China (X Wang MD); Department of Pharmaceutical Chemistry College of Pharmacy (Prof. T Wani PhD), Department of Biochemistry (Prof. S Zargar PhD), King Saud University, Riyadh, Saudi Arabia; Department of Community Medicine (N Wickramasinghe MD), Rajarata University of Sri Lanka, Anuradhapura, Sri Lanka; Department of Experimental Dentistry (Prof. M Wieckiewicz PhD), Wroclaw Medical University, Wroclaw, Poland; Department of Cells and Tissues (G Yahya PhD), Molecular Biology Institute of Barcelona, Barcelona, Spain; Department of Hepatobiliary Surgery (H Yang PhD), Capital Medical University, Beijing, China; Department of Health Management (Prof. V Yiğit PhD), Süleyman Demirel Üniversitesi (Süleyman Demirel University), Isparta, Turkiye; Department of Biostatistics (Prof. N Yonemoto PhD), University of Toyama, Toyama, Japan; Department of Public Health (Prof. N Yonemoto PhD), Juntendo University, Tokyo, Japan; Faculty of Medicine and Health Sciences (F Zakham PhD), Hodeidah University, Hodeidah, Yemen; Department of Medical and Surgical Sciences (A Zanghì MD), University of Foggia, Foggia, Italy; Department of Zoology and Entomology (M G M Zeariya PhD), Al-Azhar University, Cairo, Egypt; Center for Applied Molecular Biology (CAMB) (B Zeshan PhD), University of The Punjab, Lahore, Pakistan; Department of Pediatrics (Prof. D Yon MD), Kyung Hee University Medical Center, Kyung Hee University College of Medicine, Seoul, South Korea

**Authors’ Contributions**

Providing data or critical feedback on data sources

Aleksandra Barac, Nadin M I Abdel Razeq, Jeza Muhamad Abdul Aziz, Abdulrakib Abdulrahim, Olifan Zewdie Abil, Richard Gyan Aboagye, Khaled M Aboshanab, Amr Selim Abu Lila, Tajudeen Adesanmi Adebisi, Qorinah Estiningtyas Sakilah Adnani, Muhammad Sohail Afzal, Saira Afzal, Gizachew Beykaso Agafari, Muayyad M Ahmad, Ali Ahmed, Ayman Ahmed, Gasha Salih Ahmed, Muhammad Nadeem Akhtar, Salah Al Awaidy, Mushtak Talib Salih Al-Ouqaili, Ahmad Rajeh Al-Qudimat, Ali Salman Al-Shami, Raed M Al-Zoubi, Mohammed Albashtawy, Ali M Alfalki, Abdelazeem M Algammal, Mohammed Alissa, Alaa A. A. Aljabali, Sameer Abdulmalik Alkubati, Mohammed Z. Allouh, Joseph Uy Almazan, Mohammed Almunef, Khaldoon Aied Alnawafleh, Abdalkarem Fedgash Alsharari, Awais Altaf, Kassaye D. Demeke Altaye, Nelson Alvis-Guzman, Hany Aly, Razique Anwer, Muhammad Shahzad Aslam, Alok Atreya, Usman Ayub Awan, Habtamu Molla Ayele, AKM Azad, Ahmed Y Azzam, Syed Babar Jamal Bacha, Riadh Badraoui, Khlood K Baghlaf, Senthilkumar Balakrishnan, Dennis Bardoe, Hiba Jawdat Barqawi, Shazia Batool, Nebiyou Simegnew Bayleyegn, Narasimha M Beeraka, Desalegn Weldebrhan Bekru, Samiun Nazrin Bente Kamal Tune, Sonu Bhaskar, Jasvinder Singh Bhatti, Md Mehedi Hasan Bhuiyan, Souad Bouaoud, Christopher Boxe, Sayed Muhammad Ata Ullah Shah Bukhari, Vijay Kumar Chattu, Lam Duc Chau, Akhilanand Chaurasia, Bashir Dabo, Xiaochen Dai, Fernando Pio De la Hoz, Bryan Gervais de Liyis, Sindhura Deekonda, Ivan Delgado-Enciso, Vinoth Gnana Chellaiyan Devanbu, Devananda Devegowda, Marcello Di Pumpo, Ojas Prakashbhai Doshi, Robert Kokou Dowou, Rabie Adel El Arab, Yasser S. El-Sayed, Yasir Ahmed Mohammed Elhadi, Chadi Eltaha, Abdelgawad Salah Eltahawy, Collins Nduka Esomchi, Adeniyi Francis Fagbamigbe, Ayesha Fahim, Ebenezer Gezahegn Fanta, Aisha Farhana, Aemon Berhane Fissha, Takeshi Fukumoto, Ezhilkugan Ganessane, Mebin George Mathew, Ali Gerami Matin, Ramy Mohamed Ghazy, Sama Ghoba, Syed Abdullah Gilani, Najam Gohar, Mahaveer Golechha, Yitayal Ayalew Goshu, Sapna Gupta, Awoke Derbie Habteyohannes, Teklehaimanot Gereziher Haile, Pritam Halder, Raz Mohammed Hama Salih, Mohamed H. Hamad, Arief Hargono, Sara Harsini, Simon I Hay, Zaw Myo Hein, MD Talib Hussain, Segun Emmanuel Ibitoye, Olayinka Stephen Ilesanmi, Korakrit Imwattana, Mihajlo Jakovljevic, Arshad Jamal, Jerin James, Yovanthi Anurangi Jayasinghe, Bijay Mukesh Jeswani, Charity Ehimwenma Joshua, Fozi Musa Jundi, Dhinesh Babu K, Divya K L, Saltanat Kamenova, Oleksandr Kamyshnyi, Reddy Praneeth Karnam, Tomasz M Karpiński, Himanshu Khajuria, Hayat Ahmad Khan, Muhammad Arslan Khan, Raees Khan, Ramsha Mushtaq Khan, Khaled Khatab, Feriha Fatima Khidri, Daniel Gebrehawaria Kinfe, Shivakumar KM Kondlahalli, Aida Kondybayeva, Kewal Krishan, Shweta Kulshreshtha, Dewesh Kumar, Pradeep Kumar, Dian Kusuma, Ville Kytö, Chandrakant Lahariya, Yemane Leake, Caterina Ledda, Hoi Leong Lee, Seung Won Lee, Ellina Lytvyak, Franciele Maboni Siqueira, Monika Machoy-Rakoczy, Máté Mackei, Sasikumar Mahalingam, Nozad Hussein Mahmood, Aseer Manilal, Kaleem Maqsood, Bernardo Alfonso Martinez-Guerra, Medha Mathur, Bhagyashree Mishra, Nouh Saad Mohamed, Khabab Abbasher Hussien Mohamed Ahmed, Yahaya Mohammed, Ali H Mokdad, Lorenzo Monasta, Rohith Motappa, Sumaira Mubarik, Christopher J L Murray, Ghulam Mustafa, Zuhair S Natto, Biswa Prakash Nayak, Nini Asfaw Negash, Cuong Tat Nguyen, Trang Nguyen, Ali Aahil Noorali, Emira Noumi, Fred Nugen, Alex Odoom, Osaretin Christabel Okonji, Morufat Oluwatosin Olaitan, Amel Ouyahia, Mayowa O Owolabi, Kehinde Adewole Oyeniran, Jagadish Rao Padubidri, Chulwoo Park, Shankargouda Patil, Iuliia Pavlova, Zahra Zahid Piracha, Amit Porwal, Ishu Poudyal, Xiang Qi, Igor Radenkov, Md Sazedur Rahman, Jeffrey Pradeep Raj, Gunaseelan Rajendran, Mahmoud Mohammed Ramadan, Kadar Ramadhan, Lakshmi Ramamoorthy, Nemanja Rancic, Mithun Rao, Elrashdy M Redwan, Abdul Rehman, Wajiha Rehman, Yohanes Andy Rias, Luca Ronfani, Aly M A Saad, Umar Saeed, Ashok Kumar Sah, Fatemeh Saheb Sharif-Askari, Narjes Saheb Sharif-Askari, Joseph W Sakshaug, Samreen Saleem, Malik Adebayo Sanusi, Benn Sartorius, Brijesh Sathian, Subramanian Senthilkumaran, Yashendra Sethi, Sunder Sham, Mohammed Shannawaz, Nadim Sharif, Avimanu Sharma, Bhoopesh Kumar Sharma, Kamlesh Sharma, Ravi Kumar Sharma, Mahabalesh Shetty, Premalatha K Shetty, Aminu Shittu, Km Shivangi, Suleiman Adeiza Shuaibu, Harpreet Singh, Jasvinder A Singh, Abhijeet Prasad Sinha, Mejdi Snoussi, Ahmed M Soliman, Yizhe Song, Aayushi Sood, Marjia Sultana, Joshua Sumankuuro, Tarun Kumar Suvvari, Ramin Tabibi, Jianye Tan, Christina S Thornton, Marcos Roberto Tovani-Palone, Munkhtuya Tumurkhuu, Md Jamal Uddin, Irfan Ullah, Lawan Umar, Deepadarshan Urs, Hande Uzunçıbuk, Giacomo Pietro Vigezzi, Tuan Vinh, Yasir Waheed, Xingxin Wang, Tanveer Wani, Mieszko Wieckiewicz, Camillus Abawiera Wongnaa, Hanwen Yang, Vahit Yiğit, Naohiro Yonemoto, Seema Zargar, and Mohamed Ali Zoromba.

Developing methods or computational machinery

Xiaochen Dai, Sama Ghoba, Simon I Hay, Ali H Mokdad, Christopher J L Murray, Noga Shalev, and Dong Keon Yon.

Providing critical feedback on methods or results

Sherief Abd-Elsalam, Nadin M I Abdel Razeq, Meriem Abdoun, Jeza Muhamad Abdul Aziz, Abdulrakib Abdulrahim, Mesfin Abebe, Olifan Zewdie Abil, Richard Gyan Aboagye, Khaled M Aboshanab, Amr Selim Abu Lila, Aishah Fadila Adamu, Tajudeen Adesanmi Adebisi, Ayo Stephen Adebowale, Daniel Adeyemi Adepoju, Belete Muluadam Admassie, Qorinah Estiningtyas Sakilah Adnani, Aanuoluwapo Adeyimika Afolabi, Muhammad Sohail Afzal, Saira Afzal, Gizachew Beykaso Agafari, Percival Delali Agordoh, Salome Wumpini Agordoh, Abdul-Raheem Folorunsho Ahmad, Khabir Ahmad, Muayyad M Ahmad, Ali Ahmed, Ayman Ahmed, Gasha Salih Ahmed, Meqdad Saleh Ahmed, Mohamed Mustaf Mustaf Ahmed, Oluwasefunmi Akeju, Akil Akhtar, Muhammad Nadeem Akhtar, Olumide Odunayo Akinniyi, Karolina Akinosoglou, Salah Al Awaidy, Mushtak Talib Salih Al-Ouqaili, Ahmad Rajeh Al-Qudimat, Ali Salman Al-Shami, Raed M Al-Zoubi, Muath A Alammar, Mohammed Albashtawy, Ali M Alfalki, Abdelazeem M Algammal, Mohammad Daud Ali, Mohammed Usman Ali, Mohammed Alissa, Alaa A. A. Aljabali, Sameer Abdulmalik Alkubati, Mohammed Z. Allouh, Joseph Uy Almazan, Mohammed Almunef, Khaldoon Aied Alnawafleh, Mohammad R Alqudimat, Najim Z Alshahrani, Abdalkarem Fedgash Alsharari, Zaid Altaany, Awais Altaf, Kassaye D. Demeke Altaye, Nelson Alvis-Guzman, Hany Aly, Nada A. Alzunaidy, Mohammad Azam Ansari, Boluwatife Stephen Anuoluwa, Razique Anwer, Mosab Arafat, Muhammad Shahzad Aslam, Maha Moh'd Wahbi Atout, Alok Atreya, Matteo Augello, Usman Ayub Awan, Kemal Lemnuro Awol, Habtamu Molla Ayele, AKM Azad, Ahmed Y Azzam, Syed Babar Jamal Bacha, Riadh Badraoui, Nayereh Baghcheghi, Khlood K Baghlaf, Senthilkumar Balakrishnan, Dennis Bardoe, Hiba Jawdat Barqawi, Shazia Batool, Ravi Batra, Nebiyou Simegnew Bayleyegn, Narasimha M Beeraka, Desalegn Weldebrhan Bekru, Asnake Gashaw Belayneh, Samiun Nazrin Bente Kamal Tune, Pankaj Bhardwaj, Sonu Bhaskar, Jasvinder Singh Bhatti, Md Mehedi Hasan Bhuiyan, Trupti Bodhare, Eyob Ketema Bogale, Souad Bouaoud, Christopher Boxe, Sayed Muhammad Ata Ullah Shah Bukhari, Yasser Bustanji, Zahid A Butt, Bindiya C C Gowda, Rama Mohan Chandika, Vijay Kumar Chattu, Lam Duc Chau, Akhilanand Chaurasia, Haiyan Chen, Arrigo Francesco Giuseppe Cicero, Bashir Dabo, Omid Dadras, Xiaochen Dai, Fernando Pio De la Hoz, Bryan Gervais de Liyis, Sindhura Deekonda, Ivan Delgado-Enciso, Vinoth Gnana Chellaiyan Devanbu, Devananda Devegowda, Syed Masudur Rahman Dewan, Giuseppe Di Martino, Marcello Di Pumpo, Ojas Prakashbhai Doshi, Robert Kokou Dowou, Hasan Durmuş, Oyewole Christopher Durojaiye, Siddhartha Dutta, Venkata Ravikanth Eddula, Martin Herbas Ekat, Rabie Adel El Arab, Yasser S. El-Sayed, Muhammed Elhadi, Yasir Ahmed Mohammed Elhadi, Chadi Eltaha, Abdelgawad Salah Eltahawy, Simachew Getaneh Endalamew, Collins Nduka Esomchi, Peter Maduabuchi Eze, Adeniyi Francis Fagbamigbe, Ayesha Fahim, Ebenezer Gezahegn Fanta, Aisha Farhana, Wafa Fatima, Olufunmilayo Ibitola Fawole, Getahun Fetensa, Tommaso Filippini, Claudio Fiorilla, Florian Fischer, Aemon Berhane Fissha, Federica Fogacci, Takeshi Fukumoto, Márió Gajdács, Ezhilkugan Ganessane, Jacopo Garlasco, Rupesh K Gautam, Mebin George Mathew, Ali Gerami Matin, Shumiye Shiferaw Gessess, Ramy Mohamed Ghazy, Syed Abdullah Gilani, Najam Gohar, Mahaveer Golechha, Yitayal Ayalew Goshu, Bashar Haruna Gulumbe, Sapna Gupta, Awoke Derbie Habteyohannes, Hideharu Hagiya, Teklehaimanot Gereziher Haile, Pritam Halder, Raz Mohammed Hama Salih, Mohamed H. Hamad, Fatimah Othman Hamarasool, Samer Hamidi, Abdelrahman M Hamouda, Didem Han Yekdeş, Sara Harsini, Abdul Haseeb, Md Saquib Hasnain, Ibrahim Nagmeldin Hassan, Simon I Hay, Zaw Myo Hein, Abdelaziz Hendy, Md Sabbir Hossain, MD Talib Hussain, Segun Emmanuel Ibitoye, Sumaira Idrees, Ann Oluchi Ikeabbah, Adalia Ikiroma, Olayinka Stephen Ilesanmi, Irena M Ilic, Milena D Ilic, Korakrit Imwattana, Md. Mojahedul Islam, Aloysia Ispriantari, Mihajlo Jakovljevic, Arshad Jamal, Jazlan Jamaluddin, Jerin James, Yovanthi Anurangi Jayasinghe, Bijay Mukesh Jeswani, Nitin Joseph, Charity Ehimwenma Joshua, Fozi Musa Jundi, Dhinesh Babu K, Divya K L, Saltanat Kamenova, Oleksandr Kamyshnyi, Kehinde Kazeem Kanmodi, Suthanthira Kannan S, Reddy Praneeth Karnam, Tomasz M Karpiński, Awoke Amare Kebede, Himanshu Khajuria, Hayat Ahmad Khan, Muhammad Arslan Khan, Raees Khan, Ramsha Mushtaq Khan, Yusuf Saleem Khan, Khaled Khatab, Feriha Fatima Khidri, Daniel Gebrehawaria Kinfe, Shivakumar KM Kondlahalli, Aida Kondybayeva, Hamid Reza Koohestani, Vladimir Andreevich Korshunov, Kewal Krishan, Shweta Kulshreshtha, Dewesh Kumar, Pradeep Kumar, Abigail Kusi Amponsah, Dian Kusuma, Ville Kytö, Chandrakant Lahariya, Yemane Leake, Caterina Ledda, Hoi Leong Lee, Hwamin Lee, Seung Won Lee, Ambreen Liaqat, Anqi Lin, Madeeha Shahzad Lodhi, Peng Luo, Ellina Lytvyak, José Francisco López-Gil, Franciele Maboni Siqueira, Mahmoud Mabrok, Monika Machoy-Rakoczy, Máté Mackei, Sasikumar Mahalingam, Nozad Hussein Mahmood, Tabarak Malik, Aseer Manilal, Kaleem Maqsood, Bernardo Alfonso Martinez-Guerra, Medha Mathur, Fernanda Penido Matozinhos, Prosanjit Mazumder, Uwakwe C Mba, Agezegn Asegid Mekonnen, Hadush Negash Meles, Tomislav Mestrovic, Muhammad Agus Mikrajab, Bhagyashree Mishra, Nouh Saad Mohamed, Khabab Abbasher Hussien Mohamed Ahmed, Yahaya Mohammed, Ali H Mokdad, Lorenzo Monasta, Rohith Motappa, Sumaira Mubarik, Christopher J L Murray, Ghulam Mustafa, Hastyar Hama Rashid Najmuldeen, Zuhair S Natto, Biswa Prakash Nayak, Nini Asfaw Negash, Charles Richard James Newton, Cuong Tat Nguyen, Trang Nguyen, Vikram Niranjan, Ali Aahil Noorali, Emira Noumi, Chisom Adaobi Nri-Ezedi, Fred Nugen, Alex Odoom, Felix Akpojene Ogbo, Osaretin Christabel Okonji, Michael Chinyem Okonkwo, Morufat Oluwatosin Olaitan, Atef Oreiby, Thompson Osei, Amel Ouyahia, Mayowa O Owolabi, Kehinde Adewole Oyeniran, Ilker Ozsahin, Jagadish Rao Padubidri, Chulwoo Park, Sameena Parveen, Shankargouda Patil, Mohamed Anas Patni, Iuliia Pavlova, Zahra Zahid Piracha, Amit Porwal, Xiang Qi, Fakher Rahim, Bilal Ahmad Rahimi, Md Sazedur Rahman, Mosiur Rahman, Jeffrey Pradeep Raj, Gunaseelan Rajendran, Mahmoud Mohammed Ramadan, Kadar Ramadhan, Lakshmi Ramamoorthy, Nemanja Rancic, Mithun Rao, Elrashdy M Redwan, Abdul Rehman, Wajiha Rehman, Yohanes Andy Rias, Aly M A Saad, Umar Saeed, Ashok Kumar Sah, Indranil Saha, Fatemeh Saheb Sharif-Askari, Narjes Saheb Sharif-Askari, Joseph W Sakshaug, Samreen Saleem, Mohamed A Saleh, Malik Adebayo Sanusi, Benn Sartorius, Brijesh Sathian, Mukesh Kumar Sathya Narayanan, Subramanian Senthilkumaran, Yashendra Sethi, Muhammad Shabbir, Disha Sanjivkumar Shah, Liloma Shah, Syed Ali Raza Shah, Shazlin Shaharudin, Noga Shalev, Mohammed Shannawaz, Amit Baran Sharangi, Nadim Sharif, Sumaira Sharif, Avimanu Sharma, Bhoopesh Kumar Sharma, Kamlesh Sharma, Ravi Kumar Sharma, Bereket Beyene Shashamo, Mahabalesh Shetty, Aminu Shittu, Km Shivangi, Seyed Afshin Shorofi, Suleiman Adeiza Shuaibu, Harpreet Singh, Jasvinder A Singh, Abhijeet Prasad Sinha, Mejdi Snoussi, Yavuzalp solak, Ahmed M Soliman, Yizhe Song, Aayushi Sood, Anna Maria Spagnolo, Marjia Sultana, Joshua Sumankuuro, Tarun Kumar Suvvari, Ramin Tabibi, Zanan Mohammed-Ameen Taha, Jianye Tan, Rekha Thiruvengadam, Christina S Thornton, Marcos Roberto Tovani-Palone, Nguyen Tran Minh Duc, Munkhtuya Tumurkhuu, Md Jamal Uddin, Irfan Ullah, Lawan Umar, Deepadarshan Urs, Hande Uzunçıbuk, Giacomo Pietro Vigezzi, Tuan Vinh, Yasir Waheed, Xingxin Wang, Tanveer Wani, Nuwan Darshana Wickramasinghe, Mieszko Wieckiewicz, Camillus Abawiera Wongnaa, Yihun Miskir Miskir Wubie, Galal Yahya, Hanwen Yang, Vahit Yiğit, Dong Keon Yon, Naohiro Yonemoto, Fathiah Zakham, Giulia Zamagni, Seema Zargar, Mohammed G M Zeariya, Mohamed Ali Zoromba, and Abdulhakim Mussema Zuber.

Drafting the work or revising it critically for important intellectual content

Hasan Aalruz, Sherief Abd-Elsalam, Nadin M I Abdel Razeq, Jeza Muhamad Abdul Aziz, Abdulrakib Abdulrahim, Khaled M Aboshanab, Nermeen Abu-Elala, Aishah Fadila Adamu, Tajudeen Adesanmi Adebisi, Ayo Stephen Adebowale, Qorinah Estiningtyas Sakilah Adnani, Aanuoluwapo Adeyimika Afolabi, Muhammad Sohail Afzal, Saira Afzal, Gizachew Beykaso Agafari, Percival Delali Agordoh, Salome Wumpini Agordoh, Abdul-Raheem Folorunsho Ahmad, Khabir Ahmad, Muayyad M Ahmad, Ali Ahmed, Ayman Ahmed, Gasha Salih Ahmed, Meqdad Saleh Ahmed, Akil Akhtar, Muhammad Nadeem Akhtar, Olumide Odunayo Akinniyi, Damilare Michael Akintunde, Salah Al Awaidy, Mushtak Talib Salih Al-Ouqaili, Ahmad Rajeh Al-Qudimat, Ali Salman Al-Shami, Raed M Al-Zoubi, Muath A Alammar, Mohammed Albashtawy, Abdelazeem M Algammal, Mohammed Usman Ali, Mohammed Alissa, Alaa A. A. Aljabali, Sameer Abdulmalik Alkubati, Mohammed Z. Allouh, Mohammed Almunef, Khaldoon Aied Alnawafleh, Mohammad R Alqudimat, Najim Z Alshahrani, Abdalkarem Fedgash Alsharari, Zaid Altaany, Awais Altaf, Kassaye D. Demeke Altaye, Nelson Alvis-Guzman, Hany Aly, Nada A. Alzunaidy, Abhishek Anil, Boluwatife Stephen Anuoluwa, Demelash Areda, Muhammad Shahzad Aslam, Maha Moh'd Wahbi Atout, Alok Atreya, Matteo Augello, Usman Ayub Awan, Kemal Lemnuro Awol, Habtamu Molla Ayele, AKM Azad, Ahmed Y Azzam, Syed Babar Jamal Bacha, Riadh Badraoui, Senthilkumar Balakrishnan, Dennis Bardoe, Hiba Jawdat Barqawi, Shazia Batool, Narasimha M Beeraka, Desalegn Weldebrhan Bekru, Samiun Nazrin Bente Kamal Tune, Sonu Bhaskar, Jasvinder Singh Bhatti, Md Mehedi Hasan Bhuiyan, Can Bilgin, Souad Bouaoud, Christopher Boxe, Sayed Muhammad Ata Ullah Shah Bukhari, Yasser Bustanji, Bindiya C C Gowda, Sandip Chakraborty, Imane Chamkhi, Rama Mohan Chandika, Vijay Kumar Chattu, Lam Duc Chau, Akhilanand Chaurasia, Patrick R Ching, Arrigo Francesco Giuseppe Cicero, Bashir Dabo, Bryan Gervais de Liyis, Sindhura Deekonda, Ivan Delgado-Enciso, Vinoth Gnana Chellaiyan Devanbu, Devananda Devegowda, Syed Masudur Rahman Dewan, Giuseppe Di Martino, Marcello Di Pumpo, Ojas Prakashbhai Doshi, Hasan Durmuş, Veli DurmuŞ, Oyewole Christopher Durojaiye, Siddhartha Dutta, Venkata Ravikanth Eddula, Rabie Adel El Arab, Yasser S. El-Sayed, Muhammed Elhadi, Yasir Ahmed Mohammed Elhadi, Chadi Eltaha, Abdelgawad Salah Eltahawy, Simachew Getaneh Endalamew, Collins Nduka Esomchi, Peter Maduabuchi Eze, Adeniyi Francis Fagbamigbe, Ayesha Fahim, Ebenezer Gezahegn Fanta, Aisha Farhana, Olufunmilayo Ibitola Fawole, Getahun Fetensa, Tommaso Filippini, Claudio Fiorilla, Florian Fischer, Aemon Berhane Fissha, Federica Fogacci, Takeshi Fukumoto, Dominic Dormenyo Gadeka, Márió Gajdács, Ezhilkugan Ganessane, Jacopo Garlasco, Rupesh K Gautam, Mebin George Mathew, Ali Gerami Matin, Shumiye Shiferaw Gessess, Ramy Mohamed Ghazy, Syed Abdullah Gilani, Najam Gohar, Yitayal Ayalew Goshu, Bashar Haruna Gulumbe, Sapna Gupta, Awoke Derbie Habteyohannes, Teklehaimanot Gereziher Haile, Raz Mohammed Hama Salih, Mohamed H. Hamad, Abdelrahman M Hamouda, Didem Han Yekdeş, Ashley A Harris, Sara Harsini, Abdul Haseeb, Md Saquib Hasnain, Ibrahim Nagmeldin Hassan, Simon I Hay, Zaw Myo Hein, Abdelaziz Hendy, Md Sabbir Hossain, Segun Emmanuel Ibitoye, Ann Oluchi Ikeabbah, Adalia Ikiroma, Olayinka Stephen Ilesanmi, Irena M Ilic, Milena D Ilic, Korakrit Imwattana, Md. Mojahedul Islam, Aloysia Ispriantari, Mihajlo Jakovljevic, Jazlan Jamaluddin, Jerin James, Bijay Mukesh Jeswani, Nitin Joseph, Charity Ehimwenma Joshua, Fozi Musa Jundi, Dhinesh Babu K, Divya K L, Saltanat Kamenova, Oleksandr Kamyshnyi, Jiseung Kang, Kehinde Kazeem Kanmodi, Reddy Praneeth Karnam, Tomasz M Karpiński, Himanshu Khajuria, Sidra Khalid, Hayat Ahmad Khan, Raees Khan, Ramsha Mushtaq Khan, Yusuf Saleem Khan, Khaled Khatab, Feriha Fatima Khidri, Hyunjee Kim, Min Seo Kim, Daniel Gebrehawaria Kinfe, Shivakumar KM Kondlahalli, Aida Kondybayeva, Vladimir Andreevich Korshunov, Kewal Krishan, Mukhtar Kulimbet, Shweta Kulshreshtha, Dewesh Kumar, Pradeep Kumar, Rakesh Kumar, Abigail Kusi Amponsah, Dian Kusuma, Ville Kytö, Chandrakant Lahariya, Yemane Leake, Caterina Ledda, Hoi Leong Lee, Hwamin Lee, Madeeha Shahzad Lodhi, Peng Luo, Ellina Lytvyak, José Francisco López-Gil, Franciele Maboni Siqueira, Mahmoud Mabrok, Monika Machoy-Rakoczy, Máté Mackei, Edward Augustine Magwe, Sasikumar Mahalingam, Nozad Hussein Mahmood, Tabarak Malik, Bernardo Alfonso Martinez-Guerra, Medha Mathur, Fernanda Penido Matozinhos, Prosanjit Mazumder, Uwakwe C Mba, Agezegn Asegid Mekonnen, Hadush Negash Meles, Tomislav Mestrovic, Muhammad Agus Mikrajab, Nouh Saad Mohamed, Khabab Abbasher Hussien Mohamed Ahmed, Yahaya Mohammed, Ali H Mokdad, Lorenzo Monasta, Jonathan F Mosser, Rohith Motappa, Christopher J L Murray, Ghulam Mustafa, Zuhair S Natto, Biswa Prakash Nayak, Nini Asfaw Negash, Charles Richard James Newton, Cuong Tat Nguyen, Trang Nguyen, Vikram Niranjan, Ali Aahil Noorali, Emira Noumi, Chisom Adaobi Nri-Ezedi, Fred Nugen, Alex Odoom, Felix Akpojene Ogbo, Foluso Olamide Olamide Ojo, Osaretin Christabel Okonji, Michael Chinyem Okonkwo, Lenz Nwachinemere Okoro, Morufat Oluwatosin Olaitan, Atef Oreiby, Thompson Osei, Olutosin Ademola Otekunrin, Amel Ouyahia, Mayowa O Owolabi, Kehinde Adewole Oyeniran, Ilker Ozsahin, Jagadish Rao Padubidri, Chulwoo Park, Sameena Parveen, Shankargouda Patil, Mohamed Anas Patni, Iuliia Pavlova, Zahra Zahid Piracha, Amit Porwal, Ishu Poudyal, Xiang Qi, Igor Radenkov, Fakher Rahim, Bilal Ahmad Rahimi, Md Sazedur Rahman, Jeffrey Pradeep Raj, Gunaseelan Rajendran, Mahmoud Mohammed Ramadan, Kadar Ramadhan, Lakshmi Ramamoorthy, Nemanja Rancic, Mithun Rao, Elrashdy M Redwan, Abdul Rehman, Wajiha Rehman, Yohanes Andy Rias, Luca Ronfani, Parimal Roy, Aly M A Saad, Umar Saeed, Ashok Kumar Sah, Indranil Saha, Fatemeh Saheb Sharif-Askari, Amirhossein Sahebkar, Joseph W Sakshaug, Samreen Saleem, Malik Adebayo Sanusi, Aswini Saravanan, Mukesh Kumar Sathya Narayanan, Subramanian Senthilkumaran, Yashendra Sethi, Disha Sanjivkumar Shah, Liloma Shah, Syed Ali Raza Shah, Shazlin Shaharudin, Amit Baran Sharangi, Nadim Sharif, Sumaira Sharif, Avimanu Sharma, Kamlesh Sharma, Bereket Beyene Shashamo, Mahabalesh Shetty, Pavanchand Shetty, Premalatha K Shetty, Aminu Shittu, Seyed Afshin Shorofi, Suleiman Adeiza Shuaibu, Jasvinder A Singh, Abhijeet Prasad Sinha, Mejdi Snoussi, Yavuzalp solak, Ahmed M Soliman, Yizhe Song, Anna Maria Spagnolo, Marjia Sultana, Joshua Sumankuuro, Tarun Kumar Suvvari, Ramin Tabibi, Zanan Mohammed-Ameen Taha, Jianye Tan, Christina S Thornton, Marcos Roberto Tovani-Palone, Nguyen Tran Minh Duc, Amritha Trikkur Madom Seetharaman, Md Jamal Uddin, Irfan Ullah, Lawan Umar, Hande Uzunçıbuk, Giacomo Pietro Vigezzi, Vignesh Vikram, Tuan Vinh, Yasir Waheed, Xingxin Wang, Tanveer Wani, Nuwan Darshana Wickramasinghe, Mieszko Wieckiewicz, Camillus Abawiera Wongnaa, Galal Yahya, Hanwen Yang, Vahit Yiğit, Dong Keon Yon, Naohiro Yonemoto, Fathiah Zakham, Aurora Zanghì, Seema Zargar, Mohammed G M Zeariya, Basit Zeshan, and Mohamed Ali Zoromba.

Managing the estimation or publications process

Simon I Hay, Min Seo Kim, Ali H Mokdad, Christopher J L Murray, and Dong Keon Yon.
